# Development and validation of predictive models combining cell-Free DNA motifs and protein biomarkers for early detection of esophageal squamous cell carcinoma and precancerous lesion

**DOI:** 10.1186/s40364-025-00840-9

**Published:** 2025-10-14

**Authors:** Liu Ma, Lizhou Dou, Yong Liu, Yueming Zhang, Xudong Liu, Hoi-loi Ng, Jiangtao Chu, Yumeng Liu, Zhengqi Li, Yan Ke, Siyao Liu, Shun He, Guiqi Wang

**Affiliations:** 1 Department of Endoscopy, National Clinical Research Center for Cancer/Cancer Hospital, National Cancer Center, Chinese Academy of Medical Sciences and Peking Union Medical College, Beijing, 100021 China; 2 Breast Disease Center, Shaanxi Provincial Cancer Hospital Affiliated to Xi’an Jiaotong University, Xi’an, 710061 Shaanxi Province China; 3 Department of General Surgery & Obesity and Metabolic Disease Center, China-Japan Friendship Hospital, Beijing, 100029 China

**Keywords:** Early detection, Esophageal squamous cell carcinoma, Liquid biopsy, Multi-omics, Precancerous lesions of the oesophagus

## Abstract

**Background:**

Detecting and treating precancerous lesions can lower the incidence of esophageal squamous cell carcinoma (ESCC), making it a key preventive strategy. Although endoscopic screening and intervention can significantly reduce mortality associated with ESCC, they have certain shortcomings. We aimed to develop three predictive models: the motif, eight-protein, and combined motif-protein models to identify ESCC and its precancerous lesions.

**Methods:**

Plasma samples were collected for cfDNA sequencing, and nine commonly used clinical protein biomarkers related to the digestive system were measured. Using a total cohort of 199 patients with ESCC, 91 patients with esophageal squamous precancerous lesions (ESPL), and 201 controls, we developed an integrative model based on selected multi-omics biomarkers.

**Results:**

The motif-protein model, integrating 20 principal components of cfDNA terminal motifs with six protein features, outperformed both the motif model and the eight-protein model in distinguishing patients with ESCC and ESPL (area under the curve = 0·90). It achieved an overall sensitivity of 88·5% and a specificity of 75·4%. Notably, it successfully identified 90·9% of high-grade intraepithelial neoplasia cases, 86·8% of stage I ESCC cases, and 87·8% of HGIN or T1aN0 stage ESCC (subset of Stage I) cases who were eligible for endoscopic treatment, highlighting its potential as an effective tool for early diagnosis.

**Conclusions:**

The motif-protein model may serve as an effective tool for the early diagnosis of esophageal lesions. Our findings underscore the clinical potential of the multi-omics liquid biopsy test as a non-invasive method for detecting early esophageal lesions.

**Supplementary Information:**

The online version contains supplementary material available at 10.1186/s40364-025-00840-9.

## Introduction

Esophageal cancer is the sixth most common malignancy and the fifth leading cause of cancer-related mortality in China [1]. The asymptomatic nature of early-stage esophageal squamous cell carcinoma (ESCC) often leads to late-stage diagnosis, adversely affecting treatment outcomes and survival rates [2]. Standardised endoscopic screening remains the primary method for early detection, reducing mortality by 34% and incidence by 30% [3, 4]. However, its invasive nature, limited patient tolerance, and high costs restrict its use in large-scale early detection and monitoring programmes.

Non-invasive liquid biopsy technologies have recently gained prominence in cancer detection [5, 6]. The most widely used method involves detecting tumour-associated protein biomarkers in serum or plasma [7]. Common biomarkers for digestive system cancers include squamous cell carcinoma antigen (SCC), cytokeratin 19 fragment (Cyfra21-1), carbohydrate antigen 19 − 9 (CA19-9), carcinoembryonic antigen (CEA), carbohydrate antigen 24 − 2 (CA24-2), cancer antigen 72 − 4 (CA72-4), and alpha-foetoprotein (AFP). Although protein biomarkers provide a simple, cost-effective diagnostic approach, their sensitivity for early-stage esophageal cancer remains low. For example, Kawaguchi et al. reported only 43·9% sensitivity for CYFRA21-1 in diagnosing esophageal cancer [8]. To improve diagnostic accuracy, studies have explored biomarker combinations. Bagaria et al. and Zheng et al. found that combining CEA, CYFRA21-1, and SCC improved specificity to 88·3% for ESCC diagnosis, though the sensitivity remained at 67·3% [9, 10]. These findings highlight the need for more sensitive and specific biomarkers and improved detection strategies.

Circulating cell-free DNA (cfDNA) consists of DNA fragments released from cells into the bloodstream. In patients with cancer, a portion of plasma cfDNA originates from tumour cells, carrying tumour-specific genetic alterations [11]. cfDNA also contains valuable information related to nuclease activity and tissue origin. Fragmentation by various nucleases produces cfDNA end motifs with tumour-specific characteristics [12]. As promising tumour biomarkers, cfDNA end motifs demonstrate strong diagnostic potential, requiring only a small DNA sample for analysis, making them ideal for liquid biopsy applications [13, 14]. Research shows that tumour-associated cleavage sites are more prevalent and easier to detect than mutations [15]. While cfDNA fragmentomics has been effectively used for non-invasive detection of hepatocellular carcinoma [16, 17] and lung cancer [18, 19], predictive models using cfDNA end motifs for esophageal cancer remain largely unexplored.

Multi-omics analysis integrates data from multiple biological layers—ranging from genes to proteins, metabolites, and cellular processes—to uncover complex mechanisms underlying diseases. In esophageal cancer research, multi-omics analysis has facilitated the identification of novel biomarkers, clarified molecular pathways, defined tumour subtypes, guided personalised treatments, and contributed to therapeutic development [20–25]. While multi-omics approaches using genomic fragments and non-invasive techniques have been applied in cancers—such as hepatocellular carcinoma [16, 17, 26] and lung cancer [18, 19], their use in the early detection of esophageal cancer remains limited. For example, combining cfDNA mutation analysis with whole-genome methylation profiling has shown promise in hepatocellular carcinoma screening [27]. However, there is a notable lack of research on multi-omics predictive models for early screening and diagnosis of esophageal cancer and its precancerous lesions. Advances in cfDNA detection technologies, combined with multi-omics analyses, offer new opportunities to enhance early diagnosis and monitoring, offering a promising direction for future advancements.

This study identified differential biomarkers for ESCC and its precancerous lesions using cfDNA sequencing and clinically relevant protein biomarkers of the digestive system. Predictive models were developed using cfDNA end motifs and tumour-associated protein biomarkers through bioinformatics and machine learning techniques. A novel multi-omics predictive model was developed, integrating cfDNA end motifs with protein biomarker data, representing an innovative approach to enhancing predictive accuracy and understanding complex biological interactions. The performance of these models was systematically evaluated and compared to assess their effectiveness in early esophageal cancer detection and its precancerous stages. Furthermore, we explored the potential of these models for large-scale screening, aiming to enhance early detection and improve clinical outcomes.

## Methods

### Study participants

From December 2021 to March 2022, whole-blood samples were collected from eligible patients at the Endoscopy Department of the Cancer Hospital, Chinese Academy of Medical Sciences. The cohort included patients with ESCC, precancerous lesions, benign lesions, and healthy individuals who underwent routine check-ups. Of the 359 plasma samples collected, nine samples were excluded due to imaging or pathological findings indicating malignancies outside the oesophagus and seven samples due to incomplete clinical TNM staging. Ultimately, 343 plasma samples were included for cfDNA library construction and protein biomarker analysis, forming the basis for predictive model development. From September 2022 to January 2023, additional whole-blood samples were prospectively collected from eligible participants to serve as the testing set for model performance validation. A total of 157 plasma samples were collected, of which seven were excluded, resulting in a final dataset of 49% training data (*n* = 240), 21% validation data (*n* = 103), and 30% test data (*n* = 148).

This study adhered to the ethical principles of the Declaration of Helsinki and received approval from the Ethics Committee of the Cancer Hospital, Chinese Academy of Medical Sciences (Approval No.: 21/249–2920). Informed consent was obtained from all participants.

### Sample collection and plasma separation

Before undergoing endoscopy, participants fasted and abstained from drinking water. Researchers collected basic information and personal histories and completed case report forms. For eligible participants, 8–10 mL of fasting peripheral venous blood was drawn into disposable cfDNA preservation tubes and processed within 4 h. The sample processing protocol was as follows:

Samples were centrifuged at 1900 × g for 10 min at room temperature to separate plasma from blood cells. The supernatant was transferred to a sterile EP tube. Second Centrifugation: Plasma was centrifuged again at 3000 × g for 15 min to further purify the sample. The upper plasma layer was divided into 1 mL aliquots in sterile EP tubes. The aliquoted plasma samples were immediately transported on dry ice and stored at -80 °C for future analysis.

### CfDNA library preparation, sequencing, data alignment, and quality control

The concentration and quality of cfDNA were assessed after its isolation and extraction. Library preparation was performed using the Qiagen QIAseq cfDNA All-in-One Kit (Qiagen, Hilden, Germany). The amplified products underwent cDNA purification and fragment selection with QMN magnetic beads. The Qubit 1×dsDNA assay kit (Thermo Fisher Scientific, Waltham, MA, USA) was used to quantify the cfDNA library, and the Qsep-400 capillary electrophoresis system (BiOptic, Taipei City, Taiwan) was used for library fragment quality control. All libraries were sequenced on MGI 2000 and MGI T7 platforms (BGI, Shenzen, China) using the paired-end 100 bp (PE100) sequencing mode. The sequencing depth for cfDNA libraries was 10G per sample. The proportion of bases with a quality score ≥ 30 (Q30) averaged above 90%, ensuring high sequencing quality (Table S1).

Adapters were removed from the raw FASTQ files, and low-quality reads were filtered to obtain clean reads. The filtered reads were aligned to the human genome (hg19). Following alignment, the data underwent deduplication, sorting, and index generation to produce the BAM files. Quality control checks assessed the alignment rate, average genome coverage depth, and fragment size to ensure data integrity. Once confirmed, the sorted BAM files were used to extract the nucleotide sequences of the last four bases of the cfDNA fragments. The count of each four-base end sequence was recorded, and the ratio of each sequence count to the average count of all four-base sequences was calculated to form the end-motif matrix. The matrix was standardised using Z-score normalisation to ensure a standard normal distribution.

### Detection of tumour-associated protein biomarkers

Tumour-associated protein biomarkers, including AFP, CA19-9, CA24-2, CA72-4, CEA, Cyfra21-1, SCC, PG I, and PG II, were measured using reagent kits based on a chemiluminescence immunoassay from DIRUI Corporation and a fully automated chemiluminescence immunoassay system (CM-180, DIRUI Corporation, Jilin, China). The detection procedure was performed according to the manufacturer’s instructions, and the results were recorded.

### Construction and validation of the CfDNA motif model

The model was constructed using the training dataset. Differential analysis was performed on the Z-score standardised data from the esophageal cancer, precancerous lesion, and control groups, identifying 203 significant differential end motifs (t-test, *p* < 0·05). Principal component analysis (PCA) reduced these features to 30 dimensions. Subsequently, 10-fold cross-validation and random forest recursive feature elimination (RF-RFE) were applied to select 16 optimal feature dimensions.

A random forest classification model was developed using 10-fold cross-validation to ensure robust performance. Then, the validation set was used to evaluate the model, and the optimal threshold was determined based on Youden’s index. Finally, a test set validated the model’s effectiveness.

### Construction and validation of the protein model

Ten clinically relevant protein biomarkers—AFP, CA19-9, CA24-2, CA72-4, CEA, Cyfra21-1, SCC, PG I, PG II, and the PG I/PG II ratio (PGR)—were measured. In the training set, RF-RFE was used to identify the optimal combination of the eight protein biomarkers.

Using these eight selected biomarkers, a random forest classification model was developed with 10-fold cross-validation to ensure optimal performance. The validation set was used to evaluate the performance of the model, and the optimal threshold was determined. Finally, the testing set was used to assess the model’s ability to classify positive cases across different groups, including precancerous lesions and ESCC, based on the established threshold.

### Construction and validation of the combined motif-protein model

The protein biomarker results and Z-score-standardised cfDNA motif data from 491 samples were matched for analysis. In the training set, the 30 principal components obtained through PCA dimensionality reduction, together with the 10 protein biomarkers, were subjected to RF-RFE to identify the optimal feature set, consisting of 20 principal components and six protein biomarkers.

Using these selected features, a random forest classification model was developed using 10-fold cross-validation to ensure optimal performance. The validation set was used to evaluate the model’s performance, and the optimal threshold was determined. Finally, the testing set was employed to assess the model’s effectiveness and generalisation capability by classifying positive cases based on the established threshold.

### Statistical analysis

Statistical analyses and data visualisation were performed using R software (version 4·2·0; R Software for Statistical Computing, Vienna, Austria) and Python (version 3·8·0). Continuous variables are expressed as medians with interquartile ranges, while categorical variables are presented as numbers with percentages and compared using chi-square tests or continuity-corrected chi-square tests.

Independent-sample t-tests or Wilcoxon rank-sum tests were used for two-group comparisons of numerical variables, while the Kruskal–Wallis test was used for comparisons across three groups. Receiver operating characteristic (ROC) curves were plotted using the R package pROC (version 13·18·0) to calculate the area under the curve (AUC), along with sensitivity, specificity, and 95% confidence intervals (CIs) for model performance evaluation. Statistical significance was determined using two-tailed tests with α = 0·05.

AUCs were compared using the DeLong test. The optimal threshold for each model was determined using Youden’s indices.

## Results

### Baseline characteristics of the 491 enrolled participants

**Fig. 1:**
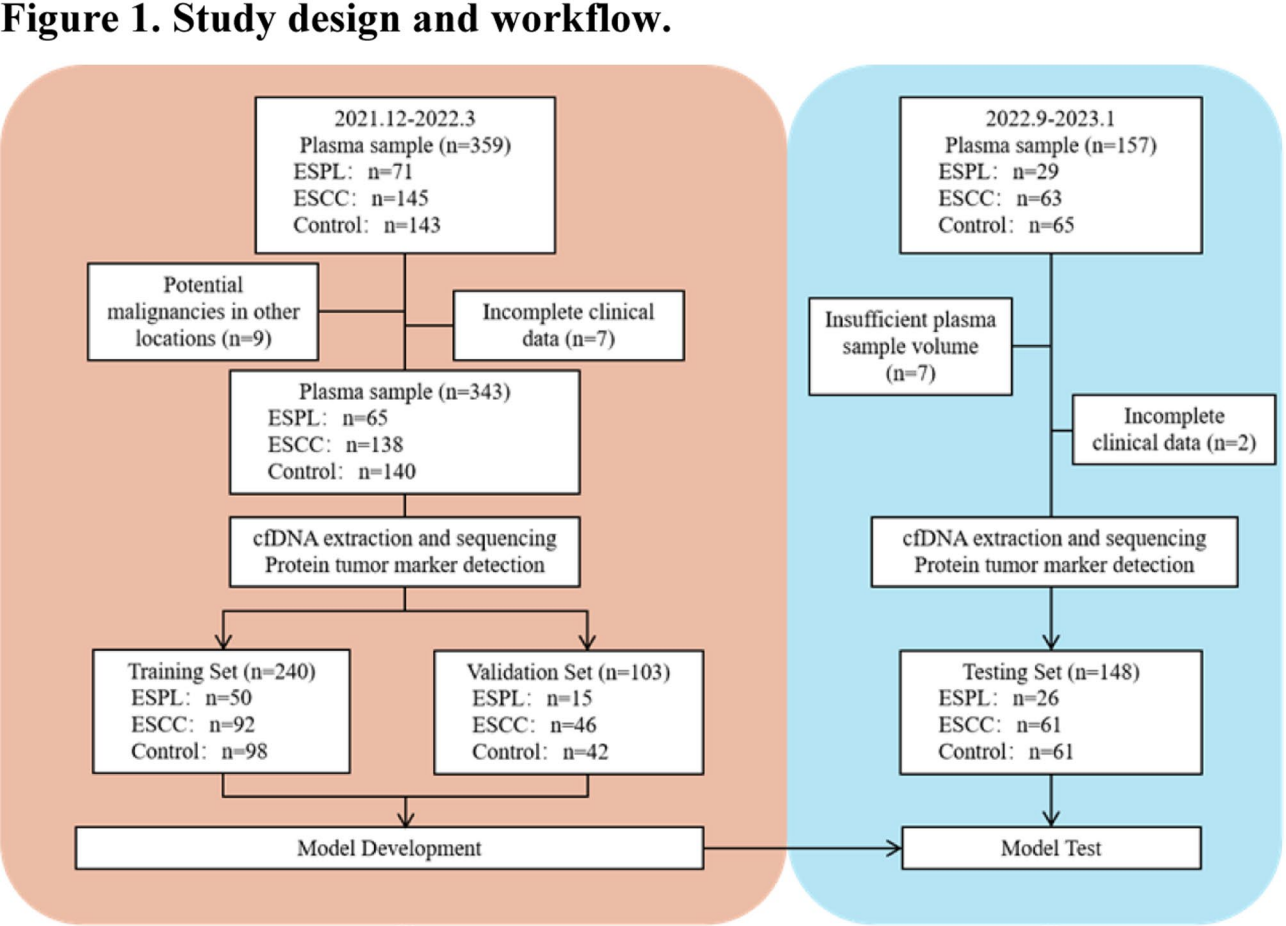
Study design and workflow. This study was conducted in two phases, with a total of 491 plasma samples collected for cfDNA library construction and protein biomarker detection, followed by predictive model development and validation. Patients with esophageal high-grade intraepithelial neoplasia (HGIN) and low-grade intraepithelial neoplasia (LGIN) were classified as the ESPL group (*n* = 91), while patients with ESCC were assigned to the ESCC group (*n* = 199), and individuals with benign esophageal conditions and healthy controls formed the control group (*n* = 201)

This study was conducted in two phases (Fig. 1), with 491 plasma samples collected for cfDNA library construction, protein biomarker detection, and subsequent predictive model development and validation. Patients with esophageal high-grade intraepithelial neoplasia (HGIN) and low-grade intraepithelial neoplasia (LGIN) were classified into the esophageal squamous precancerous lesion (ESPL) group (*n* = 91), patients with ESCC were assigned to the ESCC group (*n* = 199), and individuals with benign esophageal conditions and healthy controls formed the control group (*n* = 201).

The median age of patients in the ESCC group was 63 years, with a predominance of males (86·9%), smoking history (42·7%), and history of alcohol consumption (40·7%). In the precancerous lesion group, the median age was 62 years, with most patients being male (69·2%), non-smokers (73·6%), and non-drinkers (68·1%). The median age of the control group was 52 years, predominantly females (60·7%), non-smokers (89·6%), and non-drinkers (84·1%). Among the 491 samples, 91 cases of precancerous lesions and 124 cases of stage I esophageal cancer accounted for 43·8% of the total participants, demonstrating the cohort’s strong representation of early-stage esophageal cancer and precancerous lesions.

Table 1 presents the clinical and pathological characteristics of the participants after the datasets were randomly divided, along with statistical comparisons between them. The results showed no significant differences among the training, validation, and test datasets in terms of age (*p* = 0·576), sex (χ²=0·93, *p* = 0·628), smoking history (χ²=4·50, *p* = 0·343), alcohol consumption history (χ²=2·13, *p* = 0·711), group distribution (χ²=7·26, *p* = 0·509), or subgroup distribution (χ²=11·90, *p* = 0·614).

**Table 1:** Statistical values of clinicopathological characteristics of participants in each dataset

Clinicopathological Characteristics		Training Set (*n* = 240)	Validation Set (*n* = 103)	Testing Set (*n* = 148)	Statistical Value	*p*-value
Age, Median		60 (53, 66)	59 (52, 66)	59 (49, 66)	1·10	0·576^a^
Sex, n (%)					0·93	0·628^b^
	Male	149 (62·1)	69 (67·0)	97 (65·5)		
	Female	91 (37·9)	34 (33·0)	51 (34·5)		
Smoking History, n (%)					4·50	0·343^b^
	Smoker	55 (22·9)	34 (33·0)	36 (24·3)		
	Non-Smoker	160 (66·7)	62 (60·2)	98 (66·2)		
	Unknown	25 (10·4)	7 (6·8)	14 (9·5)		
Drinking History, n (%)					2·13	0·711^b^
	Drinker	63 (26·3)	33 (32·0)	41 (27·7)		
	Non-Drinker	151 (62·1)	63 (61·2)	92 (62·2)		
	Unknown	26 (10·8)	7 (6·8)	15 (10·1)		
Group, n (%)					7·26	0·509^b^
	ESPL	50 (20·8)	15 (14·6)	26 (17·6)		
	Stage I–II ESCC	62 (25·8)	38 (36·9)	47 (31·8)		
	Stage III–IV ESCC	30 (12·5)	8 (7·8)	14 (9·5)		
	Health	15 (6·3)	7 (6·8)	7 (4·7)		
	Benign	83 (34·6)	35 (34·0)	54 (36·5)		
Subgroup, n (%)					11·90	0·614^c^
	LGIN	23 (9·6)	8 (7·8)	15 (10·1)		
	HGIN	27 (11·3)	7 (6·8)	11 (7·4)		
	Stage I ESCC	51 (21·3)	35 (34·0)	38 (25·7)		
	Stage II ESCC	11 (4·6)	3 (2·9)	9 (6·1)		
	Stage III ESCC	21 (8·8)	6 (5·8)	8 (5·4)		
	Stage IV ESCC	9 (3·8)	2 (1·9)	6 (4·1)		
	Health	15 (6·3)	7 (6·8)	7 (4·7)		
	Benign	83 (34·6)	35 (34·0)	54 (36·5)		

### Selection of differential CfDNA end motifs and tumour protein biomarkers

The end-motif matrices of the ESCC, ESPL, and control groups were subjected to Z-score normalisation. In the training set (*n* = 240), independent-sample t-tests were performed for differential analysis, identifying 203 end motifs with significant differences between the groups (Figure S1). Among these, 128 end motifs were significantly downregulated in patients with ESCC and precancerous lesions, whereas 75 motifs were significantly upregulated.

Hierarchical clustering analysis of the differentially expressed end motifs showed that the 203 selected features effectively distinguished samples from the ESCC and ESPL groups from those of the control group (Figure S2).

Similarly, 10 clinically relevant protein biomarkers for digestive diseases (AFP, CA19-9, CA24-2, CA72-4, CEA, Cyfra21-1, SCC, PG I, PG II, and PGR) were analysed in the training set using independent-sample t-tests to evaluate differences between the combined ESCC and ESPL groups and the control group. As shown in Figure S1, five protein biomarkers (CEA, Cyfra21-1, PG I, PG II, and PGR) with significant intergroup differences were identified, all of which were upregulated in ESCC and precancerous lesions.

### Construction and validation of the CfDNA end-motif model

**Fig. 2:**
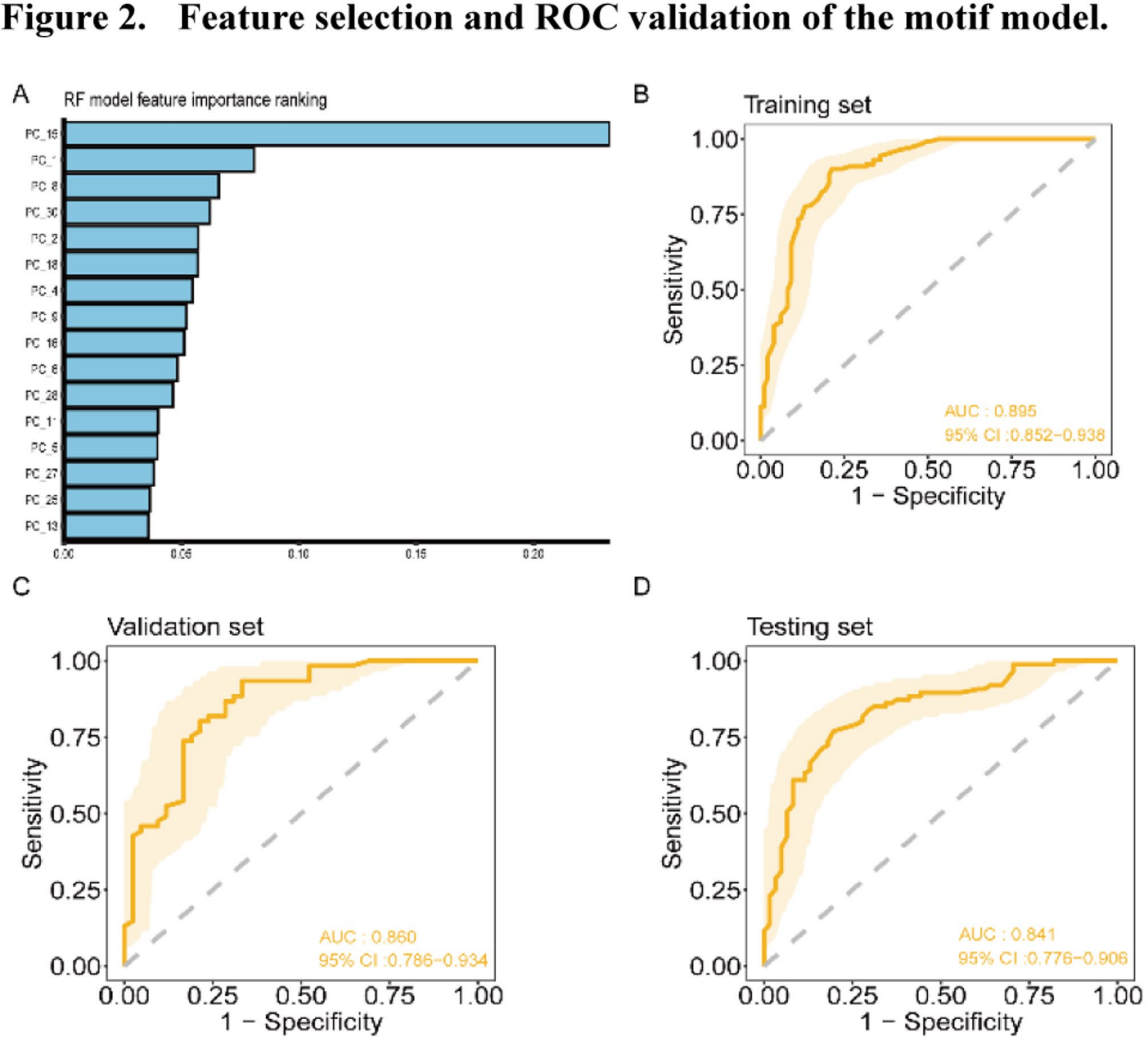
Feature selection and ROC validation of the motif model. **a**: The importance ranking of the top 16 principal component features in constructing the motif model. **b–d**: ROC test results of the motif model in the training (**b**), validation (**c**), and testing (**d**) set

PCA was applied to the 203 differentially identified end motifs, reducing them to 30 dimensions representing combinations of the original motifs. Ten-fold cross-validation and RF-RFE were performed simultaneously, resulting in 16 optimal feature dimensions. As shown in Fig. 2a, the ranked contributions of the 16 principal components to the model were identified, with PC15 contributing the most.

Using these selected principal components, the random forest motif model was further refined through ten-fold cross-validation to ensure optimal performance. The finalised model achieved average AUC values of 0·89, 0·86, and 0·84 in the training, validation, and testing sets, respectively, confirming the diagnostic performance of the motif model across datasets (Fig. 2b–d).

The optimal threshold for the motif model was 0·39 based on the Youden index using the validation set. The model’s performance was evaluated across the datasets, and the results are summarised in Table S2. The motif model achieved a sensitivity of 100% for detecting precancerous lesions in both the training and validation sets. In the testing set, the sensitivity for the precancerous lesion group was 96·2% (95% CI: 88·8%–100%), with 93·3% (95% CI: 80·7%–100%) for LGIN and 100% for HGIN, demonstrating the model’s excellent diagnostic capability for precancerous conditions. In a subgroup analysis of HGIN + T1aN0 ESCC, which is clinically eligible for endoscopic treatment, the model achieved a sensitivity for positive detection of 97·1% (95% CI: 93·2%–100%) in the training set, 94·1% (95% CI: 86·2%–100%) in the validation set, and 90·2% (95% CI: 81·2%–99·3%) in the testing set. These findings confirm the model’s strong potential for early diagnosis and clinical applications.

### Construction and validation of the protein biomarker model

**Fig. 3:**
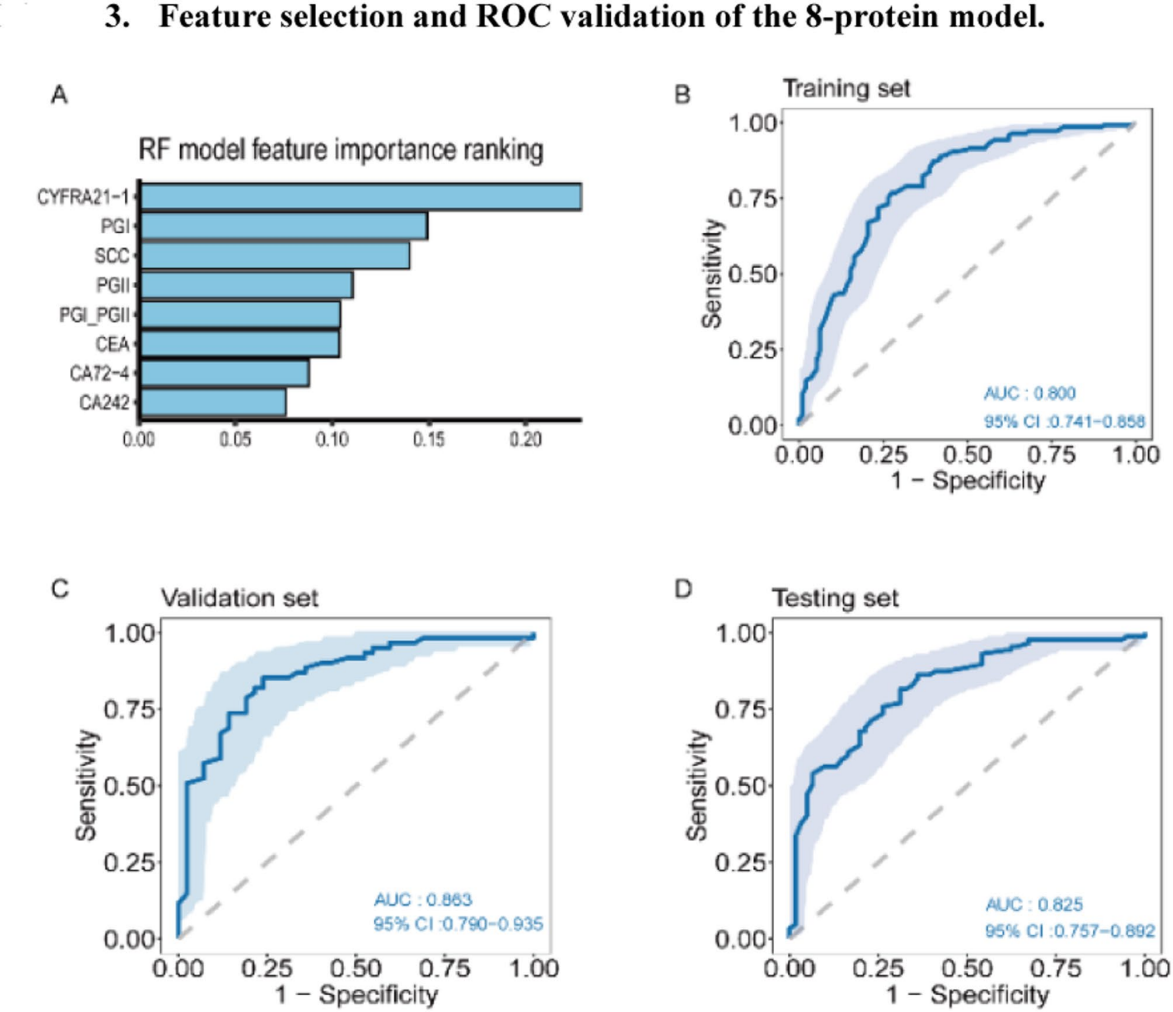
Feature selection and ROC validation of the 8-protein model. **a**: The importance ranking of eight tumour protein biomarkers in constructing the proteins model. **b–d**: ROC test results of the eight-protein model in the training (b), validation (c), and testing (d) set

RF-RFE was applied to the 10 digestive system-related protein biomarkers in the training set (*n* = 240) to identify eight optimal protein features. Using these eight biomarkers (CA24-2, CA72-4, CEA, Cyfra21-1, SCC, PG I, PG II, and PGR), a random forest model, referred to as the eight-protein model, was constructed using ten-fold cross-validation. Figure 3a presents the contribution ranking of the eight biomarkers, with Cyfra21-1, PG I, and SCC being identified as the top three contributors. The ROC curves demonstrated that the eight-protein model effectively distinguished plasma samples from the ESCC and precancerous lesion groups from those of the control group across all three datasets. The average AUCs for the training, validation, and testing sets were 0·80, 0·86, and 0·82, respectively, indicating good diagnostic performance (Fig. 3b–d).

Using the validation set, the optimal threshold for the eight-protein model was 0·55 based on the Youden index. Table S3 presents the sensitivities and specificities of the models for the three datasets. Analysis of the results in Table S3 revealed that, consistent with the findings from the training and validation sets, the sensitivity of the eight-protein model in the testing set increased with the progression of esophageal disease from LGIN to HGIN to early-stage ESCC. The model achieved an average sensitivity of 65·4% in the precancerous lesion group and 88·5% in all esophageal cancer cases. Notably, in the subgroup of patients with HGIN + T1aN0 ESCC, who had clinical indications for endoscopic surgery, the model’s sensitivity reached 80·5% (95% CI: 68·4%–92·6%).

In all three datasets, the eight-protein model demonstrated superior diagnostic performance in identifying patients with ESCC and ESPL compared with individual tumour protein biomarkers, including CA24-2, CA72-4, CEA, SCC, PG I, PG II, and PGR (Figure S3). Although the AUC of Cyfra21-1 was not significantly different from that of the eight-protein model across datasets, the sensitivity of Cyfra21-1 (62·1%, 95% CI: 51·9%–72·3% in the testing set) was lower than that of the eight-protein model (81·6%, 95% CI: 73·5%–89·7% in the testing set).

### Construction and validation of the combined motif-protein model

**Fig. 4:**
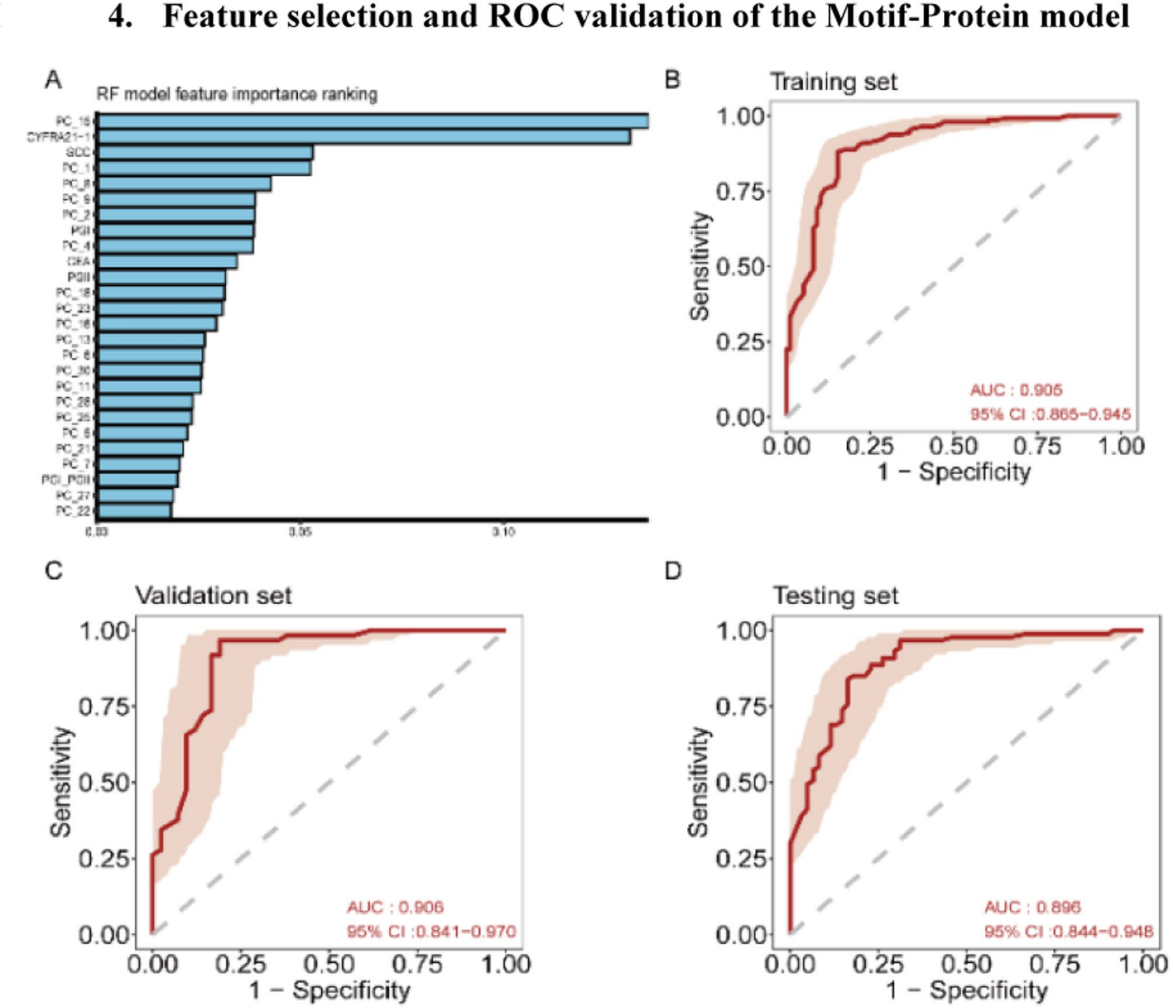
Feature selection and ROC validation of the Motif-Protein model. **a**: The importance ranking of six tumour protein biomarkers and the top 20 principal components in constructing the Motif-Protein model. **b–d**: ROC test results of the motif-Protein model in the training (b), validation (c), and testing (d) set

The Z-score-normalised cfDNA end-motif data and protein biomarker results from 491 samples were matched, with the same random division into three datasets as used for the motif model. PCA was applied to the training set, processing the 30 end-motif dimensions and 10 protein biomarkers using RF-RFE, which selected 20 optimal end-motif features and six protein biomarkers (Fig. 4a). The combined motif-protein model was developed using 10-fold cross-validation of the training set. The average AUCs of the Motif-Protein model in the training, validation, and testing sets were 0·90, 0·91, and 0·90, respectively, indicating superior performance in distinguishing patients with ESCC and precancerous lesions from the control group compared to the individual motif and eight-protein models (Fig. 4b–d).

The Youden index (0·50) from the validation set was used to optimise the model threshold, and its diagnostic performance was evaluated in terms of sensitivity and specificity (Table S4). The motif-protein model achieved 100% sensitivity for diagnosing stage II, III, and IV ESCC in both the validation and testing sets. In the testing set, the model correctly identified 90·9% (95% CI: 73·9%–100%) of HGIN cases, 86·8% (95% CI: 76·1%–97·6%) of stage I ESCC cases, and 87·8% (95% CI: 77·8%–97·8%) of HGIN or T1aN0-stage ESCC cases, underscoring its potential as a promising tool for early diagnosis.

### Comparison of diagnostic performance: motif-protein model, motif model, eight-protein model, and individual protein biomarkers

We conducted a comparative analysis of the AUC, sensitivity, and specificity of the three constructed models, along with individual protein biomarkers, to distinguish ESCC and precancerous lesions from the control group (Tables S5, S6, and 2).

The results indicated no significant differences in the AUC values among the three models in the testing set (all *p* > 0·05, Table 2). Among the models, the motif-protein model exhibited the best overall balance between sensitivity and specificity. The motif model achieved the highest average sensitivity (89·7%) for identifying high-risk individuals, and in the testing set, the AUC of the motif-protein model was significantly higher than the AUCs of all eight individual protein biomarkers (all *p* < 0·05), highlighting the superior diagnostic performance of the motif-Protein model in distinguishing ESCC and ESPL samples from control samples.

**Table 2:** Comparison of the detection performance of the motif-protein model, motif model, eight-protein model, and protein markers in the testing set

Testing Set	Sensitivity (%) 95% CI	Specificity (%) 95% CI	AUC	Threshold	*p*-value^a^	*p*-value^b^
Motif-Protein Model	88·5 (81·8–95·2)	75·4 (64·6–86·2)	0·90	0·50	1·000	0·101
Motif Model	89·7 (83·3–96·1)	55·7 (43·3–68·2)	0·84	0·39	0·195	0·705
Eight-protein Model	81·6 (73·5–89·7)	68·9 (57·2–80·5)	0·82	0·55	0·101	1·000
CEA	33·3 (23·4–43.2)	90·2 (82·7–97·6)	0·71	2·21	< 0·001	0·028
SCC	70·1 (60·5–79·7)	67·2 (55·4–79·0)	0·73	0·65	0·001	0·026
CA19-9	24·1 (15·1–33·1)	72·1 (60·9–83·4)	0·53	10·95	< 0·001	< 0·001
CYFRA21-1	62·1 (51·9–72·3)	83·6 (74·3–92·9)	0·80	1·^3^3	0·036	0·273
CA24-2	20·7 (12·2–29·2)	72·1 (60·9–83·4)	0·56	4·56	< 0·001	< 0·001
PG I	60·9 (50·7–71·2)	70·5 (59·0–81·9)	0·74	45·44	0·001	0·025
PG II	52·9 (42·4–63·4)	73·8 (62·7–84·8)	0·69	6·95	< 0·001	0·002
PG I/PG II (PGR)	37·9 (27·7–48·1)	85·2 (76·3–94·1)	0·60	9·76	< 0·001	< 0·001

^a^ Comparison with the Motif-Protein model calculated using DeLong’s test

^b^ Comparison with the eight-protein model calculated using DeLong’s test

**Fig. 5:**
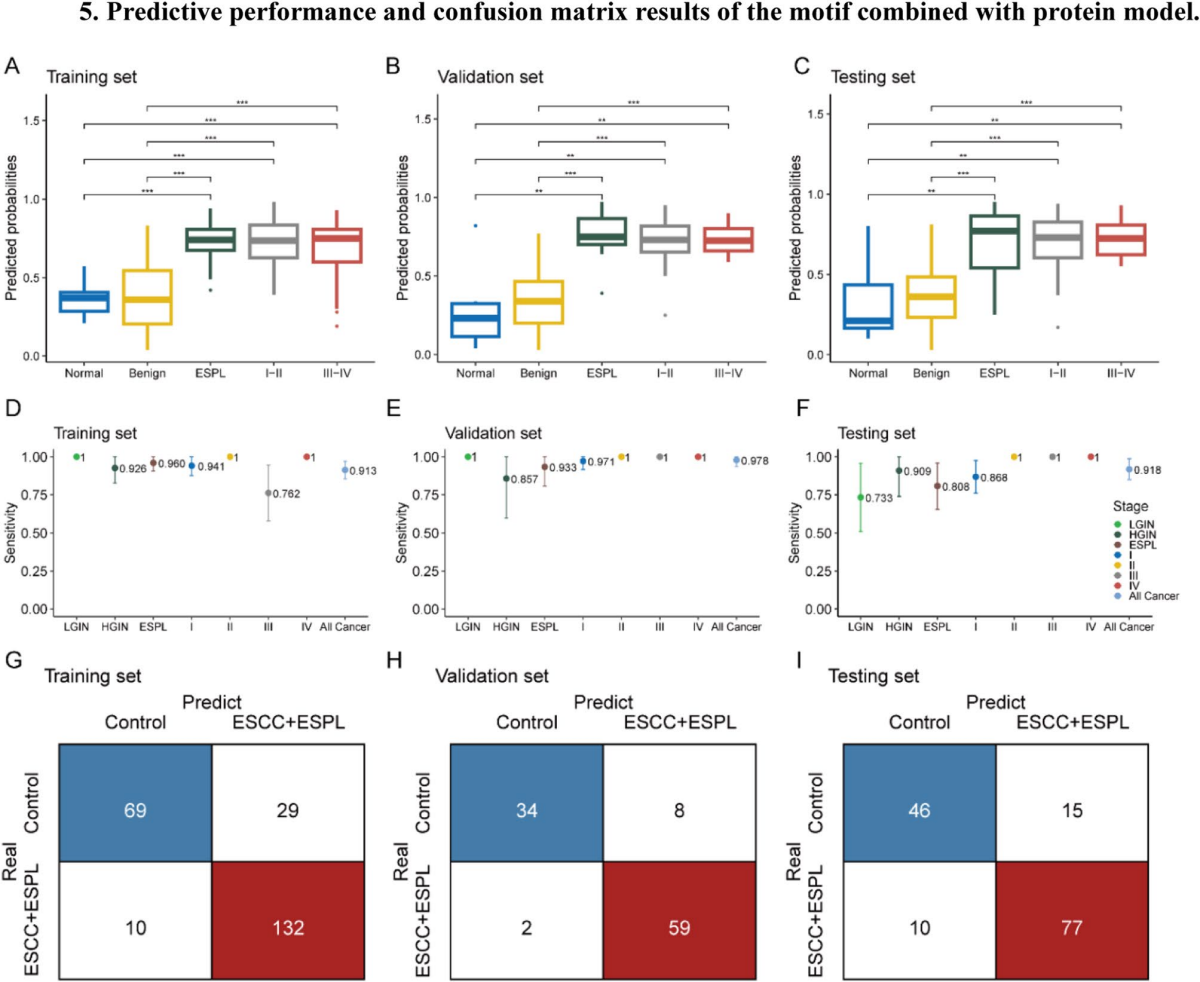
Predictive performance and confusion matrix results of the motif combined with protein model. **a–c**: Model prediction scores for healthy controls, benign esophageal diseases, precancerous lesions, stage I-II, and stage III–IV ESCC in the training (a), validation (b), and testing (c) sets. **d–f**: Sensitivity of the model’s predictions for LGIN, HGIN, stage I, II, III, IV ESCC subgroups, and all ESCC and precancerous lesions in the training (d), validation (e), and testing (f) sets. **g–i**: Confusion matrix results of the model predictions in the training (g), validation (h), and testing (i) sets

This study further explored the earliest detectable stages of esophageal lesions using risk prediction scores generated by the motif-protein model. The results showed that the model prediction scores effectively identified patients with esophageal precancerous lesions, stage I–II ESCC, and stage III–IV ESCC (Fig. 5a–c). The model demonstrated good sensitivity across all subgroups (Fig. 5d–f), with a higher overall sensitivity in the cancer group than in the precancerous lesion group (Fig. 5e, f). The confusion matrix results further confirmed the model’s ability to accurately predict and distinguish individuals at high risk of ESCC (Fig. 5g–i).

## Discussion

Endoscopic screening and intervention in patients with esophageal cancer can significantly reduce mortality associated with the disease, and detecting and treating precancerous lesions can lower its incidence. The development of appropriate early screening methods for treatable lesions offers promising opportunities to reduce ESCC mortality [4]. By improving patient compliance and screening efficiency through non-invasive liquid biopsy methods, detection and early diagnosis rates can be increased. This approach aims to reduce esophageal cancer mortality in the Chinese population and ultimately lower the incidence of the disease [3].

This study analysed plasma cfDNA and clinically relevant digestive system protein biomarkers in 491 patients with ESCC, precancerous lesions, and a control group. Differentially expressed biomarkers were identified at both the genomic and proteomic levels in ESCC and precancerous lesions. Two predictive models were constructed using a random forest model based on this content. We developed an innovative multi-omics predictive model by combining differential cfDNA end motifs with protein biomarkers. All three models demonstrated strong diagnostic performance for ESCC and its precancerous lesions, with AUCs exceeding 0·82, confirming their potential for early diagnosis and screening of esophageal cancer. This study highlights the utility of machine learning-based multi-omics analysis for early diagnosis and monitoring of ESCC, offering a novel approach for non-invasive cancer screening.

Previous studies on esophageal cancer screening models based on genomic detection are limited, particularly concerning precancerous lesions. For instance, Qiao et al. included only five cases of stage 0 ESCC in their model [28], whereas Tian et al. analysed differential hydroxy methylation in only four HGIN cases [29]. Most studies have not incorporated precancerous lesions during model construction or performance evaluations. In contrast, our study included 46 LGIN, 45 HGIN, 100 T1aN0 ESCC, and 24 T1bN0 ESCC cases, accounting for 43·8% of the total sample size. This makes it one of the most comprehensive studies to incorporate both precancerous lesions and early-stage esophageal cancer into a single cancer screening model. The inclusion of precancerous samples during feature selection provided a significant sensitivity advantage for detecting early-stage ESCC. Regarding early-stage ESCC prediction, Qiao et al.‘s cfDNA methylation-based model had a sensitivity of only 50·0% for stage 0 ESCC and 58·8% for stage 0–II ESCC [28]. Similarly, Klein et al. reported only 12·5% sensitivity (*n* = 8) for stage I ESCC in their pan-cancer cfDNA methylation model [30]. In contrast, our motif model demonstrated superior sensitivity in detecting precancerous lesions, with sensitivities of 93·3% for LGIN, 100% for HGIN, and 86·8% for stage I ESCC. The motif-protein model correctly identified 73·3% of the LGIN cases, 90·9% of the HGIN cases, and 86·8% of the stage I ESCC cases. Moreover, in diagnosing HGIN and T1aN0 ESCC, which are clinically eligible for endoscopic treatment, the average sensitivities of the three models were 90·2% (motif model), 87·8% (motif-protein model), and 80·5% (eight-protein model), further highlighting the superior sensitivity of our models for detecting ESCC and its precancerous lesions.

In distinguishing all ESCC cases from the control group, the motif model achieved a sensitivity of 86·9% and specificity of 55·7%; the eight-protein model demonstrated a sensitivity of 88·5% and specificity of 68·9%, while the motif-protein model exhibited the highest sensitivity (91·8%) and specificity (75·4%). The higher sensitivity of our models indicates a clear advantage in identifying ESCC, although it also suggests the potential for false positives. Given that the primary goal of our models is to identify individuals at high-risk individuals for esophageal cancer and reduce unnecessary endoscopic examinations, those who test positive will undergo further endoscopy and pathological biopsy, with pathology serving as the diagnostic gold standard for ESCC and precancerous lesions. Participants who test negative will continue regular follow-ups and non-invasive blood-based monitoring. Therefore, the higher sensitivity of our liquid biopsy models helps minimise the risk of missed diagnoses among high-risk populations, especially in the absence of routine endoscopic screening.

In the field of multi-omics detection models, this study represents the most extensive single cancer screening investigation for ESCC, incorporating the largest number of precancerous lesions and early-stage esophageal cancer cases to date. Previous studies have indicated that combining multi-omics biomarkers or integrating different detection technologies, such as protein-DNA mutations, RNA expression, and genomic alterations, can significantly enhance the sensitivity of liquid biopsies for early cancer diagnosis [26, 31, 32]. Our study confirmed that the motif-protein model, which integrates end-motif and protein detection, exhibited the best diagnostic performance among the five models constructed, with an AUC of 0·90, overall sensitivity of 88·5%, and specificity of 75·4%. Cohen et al. developed CancerSEEK, a multi-component blood test that combines genomic mutations and protein biomarkers using artificial intelligence [32]. CancerSEEK was capable of organ-of-origin identification and detection across eight cancer types (ovarian, liver, gastric, pancreatic, esophageal, colorectal, lung, and breast cancers), with an overall sensitivity of 69–98% and a specificity of 99%. However, the sensitivity for esophageal cancer was only approximately 70%, and the sensitivity for stage I esophageal cancer was the lowest among the eight cancers, at just 20%. By comparison, in the detection of early esophageal lesions, our motif-protein model achieved a sensitivity of 86·8% for stage I ESCC and correctly identified 90·9% of HGIN cases as positive, demonstrating superior early-stage predictive performance.

Previous research and clinical practice have indicated that single-protein tumour biomarkers generally exhibit limited sensitivity for predicting esophageal cancer, particularly in early-stage cases. For example, using a threshold of 1·40 ng/mL, Brockmann et al. reported an overall sensitivity of 36% for esophageal cancer (45·5% for squamous cell carcinoma) and a specificity of 97·3% for Cyfra21-1 [33]. Similarly, Kawaguchi et al. found a sensitivity of only 43·9% for Cyfra21-1 in diagnosing esophageal cancer. In our study, Cyfra21-1, a known biomarker for esophageal cancer, demonstrated high specificity (83·6%) but relatively low sensitivity (62·1%) at a threshold of 1·33 ng/mL, reinforcing its limited utility for the early detection of ESCC and precancerous lesions. However, our eight-protein model outperformed previously reported models combining CEA, Cyfra21-1, and SCC [10], achieving a higher sensitivity (81·6% vs. 67·3%). Although Cyfra21-1 alone is insufficiently sensitive to determine cancer status, its AUC of 0·80 showed no significant difference compared to the eight-protein model, suggesting it still holds value as a single biomarker for identifying ESCC and precancerous lesions. Therefore, Cyfra21-1 may serve as an adjunct to the eight-protein model. When a positive result is identified by the eight-protein model, a subsequent negative Cyfra21-1 test could refine risk stratification, improve the true-negative rate, and enhance the utility of the eight-protein model for early screening applications.

This study has some limitations. First, selecting 203 cfDNA differential end motifs for model construction resulted in relatively high detection costs. Future research could validate these differential features using tissue samples from ESCC and precancerous lesions or refine the model by focusing on motifs that show significant increases or decreases in ESCC and precancerous patients. Additionally, the control group consisted of healthy individuals undergoing screening at the hospital, and the sample size for this group was relatively small. To strengthen these findings, future studies should incorporate large-scale, prospective, multicentre data from regions with a high incidence of esophageal cancer for external validation.

## Conclusions

In summary, this study innovatively developed multi-omics predictive models based on cfDNA end-motif detection, tumour-associated protein biomarker detection, and a combined model integrating both approaches. These models demonstrated reliable diagnostic performance for screening ESCC and its precancerous lesions, paving the way for non-invasive liquid biopsy technology using cfDNA for esophageal cancer screening. The next step involves a large-scale, prospective, multicentre, independent validation to further assess the potential of this technology for early diagnosis, treatment, and patient triage prior to endoscopic examinations. The development of this technology also opens the possibility of translation into at-home testing kits, which could enhance its practical value for routine health monitoring and the early identification of esophageal cancer. Prospective independent validation studies are currently underway.

## Supplementary Information

Below is the link to the electronic supplementary material.

Supplementary Material 1

Supplementary Material 2

## Data Availability

The datasets generated and/or analysed during the current study are not publicly available due we are conducting follow-up multicentre research work but are available from the corresponding author on reasonable request.

## Abbreviations

AUC: Area under the curve
cfDNA: Cell-free DNA
CI: Confidence intervals
ESPL: Esophageal squamous precancerous lesion
ESCC: Esophageal squamous cell carcinoma
HGIN: High-grade intraepithelial neoplasia
LGIN: low-grade intraepithelial neoplasia
PCA: Principal component analysis
PGR: PG I/PG II ratio
RF-RFE: Random forest recursive feature elimination
ROC: Receiver operating characteristic
