# Supplementary material for: Development and validation of predictive models combining cell-Free DNA motifs and protein biomarkers for early detection of esophageal squamous cell carcinoma and precancerous lesion
Source: Biomark Res. 2025 Oct 14;13:126. doi: 10.1186/s40364-025-00840-9 (PMC12522225; doi:10.1186/s40364-025-00840-9)
Supplement: Supplementary file 1 — Supplementary Material 1 [file 40364_2025_840_MOESM1_ESM.docx]

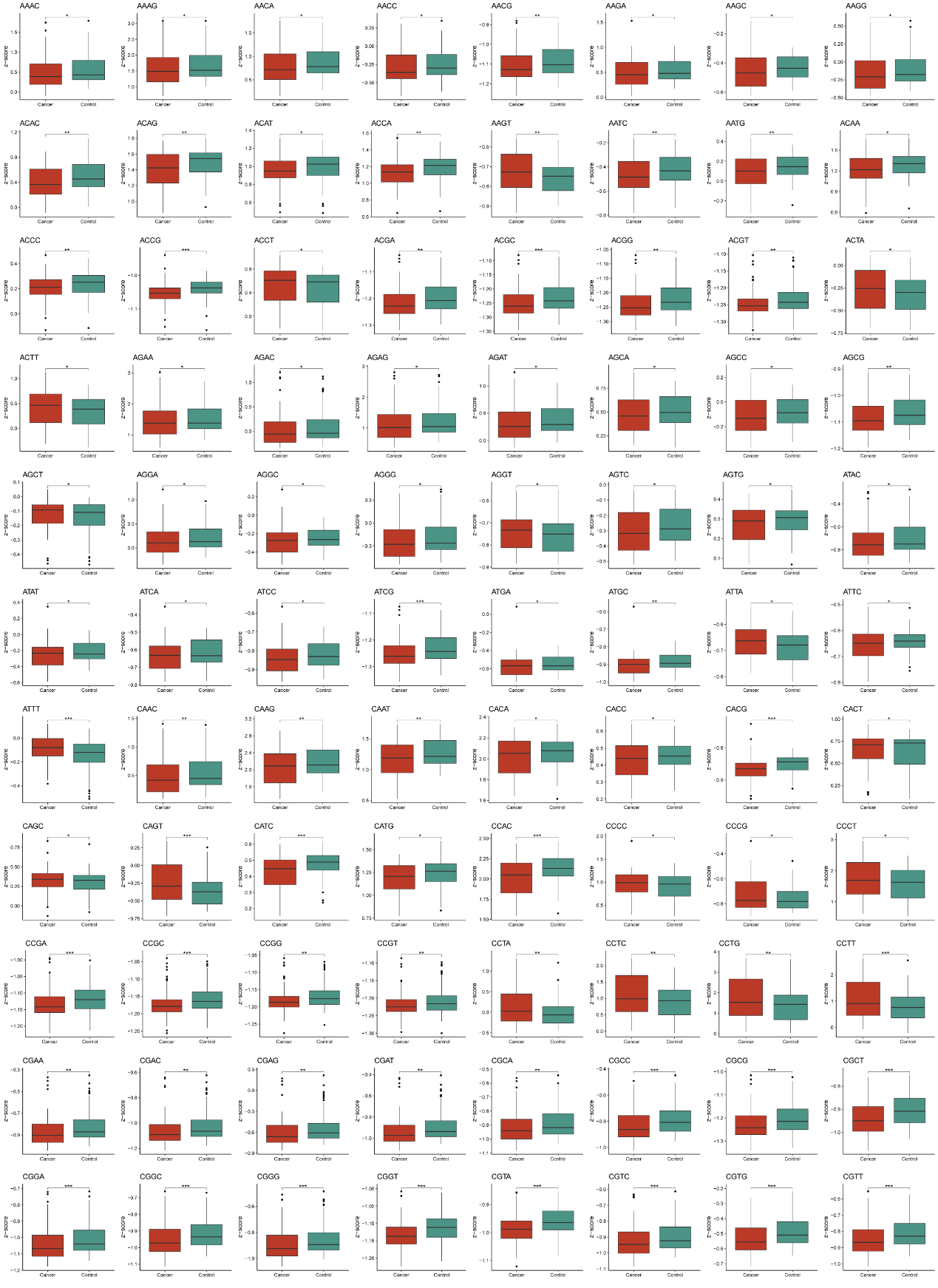


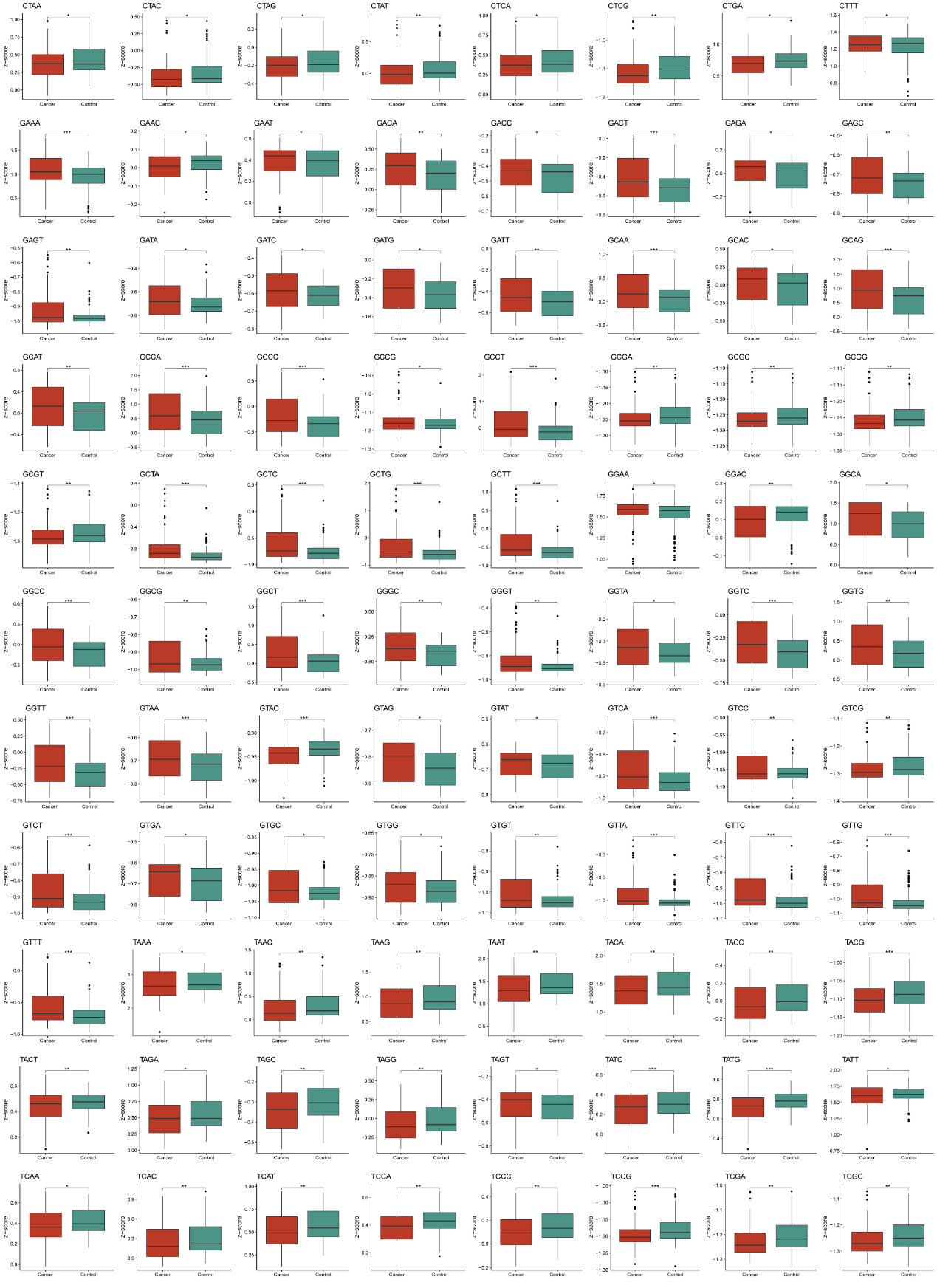


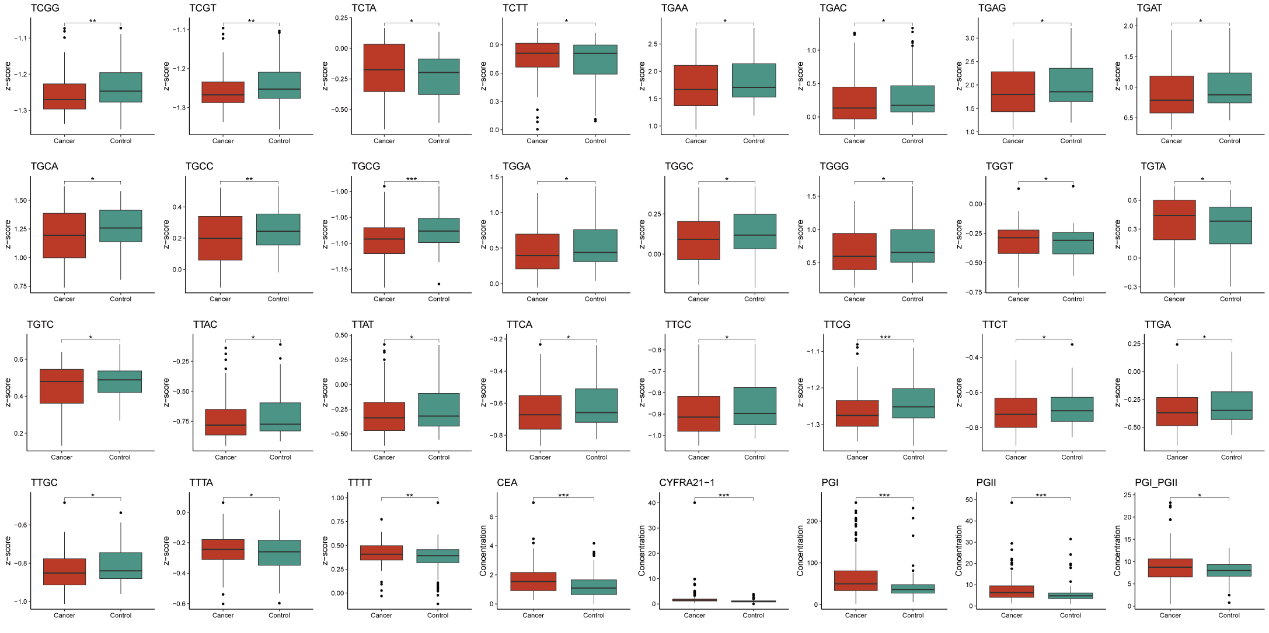


**Figure S1.** The 203 differential terminal motifs and five differential proteins identified in the training set

***p<0^.^001; **p<0^.^01; *p<0^.^05


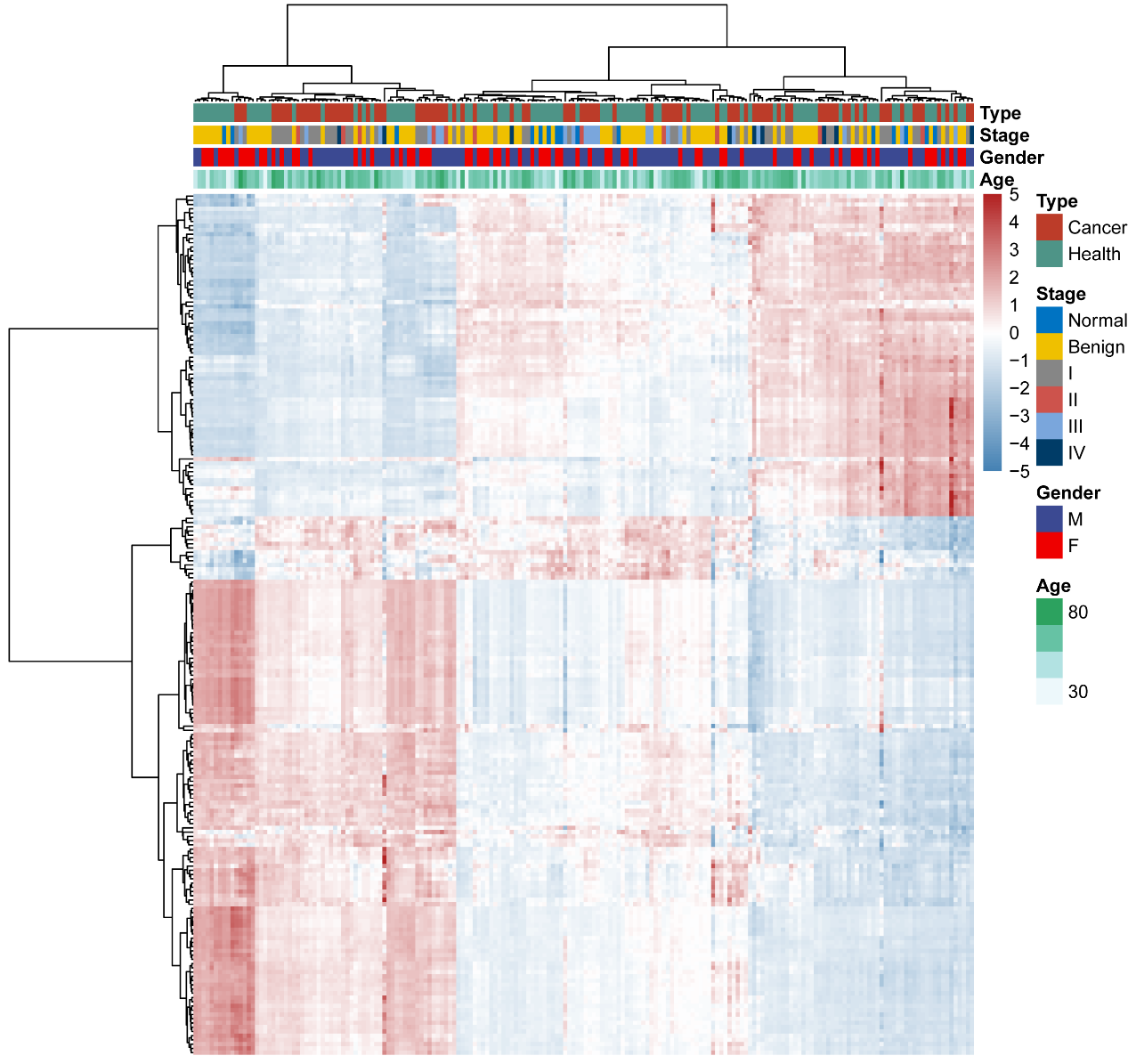


**Figure S2.** Hierarchical clustering analysis of 203 differential terminal motifs between the ESCC, EPSL, and control groups


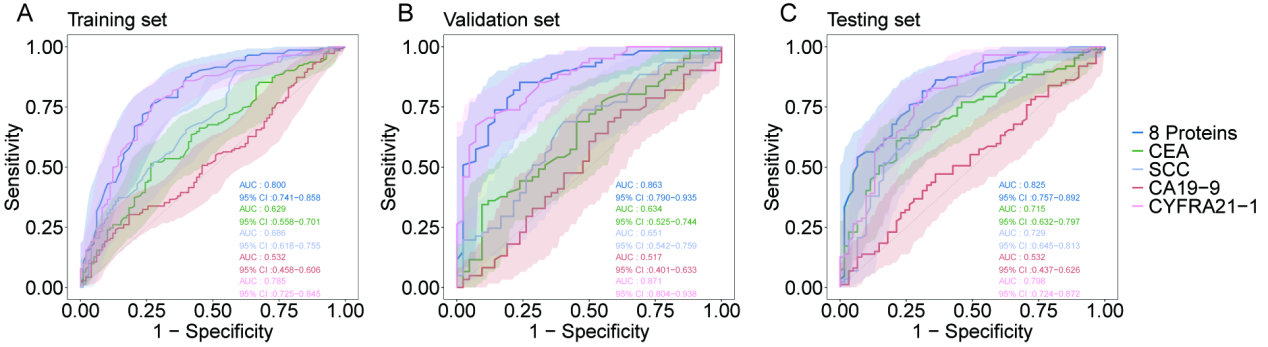


**Figure S3.** Comparison of ROC curves and evaluation metrics for the eight–protein model and CEA, SCC, CA19–9, and Cyfra21–1 in the training (a), validation (b) and testing (c) sets.

**Table S1.** **Quality control results of cfDNA sequencing data from 491 samples**

| **Group** | **Number of cases** | **Raw base count** | **Number of filtered reads** | **Filtered base count** | **Q30** | **GC content** | **Median fragment size** | **Average sequencing depth** | **Number of reads mapped to the genome** | **Mapping rate** | **Number of reads after PCR duplicate removal** | **Genomic coverage** |
| --- | --- | --- | --- | --- | --- | --- | --- | --- | --- | --- | --- | --- |
| ESCC | 199 | 12367132866 | 122690680 | 12004907152 | 0^.^94 | 0^.^42 | 167 | 2^.^81 | 121648421 | 0^.^99 | 109259226 | 0^.^88 |
| ESPL | 91 | 12700695741 | 126462960 | 12377671375 | 0^.^95 | 0^.^42 | 167 | 2^.^98 | 125755203 | 0^.^99 | 114894301 | 0^.^88 |
| Control | 201 | 12575868624 | 126176725 | 12293318085 | 0^.^94 | 0^.^42 | 167 | 2^.^85 | 123935111 | 0^.^98 | 110209425 | 0^.^89 |

**Table S2. Sensitivity and Specificity of the Motif Model in the training, validation, and testing sets**

| **Group** | | **Training Set (n=240)** | | | | **Validation Set (n=103)** | | | | **Testing Set (n=148)** | | | |
| --- | --- | --- | --- | --- | --- | --- | --- | --- | --- | --- | --- | --- | --- |
|  |  | **test cases** | **positive cases** | **Sensitivity (%)**  **95%CI** | **Specificity (%)**  **95%CI** | **test cases** | **positive cases** | **Sensitivity (%)**  **95%CI** | **Specificity (%)**  **95%CI** | **Test**  **cases** | **Positive cases** | **Sensitivity (%)**  **95%CI** | **Specificity (%)**  **95%CI** |
| ESPL |  | 50 | 50 | 100 |  | 15 | 15 | 100 |  | 26 | 25 | 96.2(88^.^8–100) |  |
|  | LGIN | 23 | 23 | 100 |  | 8 | 8 | 100 |  | 15 | 14 | 93.3(80^.^7–100) |  |
|  | HGIN | 27 | 27 | 100 |  | 7 | 7 | 100 |  | 11 | 11 | 100 |  |
| ESCC |  | 92 | 88 | 95^.^7(91^.^5–99^.^8) |  | 46 | 42 | 91.3(83^.^2–99^.^4) |  | 61 | 53 | 86^.^9(78^.^4–95^.^4) |  |
| I |  | 51 | 48 | 94^.^1(87^.^7–100) |  | 35 | 35 | 91.4(82^.^2–100) |  | 38 | 33 | 86^.^8(76^.^1–97^.^6) |  |
|  | T1a | 43 | 41 | 95^.^3(89^.^1–100) |  | 27 | 25 | 92^.^6(82^.^7–100) |  | 30 | 26 | 86^.^7(74^.^5–98^.^8) |  |
|  | T1b | 8 | 7 | 87^.^5(64^.^6–100) |  | 8 | 7 | 87^.^5(64^.^6–100) |  | 8 | 7 | 87^.^5(64^.^6–100) |  |
| HGIN+T1aN0 | | 70 | 68 | 97^.^1(93^.^2–100) |  | 34 | 32 | 94^.^1(86^.^2–100) |  | 41 | 37 | 90^.^2(81^.^2–99^.^3) |  |
| II |  | 11 | 11 | 100 |  | 3 | 3 | 100 |  | 9 | 8 | 88^.^9(68^.^4–100) |  |
| III |  | 21 | 20 | 95^.^2(86^.^1–100) |  | 6 | 5 | 83^.^3(53^.^5–100) |  | 8 | 8 | 100 |  |
| IV |  | 9 | 9 | 100 |  | 2 | 2 | 100 |  | 6 | 4 | 66^.^7(28^.^9–100) |  |
| Control |  | 98 | 44 |  | 55^.^1(45^.^3–64^.^9) | 42 | 14 |  | 66^.^7(52^.^4–80^.^9) | 61 | 27 |  | 55^.^7(4^.^3^.^3–68^.^2) |
|  | Health | 15 | 9 |  | 40^.^0(15^.^2–64^.^8) | 7 | 3 |  | 57^.^1(20^.^5–93^.^8) | 7 | 3 |  | 57^.^1(20^.^5–93^.^8) |
|  | Benign | 83 | 35 |  | 57^.^8(47^.^2–68^.^5) | 35 | 11 |  | 68^.^6(53^.^2–84^.^0) | 54 | 24 |  | 55^.^6(42^.^3–68^.^8) |

Cutoff value: 0^.^39; Control group: healthy controls and benign oesophageal diseases; Precancerous oesophageal lesions: oesophageal mucosal LGIN and HGIN; Control group: healthy controls and benign oesophageal diseases.

**Table S3. Sensitivity and specificity of the eight-protein model in the training, validation, and testing sets**

| **Group** | | **Training Set (n=240)** | | | | **Validation Set (n=103)** | | | | **Testing Set (n=148)** | | | |
| --- | --- | --- | --- | --- | --- | --- | --- | --- | --- | --- | --- | --- | --- |
|  |  | **test cases** | **positive cases** | **Sensitivity (%)**  **95%CI** | **Specificity (%)**  **95%CI** | **test cases** | **positive cases** | **Sensitivity (%)**  **95%CI** | **Specificity (%)**  **95%CI** | **Test**  **cases** | **Positive cases** | **Sensitivity (%)**  **95%CI** | **Specificity (%)**  **95%CI** |
| ESPL |  | 50 | 36 | 72.0(58^.^6–84^.^4) |  | 15 | 11 | 73^.^3(51^.^0–95^.^7) |  | 26 | 17 | 65^.^4(47^.^1–83^.^7) |  |
|  | LGIN | 23 | 15 | 65^.^2(45^.^8–84^.^7) |  | 8 | 5 | 62^.^5(29^.^0–96^.^0) |  | 15 | 9 | 60^.^0(35^.^2–84^.^8) |  |
|  | HGIN | 27 | 21 | 77^.^8(62^.^1–93^.^5) |  | 7 | 6 | 85^.^7 (59^.^8–100) |  | 11 | 8 | 72^.^7(46^.^4–99^.^0) |  |
| ESCC |  | 92 | 76 | 82^.^6(74^.^9–90^.^4) |  | 46 | 41 | 89^.^1(80^.^1–98^.^1) |  | 61 | 54 | 88^.^5(80^.^5–96^.^5) |  |
| I |  | 51 | 44 | 86^.^3(76^.^8–95^.^6) |  | 35 | 31 | 88^.^6(78^.^0–99^.^1) |  | 38 | 32 | 84^.^2(72^.^6–95^.^8) |  |
|  | T1a | 43 | 37 | 86^.^0(75^.^7–96^.^4) |  | 27 | 23 | 85^.^2(71^.^8–98^.^6) |  | 30 | 25 | 83^.^3(70^.^0–96^.^7) |  |
|  | T1b | 8 | 7 | 87^.^5 (64^.^6–100) |  | 8 | 8 | 100 |  | 8 | 7 | 87^.^5 (64^.^6–100) |  |
| HGIN+T1aN0 | | 70 | 58 | 82^.^9(74^.^0–91^.^7) |  | 34 | 29 | 85^.^3(73^.^4–97^.^2) |  | 41 | 33 | 80^.^5(68^.^4–92^.^6) |  |
| II |  | 11 | 10 | 90^.^9 (73^.^9–100) |  | 3 | 2 | 66^.^7 (13^.^3–100) |  | 9 | 9 | 100 |  |
| III |  | 21 | 13 | 61^.^9(41^.^1–82^.^7) |  | 6 | 6 |  |  | 8 | 7 | 87^.^5 (64^.^6–100) |  |
| IV |  | 9 | 9 | 100 |  | 2 | 2 |  |  | 6 | 6 | 100 |  |
| Control |  | 98 | 35 |  | 64^.^3(54^.^8–73^.^8) | 42 | 10 |  | 76^.^2(63^.^3–89^.^1) | 61 | 19 |  | 68^.^9(57^.^2–80^.^5) |
|  | Health | 15 | 5 |  | 66^.^7(42^.^8–90^.^5) | 7 | 1 |  | 85^.^7 (59^.^8–100) | 7 | 1 |  | 85^.^7 (59^.^8–100) |
|  | Benign | 83 | 30 |  | 63^.^9(53^.^5–74^.^2) | 35 | 9 |  | 74^.^3(59^.^8–88^.^8) | 54 | 18 |  | 66^.^7(54^.^1–79^.^2) |

Cutoff value: 0^.^55; Control group: healthy controls and benign oesophageal diseases; Precancerous oesophageal lesions: oesophageal mucosal LGIN and HGIN; Control group: healthy controls and benign oesophageal diseases.

**Table S4. Sensitivity and specificity of the motif–protein model in the training, validation, and testing sets**

| **Group** | | **Training Set (n=240)** | | | | **Validation Set (n=103)** | | | | **Testing Set (n=148)** | | | |
| --- | --- | --- | --- | --- | --- | --- | --- | --- | --- | --- | --- | --- | --- |
|  |  | **test cases** | **positive cases** | **Sensitivity (%)**  **95%CI** | **Specificity (%)**  **95%CI** | **test cases** | **positive cases** | **Sensitivity (%)**  **95%CI** | **Specificity (%)**  **95%CI** | **Test**  **cases** | **Positive cases** | **Sensitivity (%)**  **95%CI** | **Specificity (%)**  **95%CI** |
| ESPL |  | 50 | 48 | 96^.^0(90^.^6–100) |  | 15 | 14 | 93^.^3(80^.^7–100) |  | 26 | 21 | 80^.^8(65^.^5–95^.^9) |  |
|  | LGIN | 23 | 23 | 100 |  | 8 | 8 | 100 |  | 15 | 11 | 73^.^3(51^.^0–95^.^7) |  |
|  | HGIN | 27 | 25 | 92^.^6(82^.^7–100) |  | 7 | 6 | 85^.^7(59^.^8–100) |  | 11 | 10 | 90^.^9(73^.^9–100) |  |
| ESCC |  | 92 | 84 | 91^.^3(85^.^5–97^.^1) |  | 46 | 45 | 97^.^8(93^.^6–100) |  | 61 | 56 | 91^.^8(84^.^9–98^.^7) |  |
| I |  | 51 | 48 | 94^.^1(87^.^7–100) |  | 35 | 34 | 97^.^1(91^.^6–100) |  | 38 | 33 | 86^.^8(76^.^1–97^.^6) |  |
|  | T1a | 43 | 41 | 95^.^3(89^.^1–100) |  | 27 | 26 | 96^.^3(89^.^2–100) |  | 30 | 26 | 86^.^7(74^.^5–98^.^8) |  |
|  | T1b | 8 | 7 | 87^.^5(64^.^6–100) |  | 8 | 8 | 100 |  | 8 | 7 | 87^.^5(64^.^6–100) |  |
| HGIN+T1aN0 | | 70 | 66 | 94^.^3(88^.^8–99^.^7) |  | 34 | 32 | 94^.^1(86^.^2–100) |  | 41 | 36 | 87^.^8(77^.^8–97^.^8) |  |
| II |  | 11 | 11 | 100 |  | 3 | 3 | 100 |  | 9 | 9 | 100 |  |
| III |  | 21 | 16 | 76^.^2(58^.^0–94^.^4) |  | 6 | 6 | 100 |  | 8 | 8 | 100 |  |
| IV |  | 9 | 9 | 100 |  | 2 | 2 | 100 |  | 6 | 6 | 100 |  |
| Control |  | 98 | 29 |  | 70^.^4(61^.^4–79^.^4) | 42 | 8 |  | 81^.^0(69^.^1–92^.^8) | 61 | 15 |  | 75^.^4(64^.^4–86^.^2) |
|  | Health | 15 | 1 |  | 93^.^3(80^.^7–100) | 7 | 1 |  | 85^.^7(59^.^8–100) | 7 | 2 |  | 71^.^4(38^.^0–100) |
|  | Benign | 83 | 28 |  | 66^.^3(56^.^1–76^.^4) | 35 | 7 |  | 80^.^0(66^.^7–93^.^3) | 54 | 13 |  | 75^.^9(64^.^5–87^.^3) |

Cutoff value: 0^.^50; Control group: healthy controls and benign oesophageal diseases; Oesophageal precancerous lesions: oesophageal mucosal LGIN and HGIN; Control group: healthy controls and benign oesophageal diseases.

**Table S5. Comparison of the detection performance of the motif–protein model, motif model, eight–protein model, and protein markers in the validation set**

| **Validation Set** | **Sensitivity (%)**  **95%CI** | **Specificity (%)**  **95%CI** | **AUC** | **Threshold** | **p–value^a^** | **p–value^b^** |
| --- | --- | --- | --- | --- | --- | --- |
| Motif–Protein Model | 96^.^7 (92^.^3–100) | 81^.^0(69^.^1–92^.^8) | 0^.^91 | 0^.^50 | 1^.^000 | 0^.^388 |
| Motif Model | 93^.^4(87^.^2–99^.^7) | 66^.^7(52^.^4–80^.^9) | 0^.^86 | 0^.^39 | 0^.^364 | 0^.^953 |
| Eight–protein Model | 85^.^2(76^.^3–94^.^1) | 76^.^2(63^.^3–89^.^1) | 0^.^86 | 0^.^55 | 0^.^388 | 1^.^000 |
| CEA | 34^.^4(22^.^5–46^.^3) | 90^.^5(81^.^6–99^.^4) | 0^.^63 | 2^.^21 | <0^.^001 | 0^.^001 |
| SCC | 68^.^9(57^.^2–80^.^5) | 59^.^5(44^.^7–74^.^4) | 0^.^65 | 0^.^65 | <0^.^001 | <0^.^001 |
| CA19–9 | 26^.^2(15^.^2–37^.^3) | 61^.^9(47^.^2–76^.^6) | 0^.^52 | 10^.^95 | <0^.^001 | <0^.^001 |
| CYFRA21–1 | 67^.^2(55^.^4–79^.^0) | 92^.^9 (85^.^1–100) | 0^.^87 | 1^.^33 | 0^.^465 | 0^.^781 |
| CA24–2 | 14^.^8 (5^.^9–23^.^7) | 64^.^3(49^.^8–78^.^8) | 0^.^54 | 4^.^56 | <0^.^001 | <0^.^001 |
| PG I | 68^.^9(36^.^6–61^.^7) | 81^.^0(69^.^1–92^.^8) | 0^.^76 | 45^.^44 | 0^.^016 | 0^.^030 |
| PG II | 49^.^2(36^.^6–61^.^7) | 90^.^5(81^.^6–99^.^4) | 0^.^70 | 6^.^95 | 0^.^001 | 0^.^001 |
| PG I/PG II(PGR) | 32^.^8(21^.^0–44^.^6) | 85^.^7(75^.^1–96^.^3) | 0^.^56 | 9^.^76 | <0^.^001 | <0^.^001 |

^a^ indicates comparison with the Motif–Protein model calculated using DeLong’s test.

^b^ indicates comparison with the 8–Proteins model calculated using DeLong’s test.

**Table S6. Comparison of the detection performance of the motif–protein model, motif model, eight–protein model, and protein markers in the training set**

| **Training Set** | **Sensitivity (%)**  **95%CI** | **Specificity (%)**  **95%CI** | **AUC** | **Threshold** | **p–value^a^** | **p–value^b^** |
| --- | --- | --- | --- | --- | --- | --- |
| Motif–Protein Model | 93^.^0(88^.^7–97^.^2) | 70^.^4(61^.^4–79^.^4) | 0^.^90 | 0^.^50 | 1^.^000 | 0^.^004 |
| Motif Model | 97^.^2(94^.^5–99^.^9) | 55^.^1(45^.^3–64^.^9) | 0^.^89 | 0^.^39 | 0^.^744 | 0^.^005 |
| Eight–Protein Model | 78^.^9(72^.^2–85^.^6) | 64^.^3(54^.^8–73^.^8) | 0^.^80 | 0^.^55 | 0^.^004 | 1^.^000 |
| CEA | 23^.^9(16^.^9–31^.^0) | 88^.^8(82^.^5–95^.^0) | 0^.^63 | 2^.^21 | <0^.^001 | <0^.^001 |
| SCC | 65^.^5(57^.^7–73^.^3) | 60^.^2(50^.^5–69^.^9) | 0^.^69 | 0^.^65 | <0^.^001 | 0^.^014 |
| CA19–9 | 33^.^8(26^.^0–41^.^6) | 70^.^4(61^.^4–79^.^4) | 0^.^53 | 10^.^95 | <0^.^001 | <0^.^001 |
| CYFRA21–1 | 64^.^8(56^.^9–72^.^6) | 78^.^6(70^.^4–86^.^7) | 0^.^79 | 1^.^33 | 0^.^001 | 0^.^738 |
| CA24–2 | 19^.^0(12^.^6–25^.^5) | 73^.^5(64^.^7–82^.^2) | 0^.^53 | 4^.^56 | <0^.^001 | <0^.^001 |
| PG I | 55^.^6(47^.^5–63^.^8) | 70^.^4(61^.^4–79^.^4) | 0^.^68 | 45^.^44 | <0^.^001 | 0^.^007 |
| PG II | 44^.^4(36^.^2–52^.^5) | 81^.^6(74^.^0–89^.^3) | 0^.^65 | 6^.^95 | <0^.^001 | 0^.^002 |
| PG I/PG II(PGR) | 36^.^6(28^.^7–44^.^5) | 78^.^6(70^.^4–86^.^7) | 0^.^57 | 9^.^76 | <0^.^001 | <0^.^001 |

^a^ indicates comparison with the motif–protein model calculated using DeLong’s test.

^b^ indicates comparison with the eight–protein model calculated using DeLong’s test.
