# Supplementary figures and images for: Development and validation of predictive models combining cell-Free DNA motifs and protein biomarkers for early detection of esophageal squamous cell carcinoma and precancerous lesion
Source: Biomark Res. 2025 Oct 14;13:126. doi: 10.1186/s40364-025-00840-9 (PMC12522225; doi:10.1186/s40364-025-00840-9)

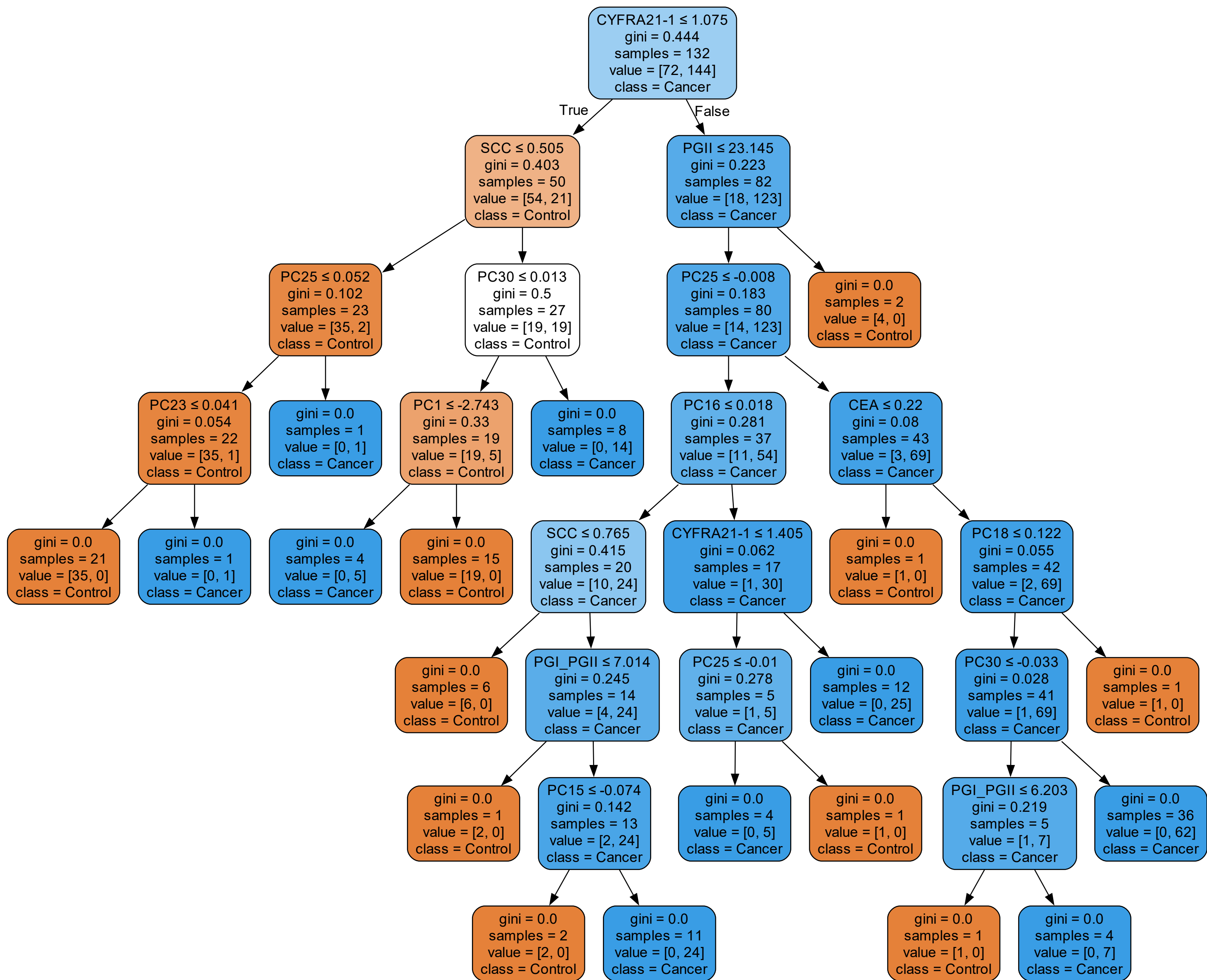

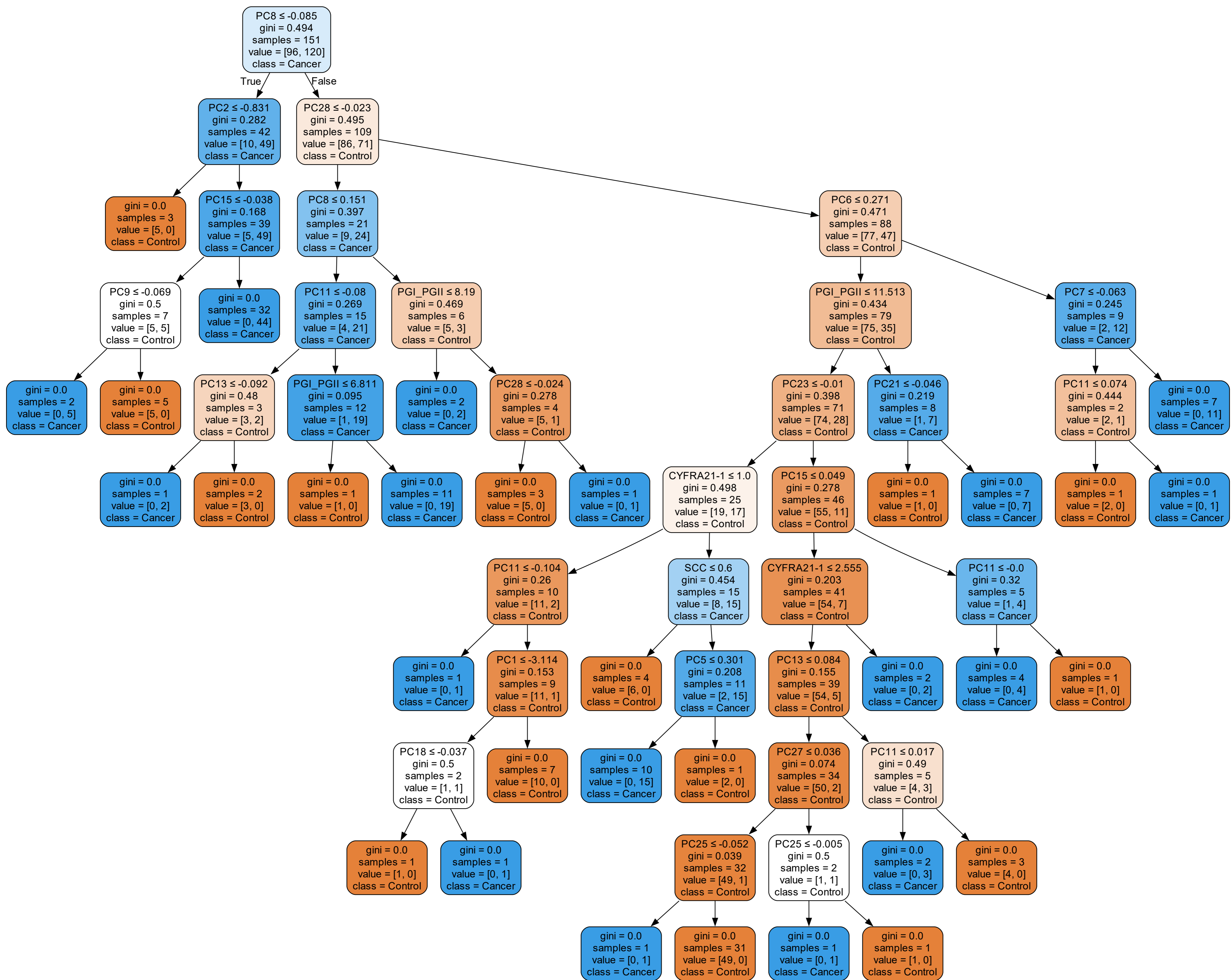

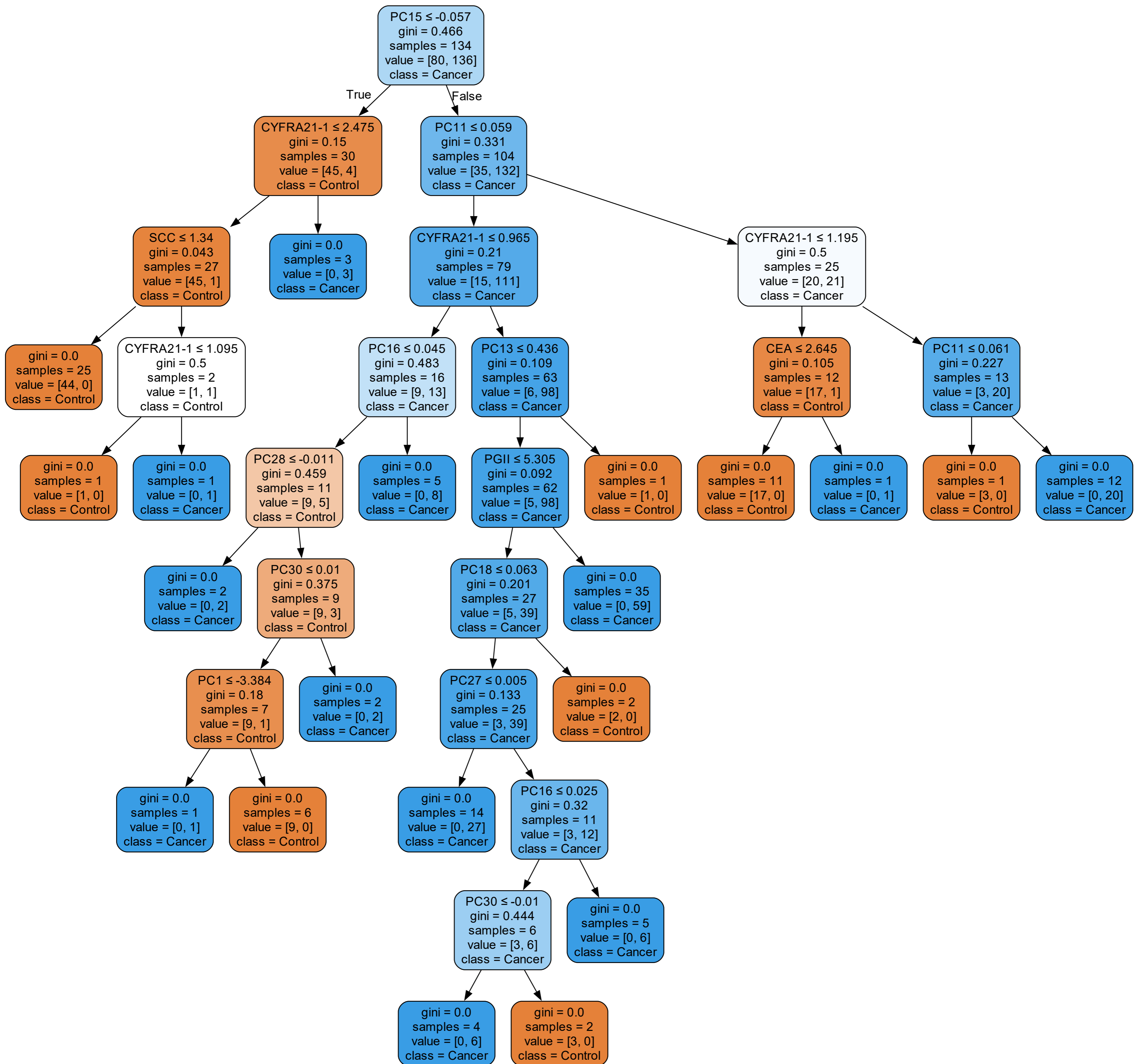

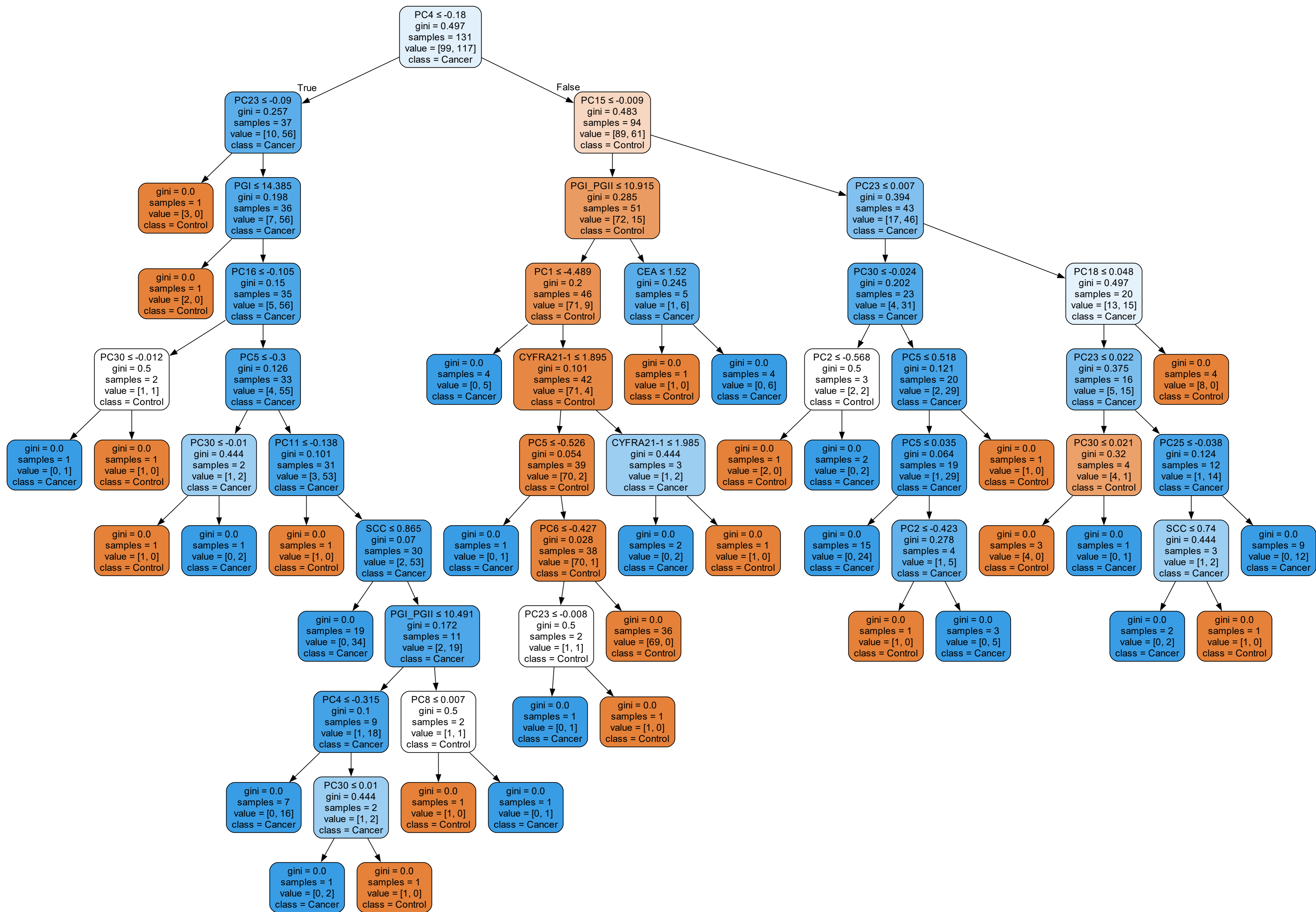

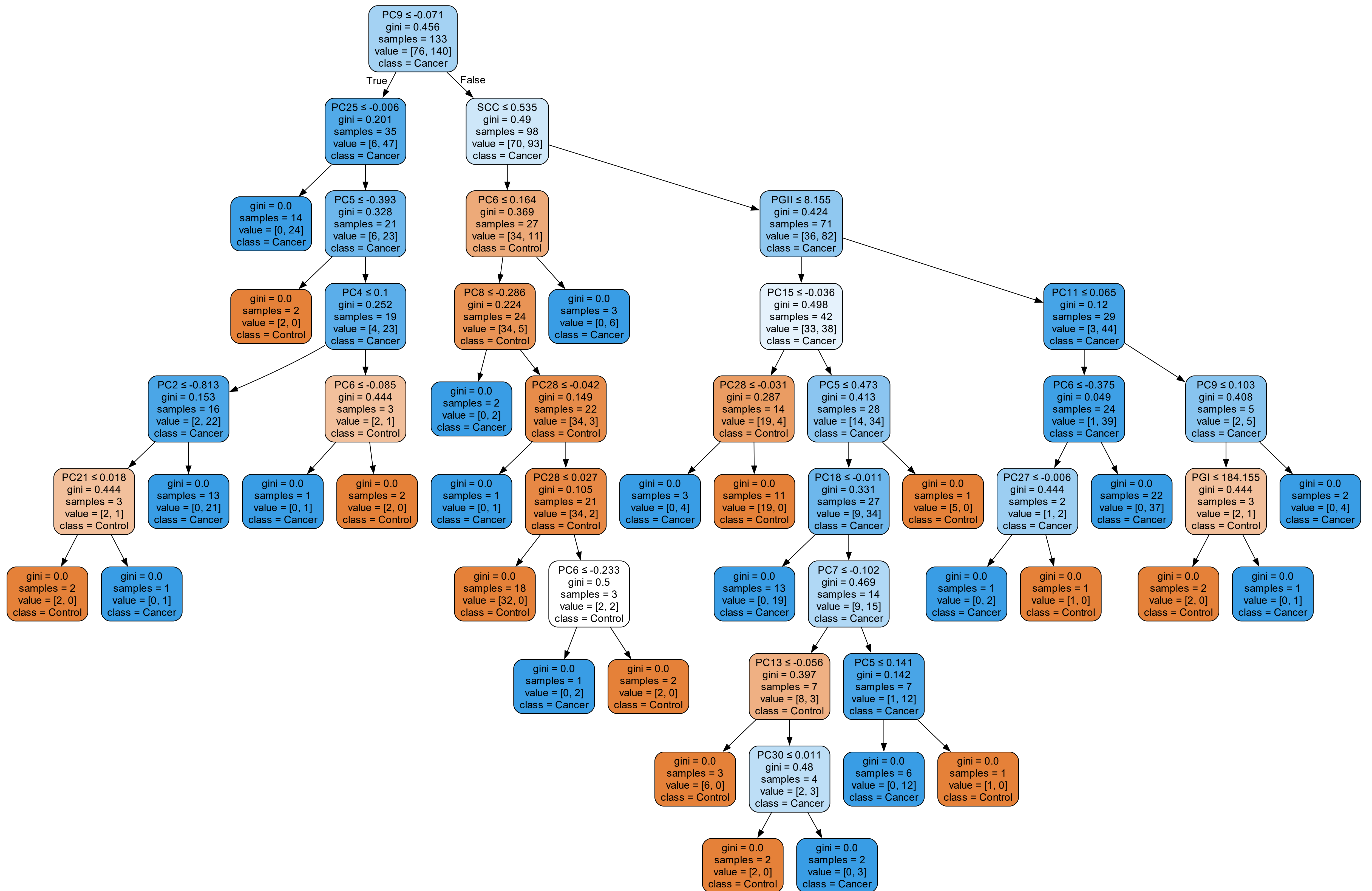

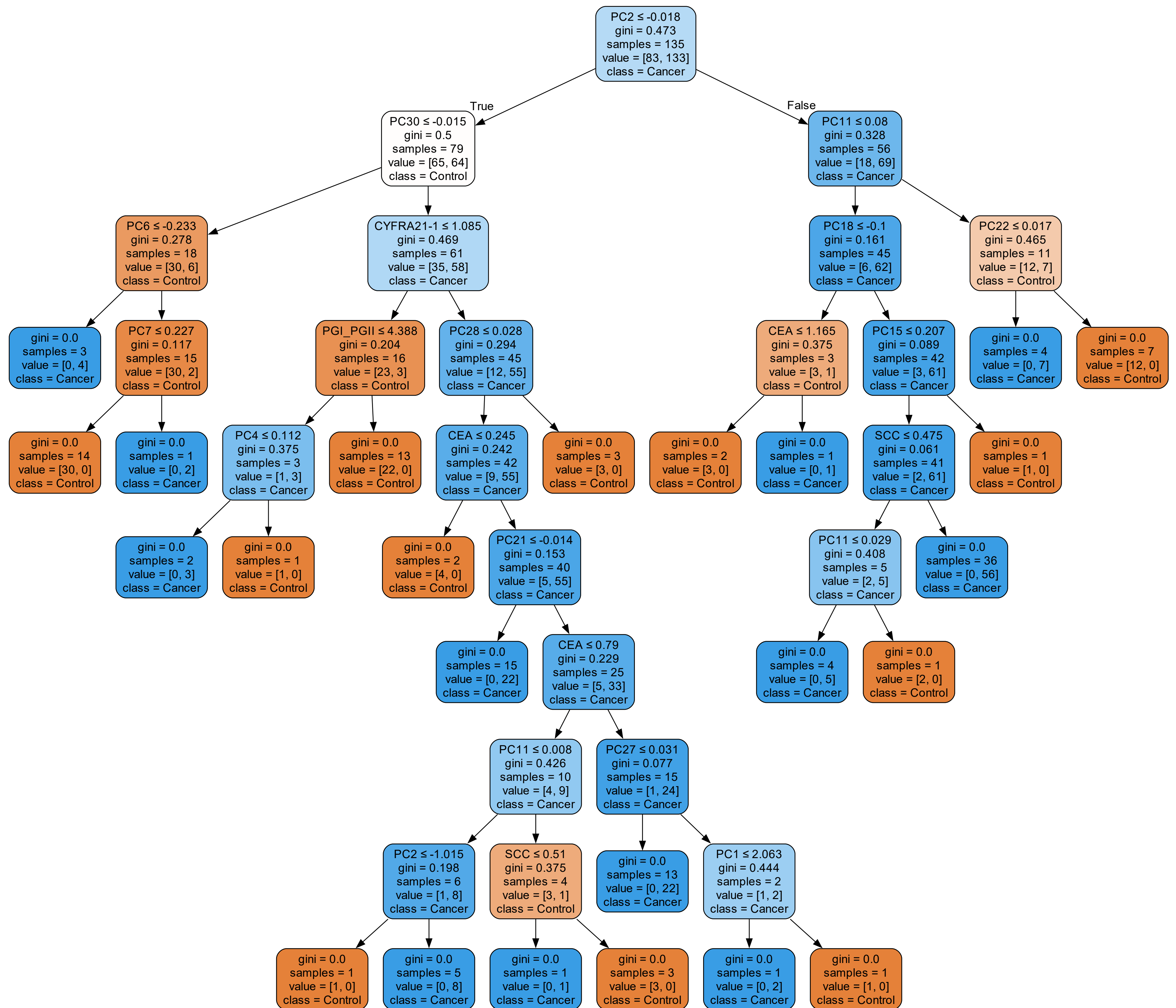

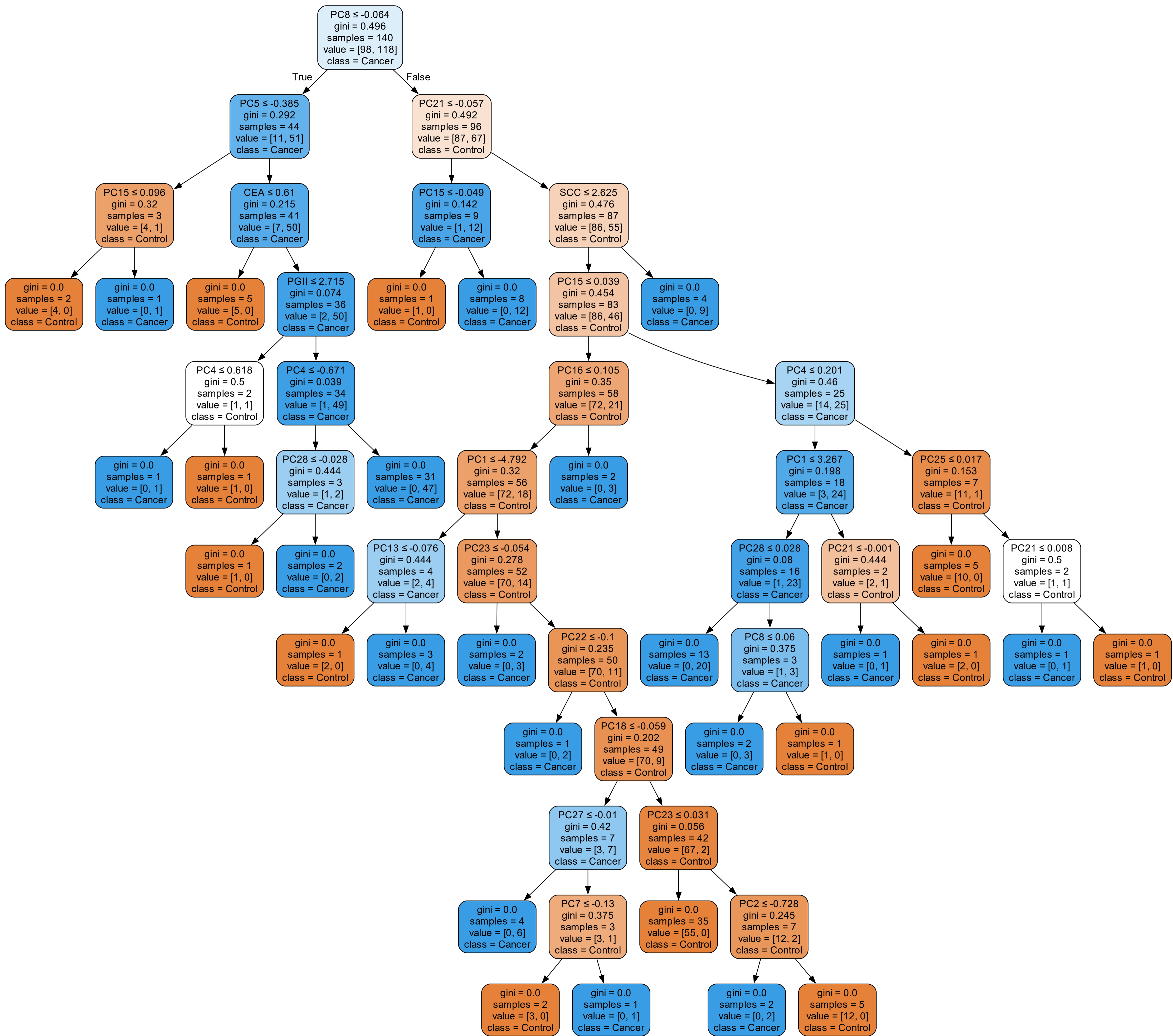

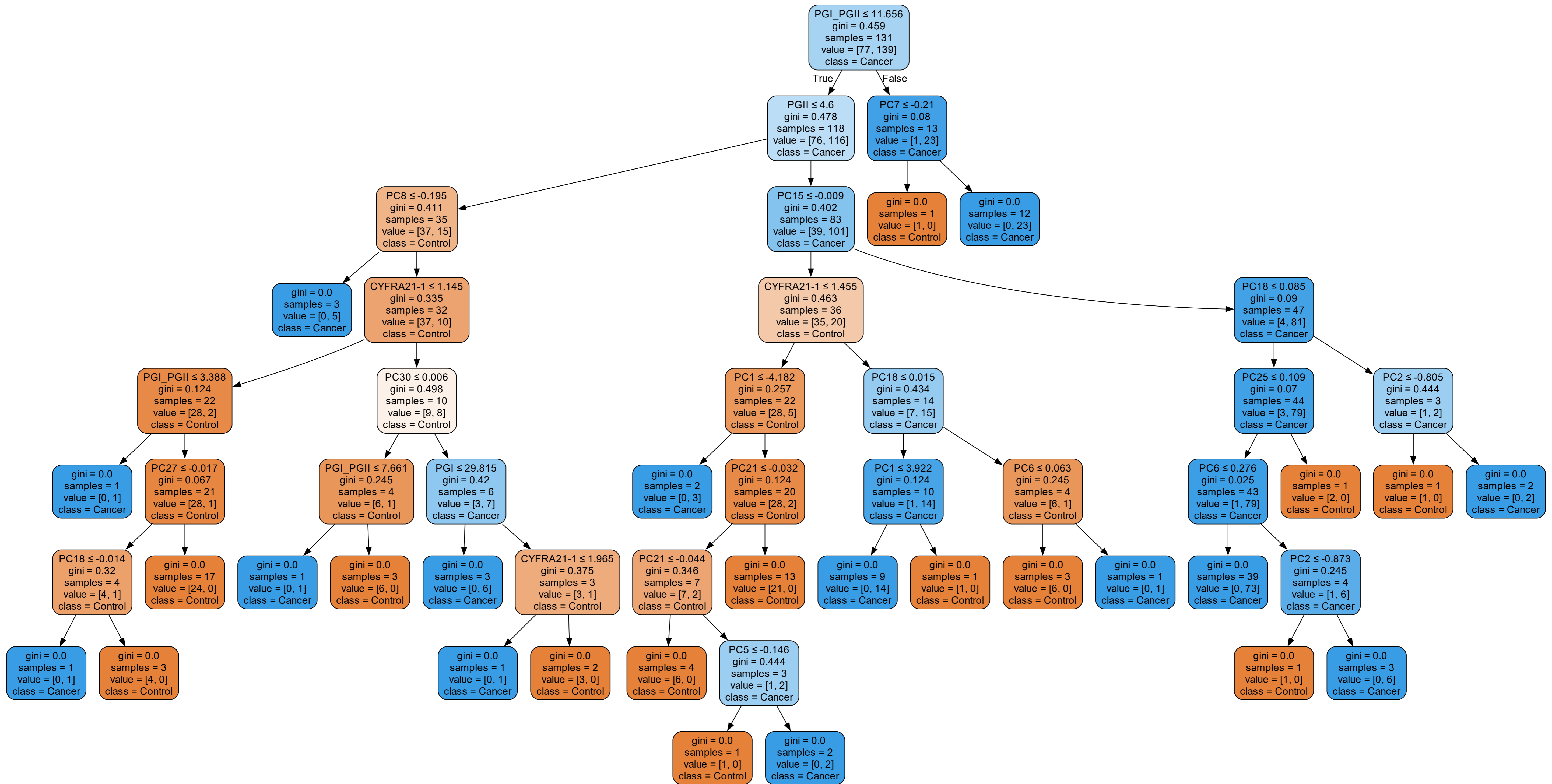

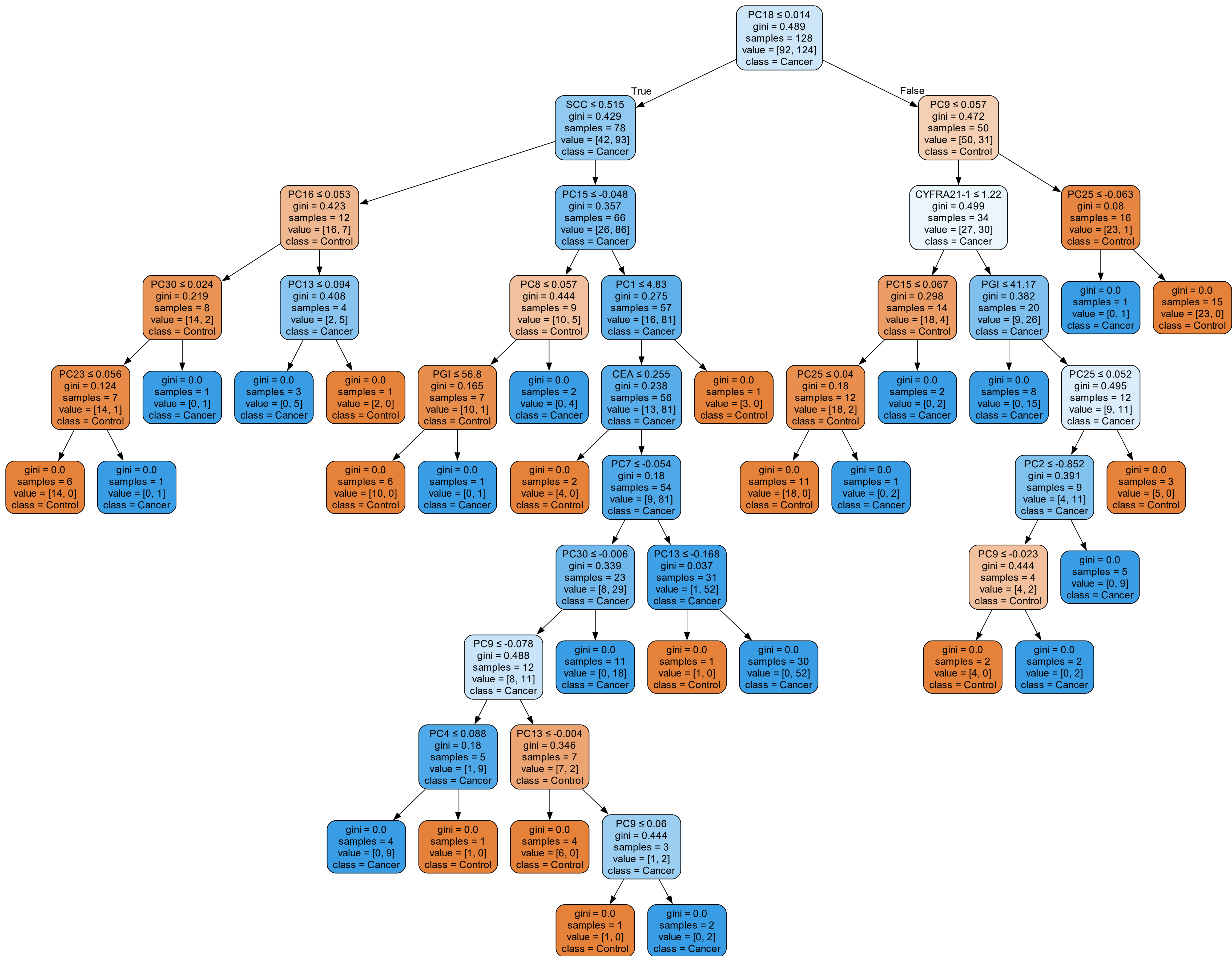

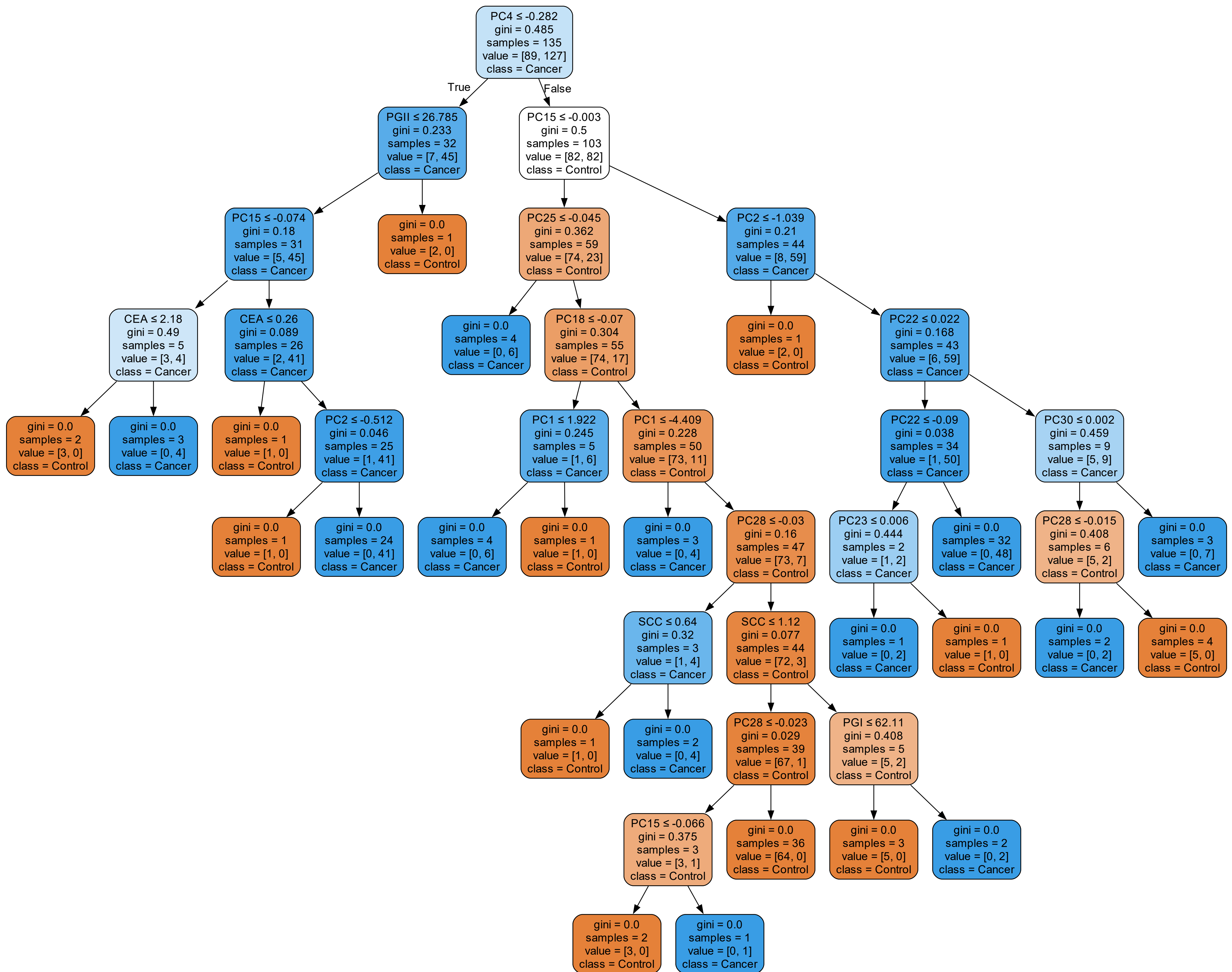

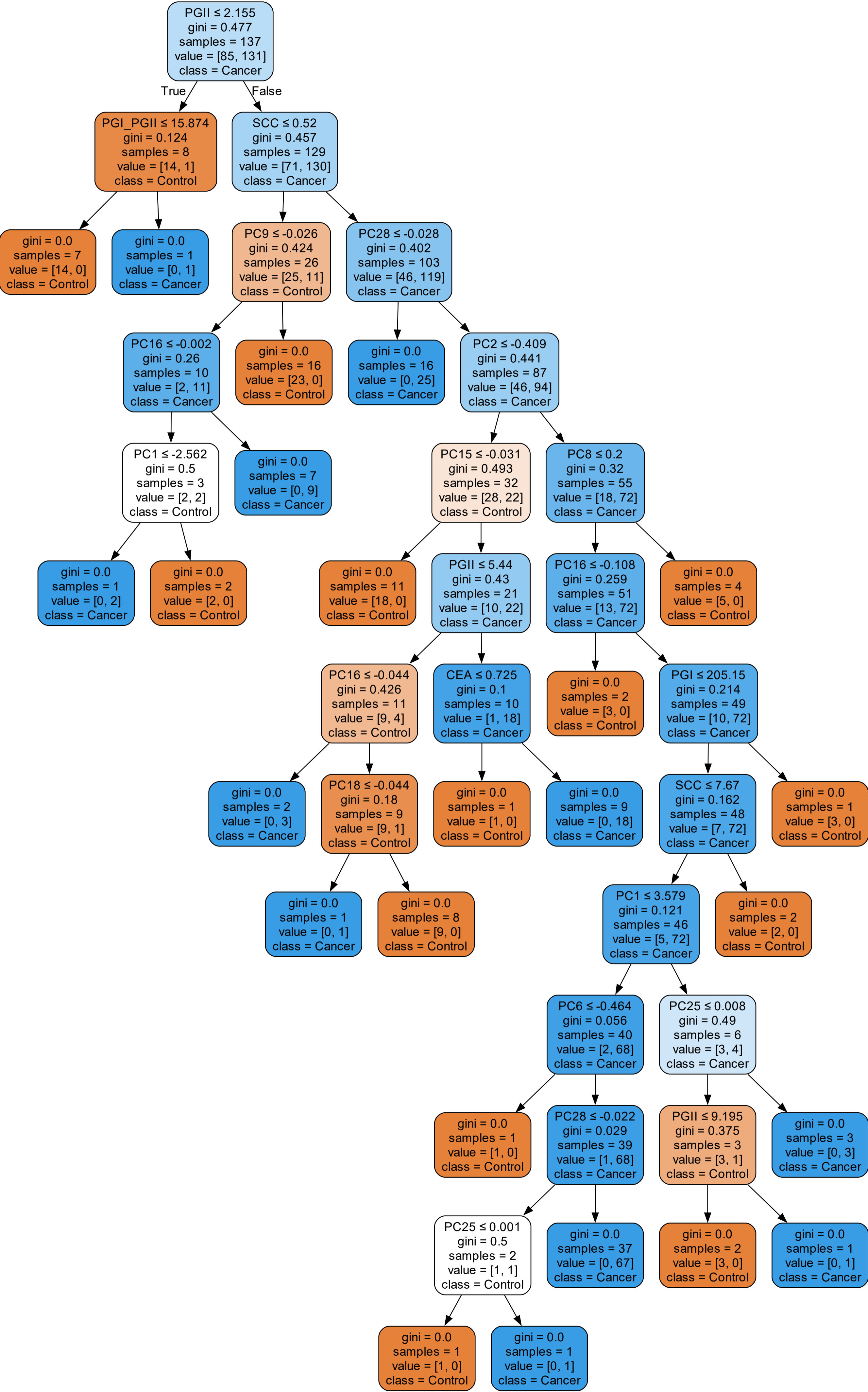

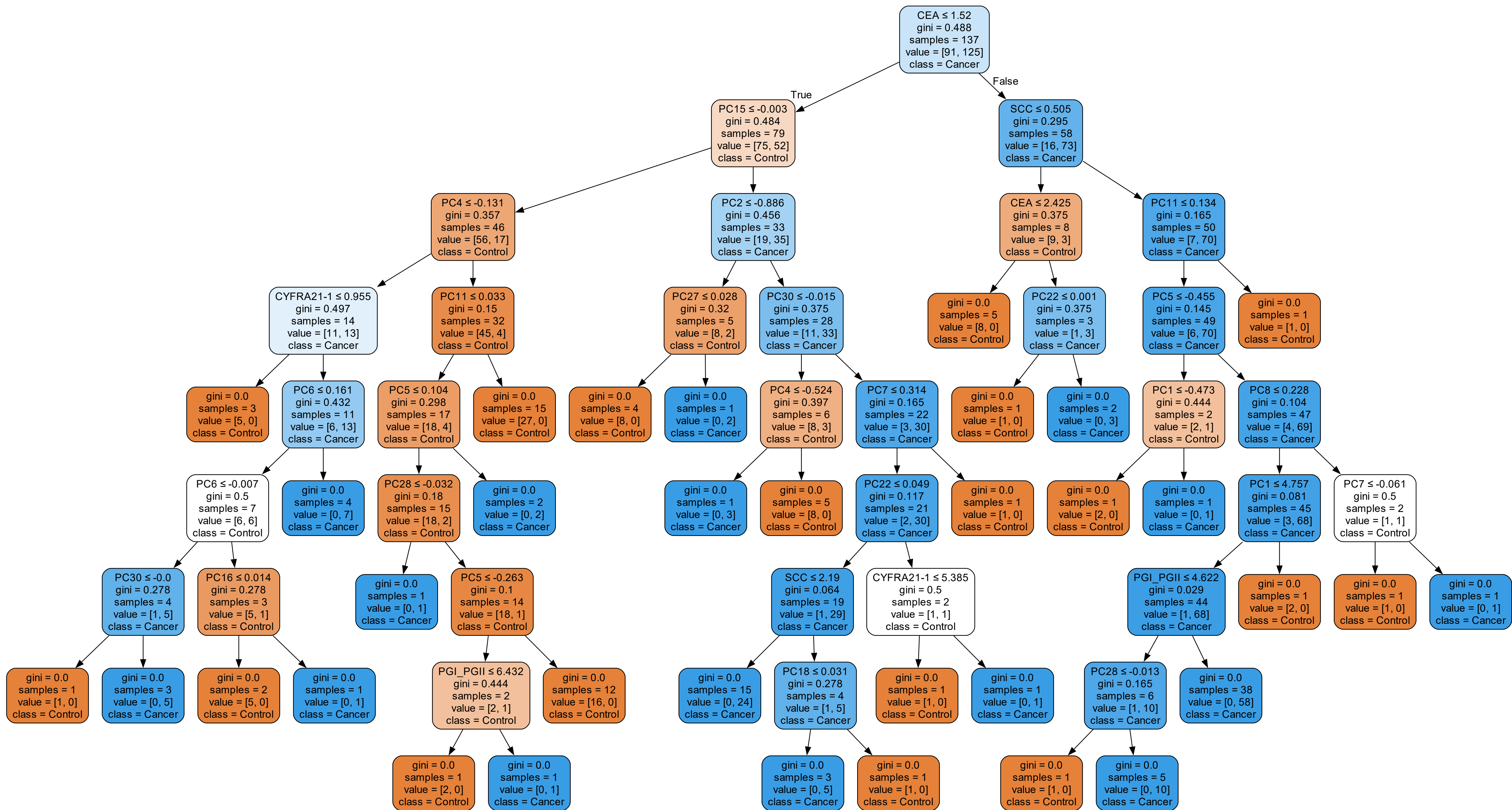

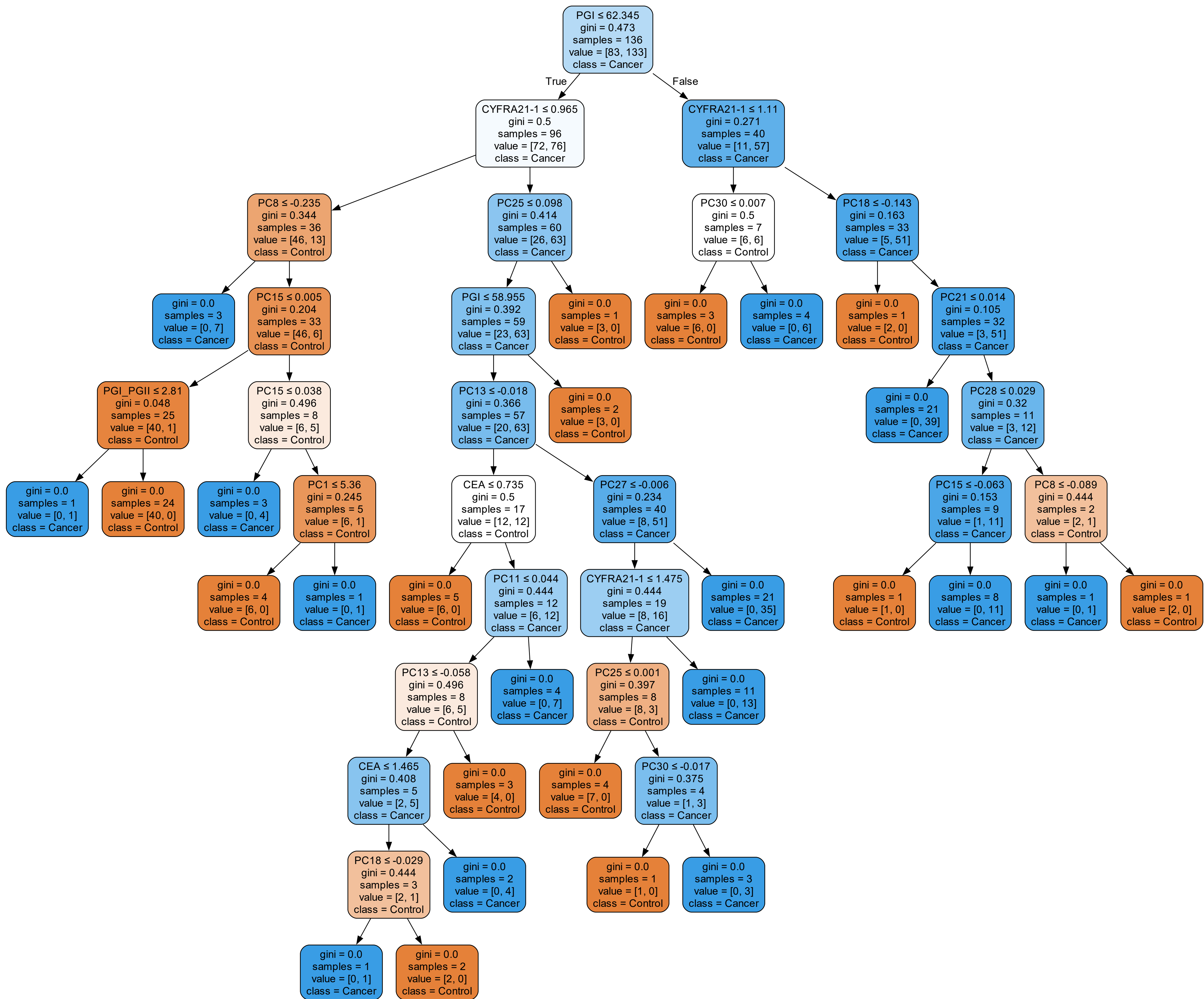

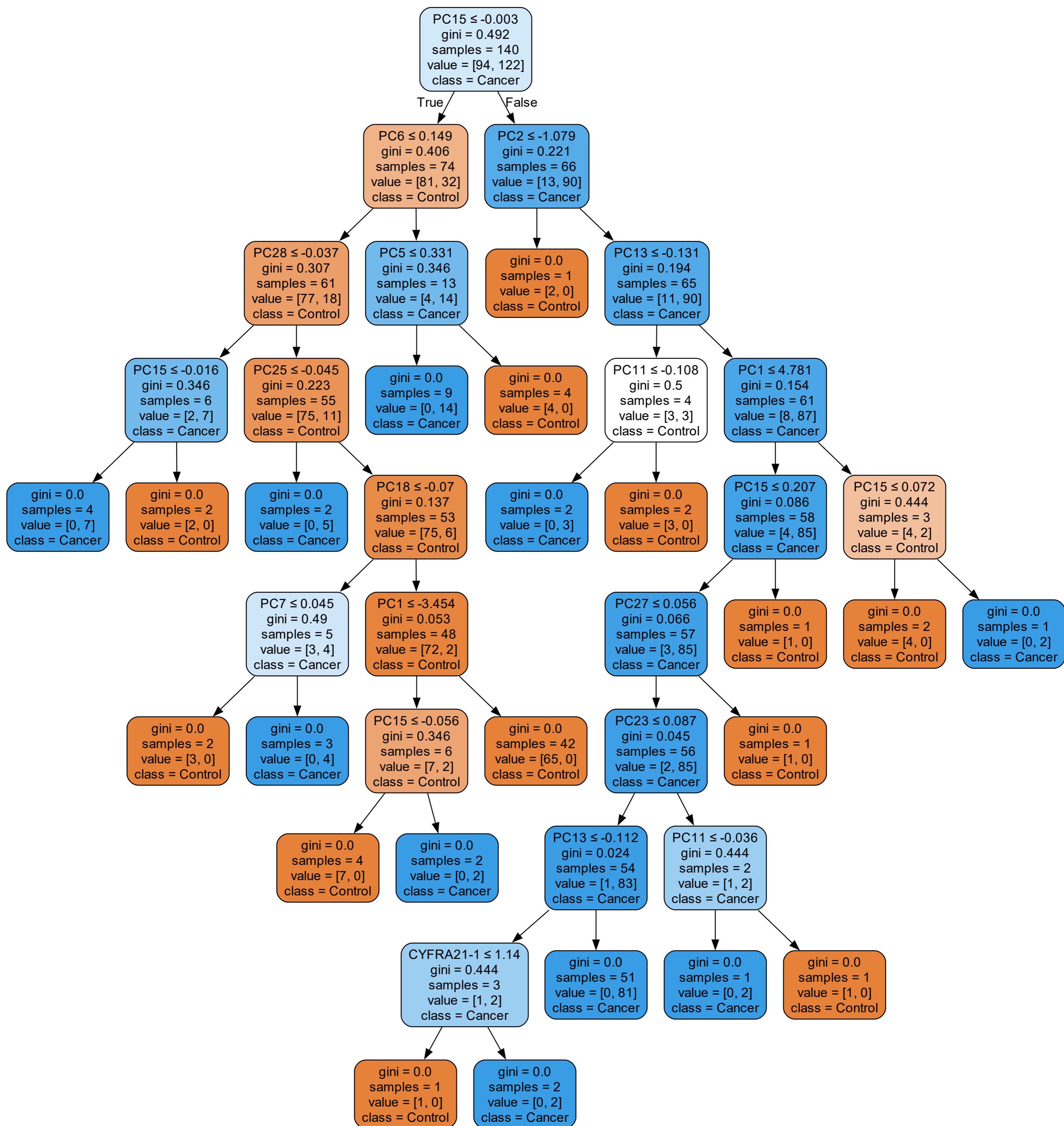

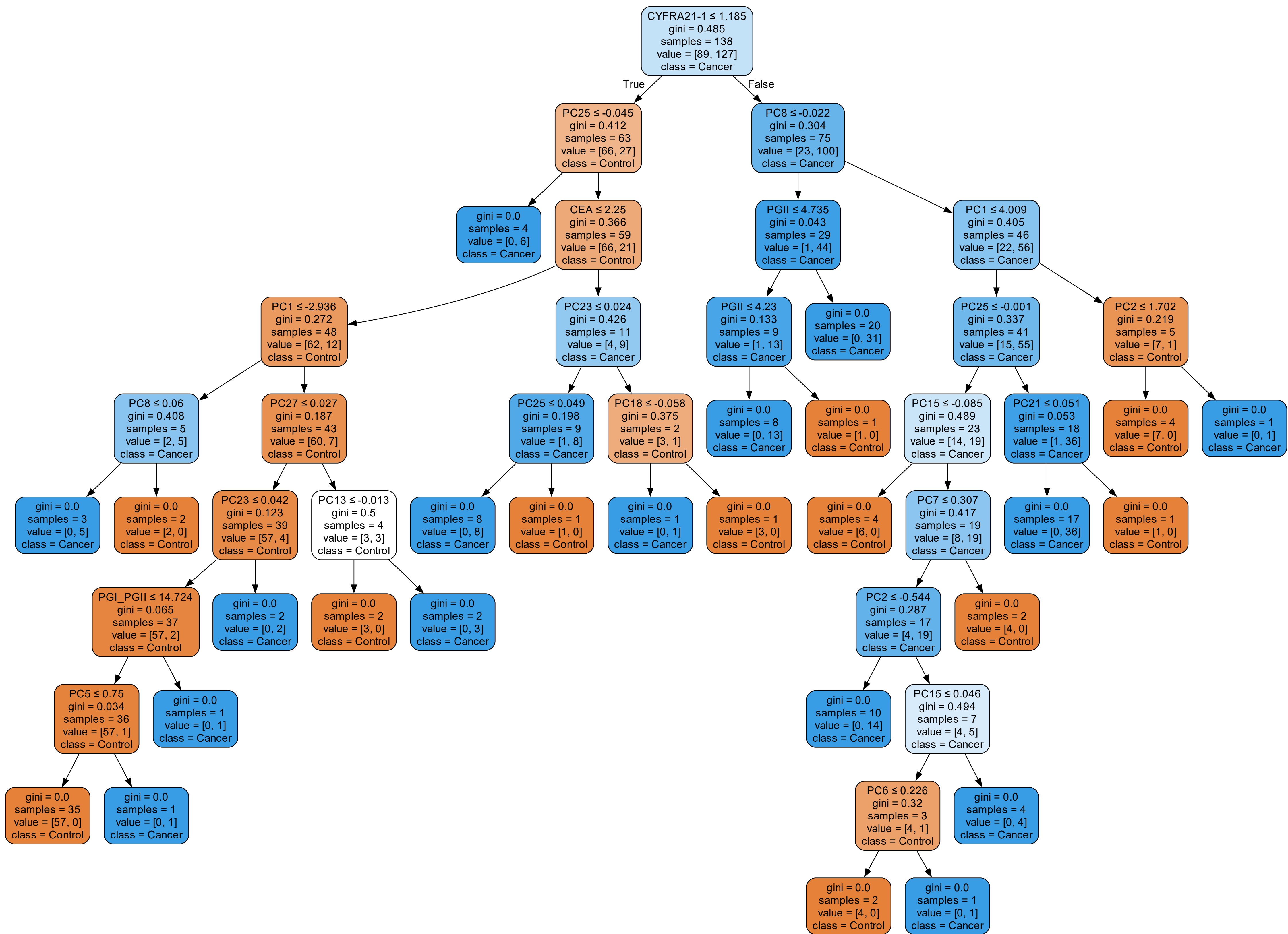

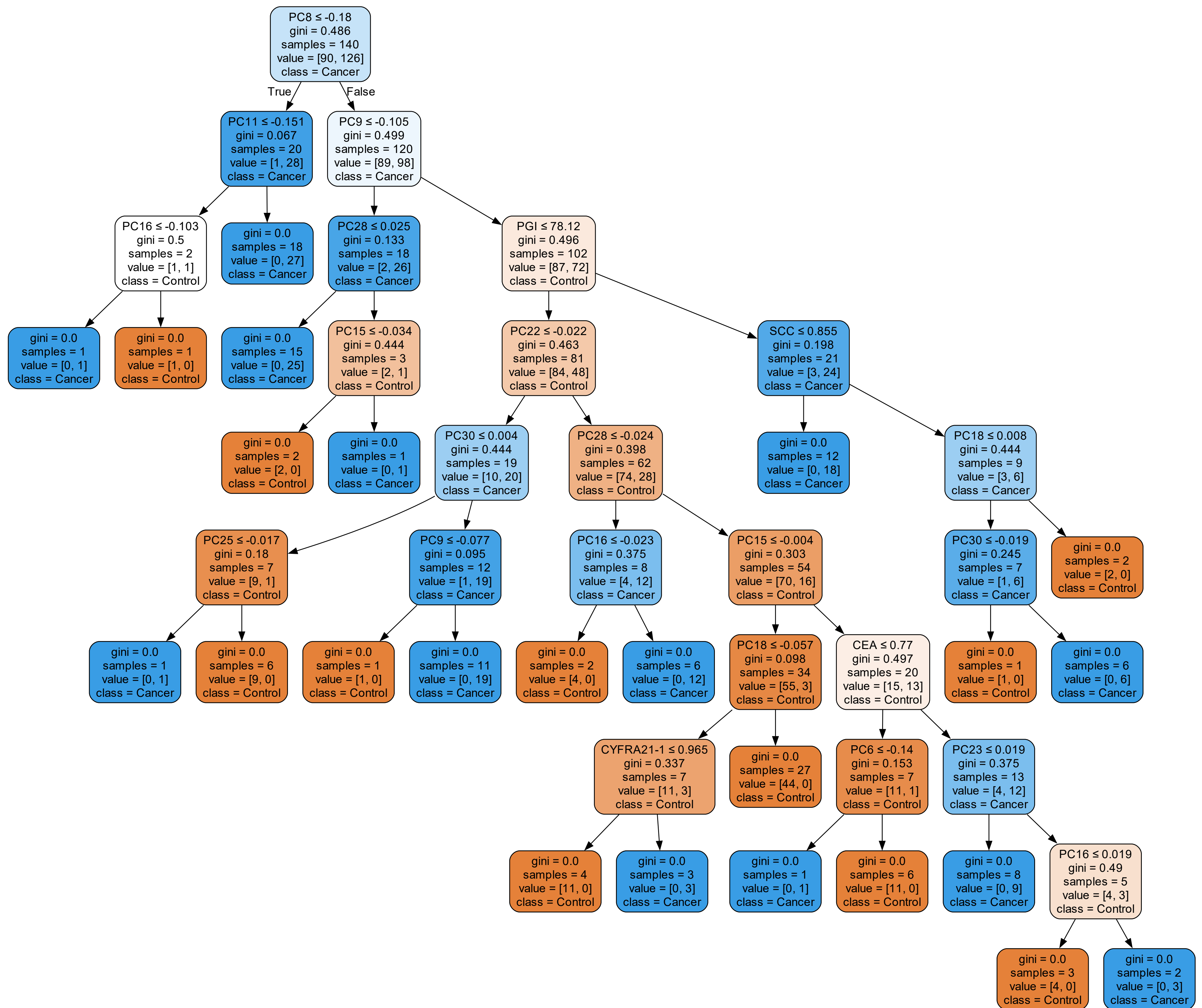

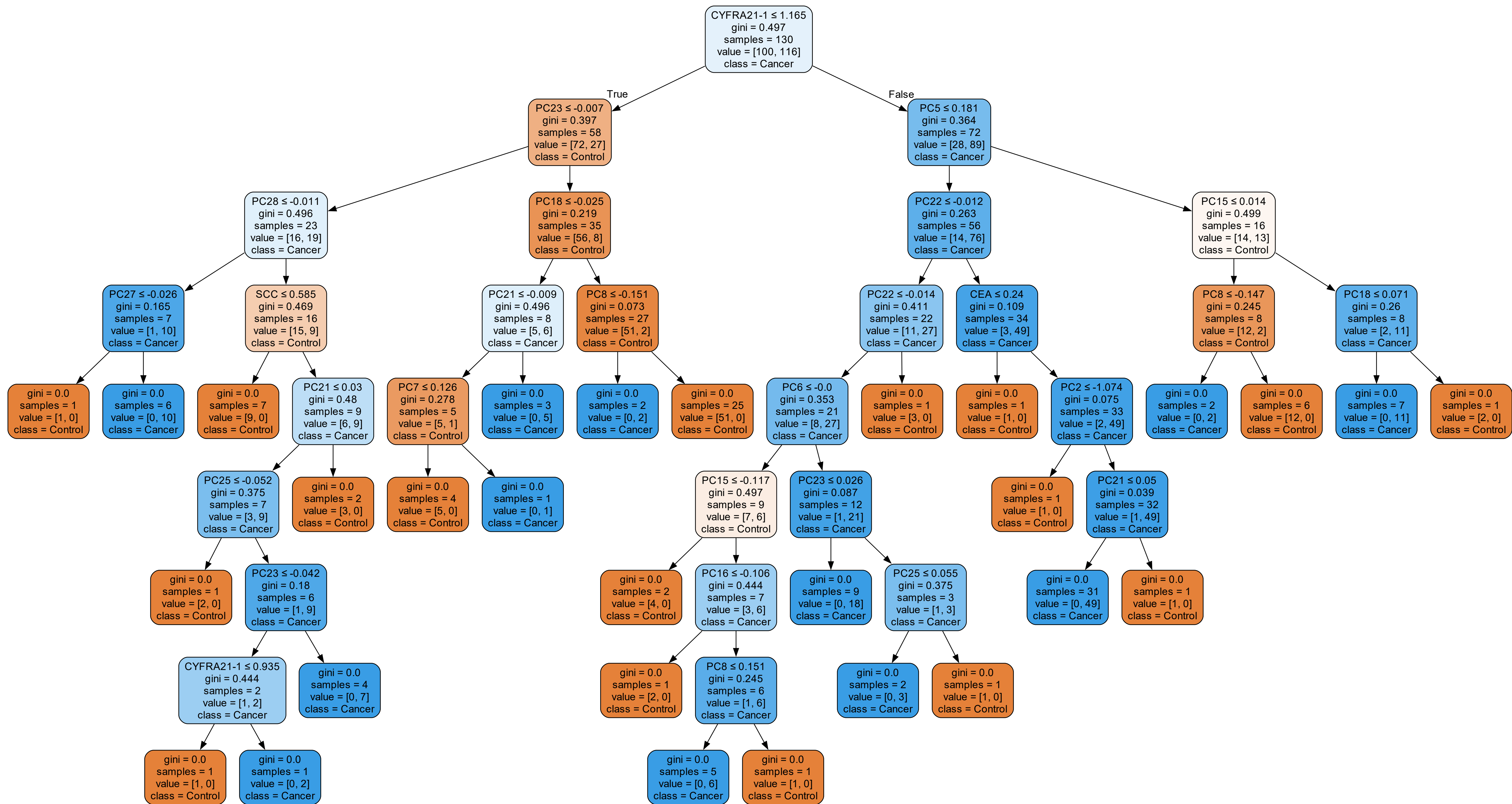

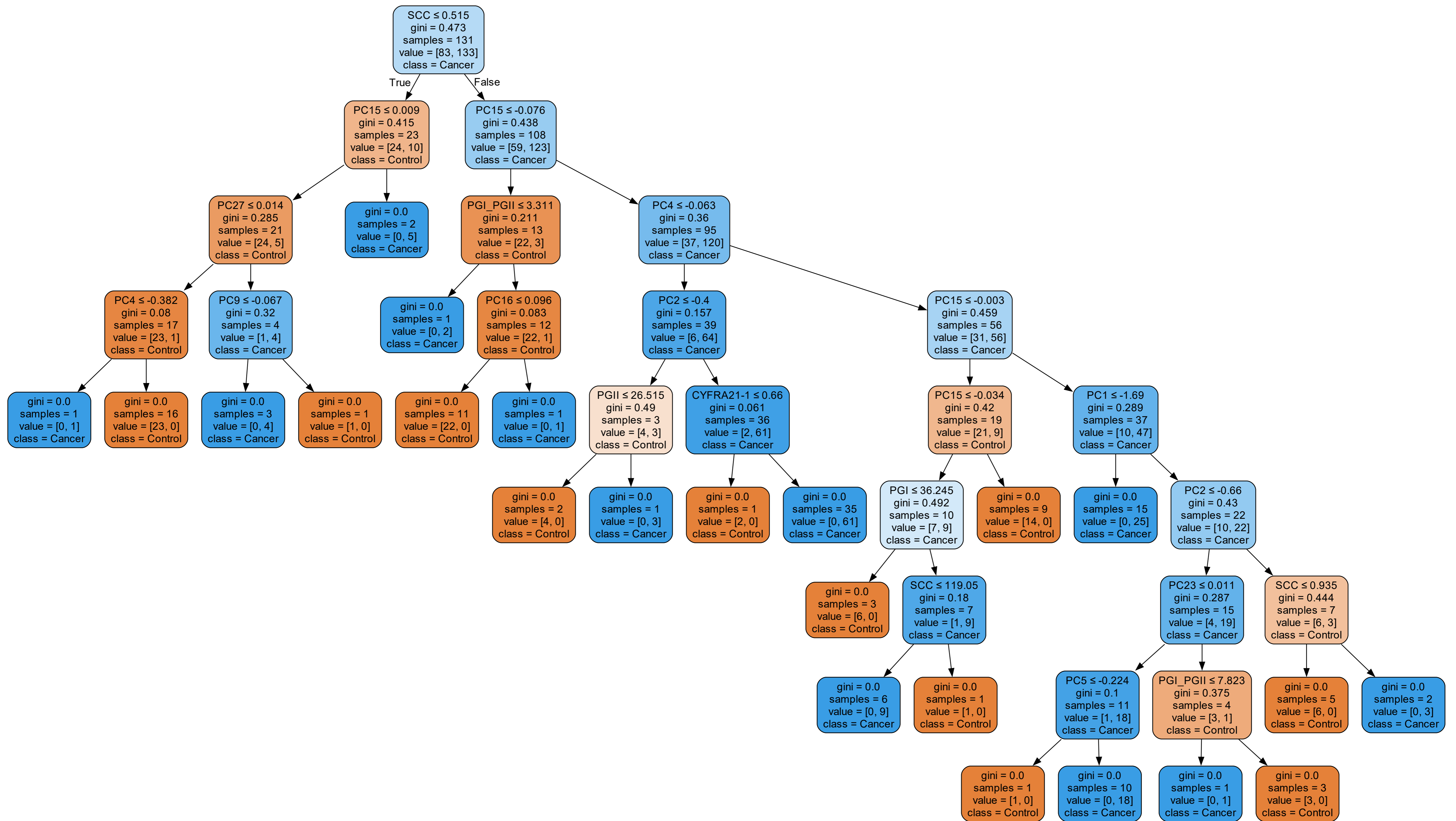

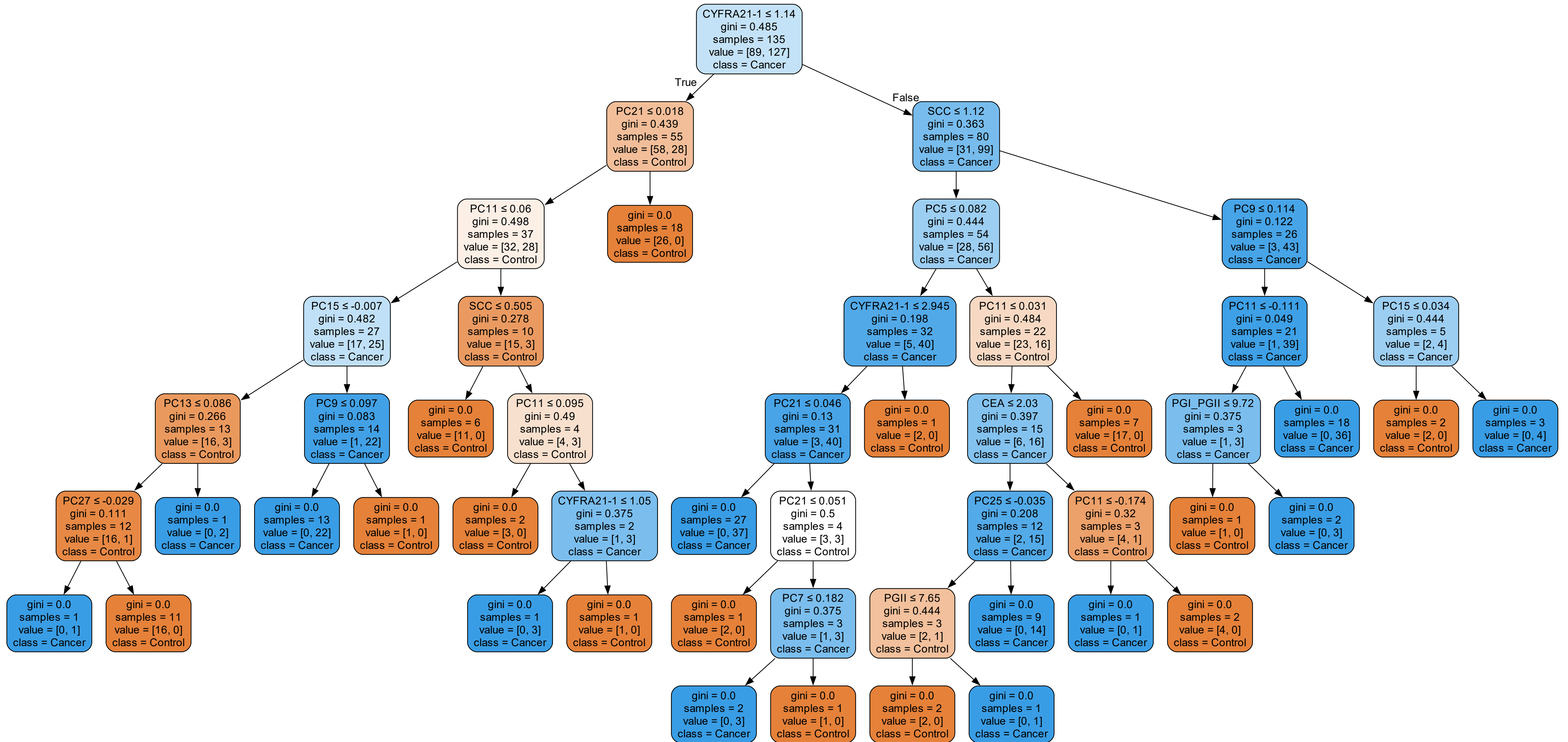

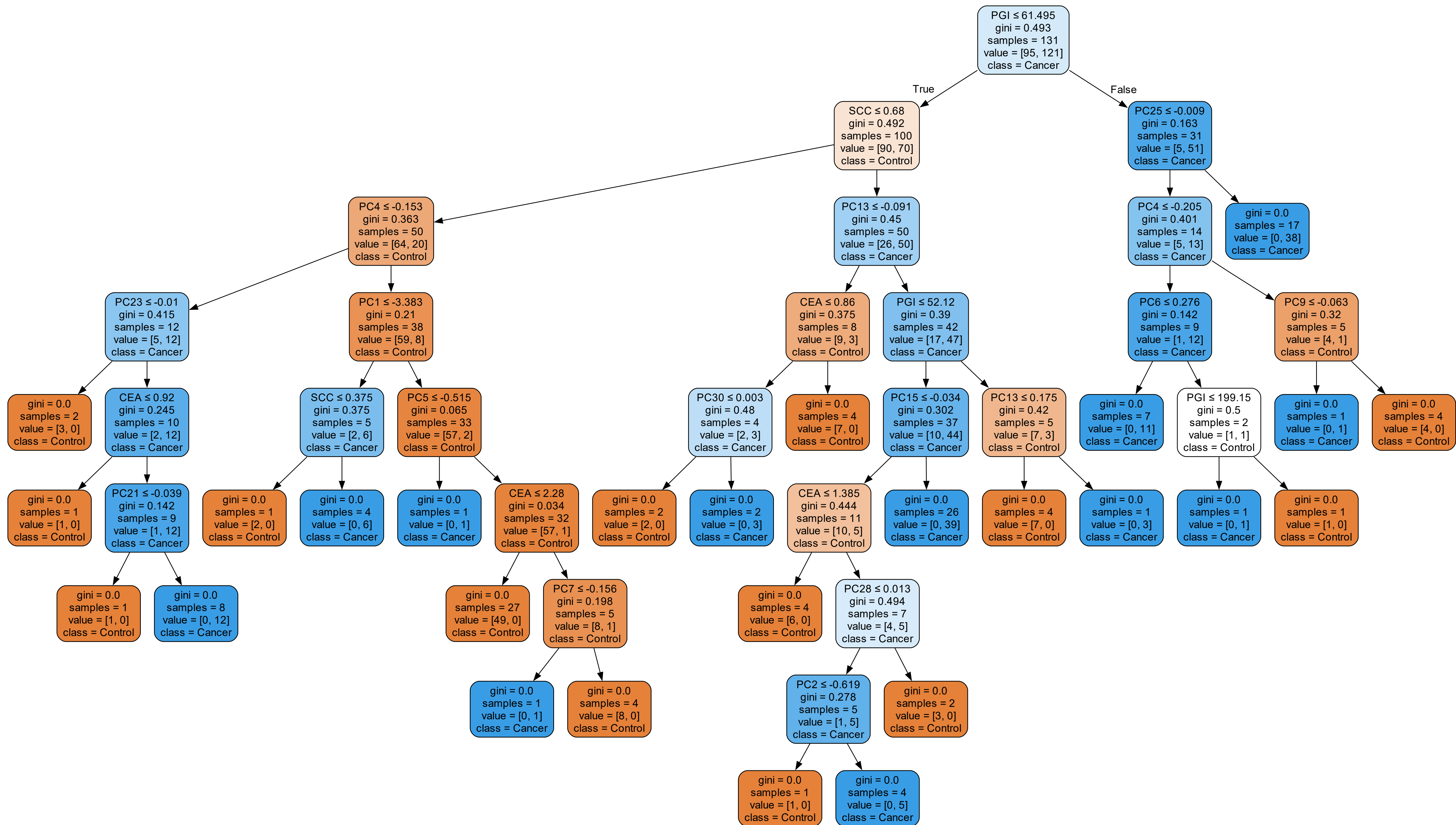

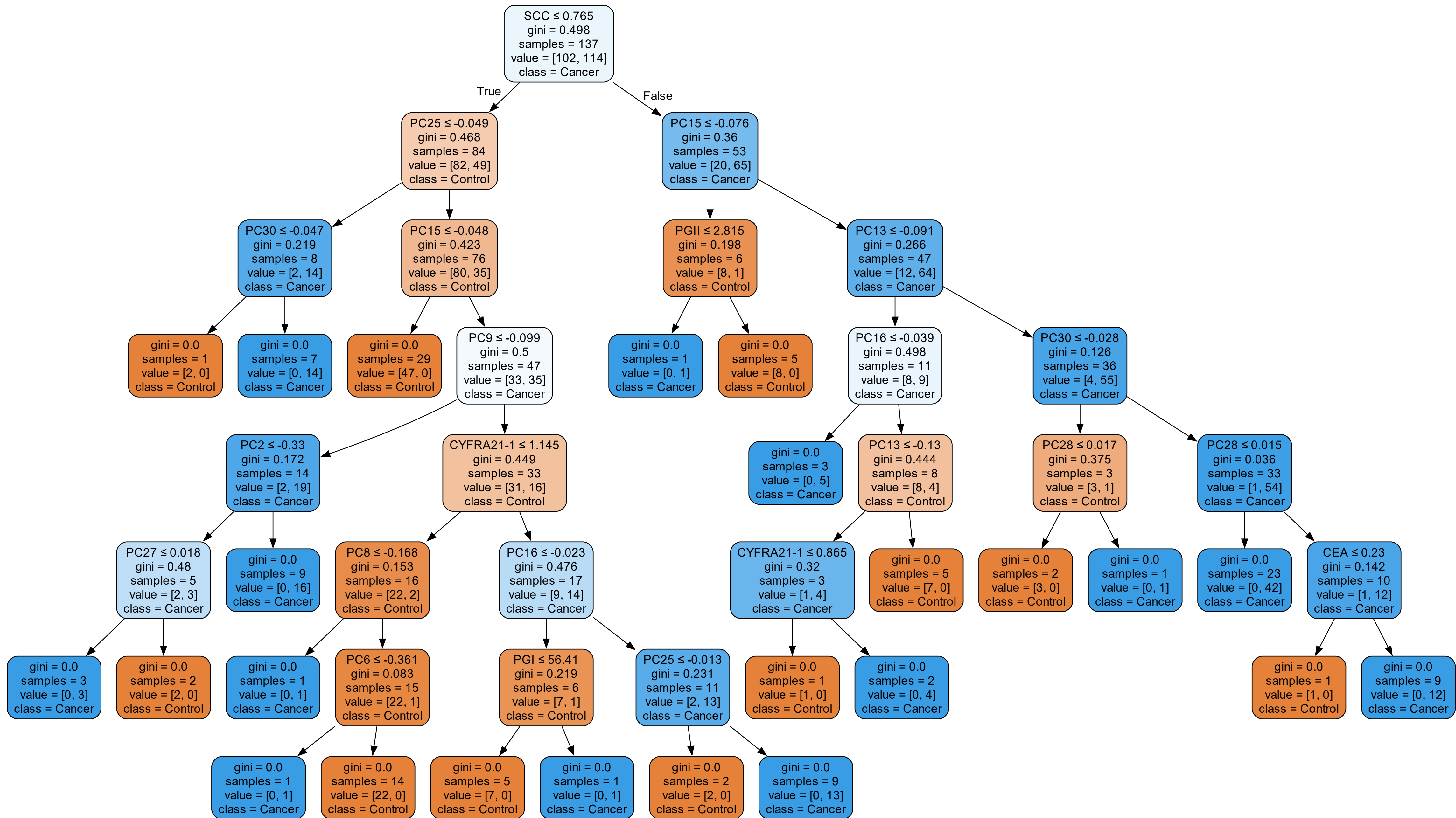

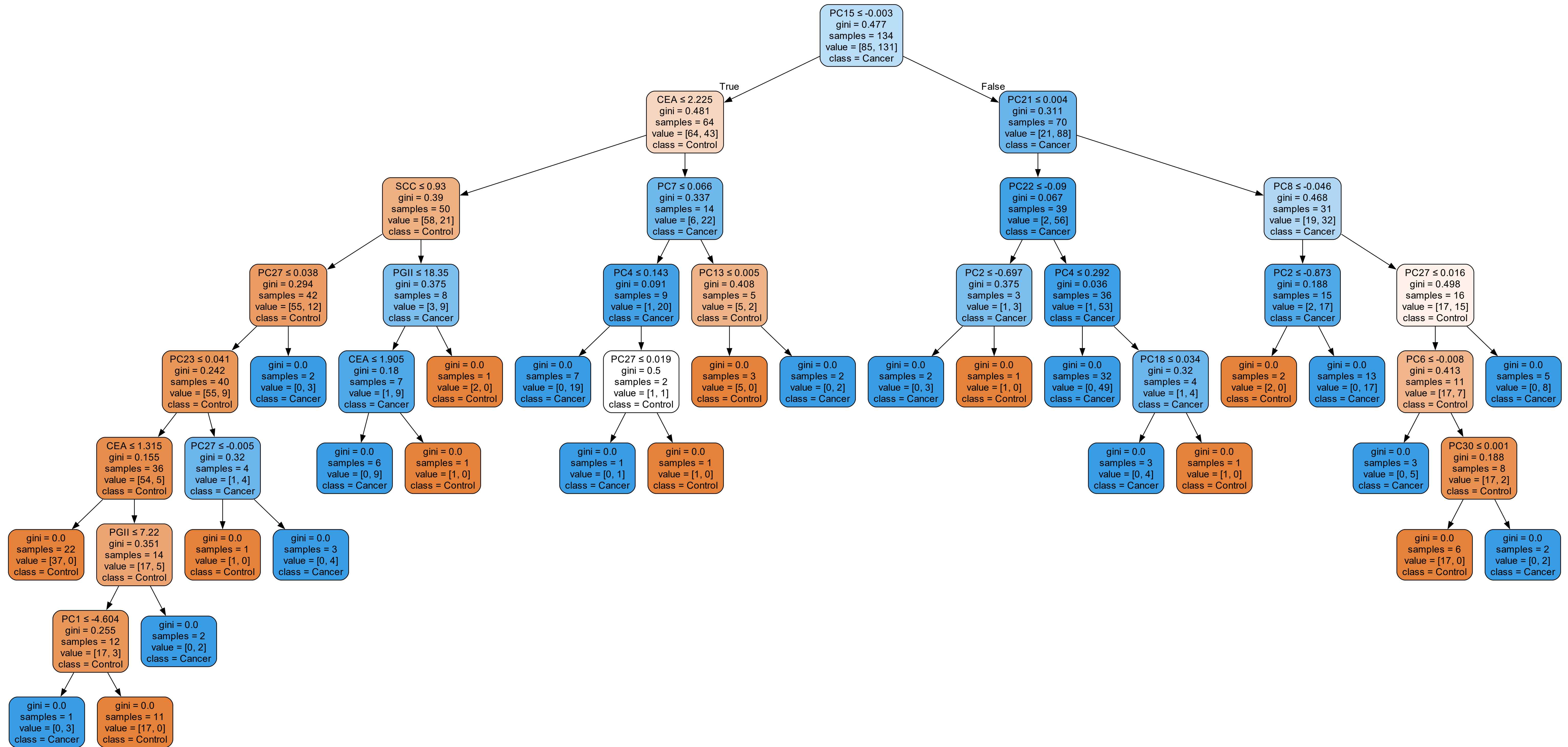

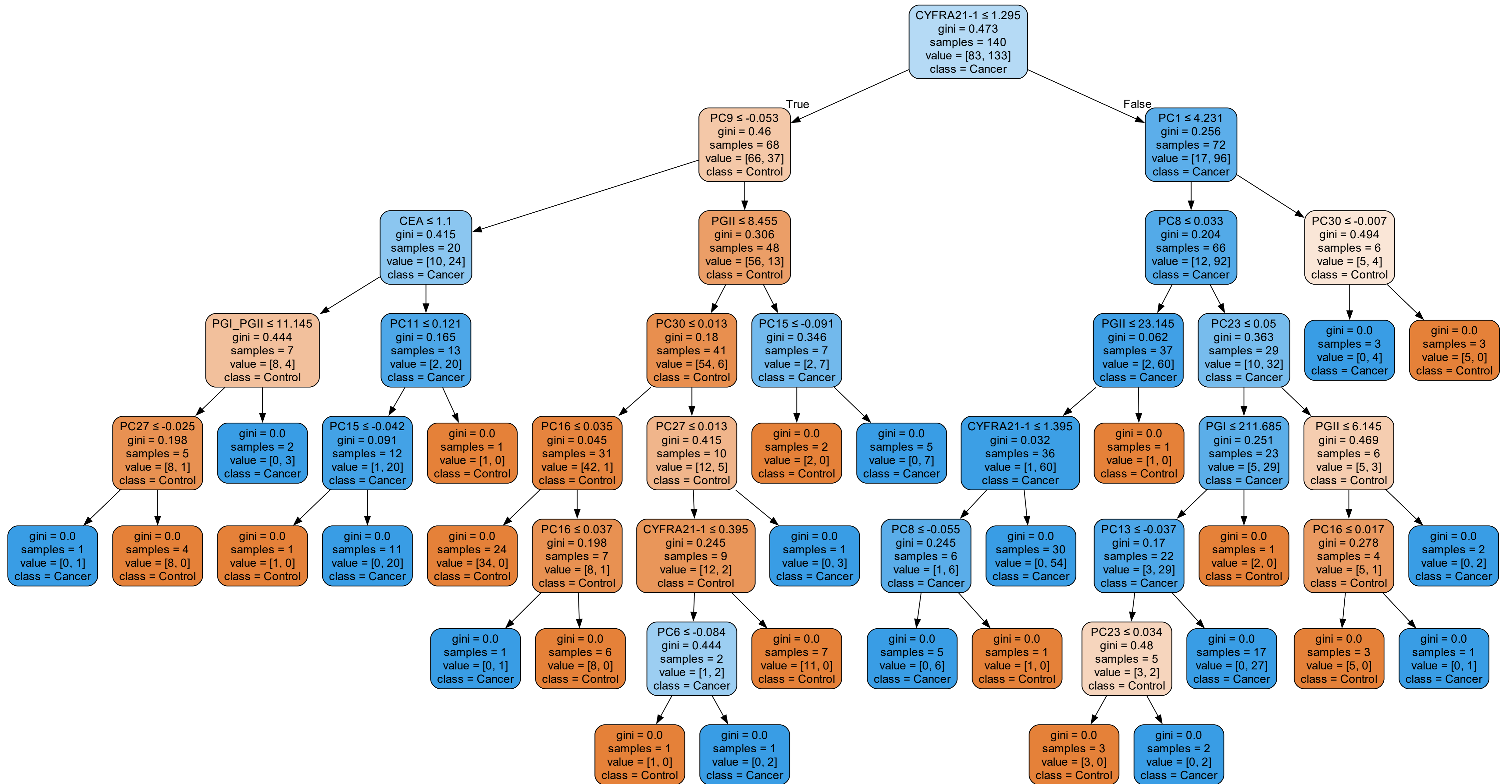

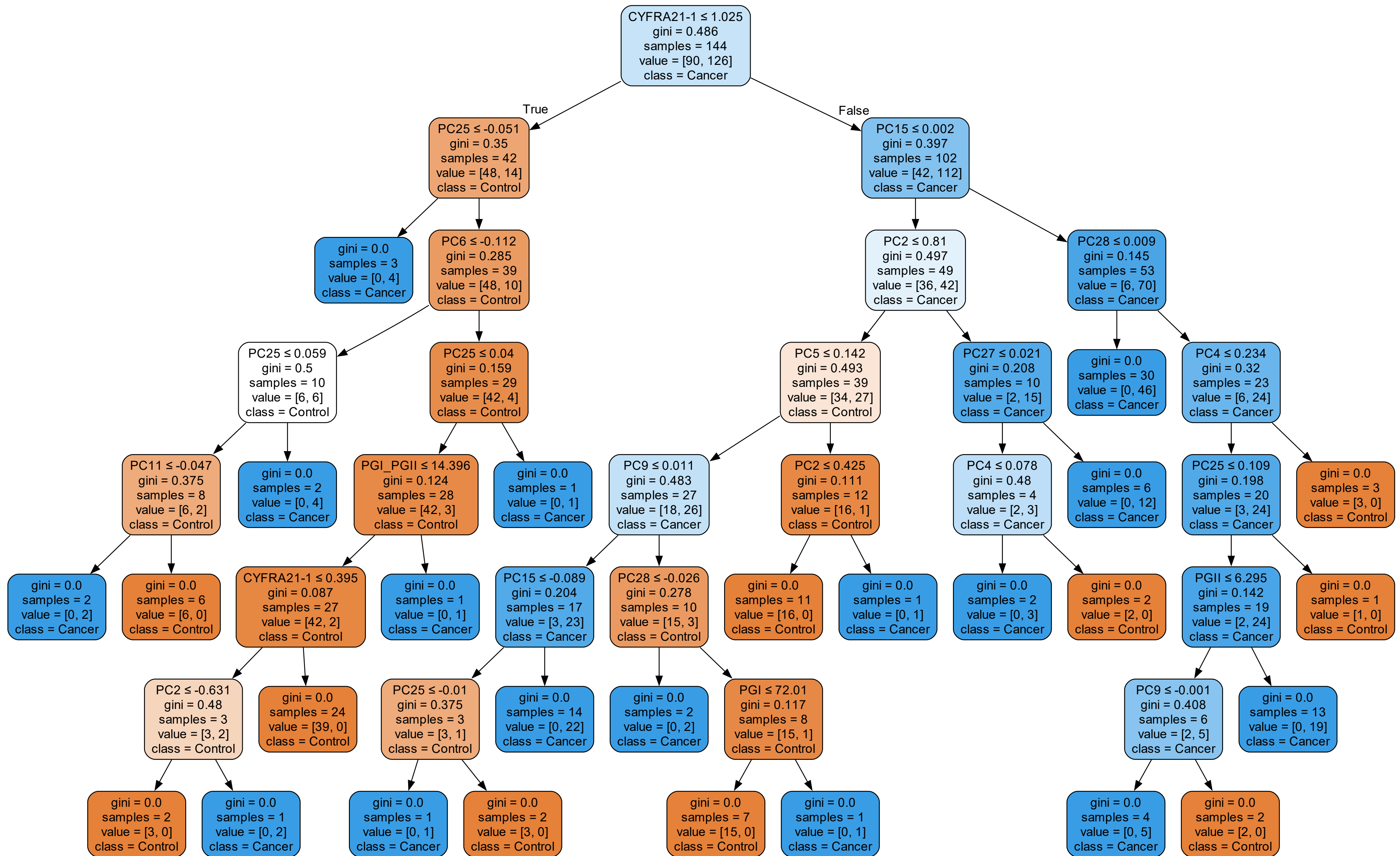

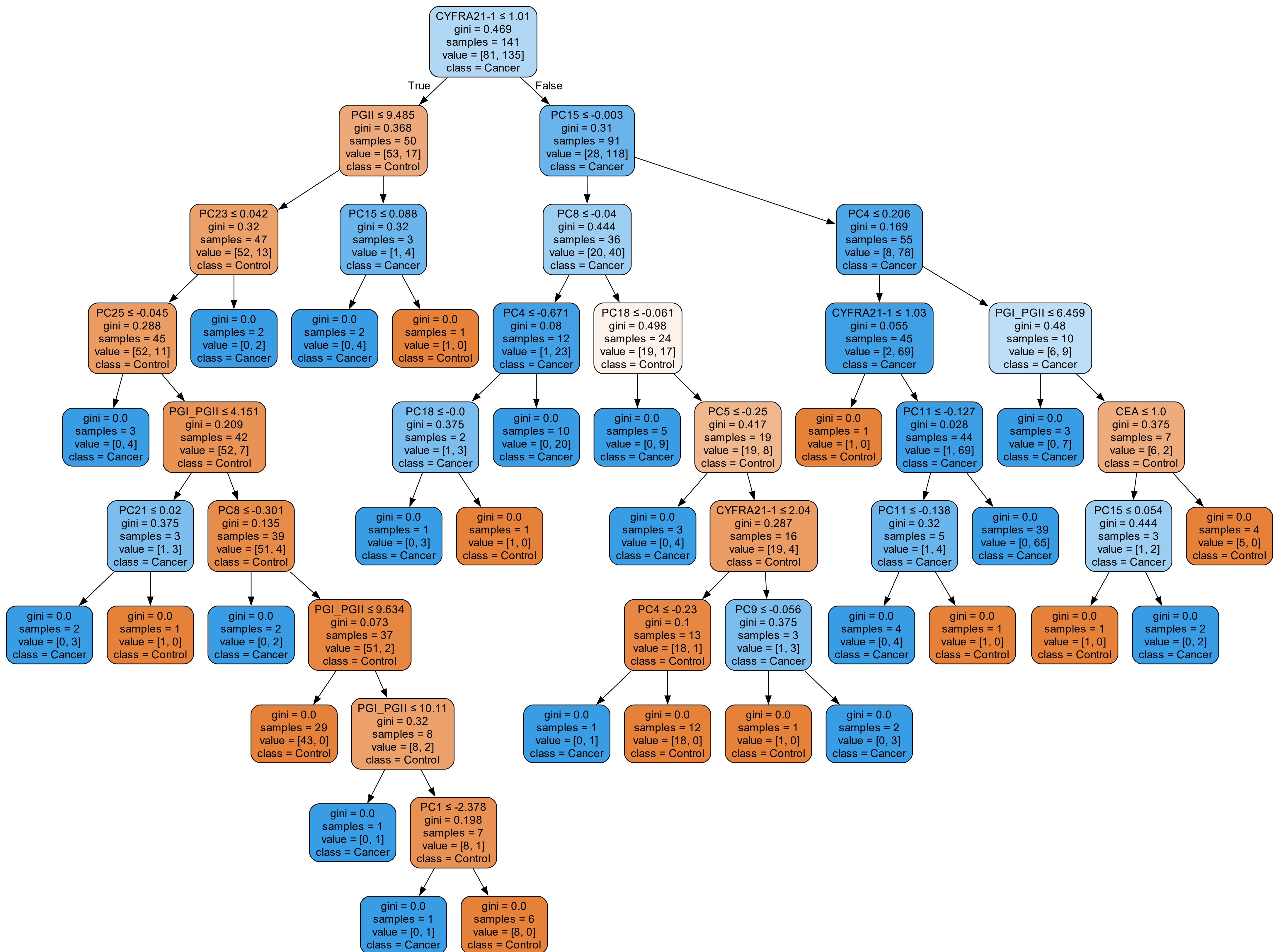

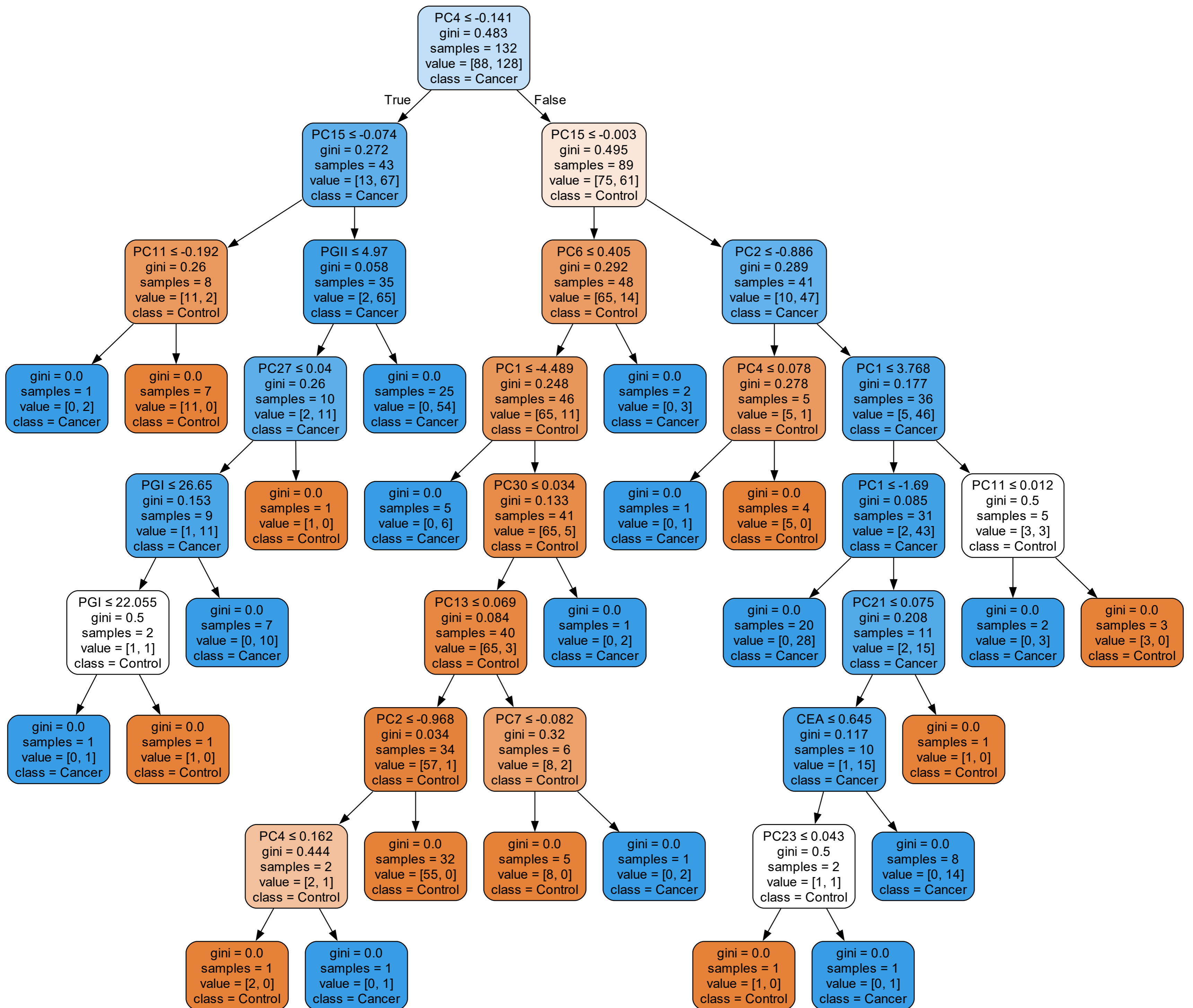

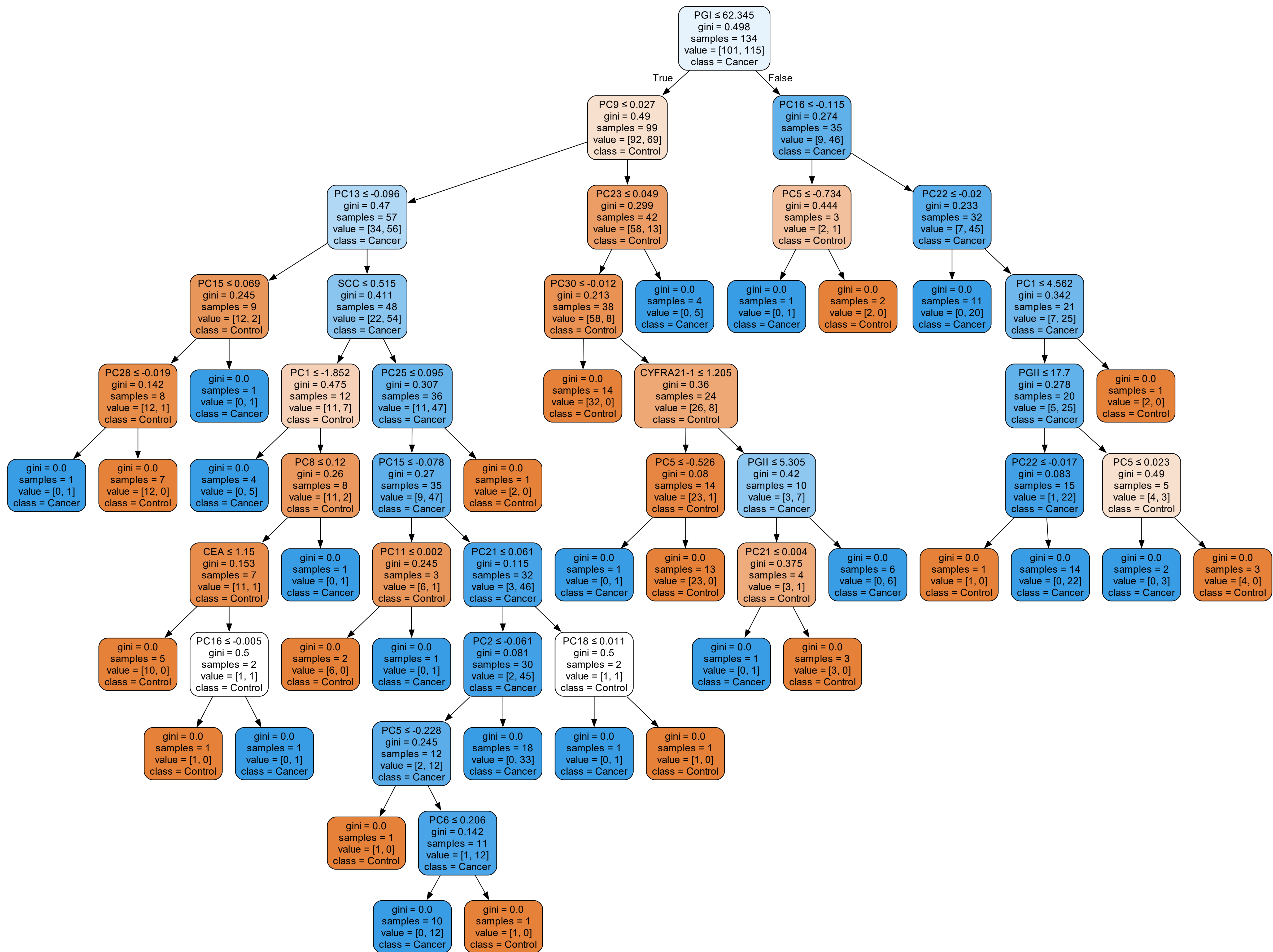

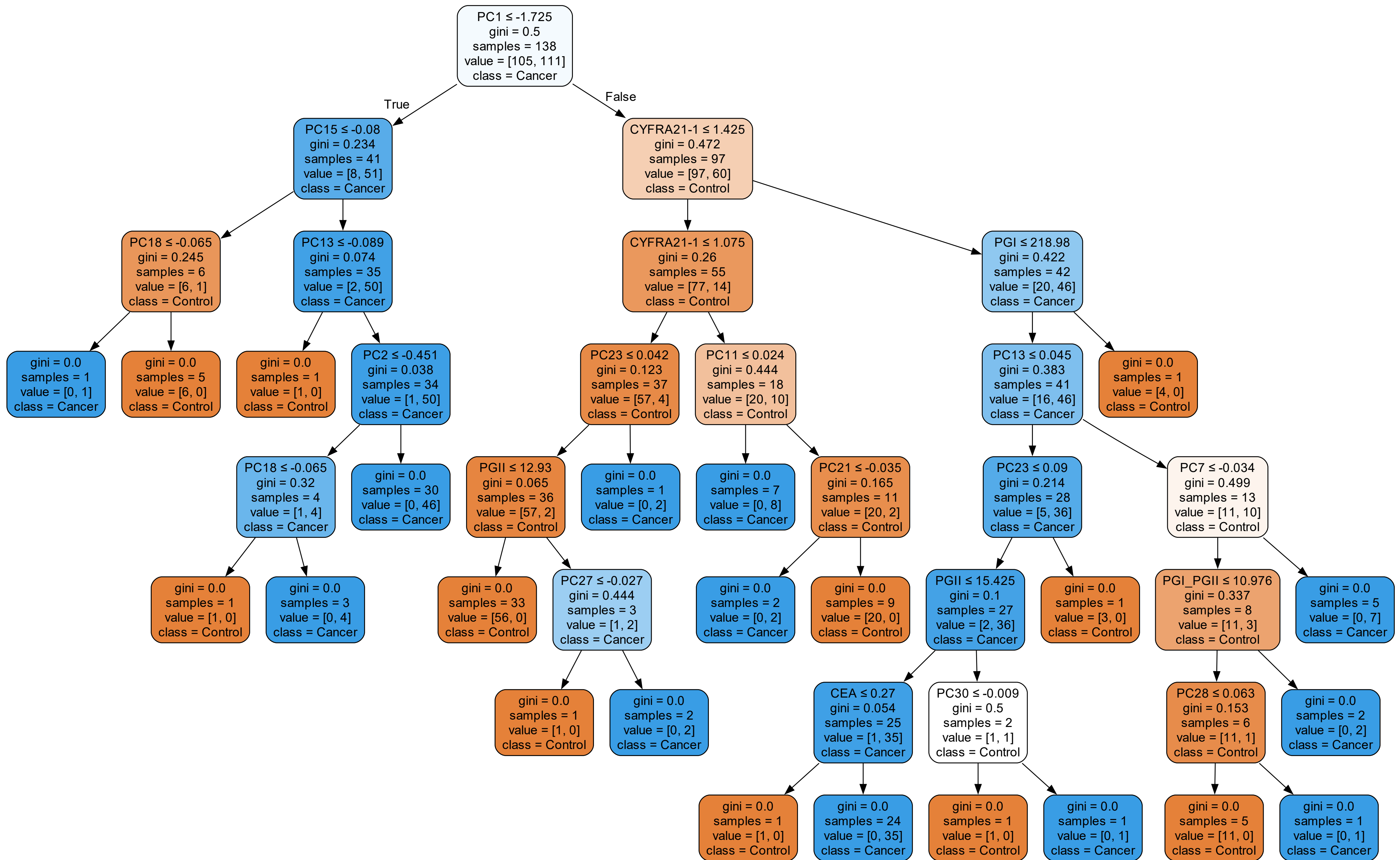

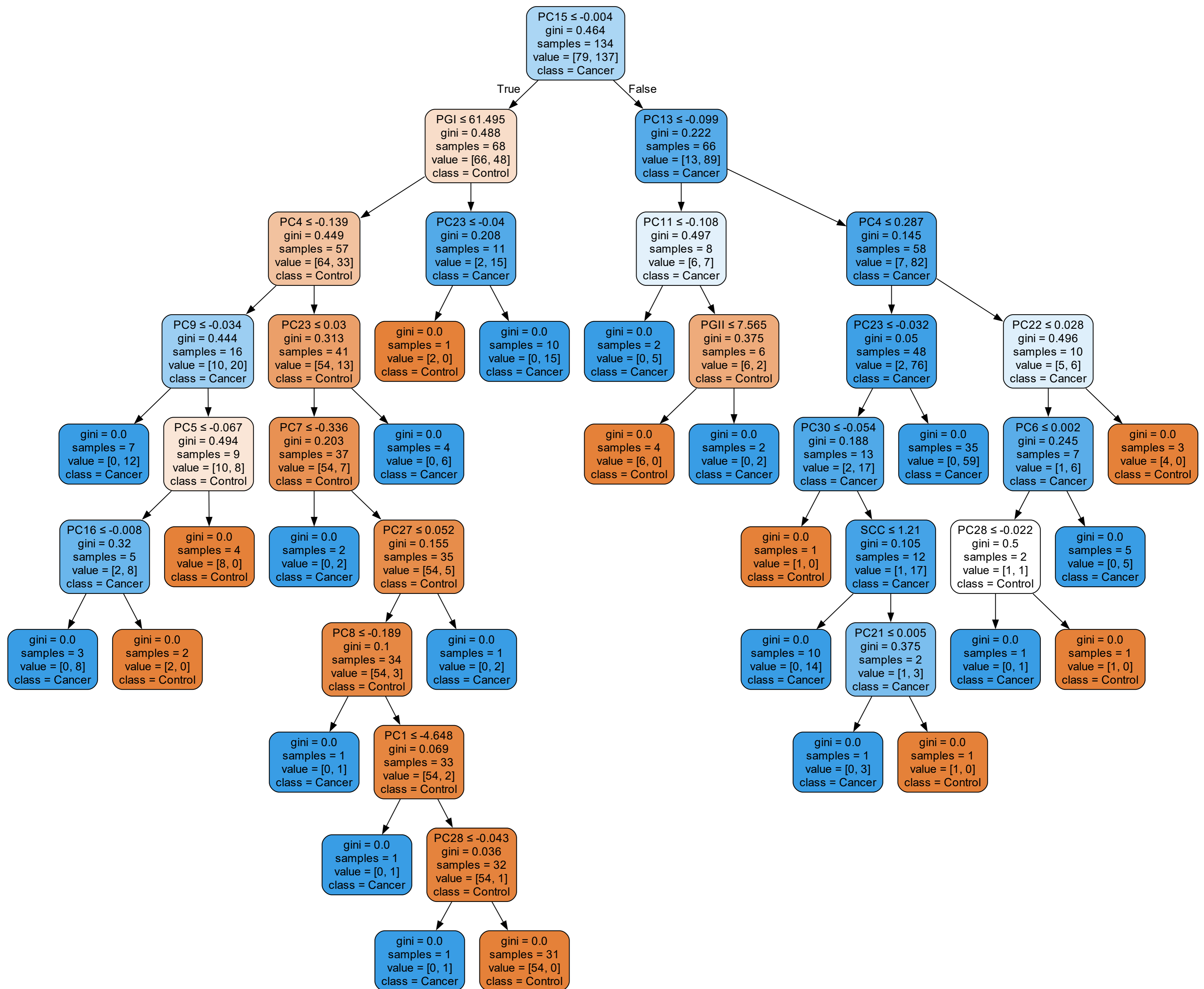

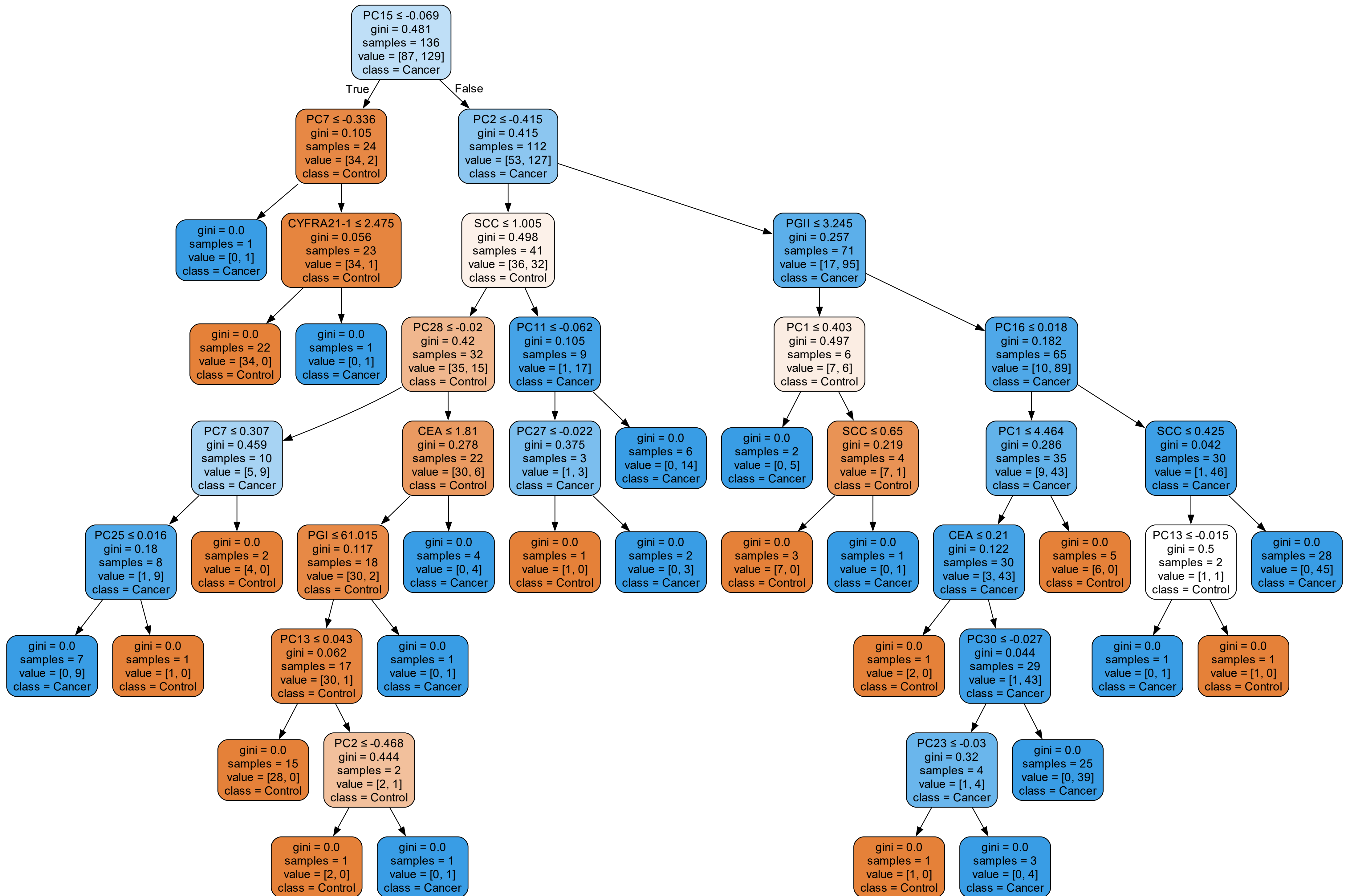

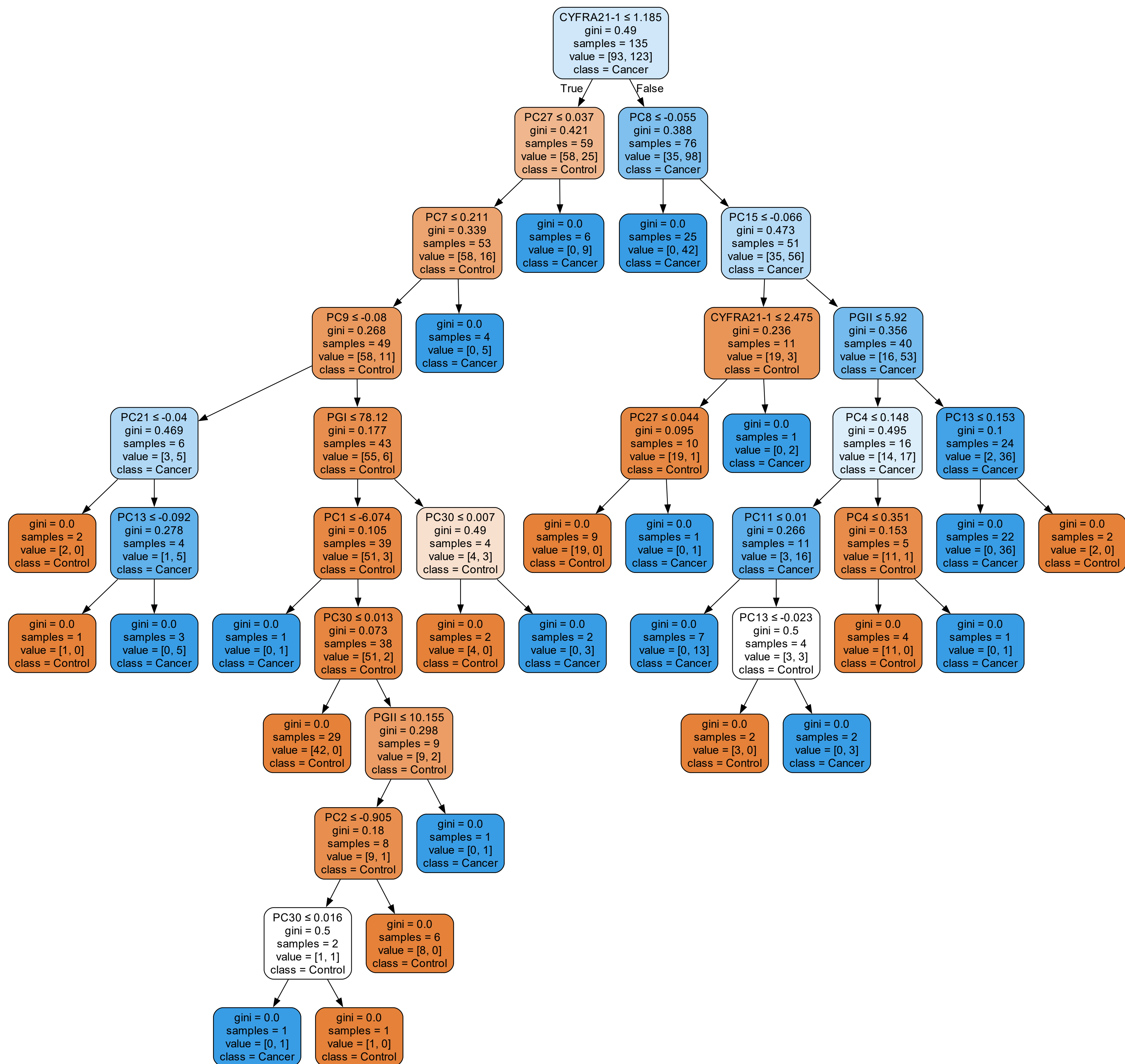

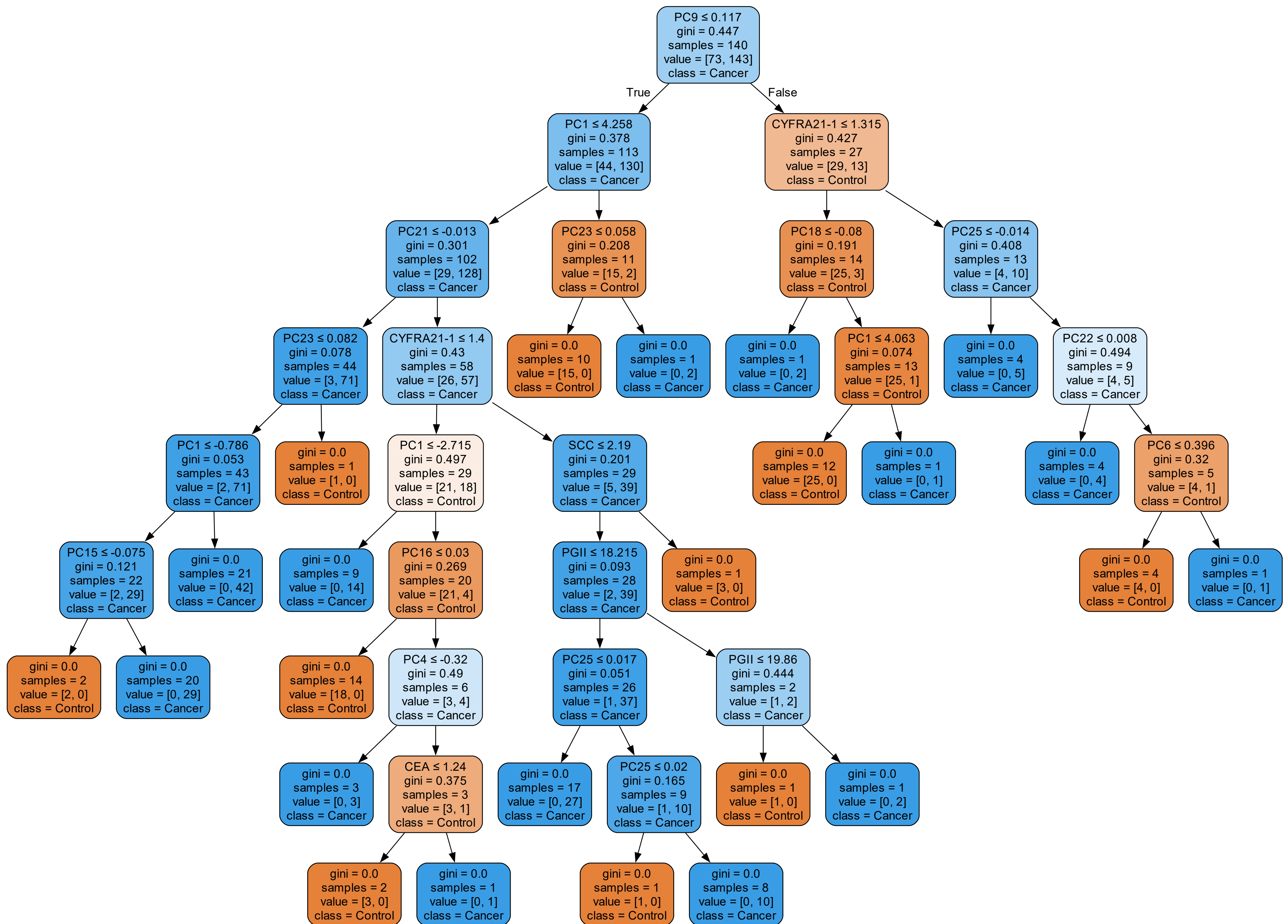

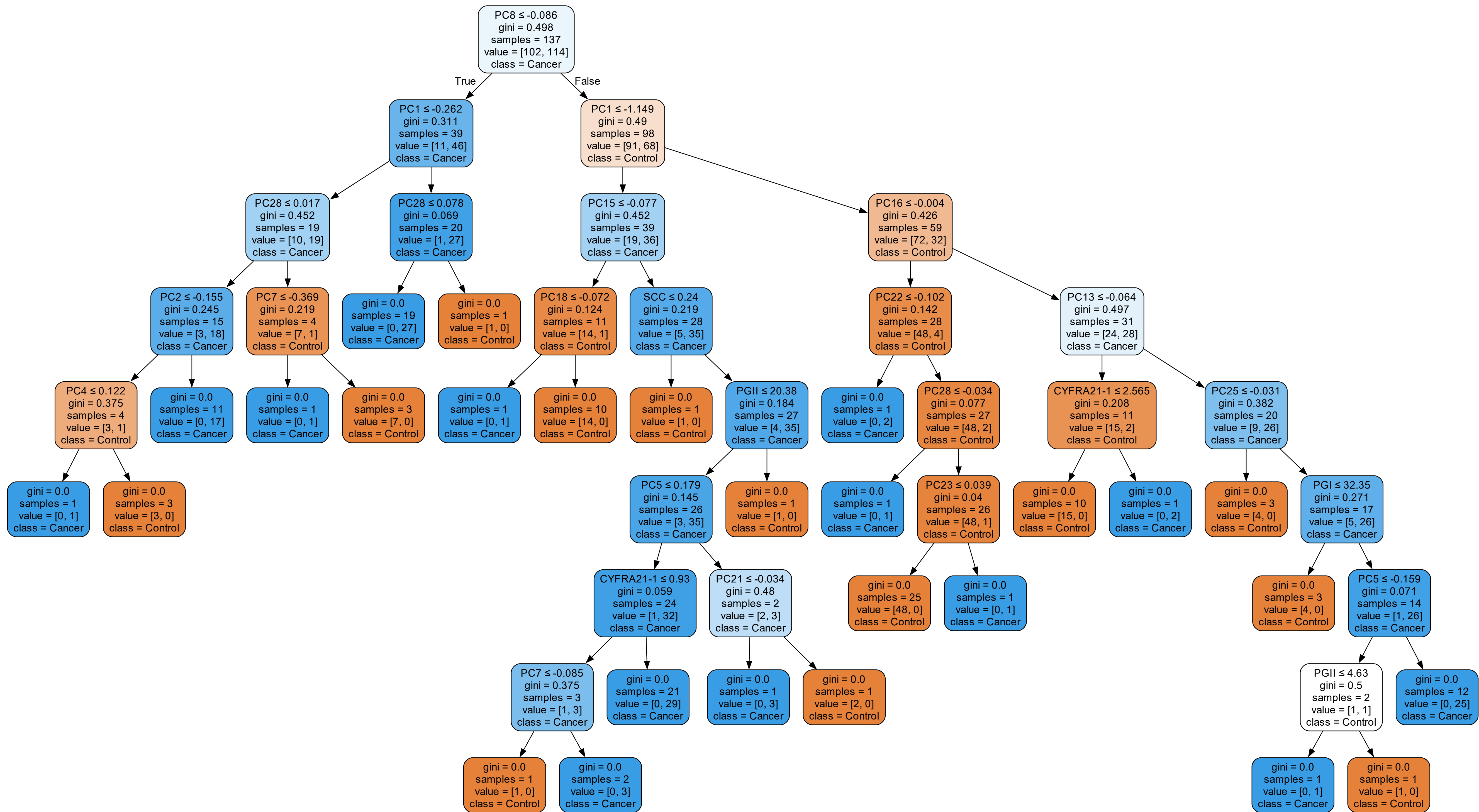

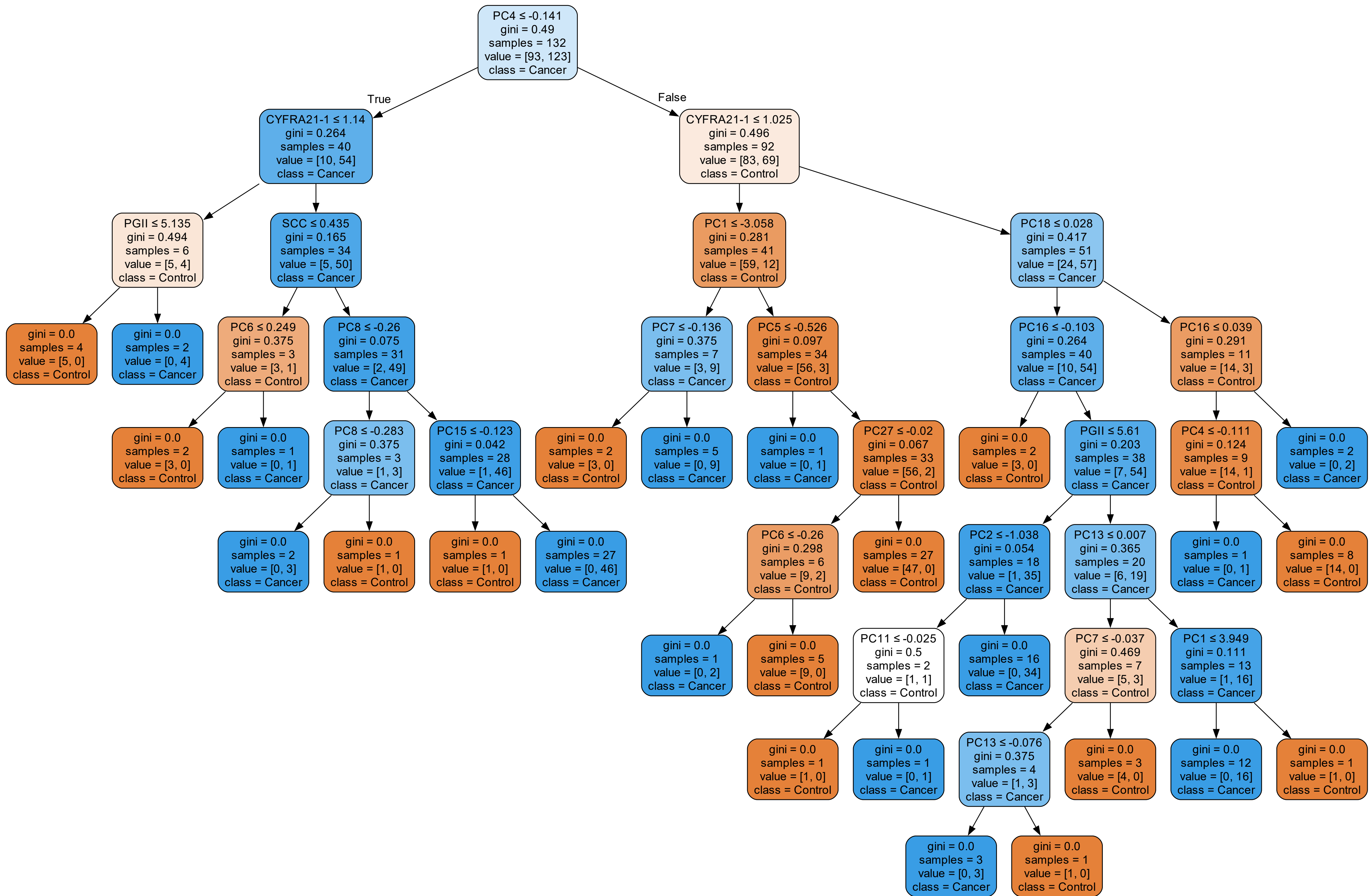

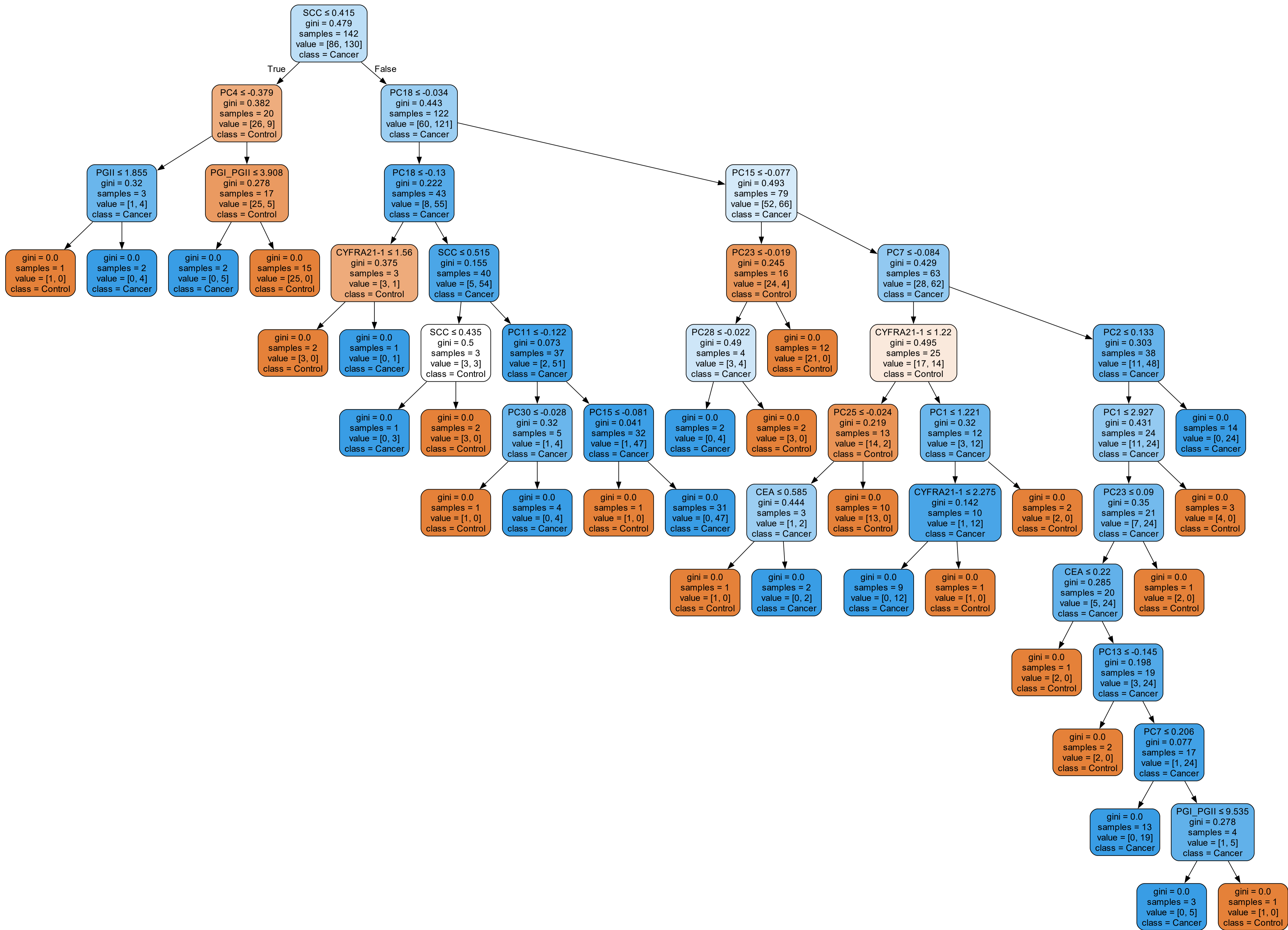

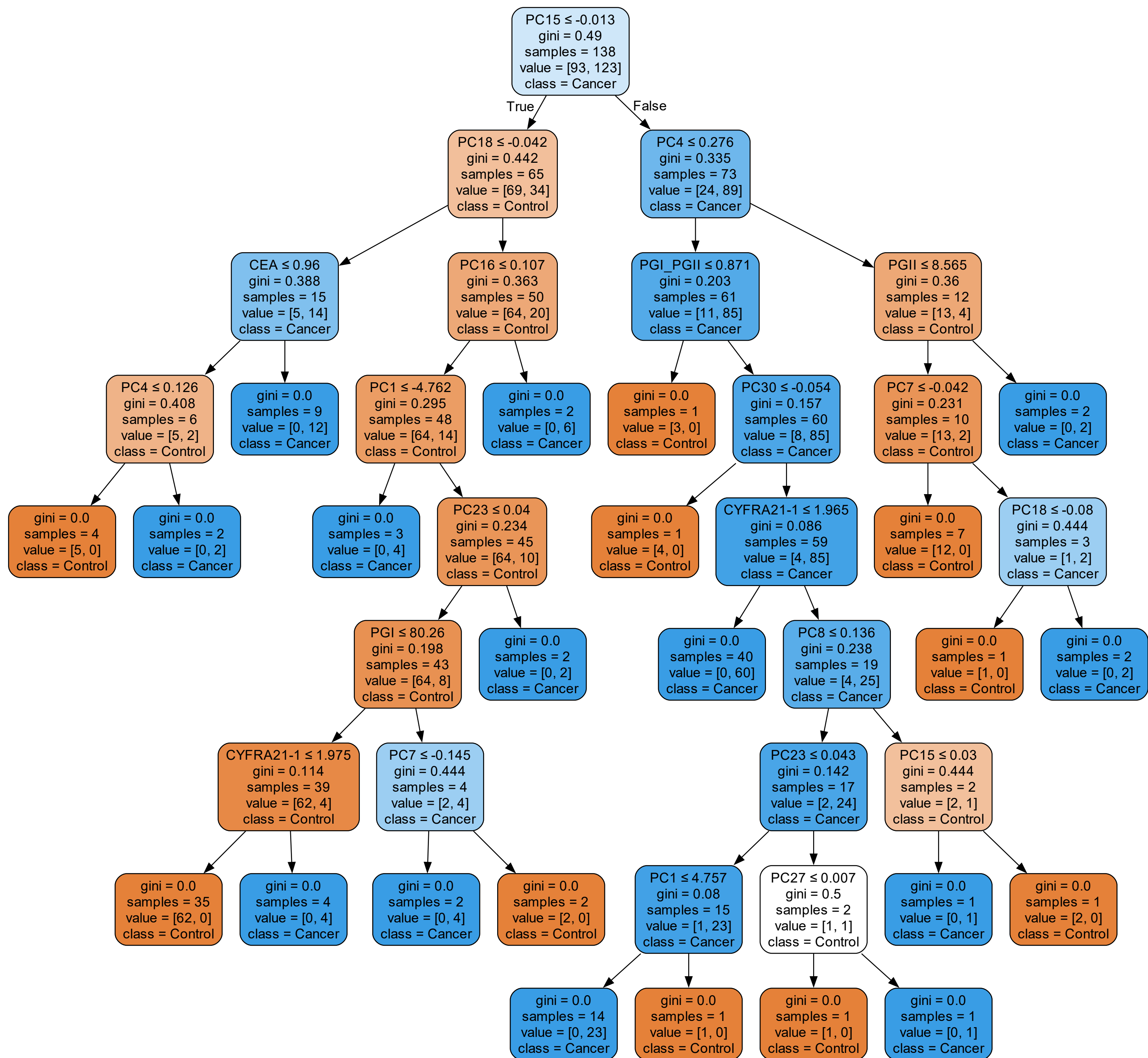

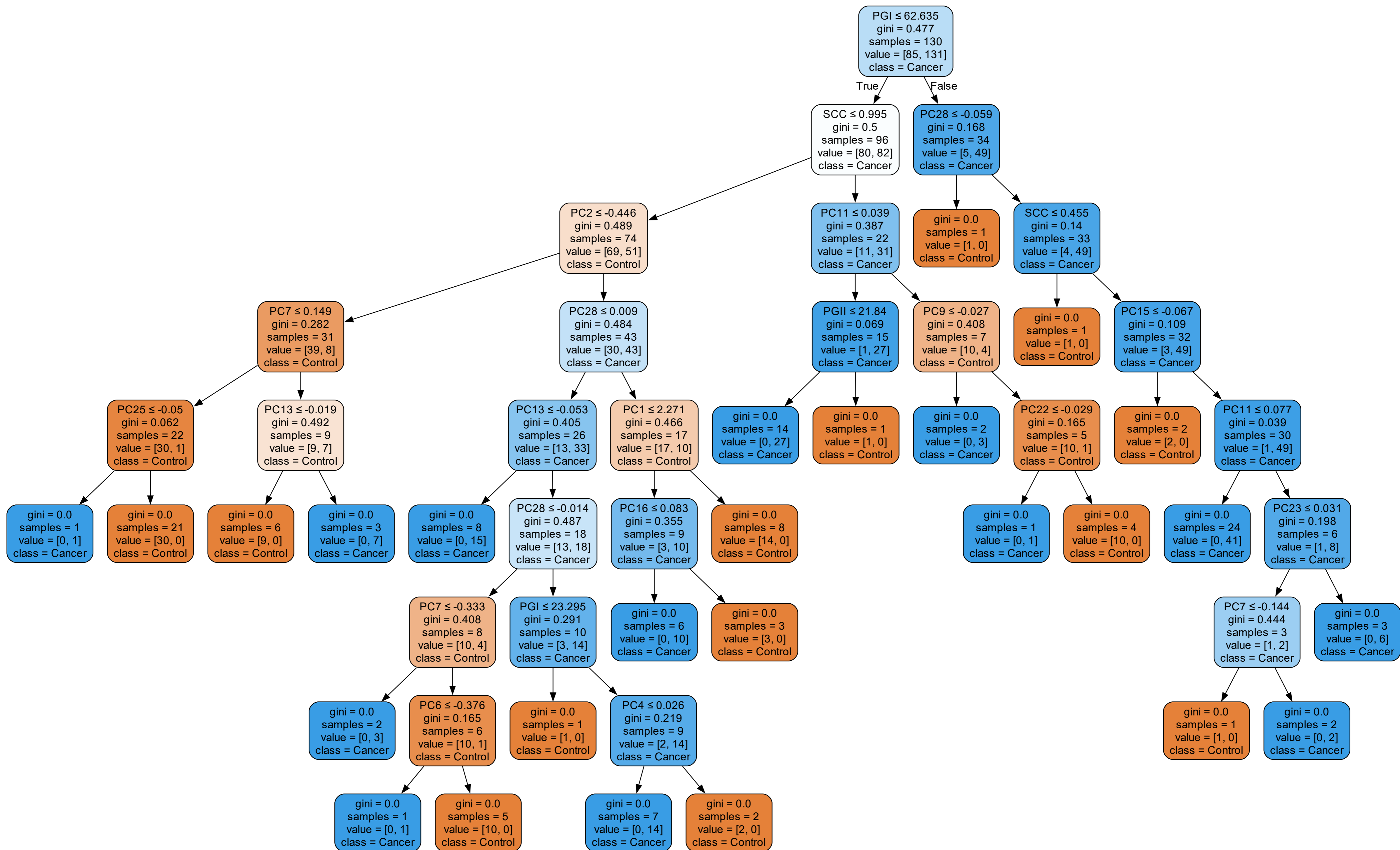

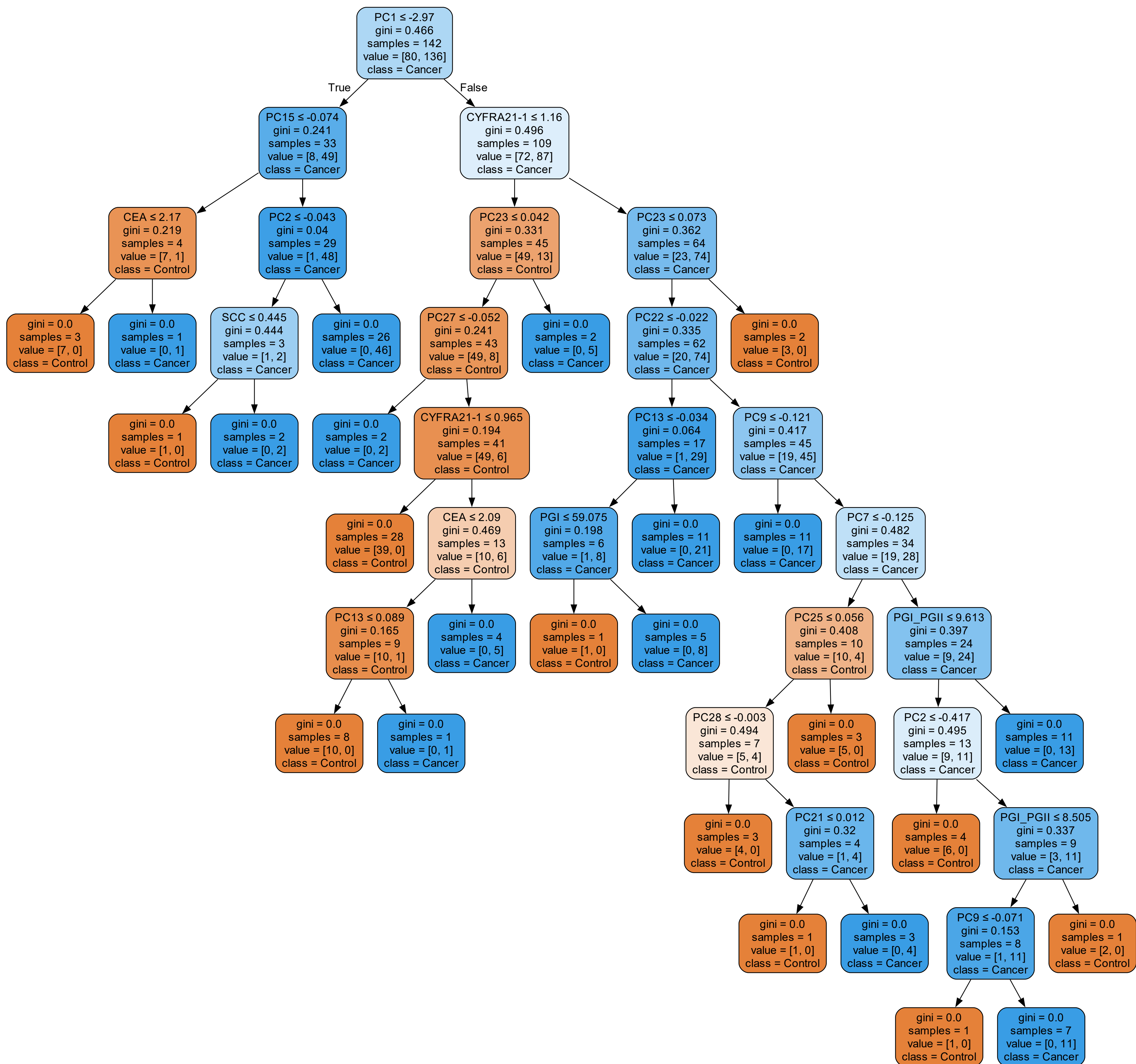

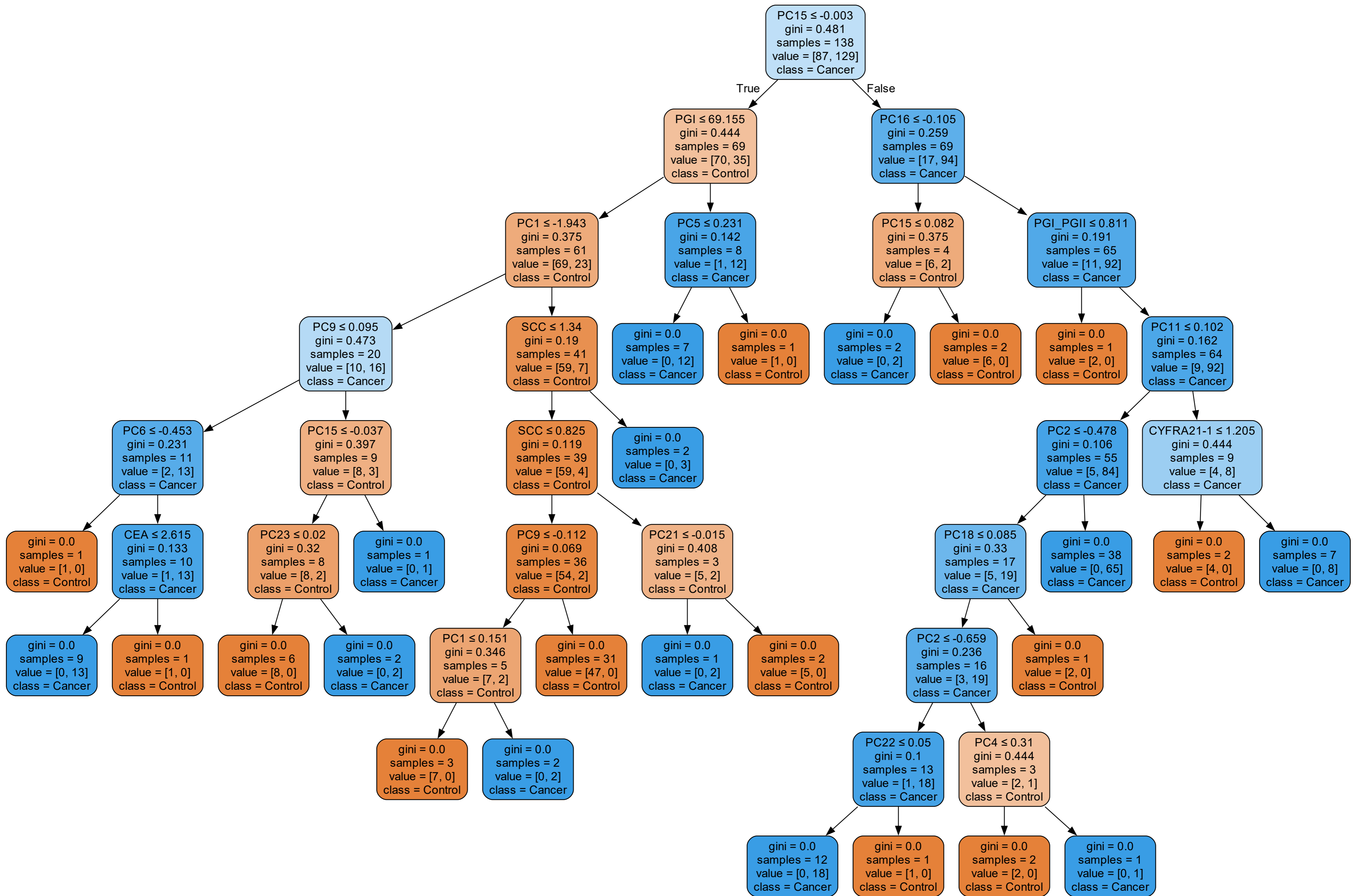

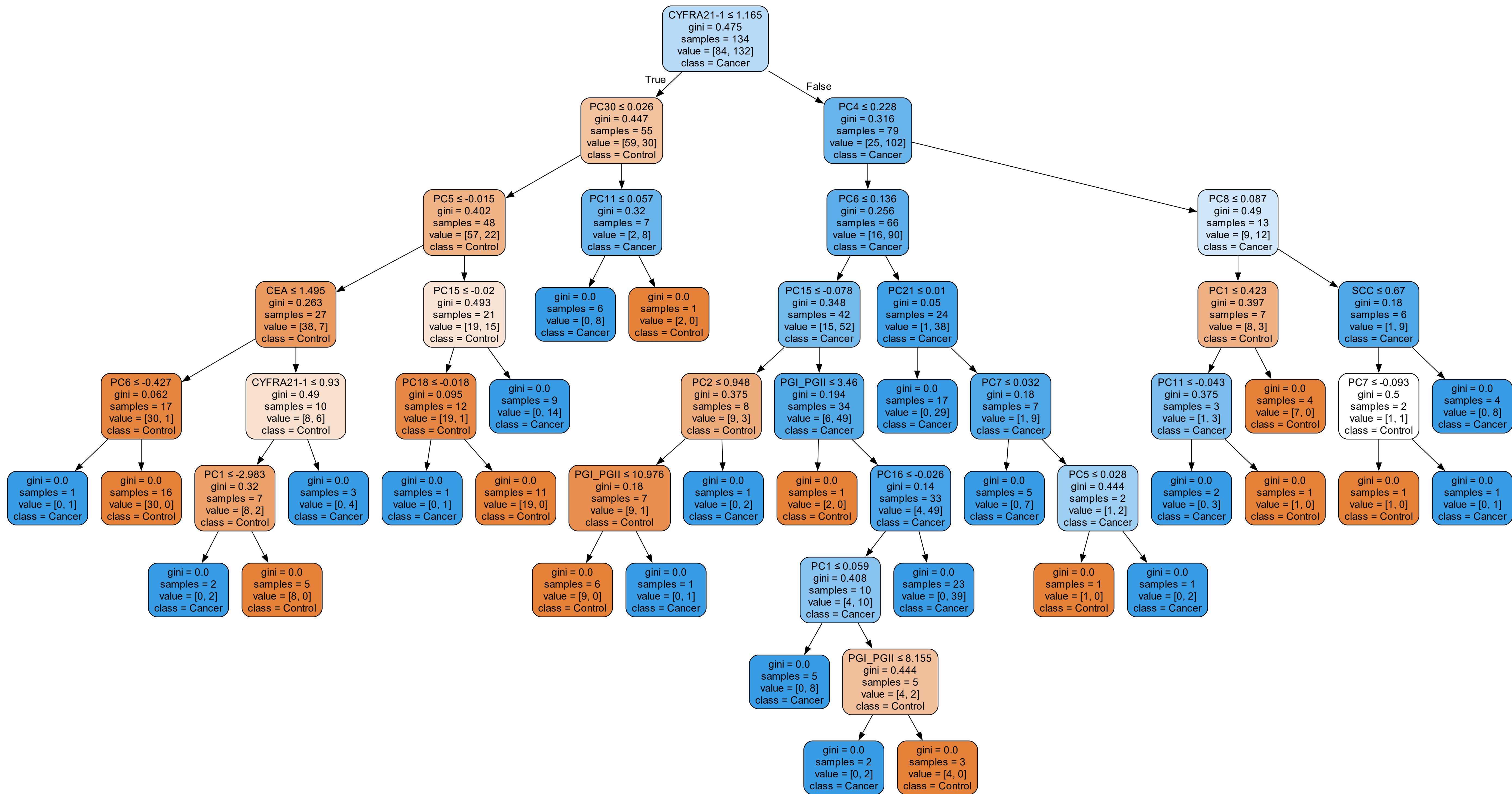

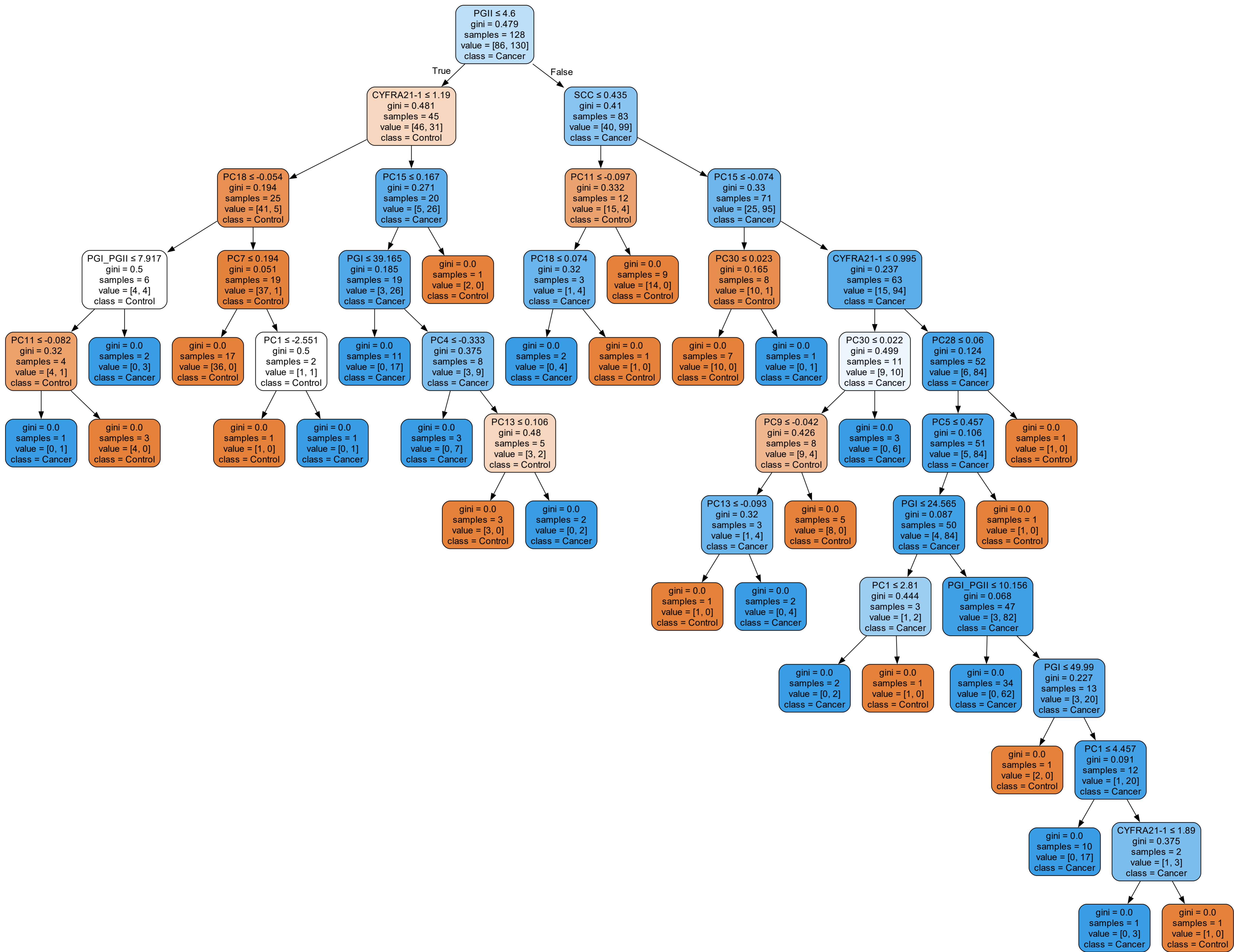

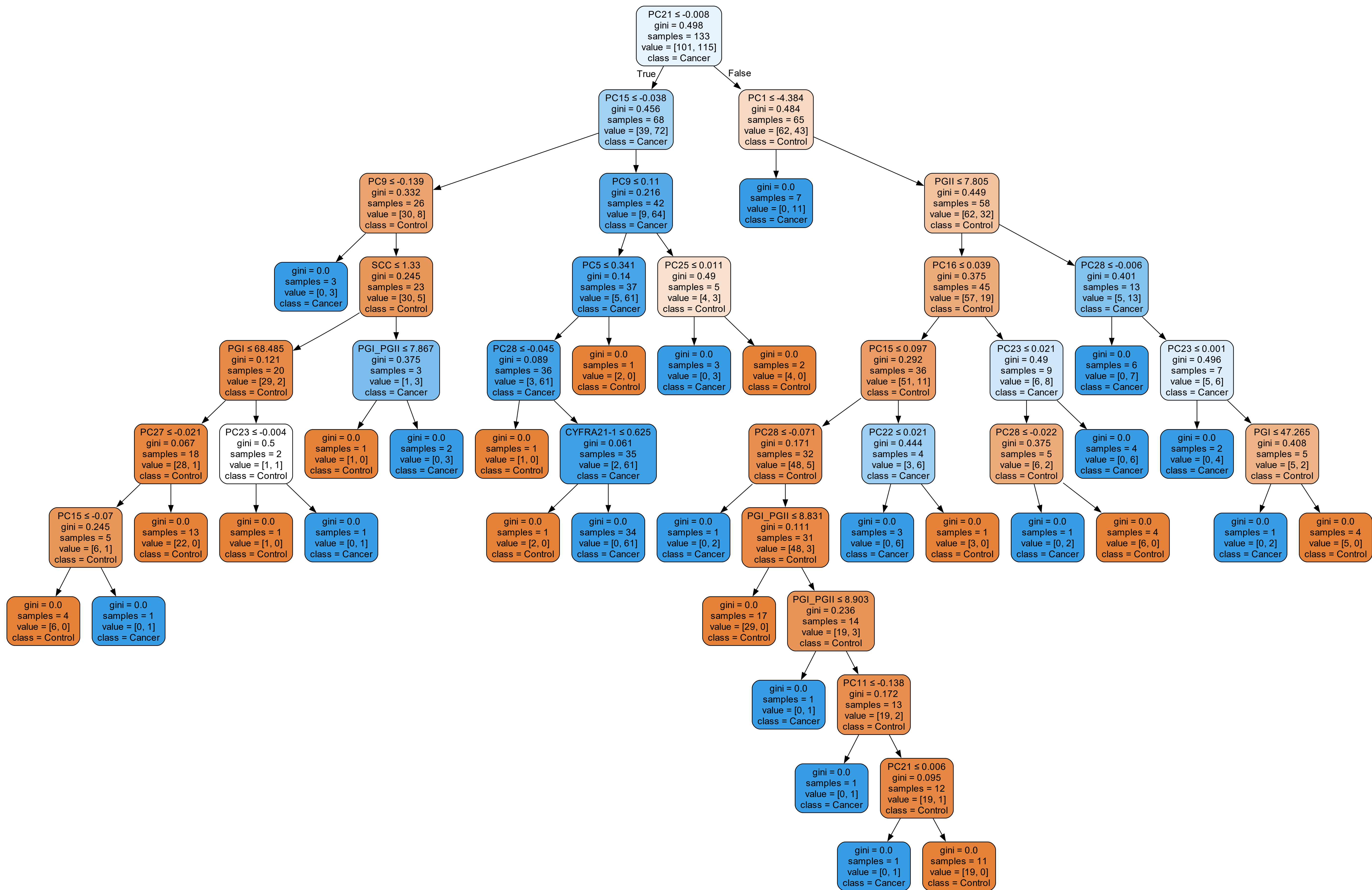

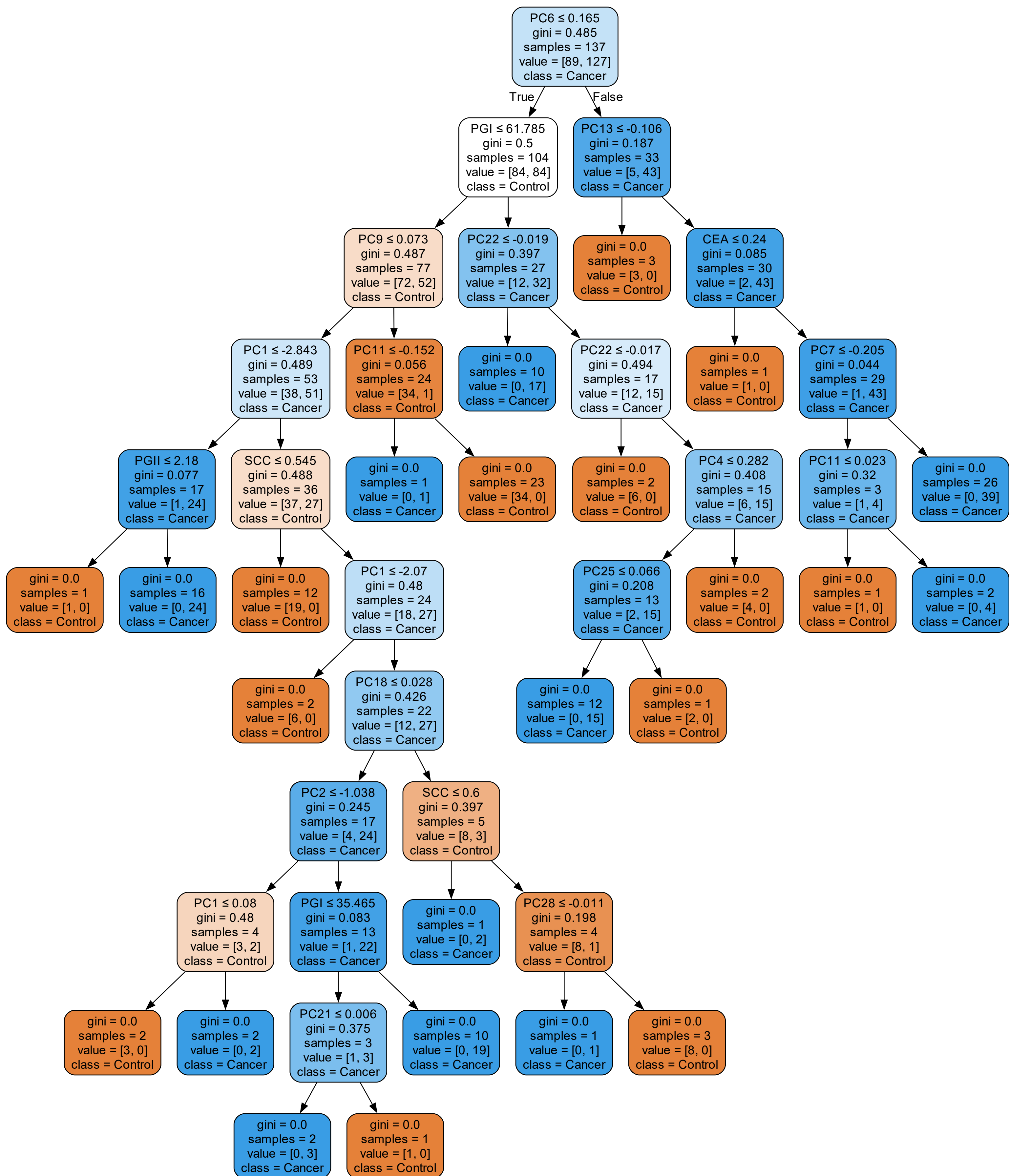

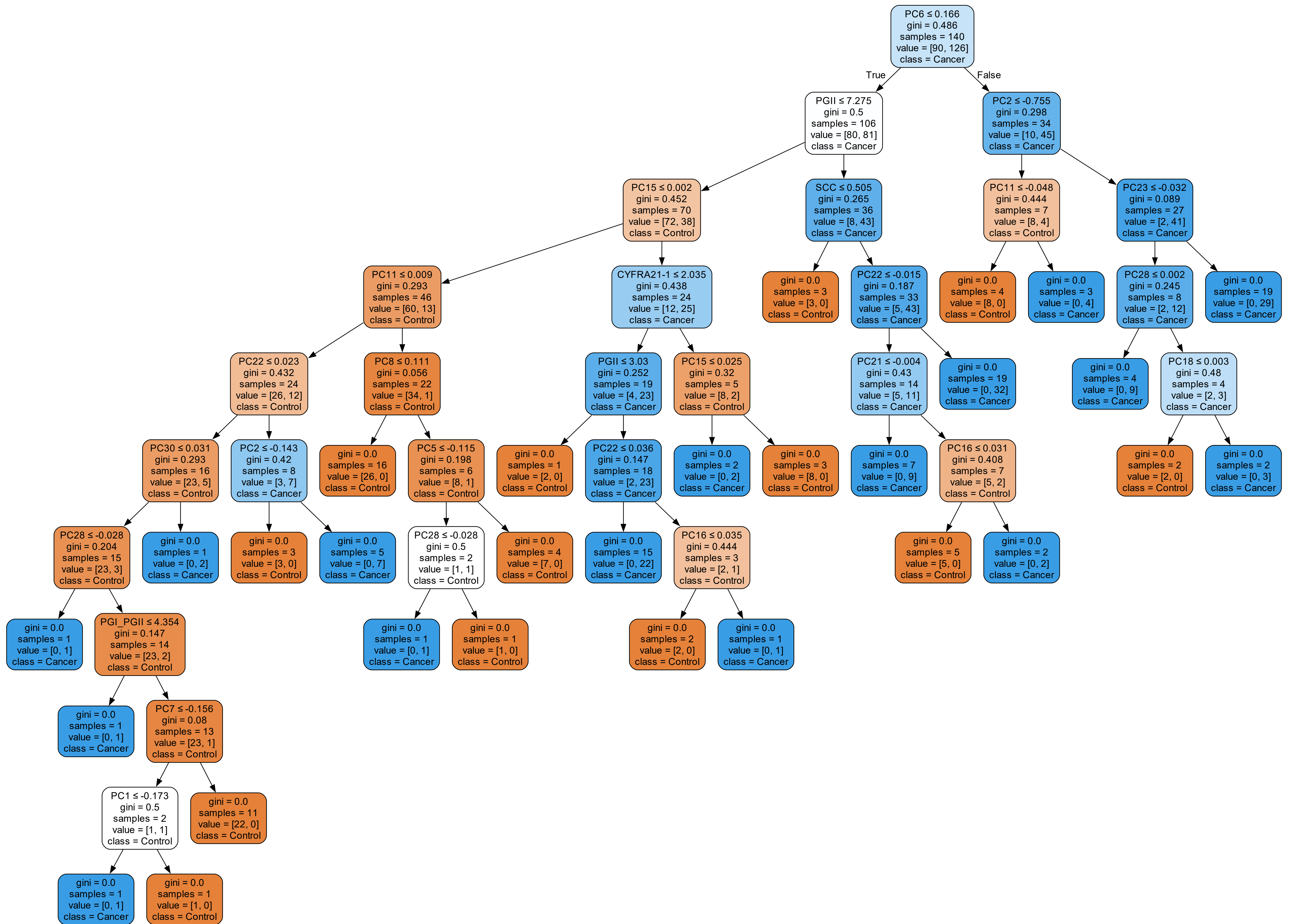

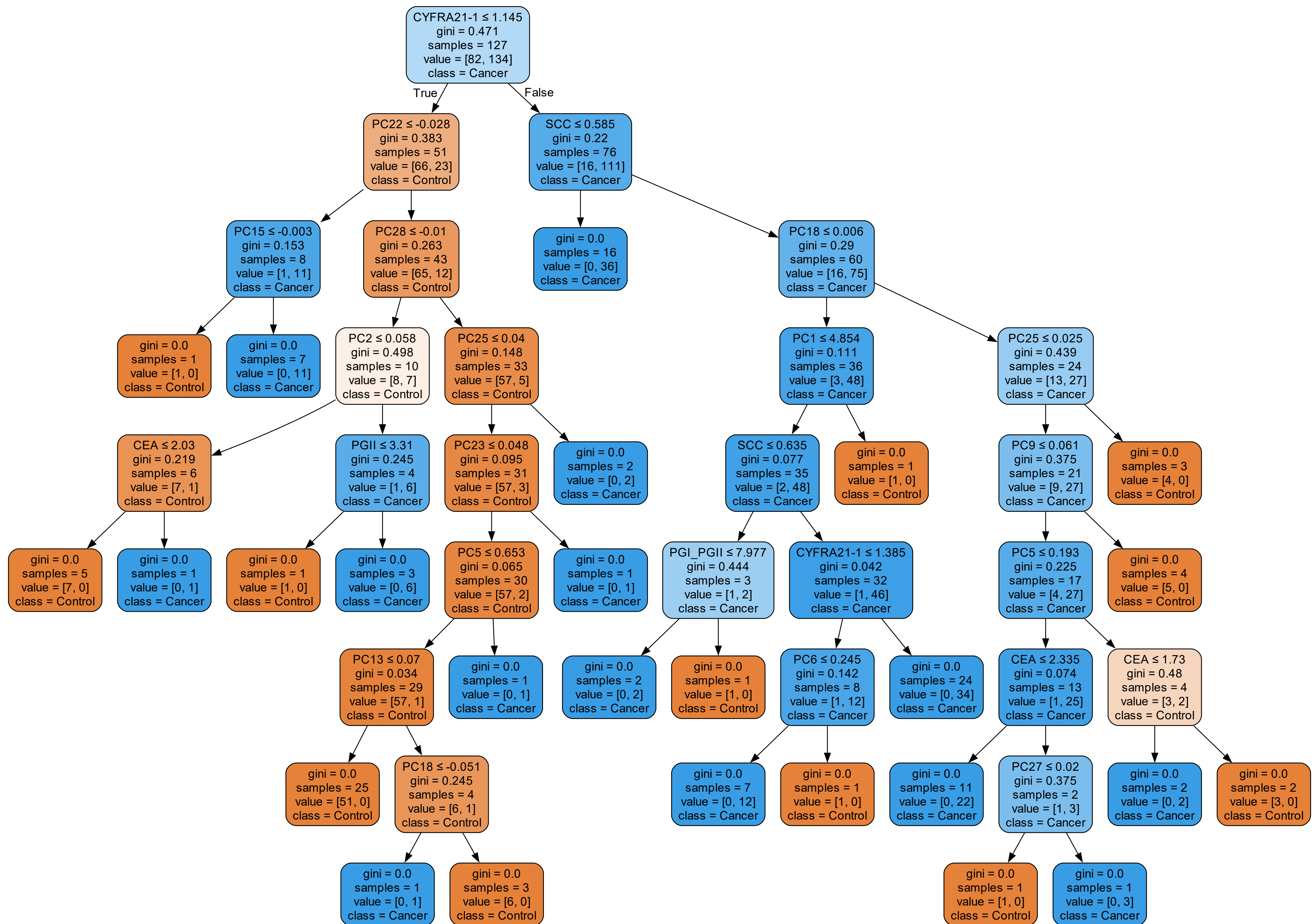

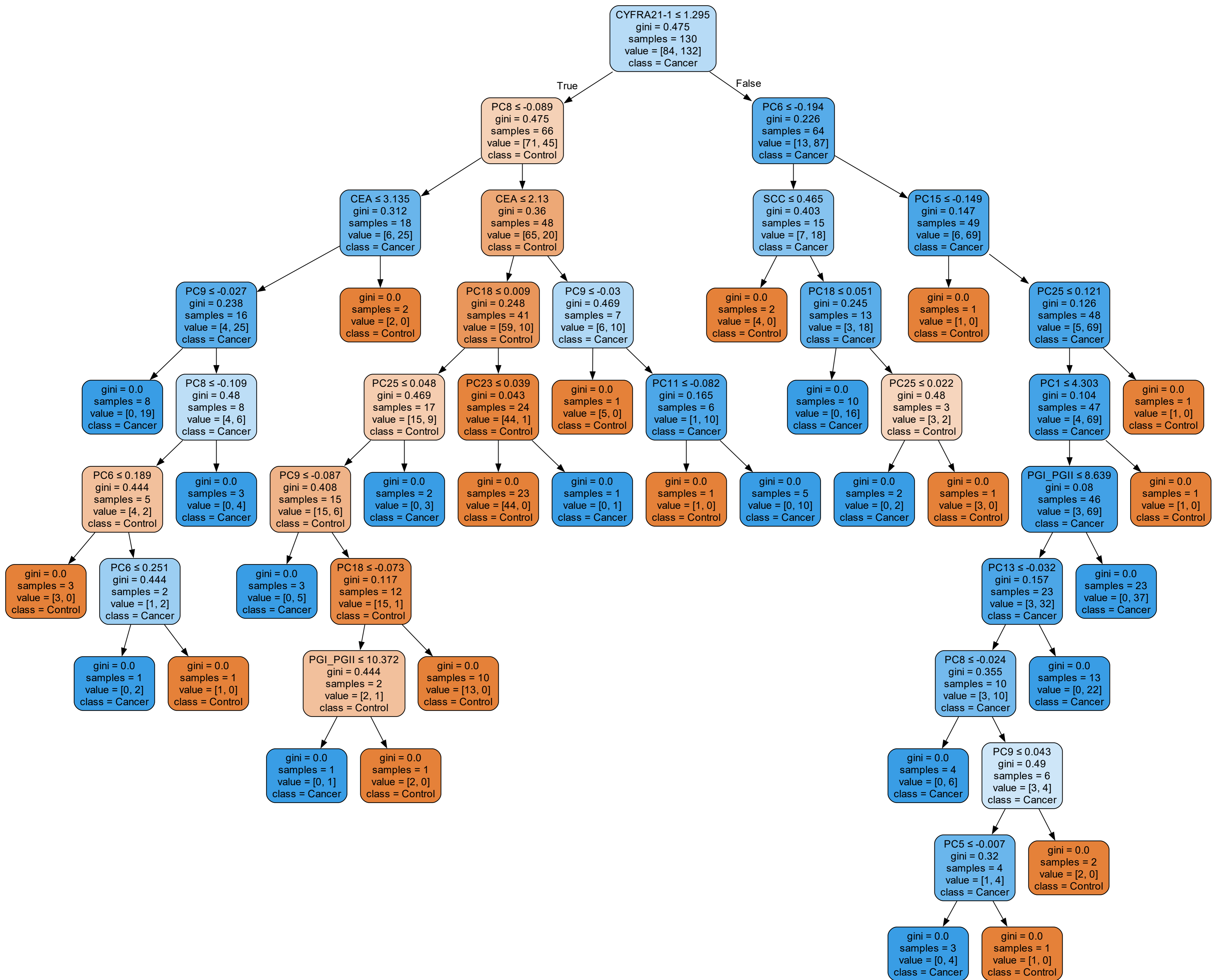

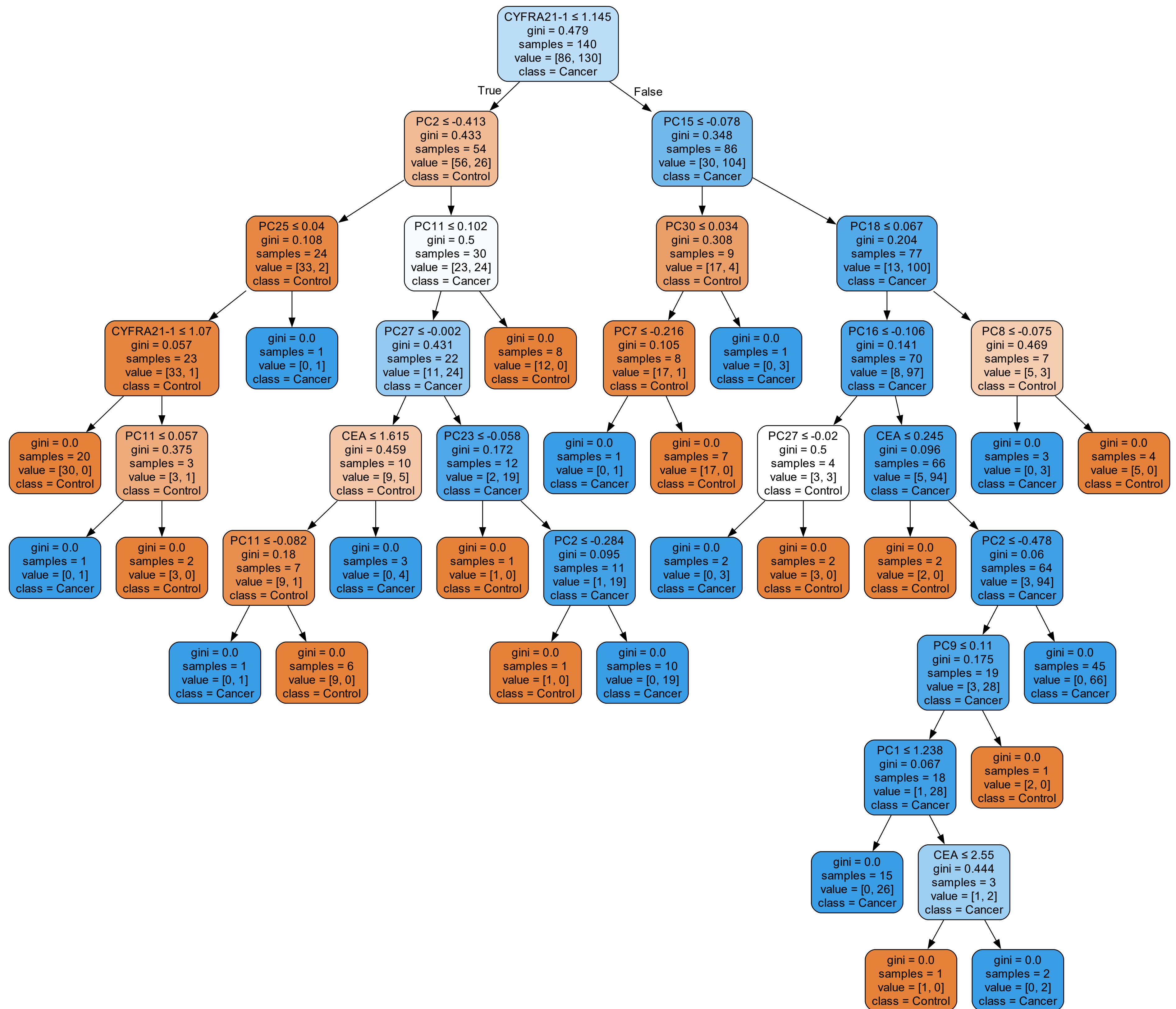

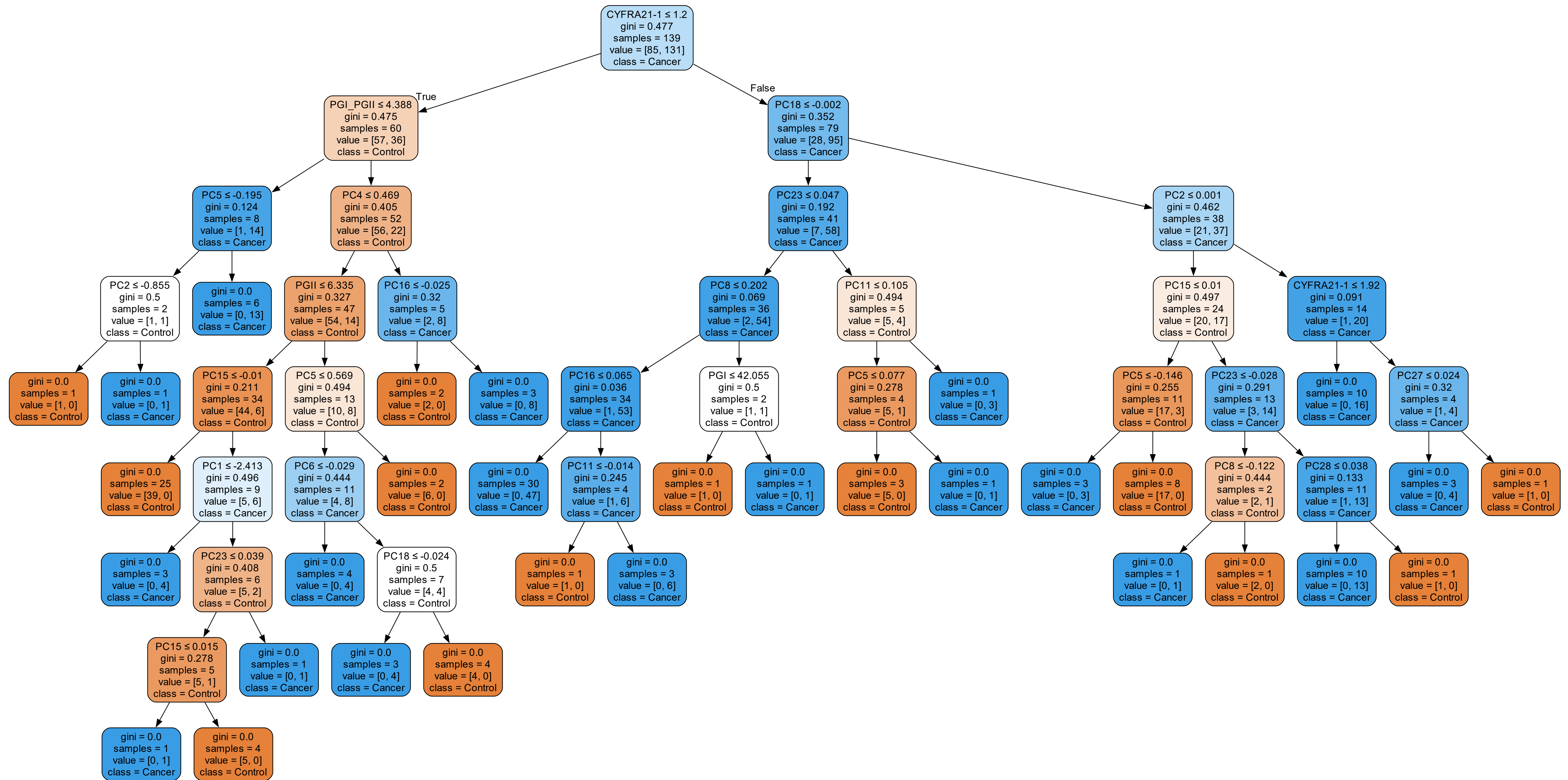

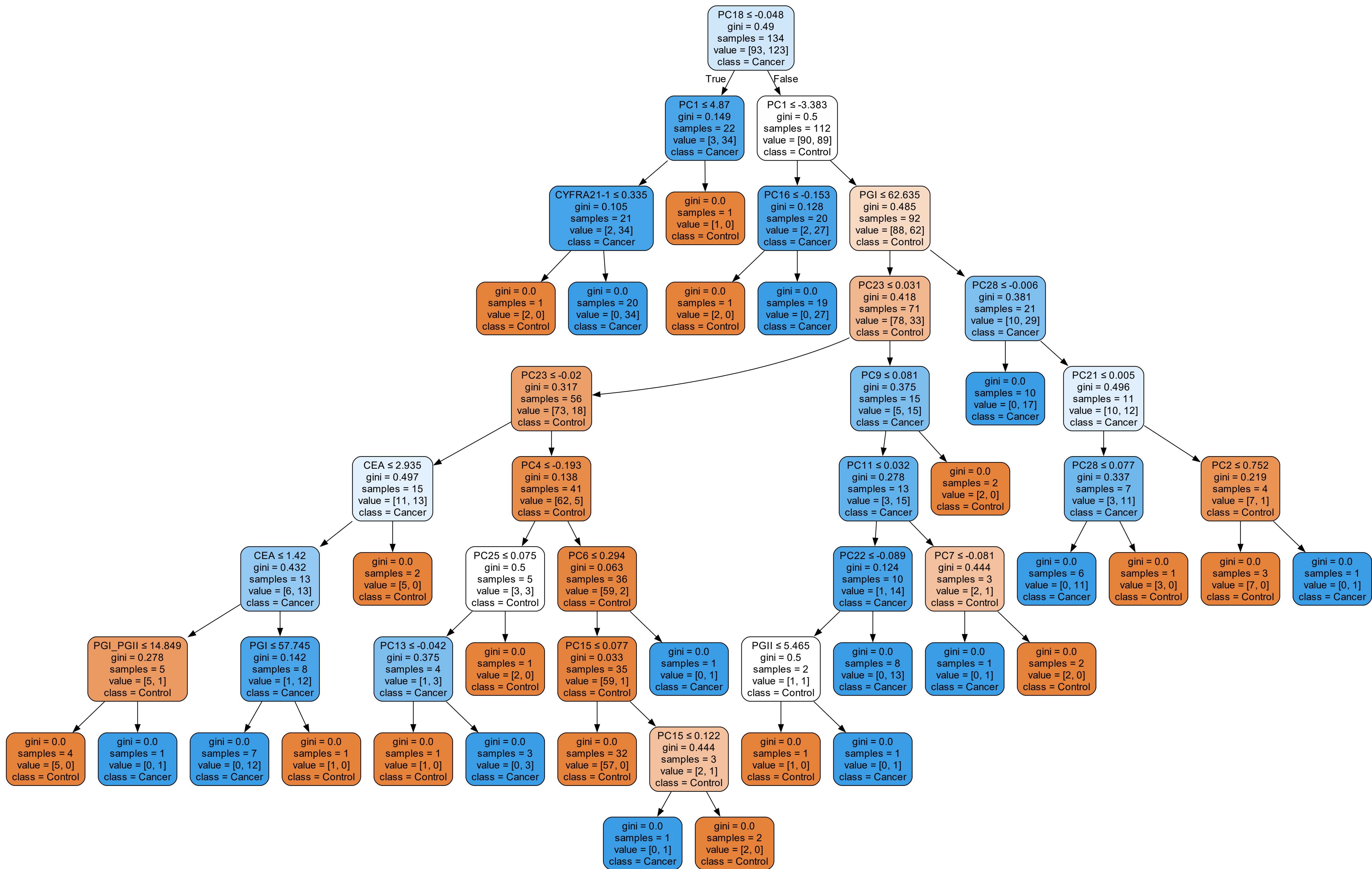

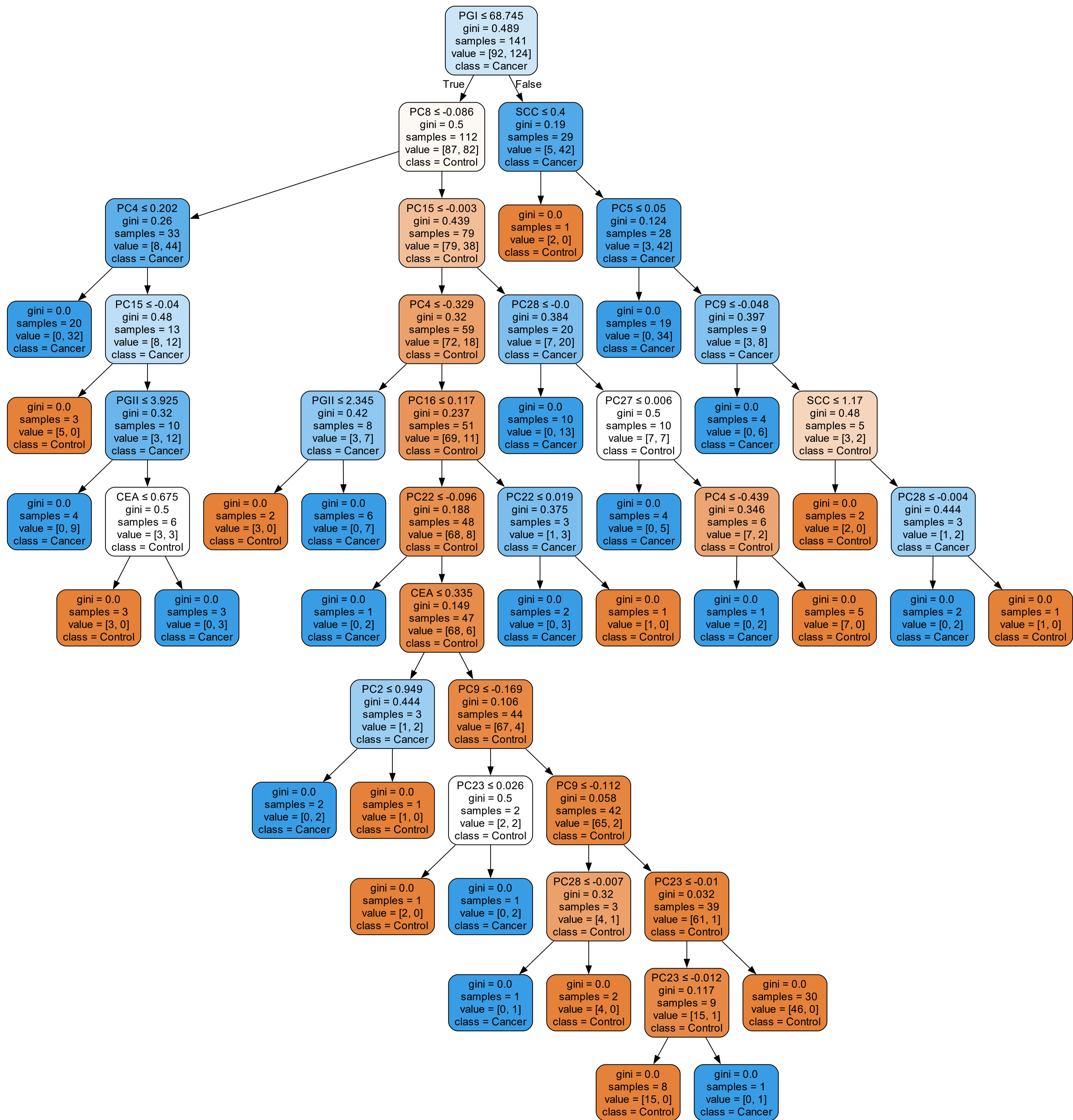

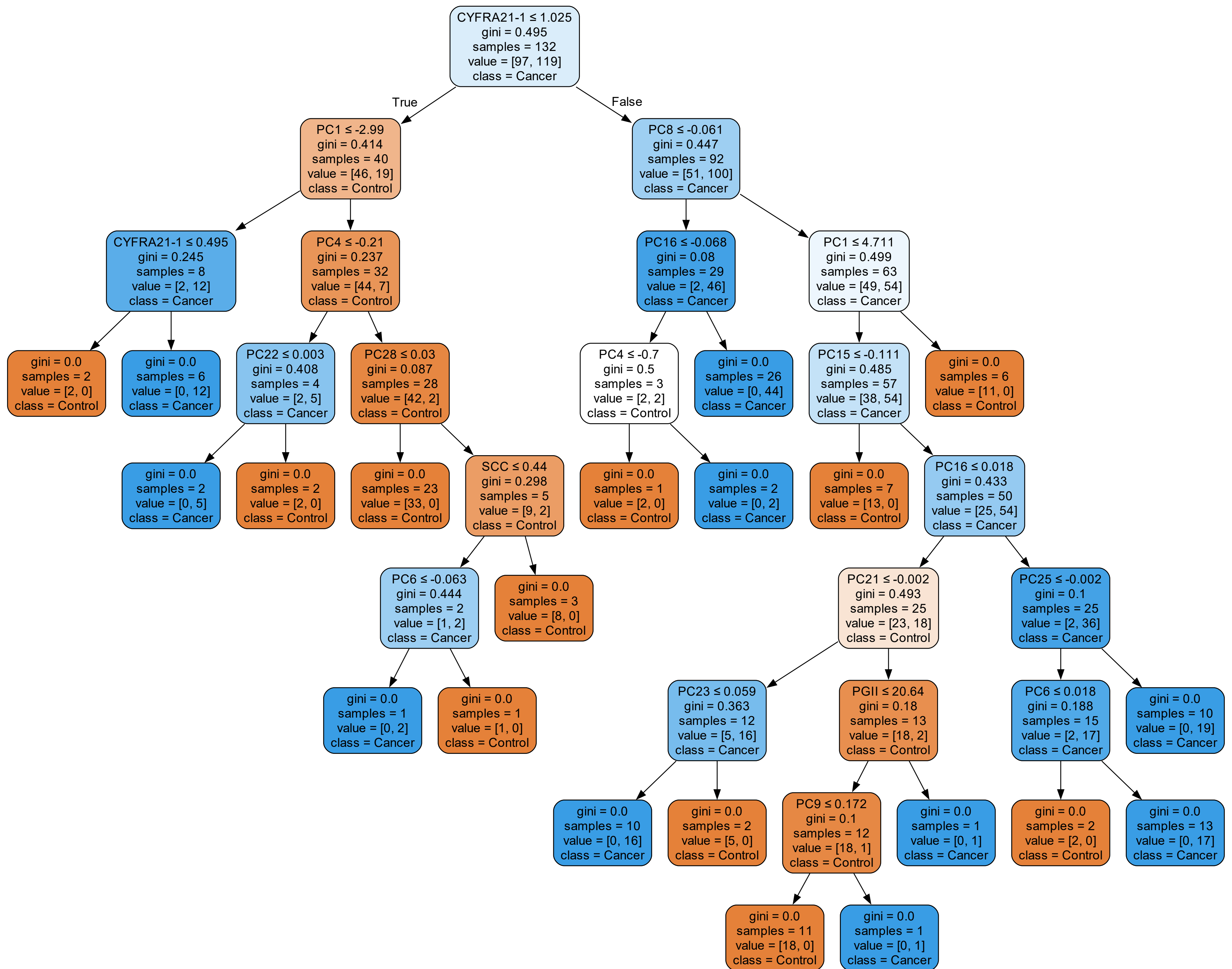

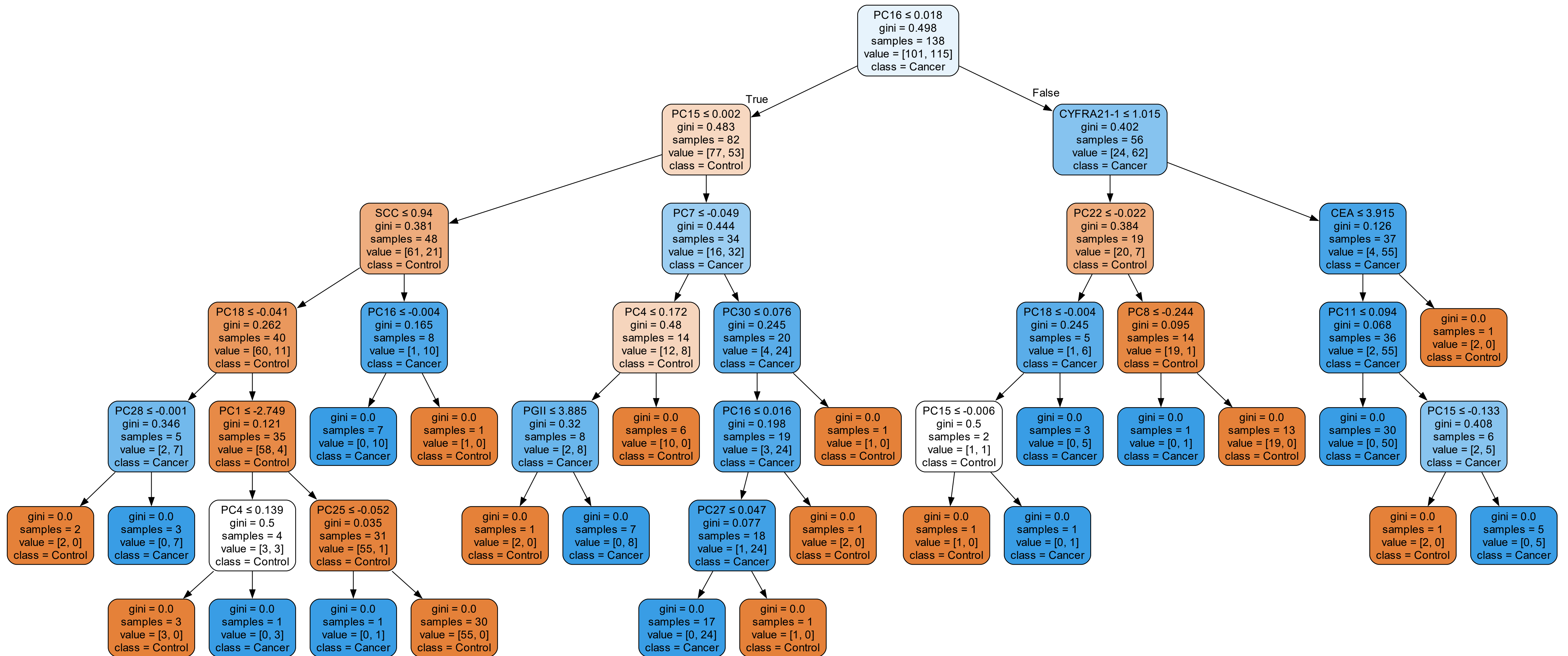

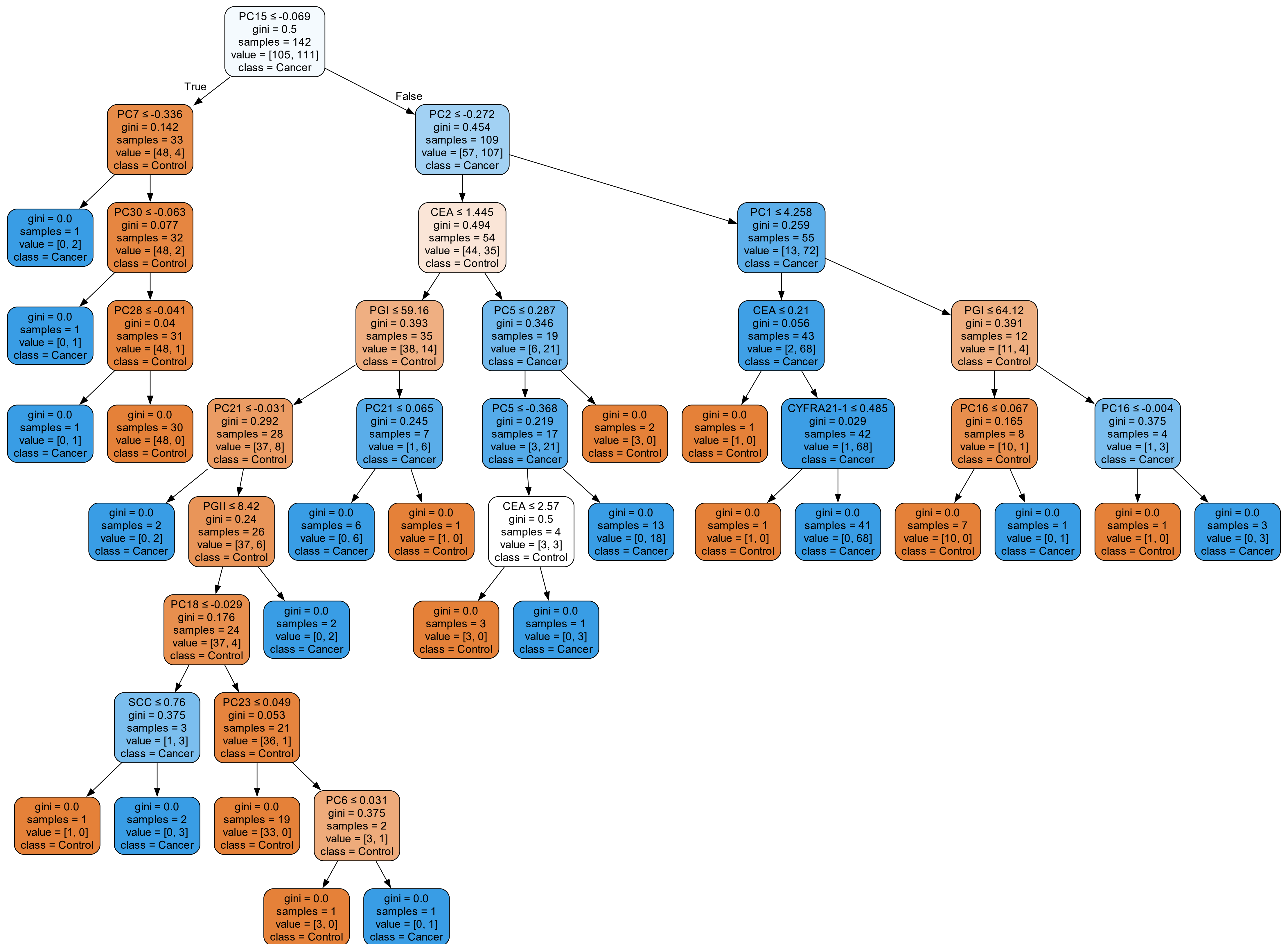

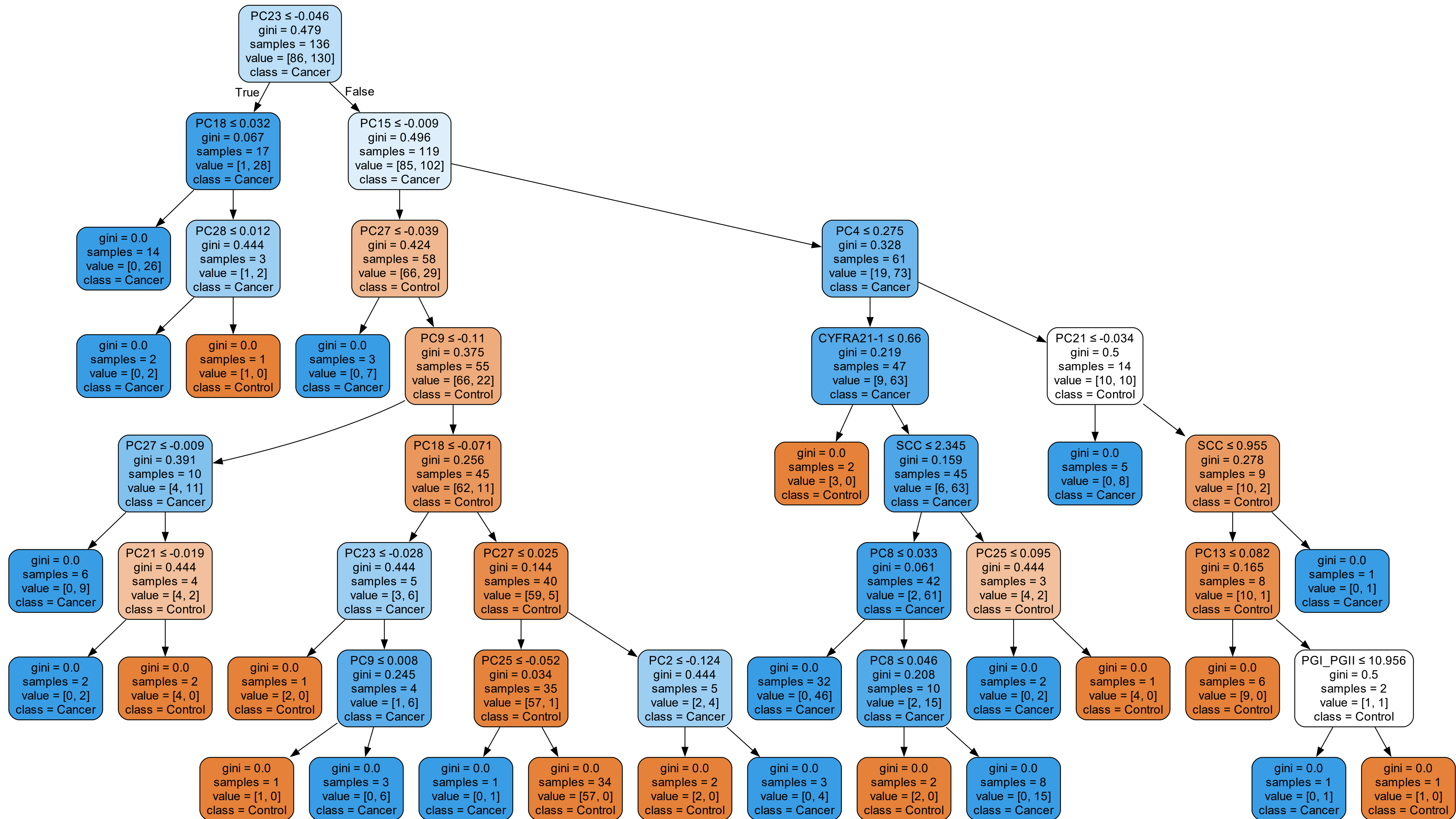

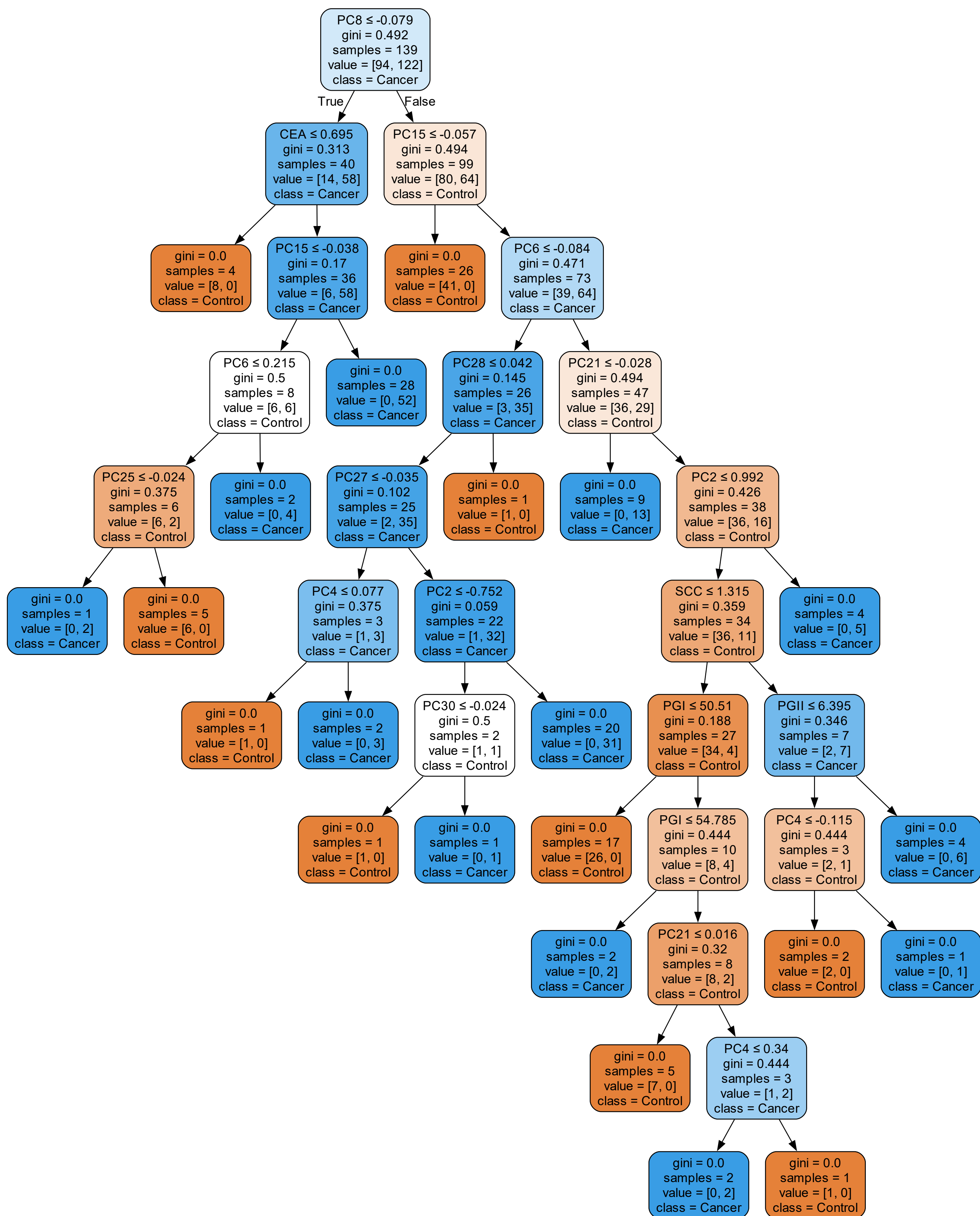

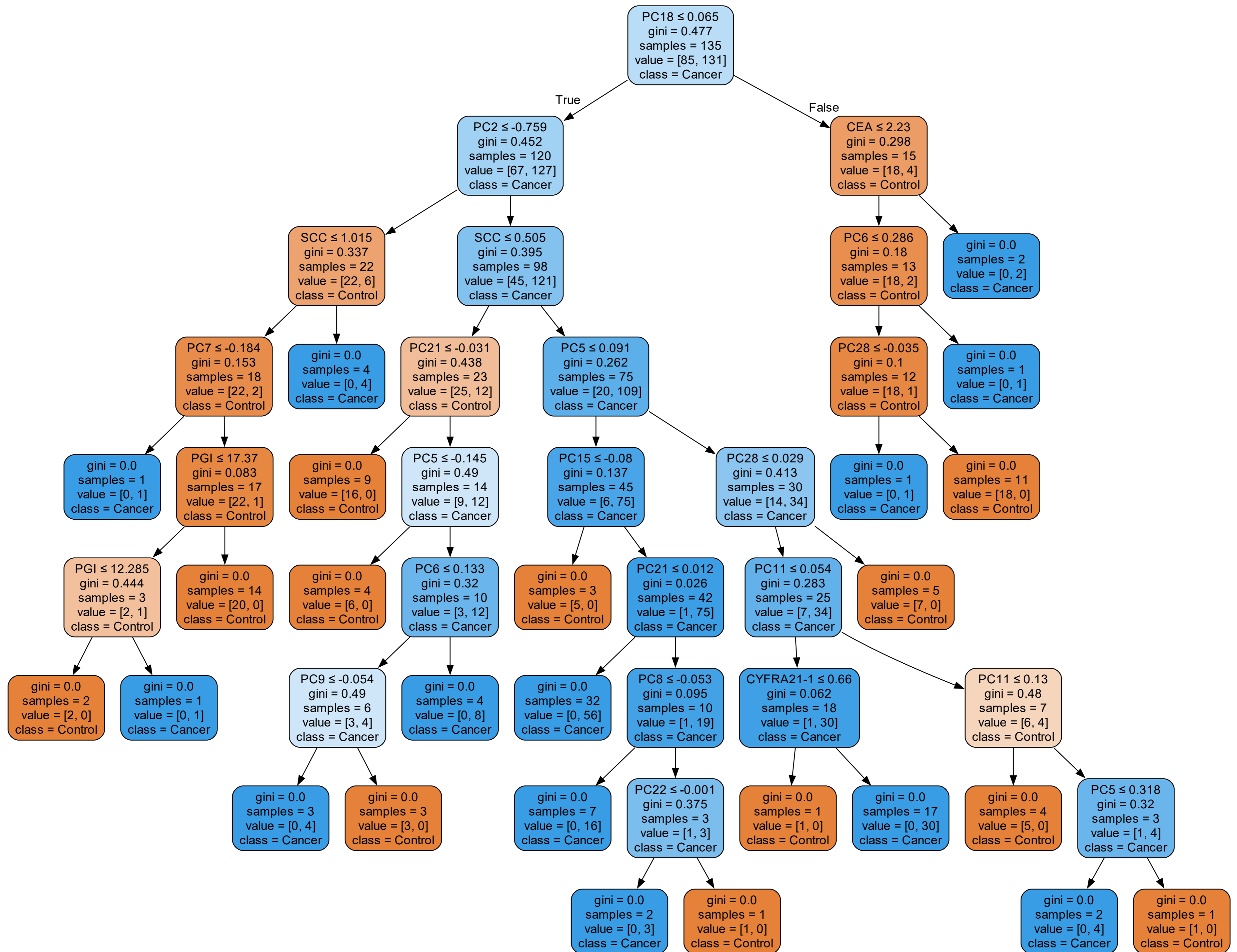

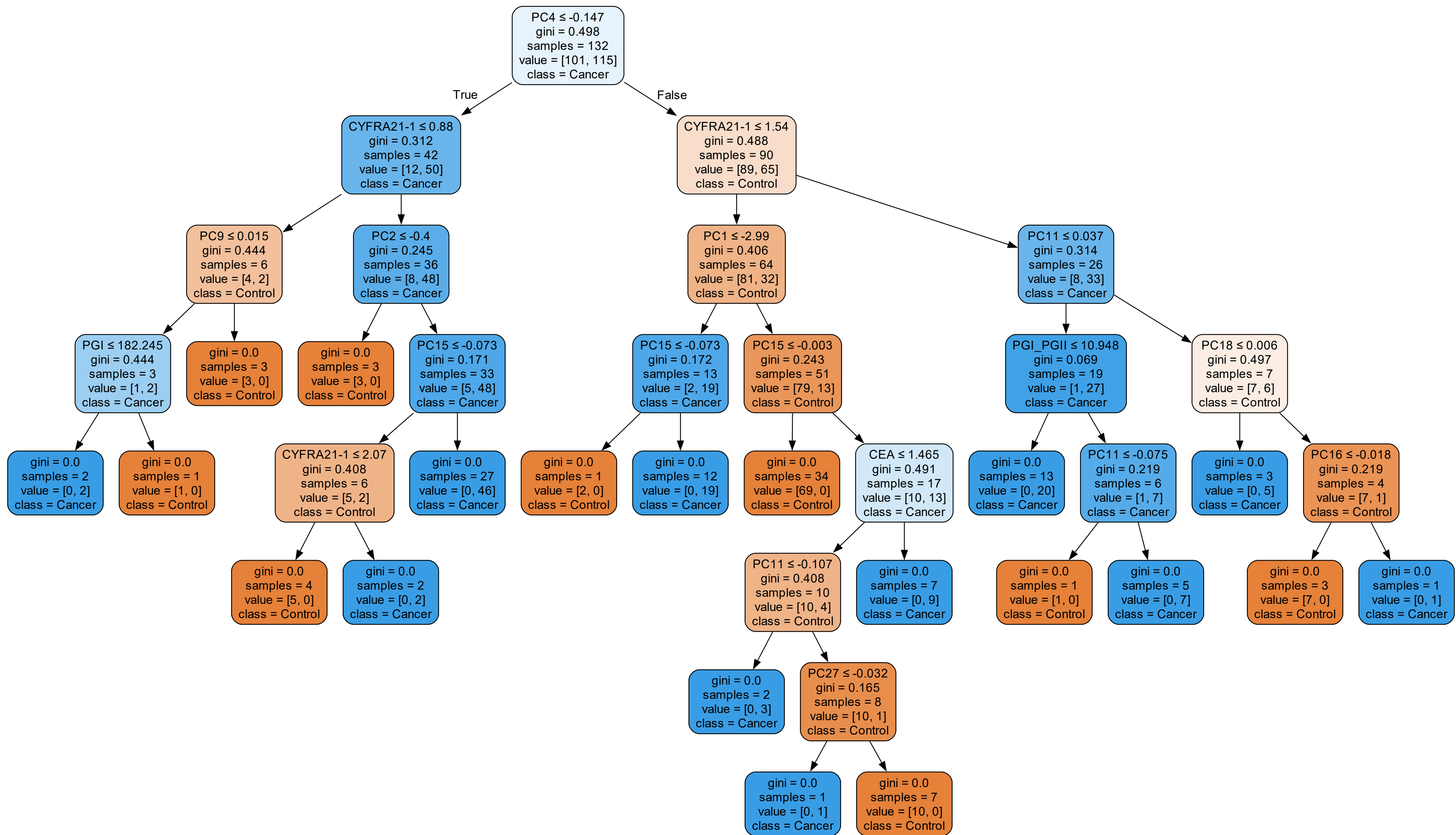

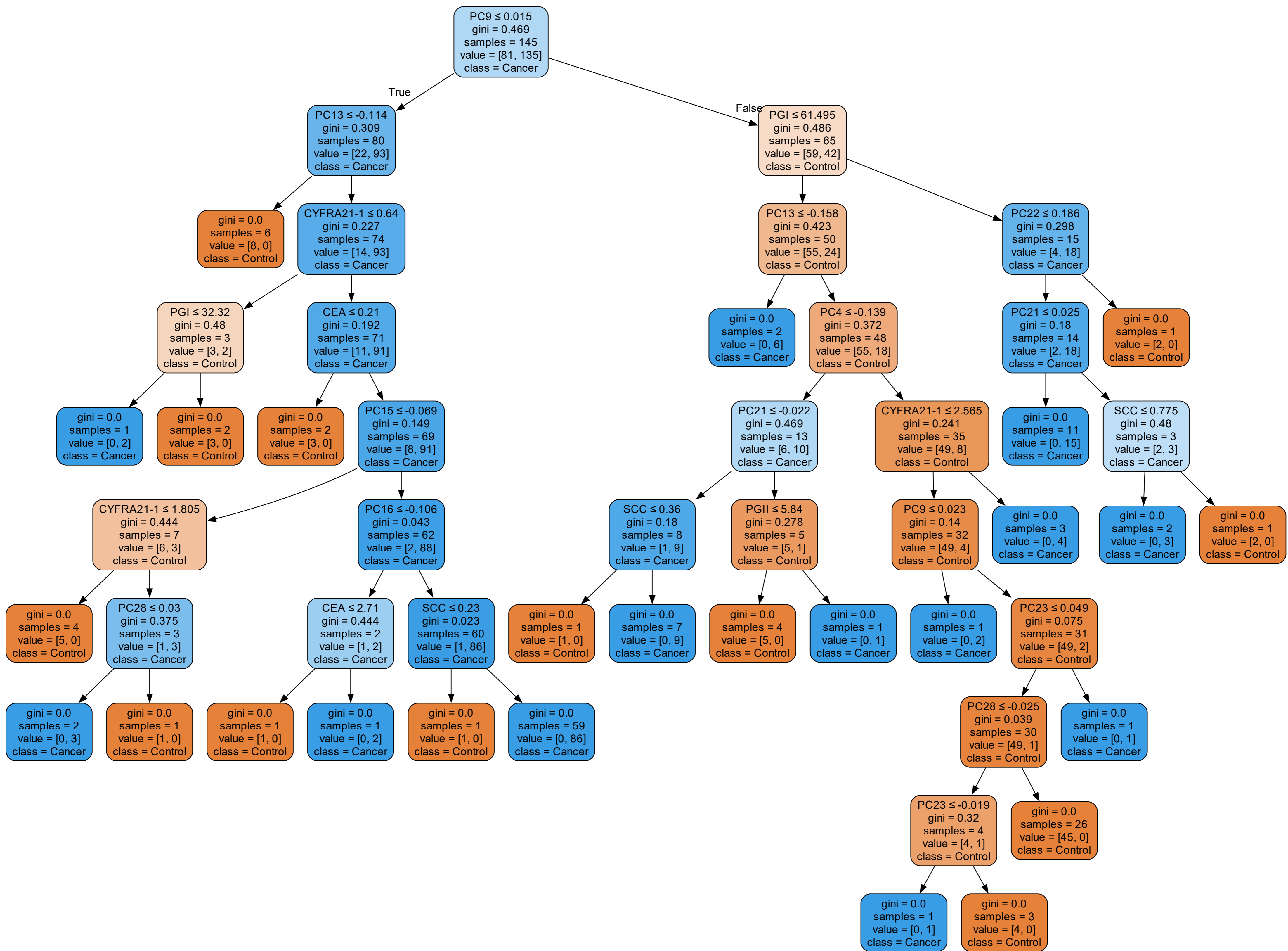

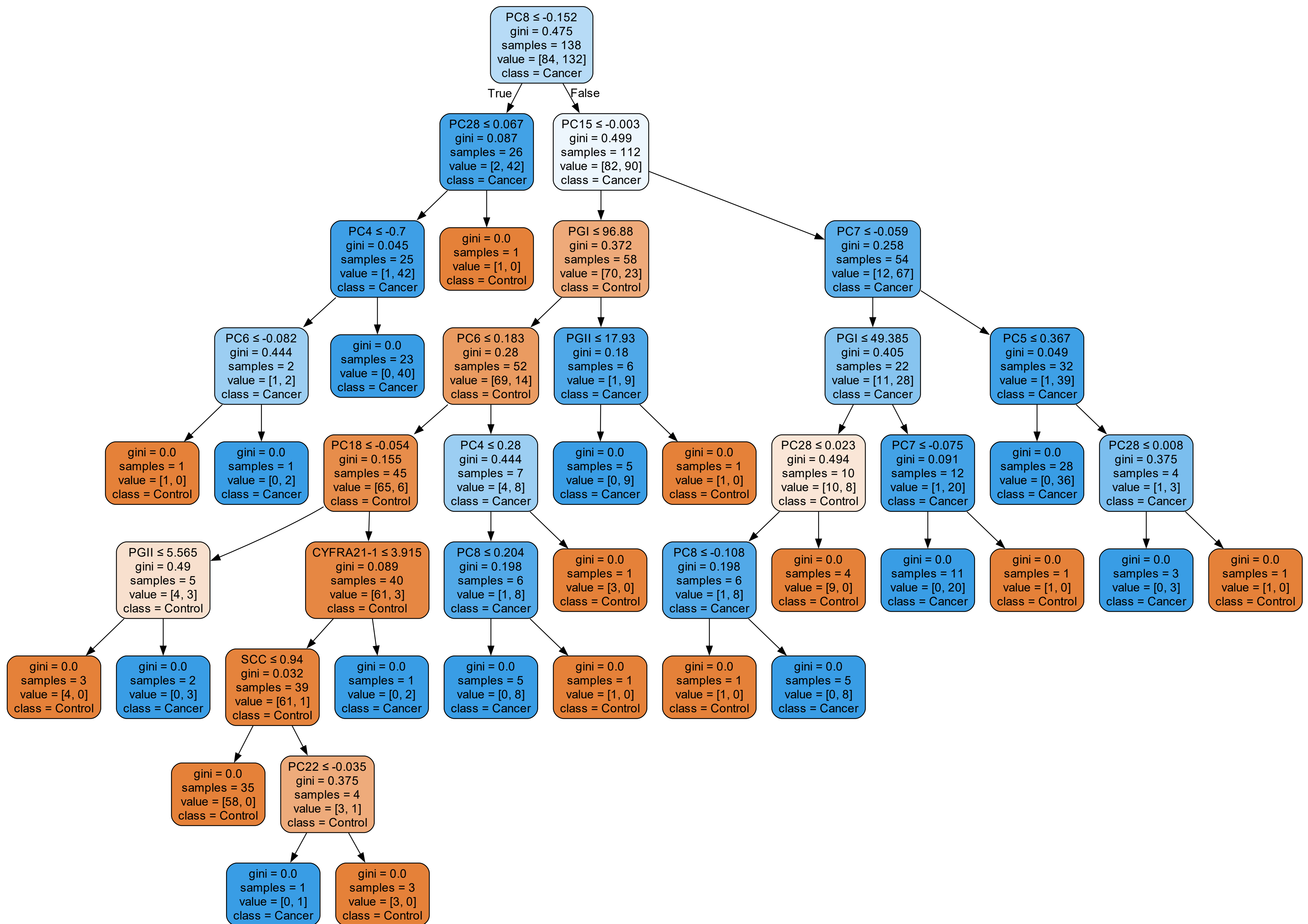

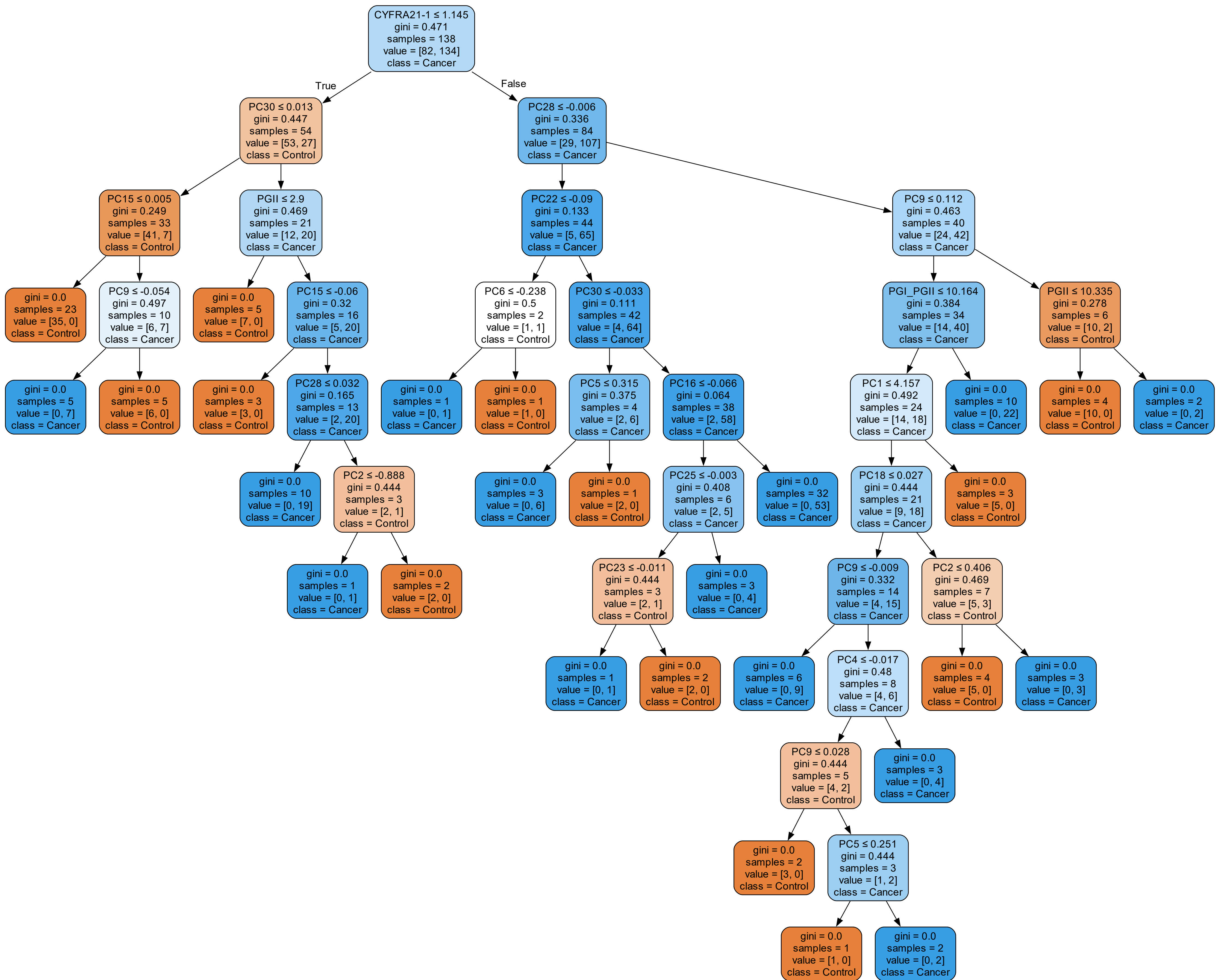

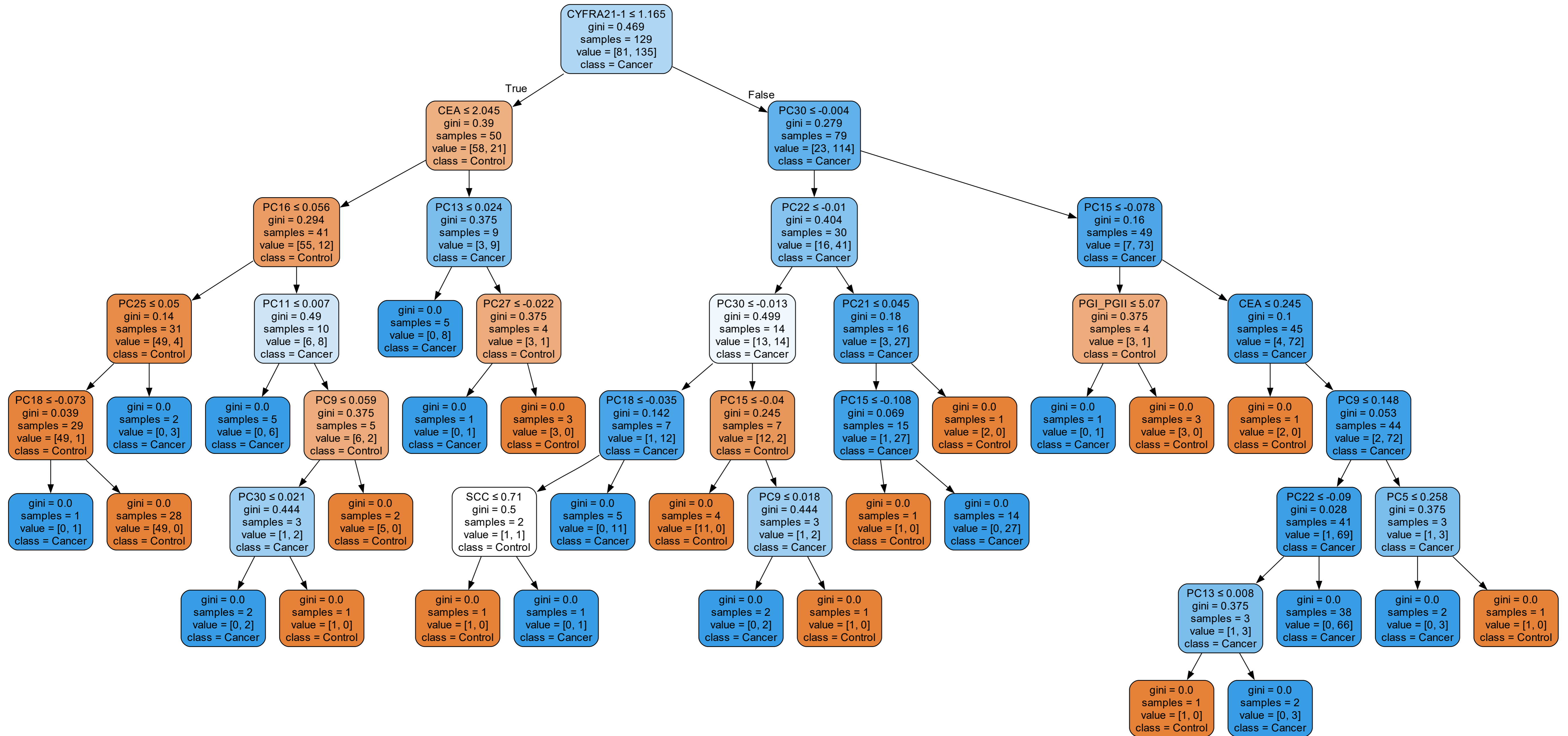

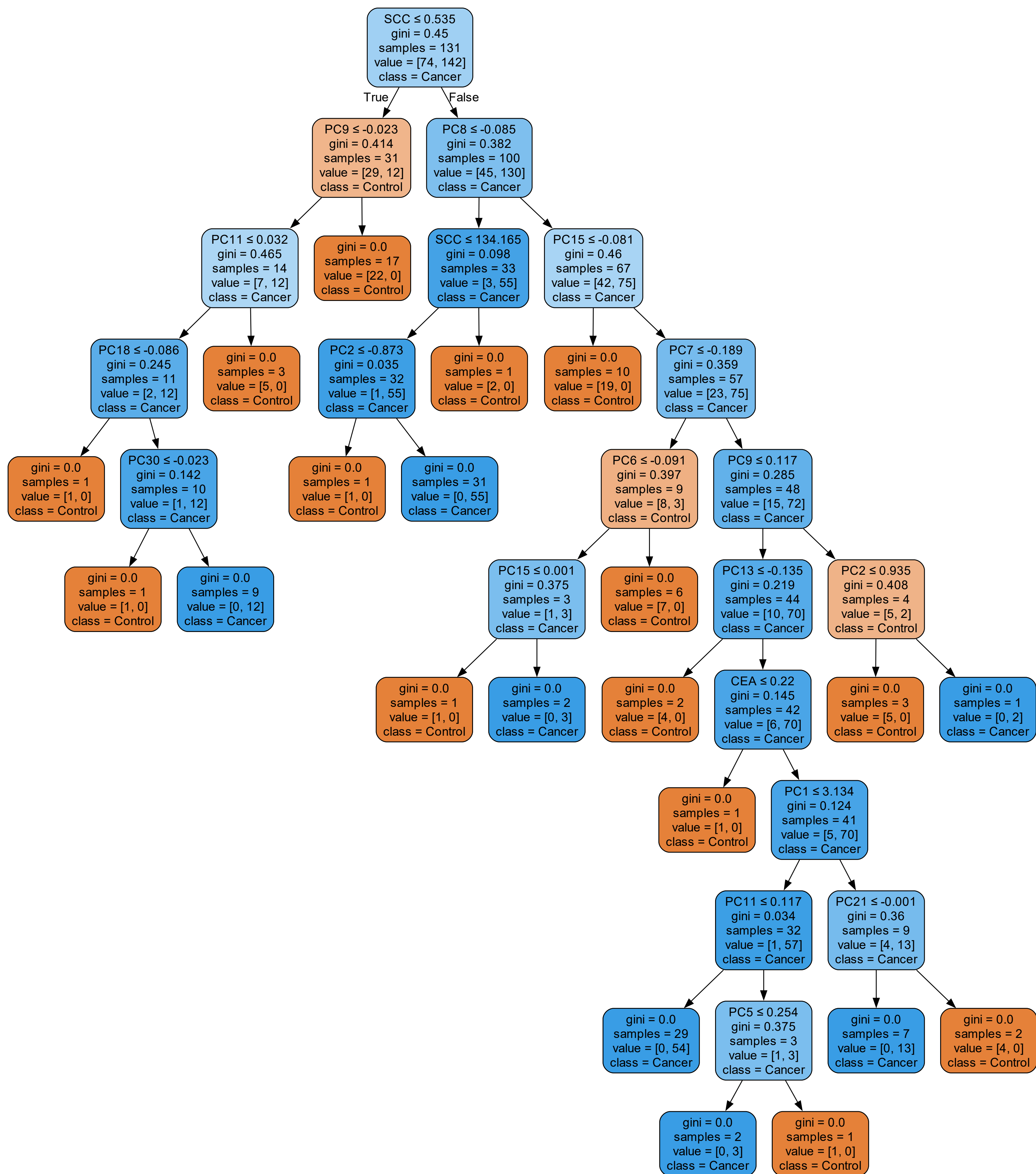

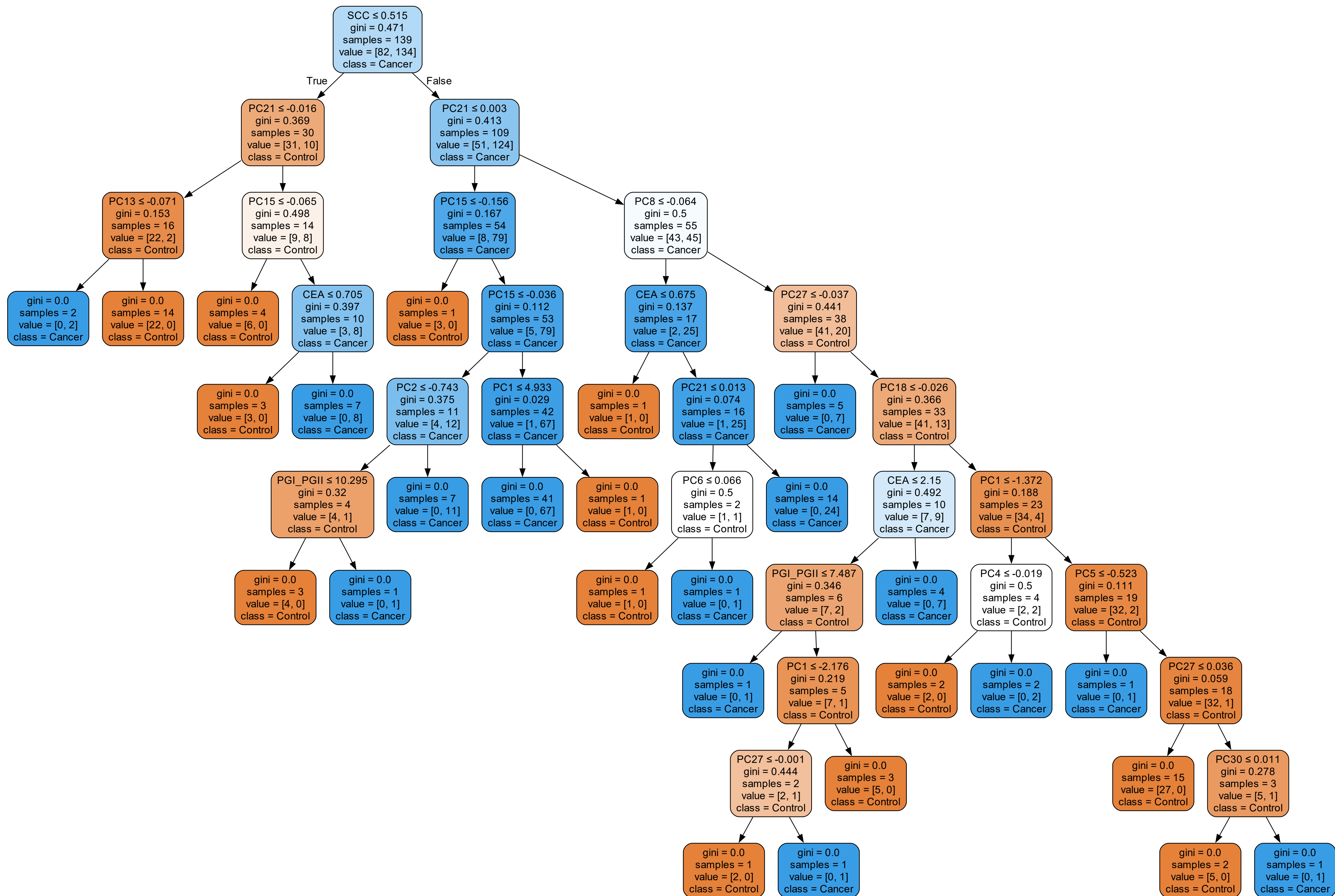

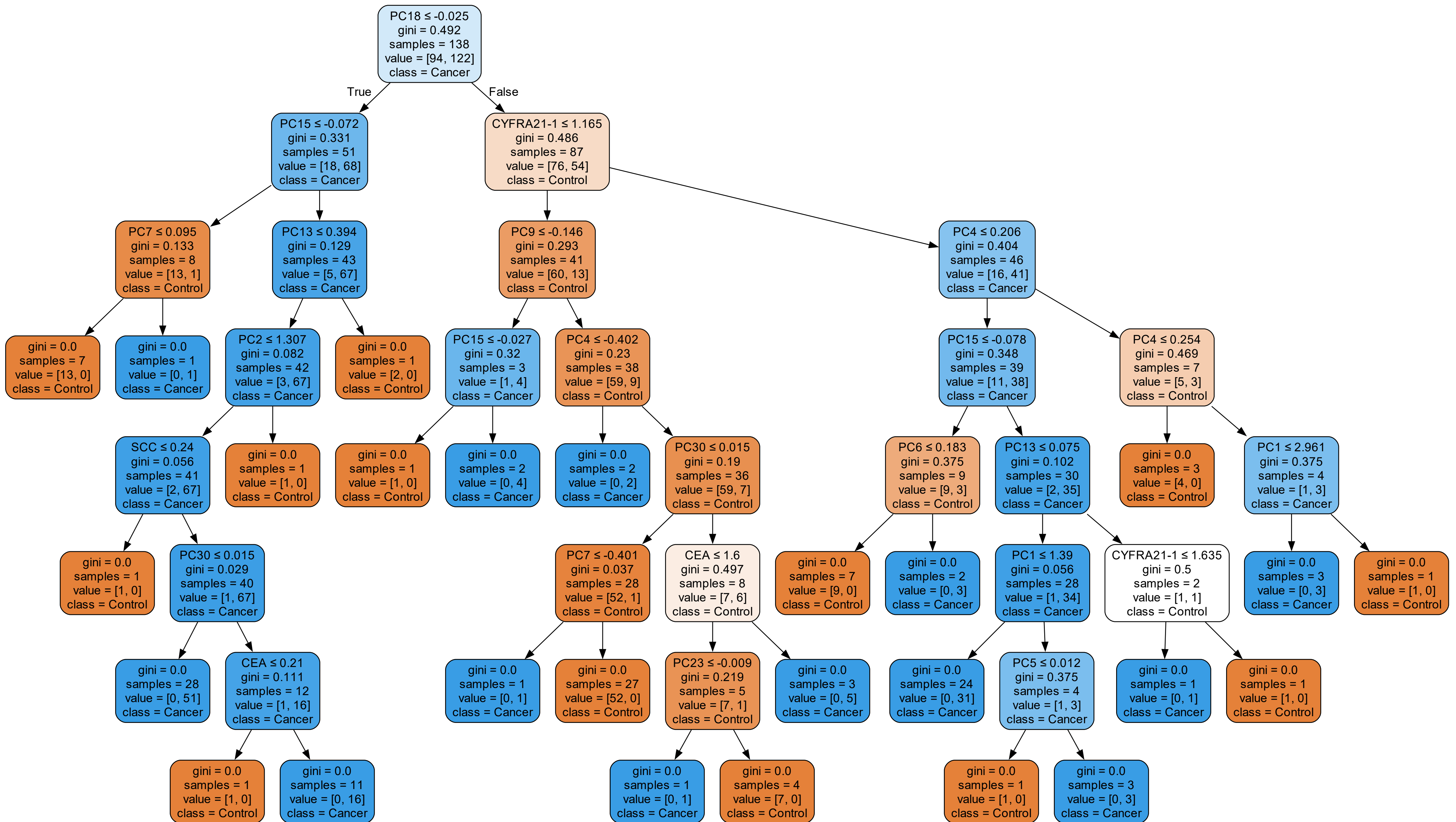

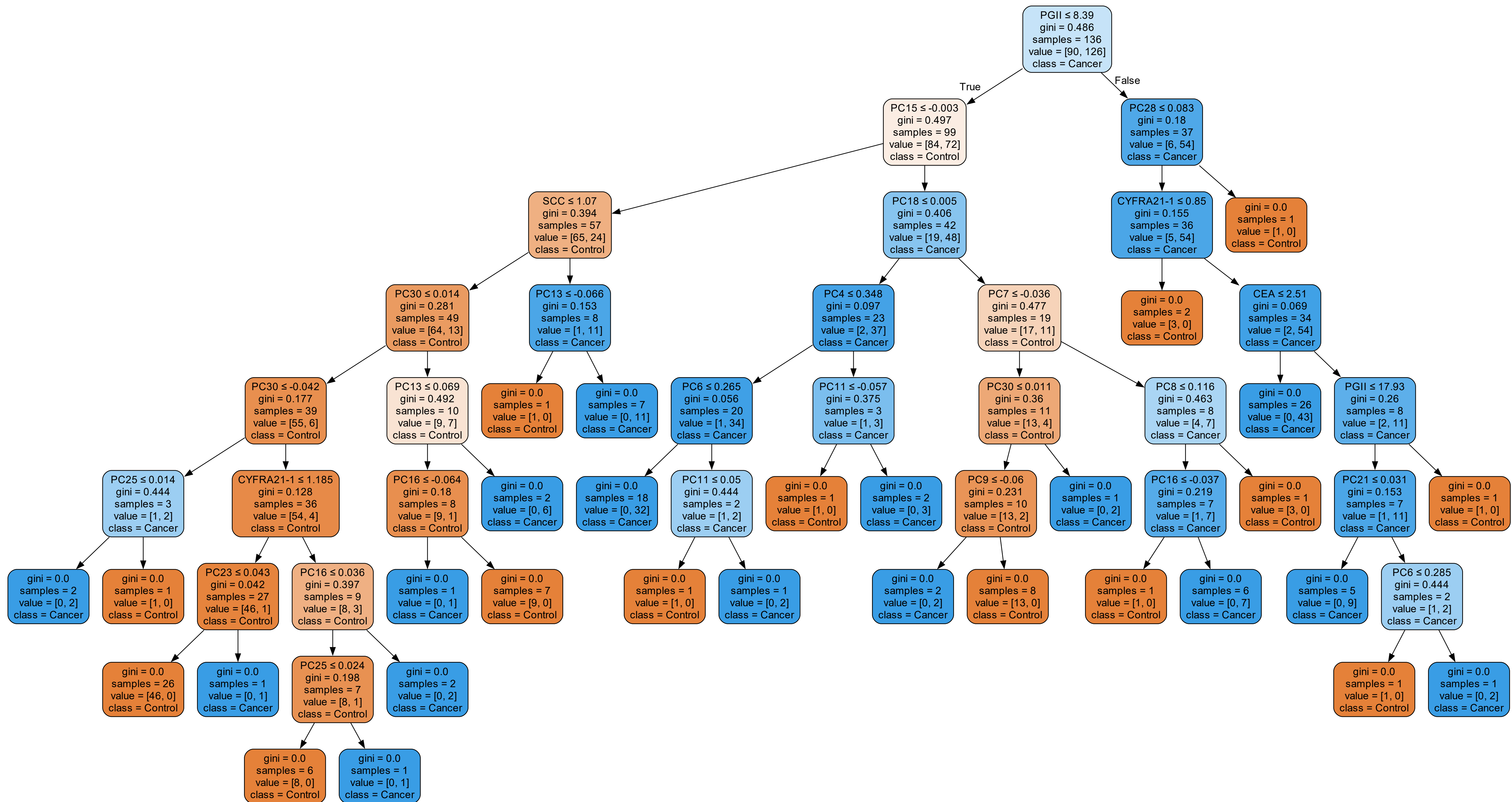

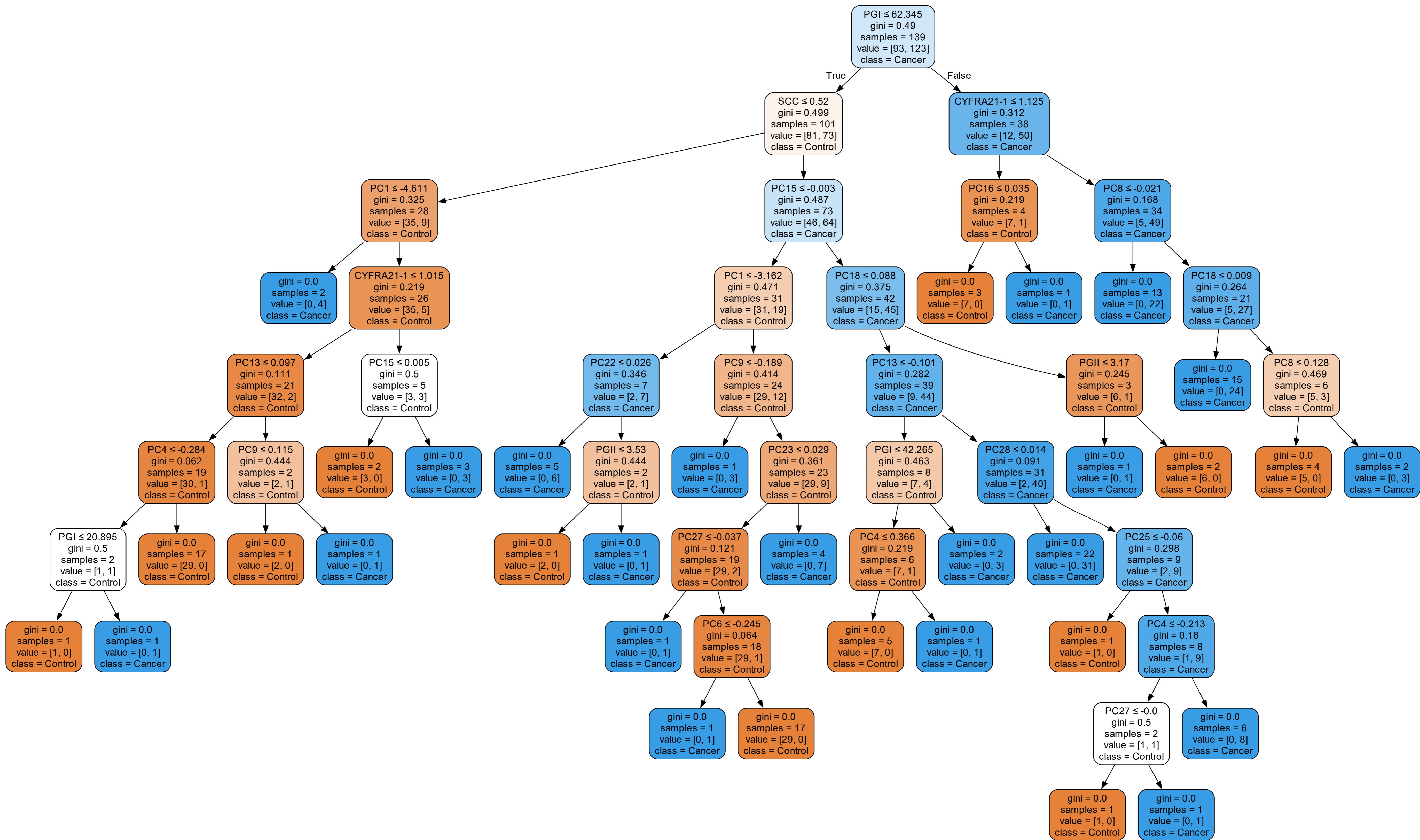

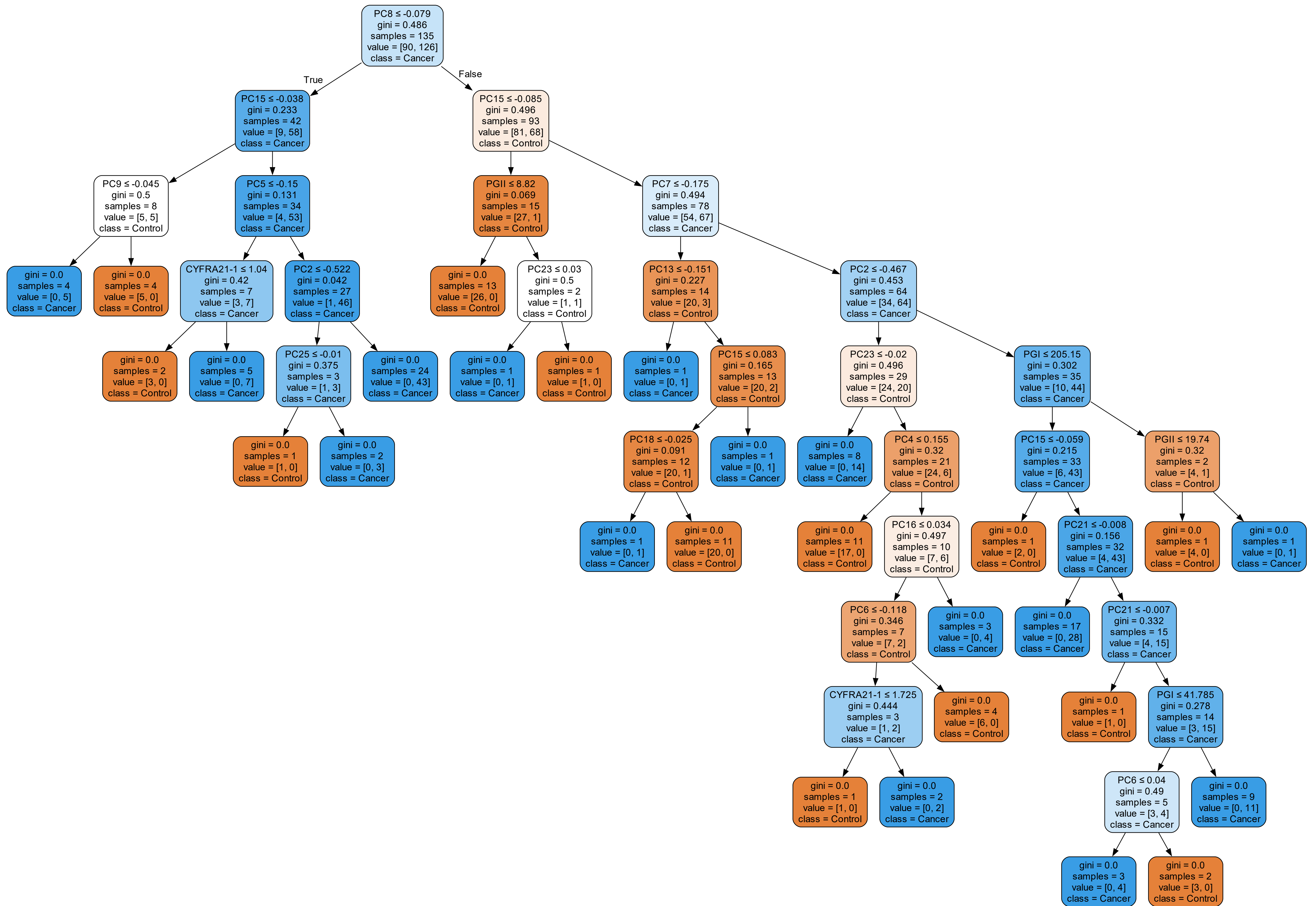

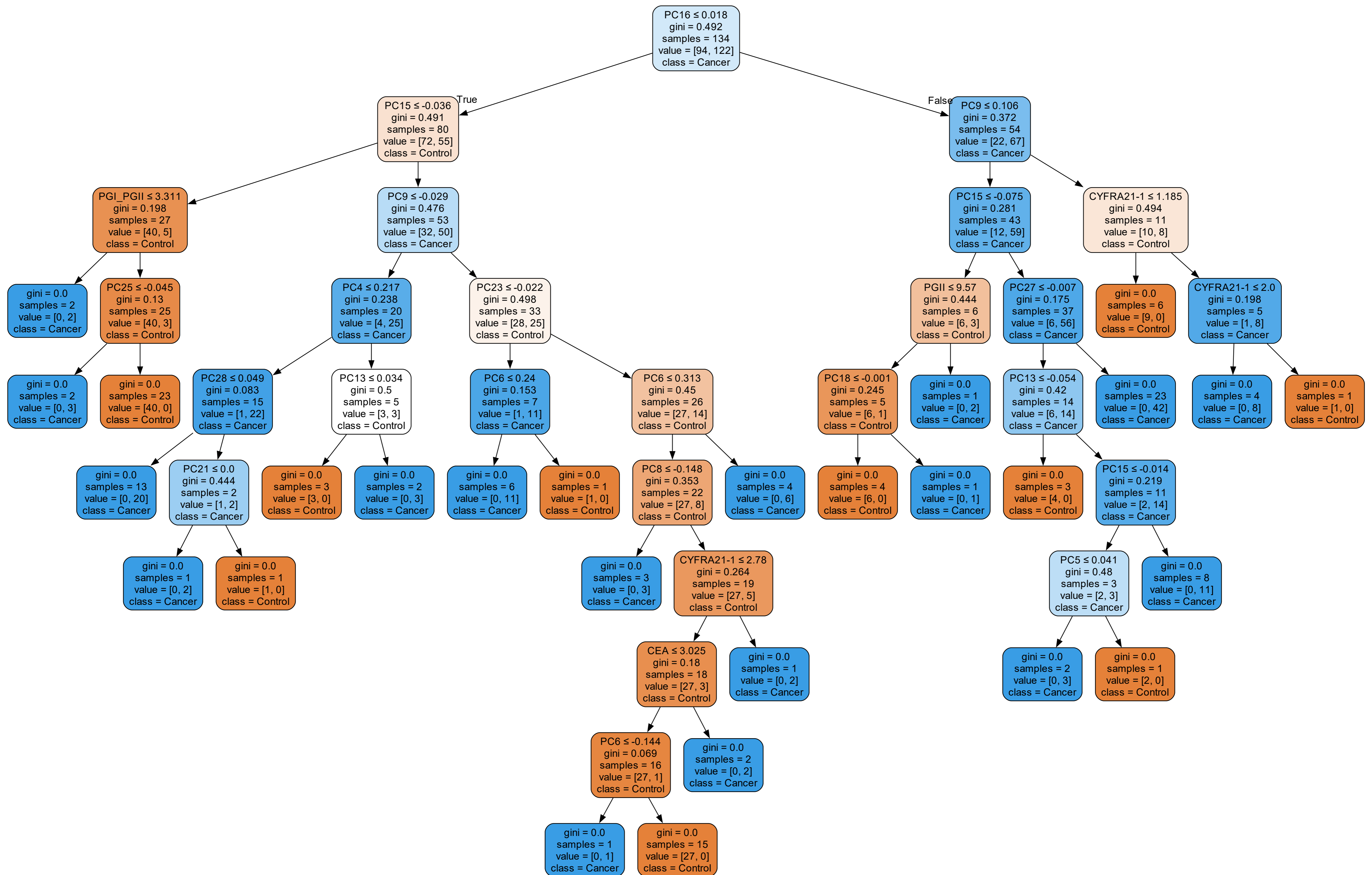

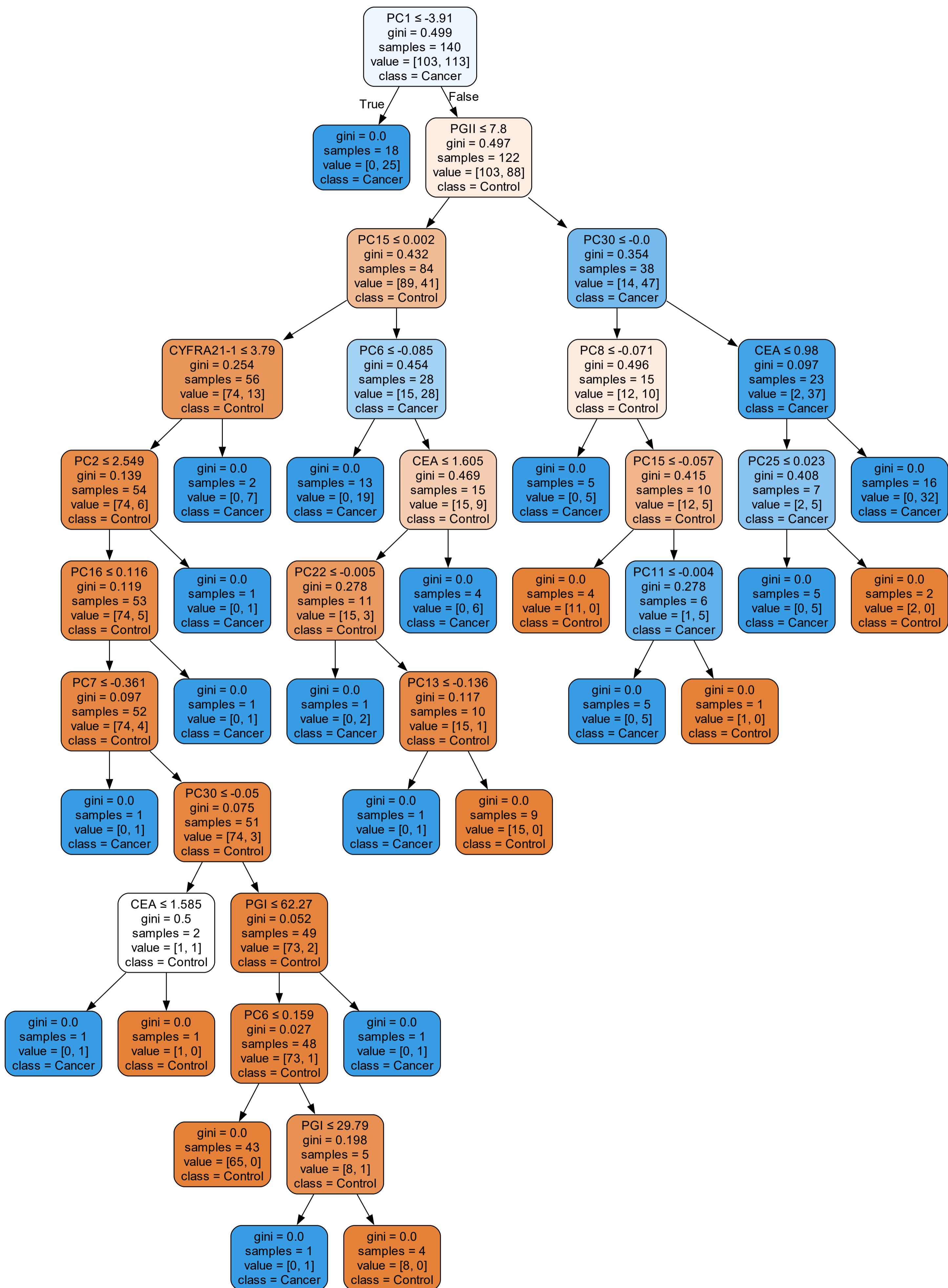

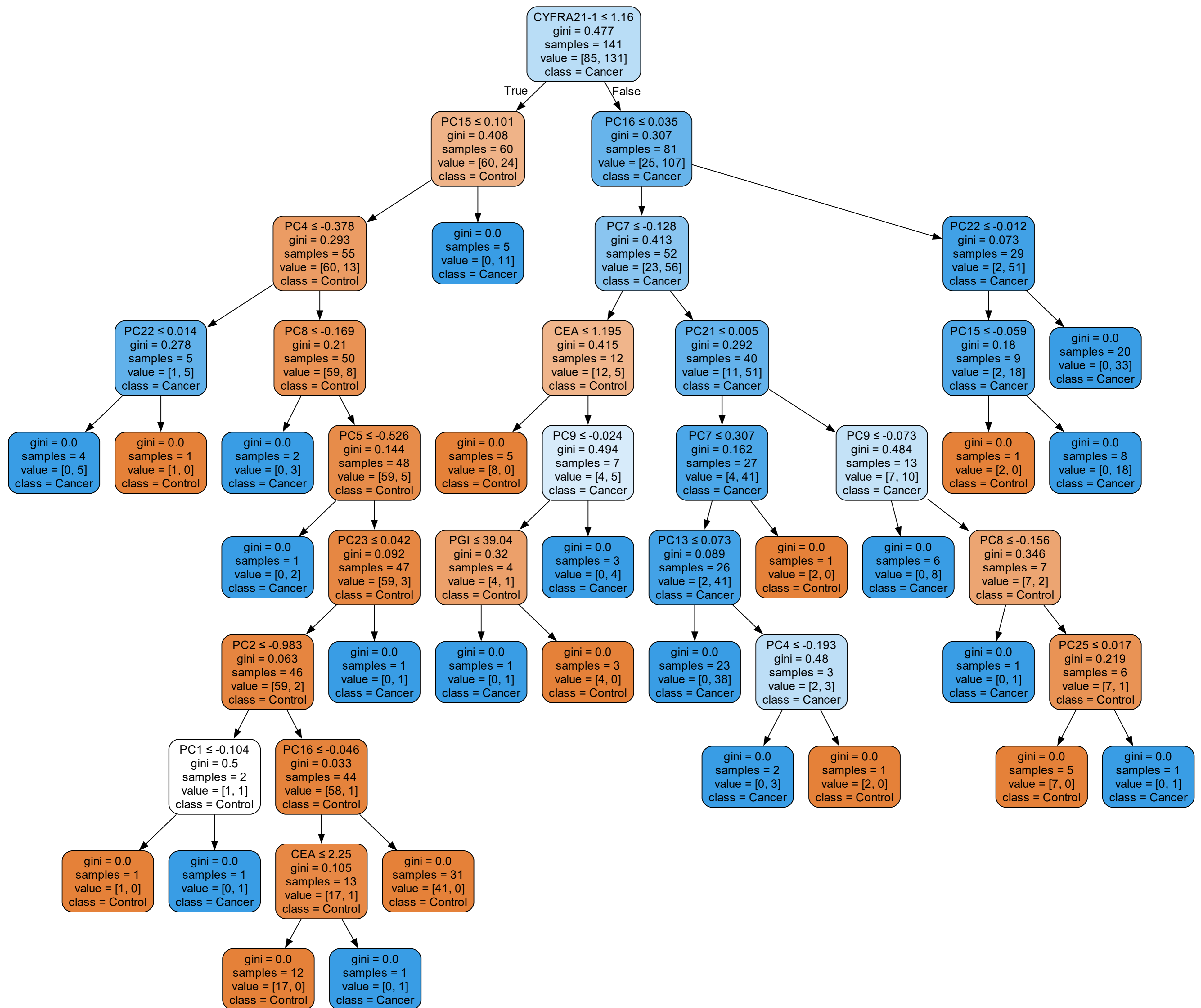

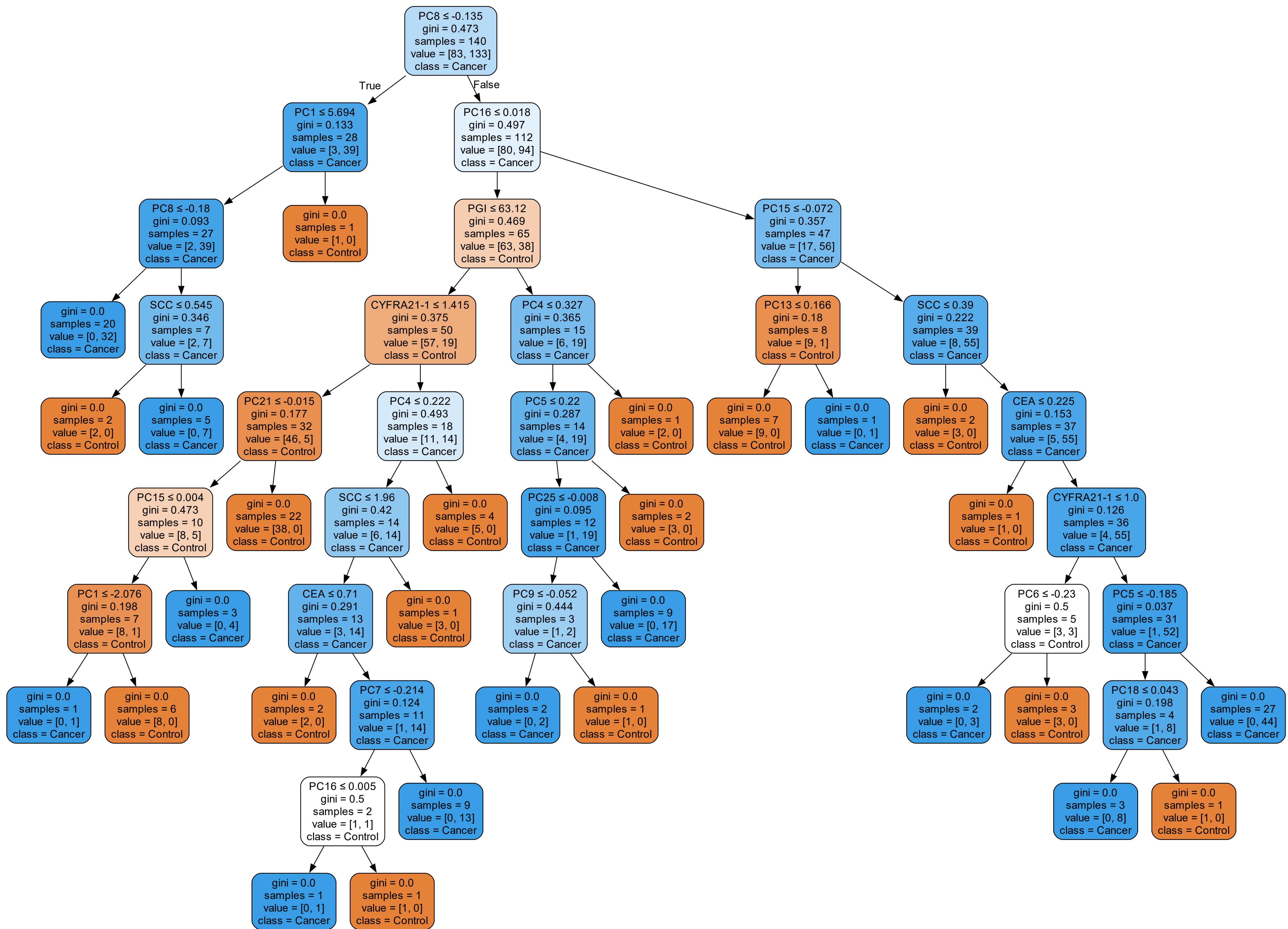

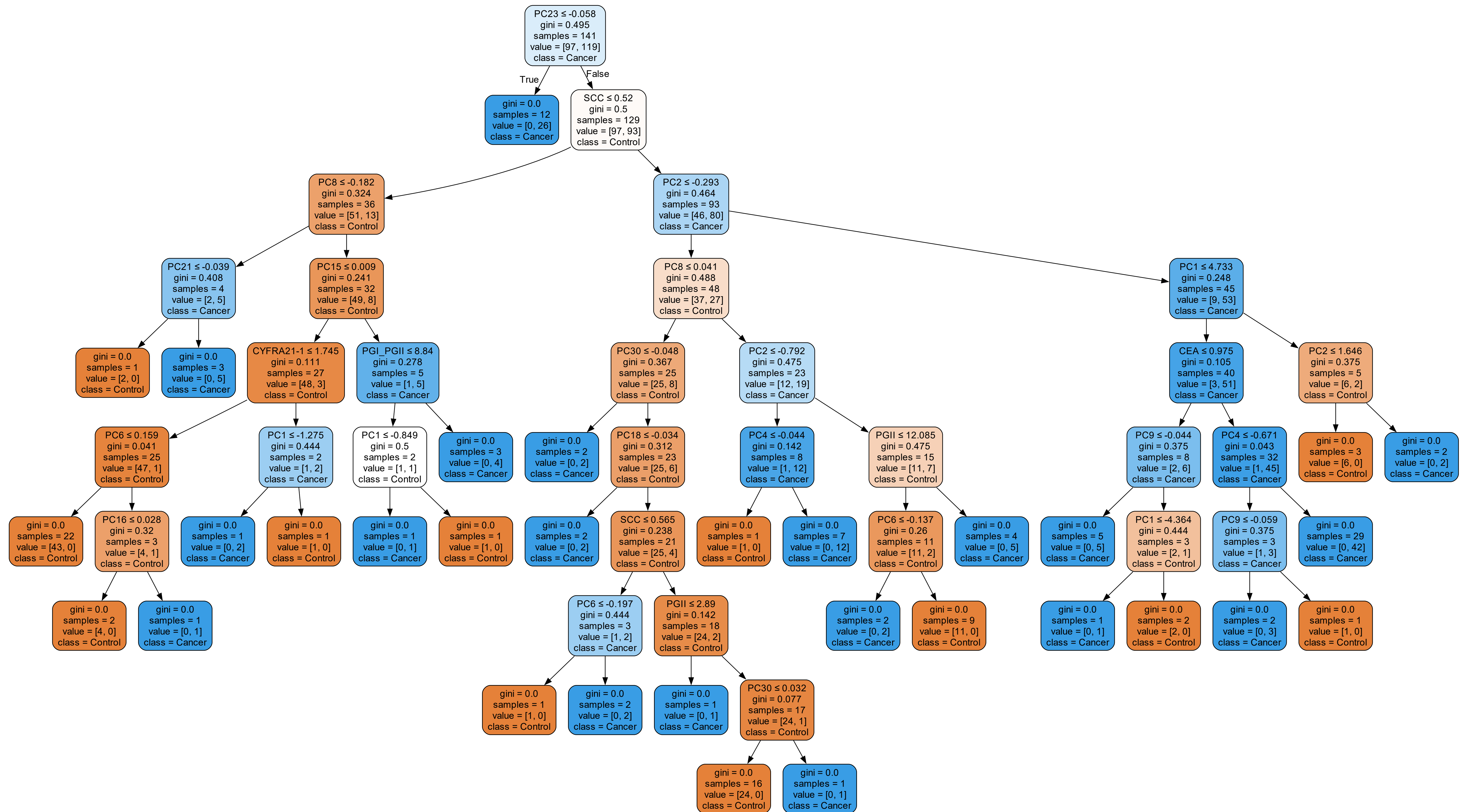

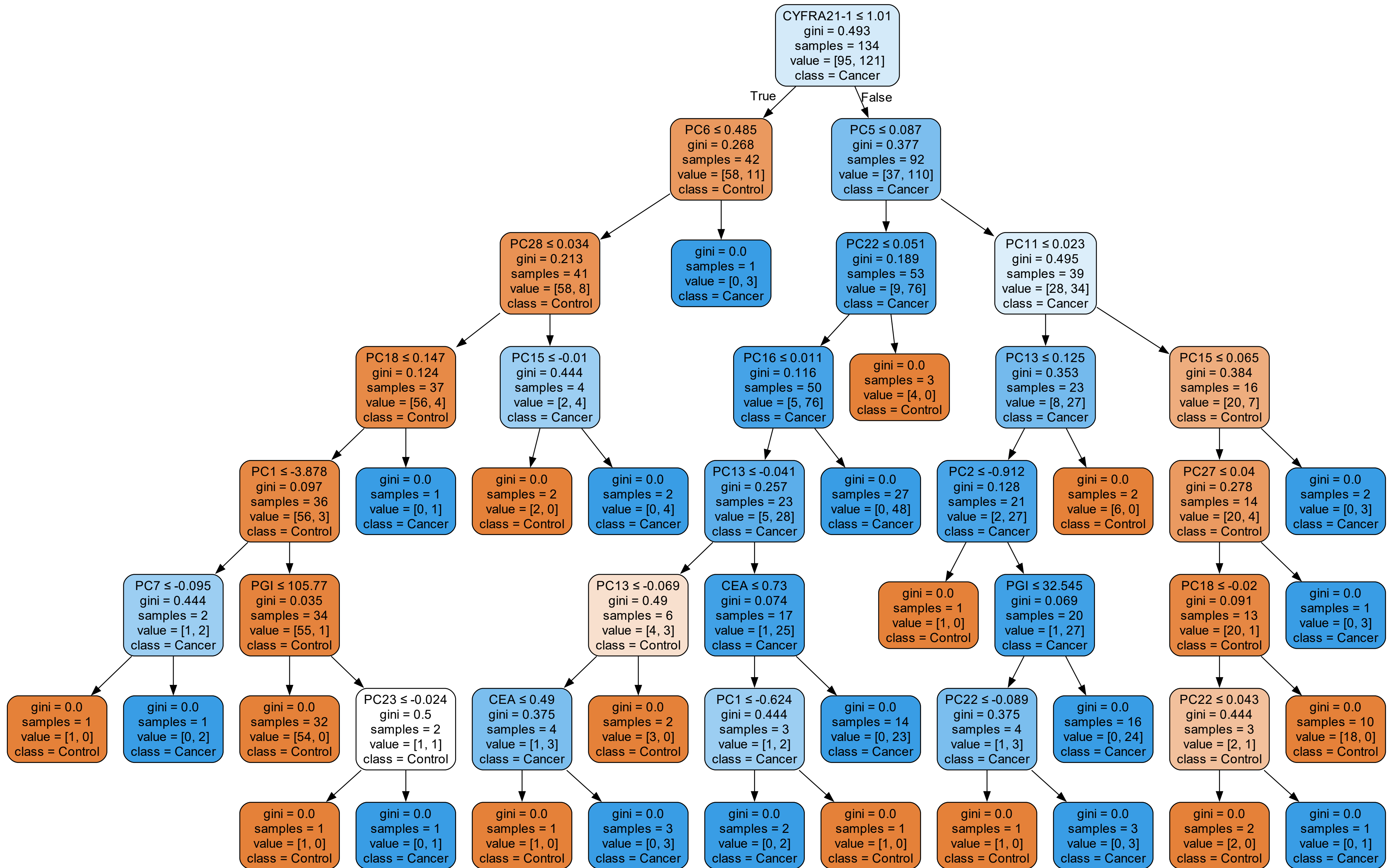

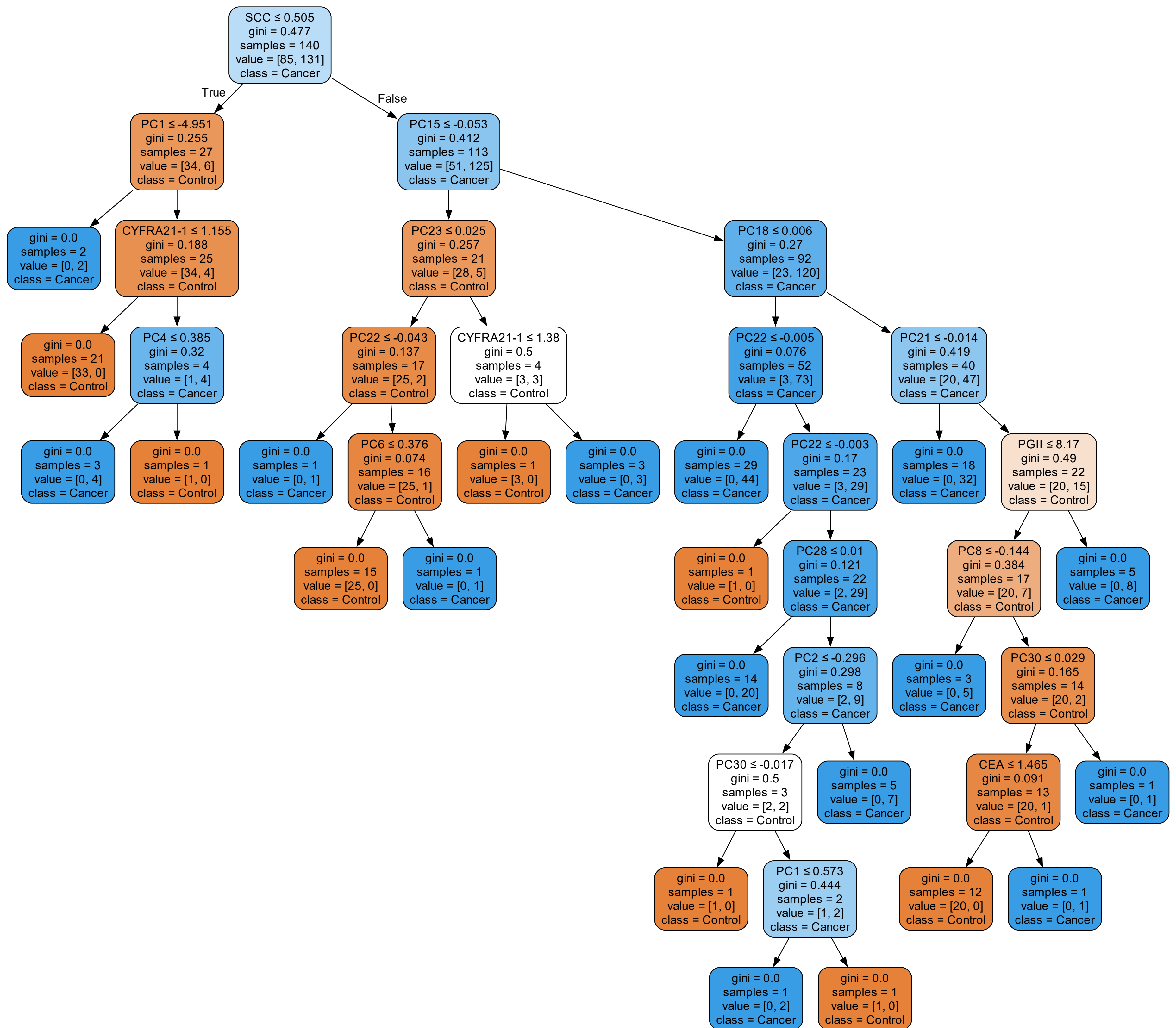

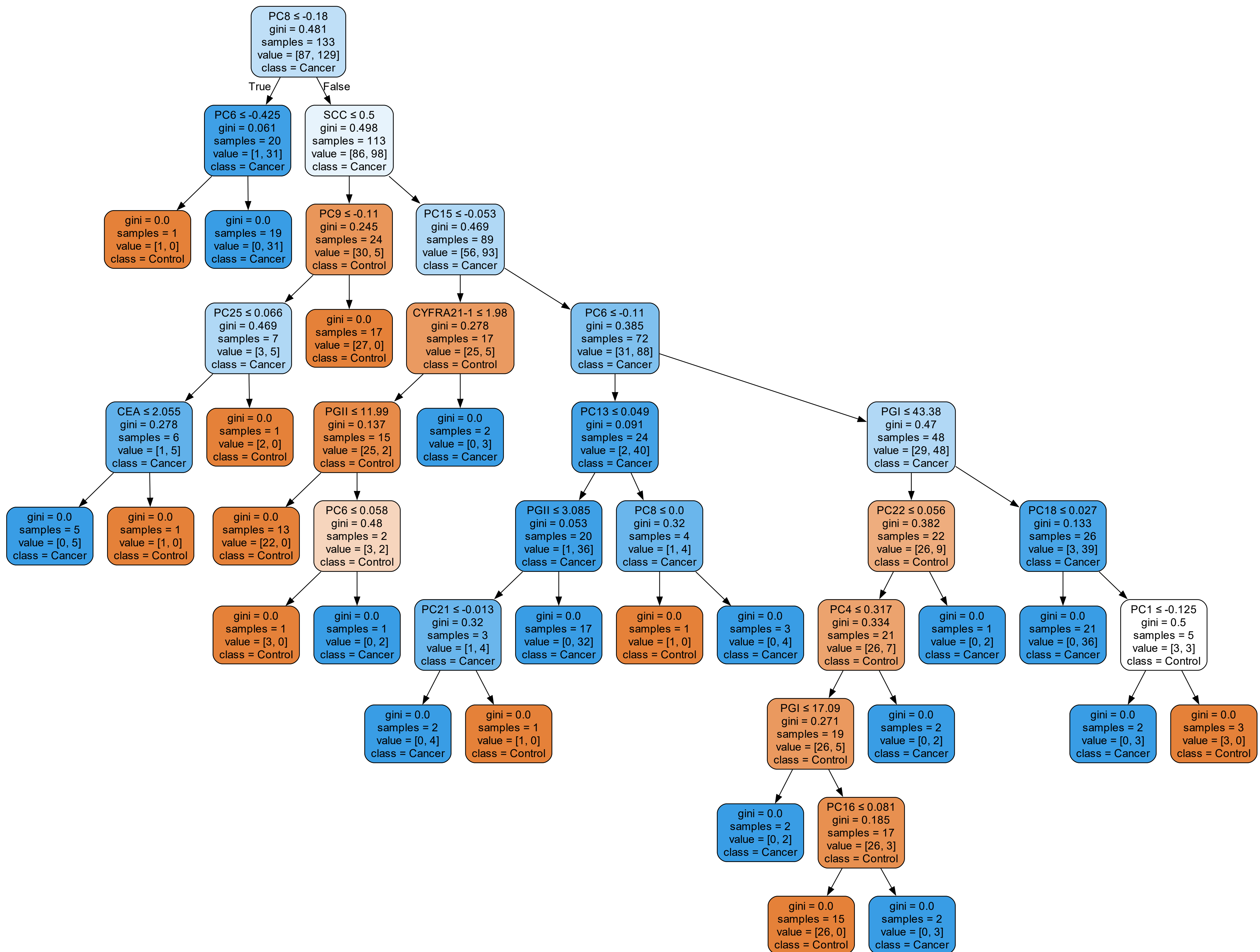

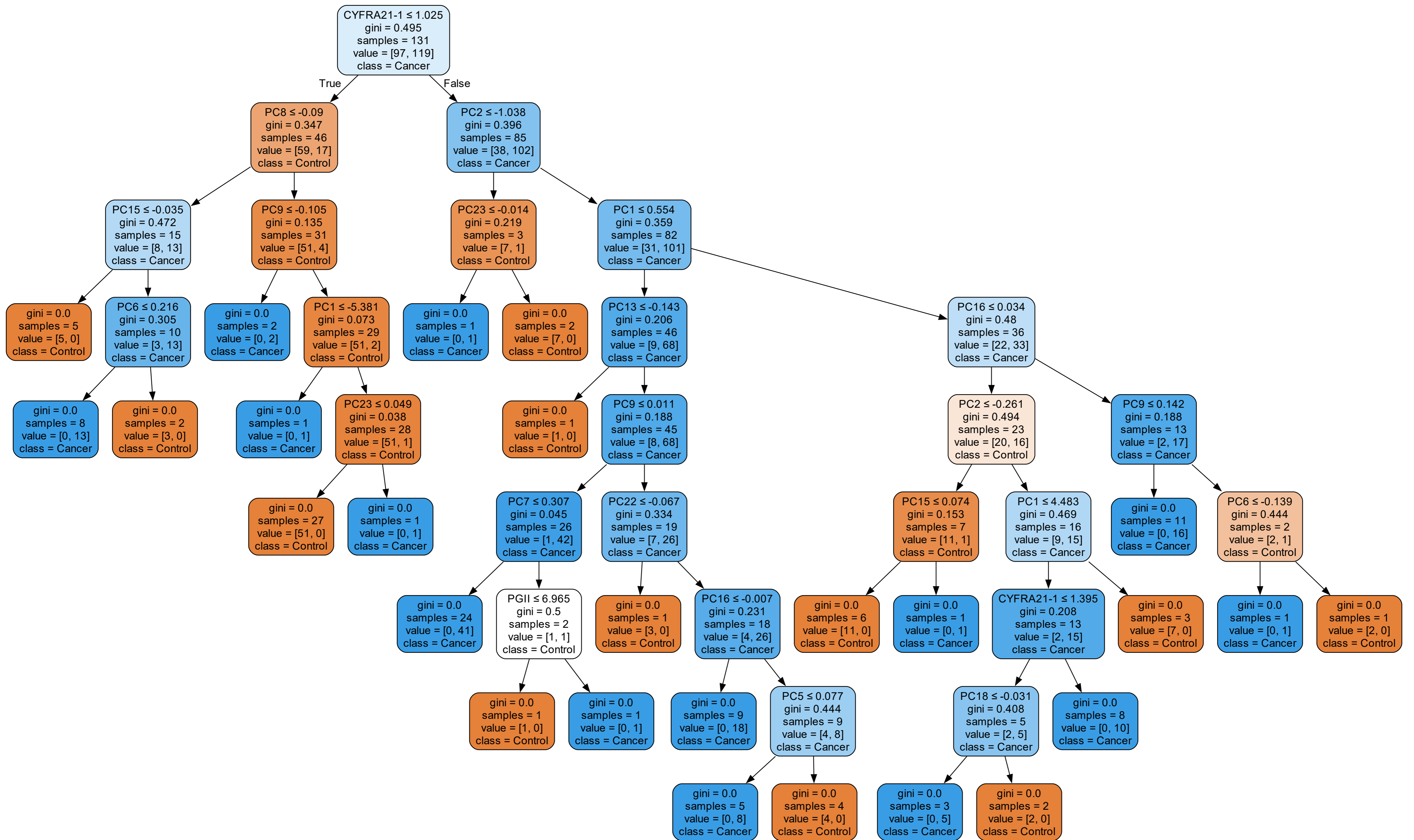

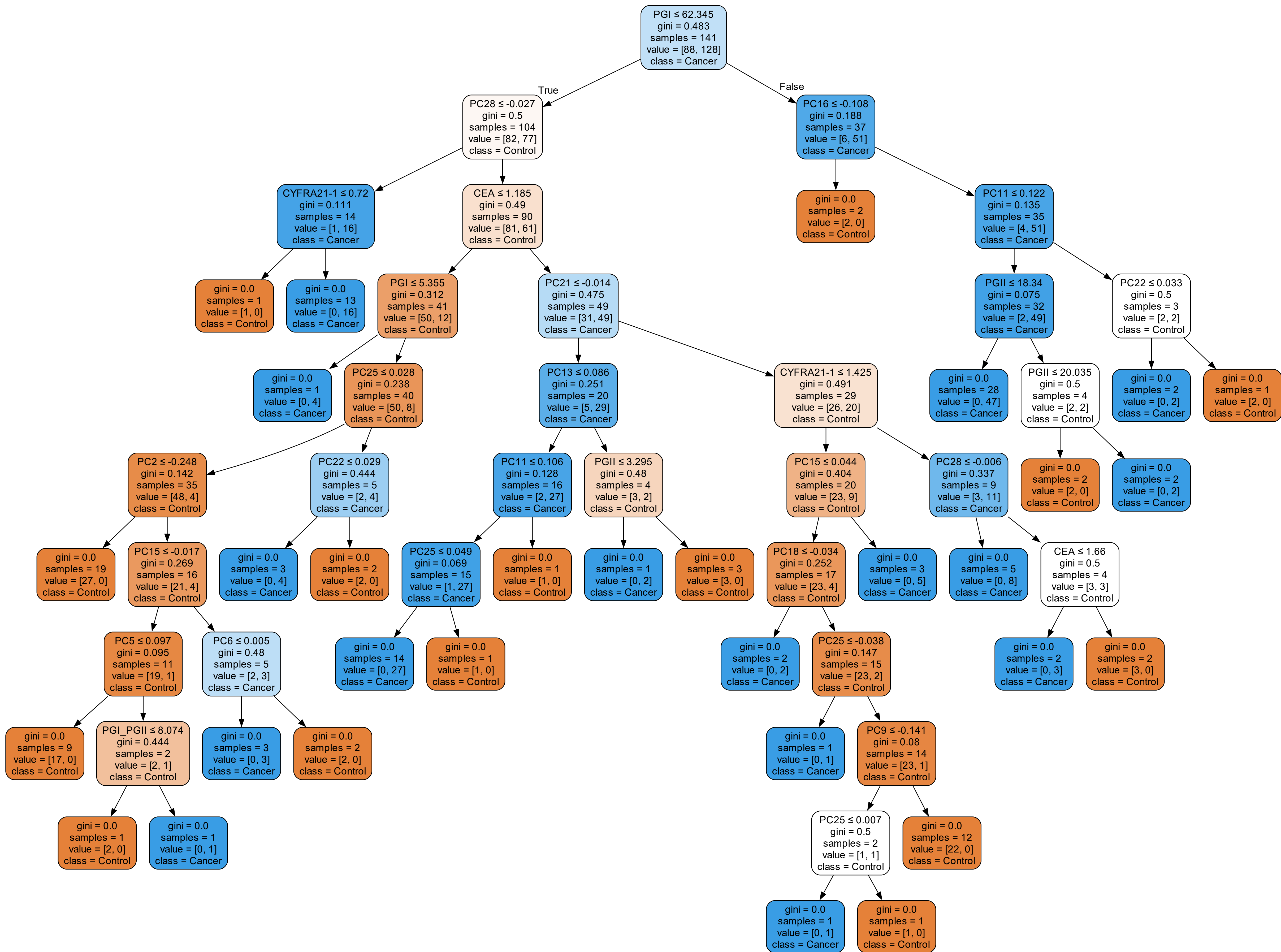

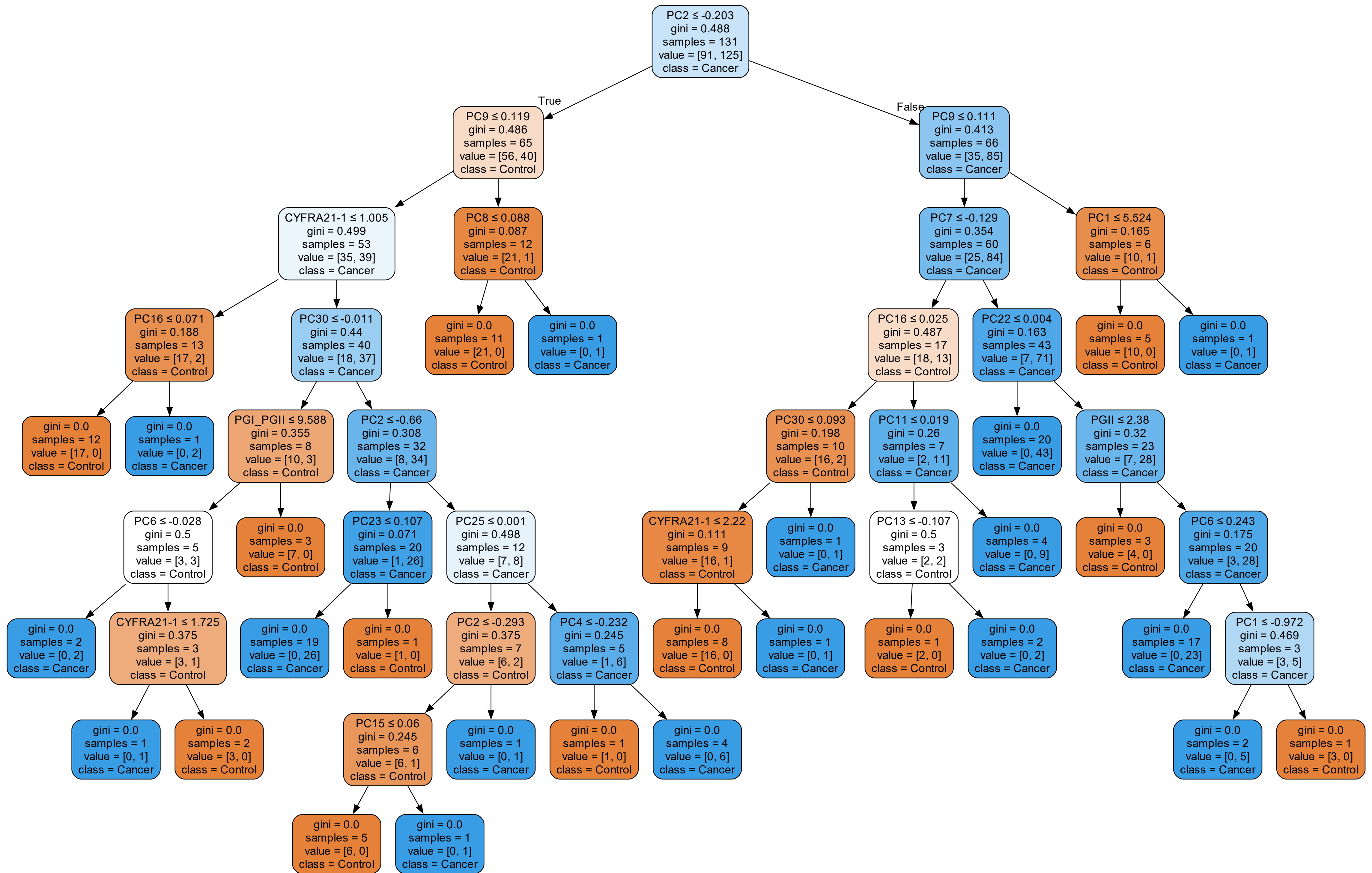

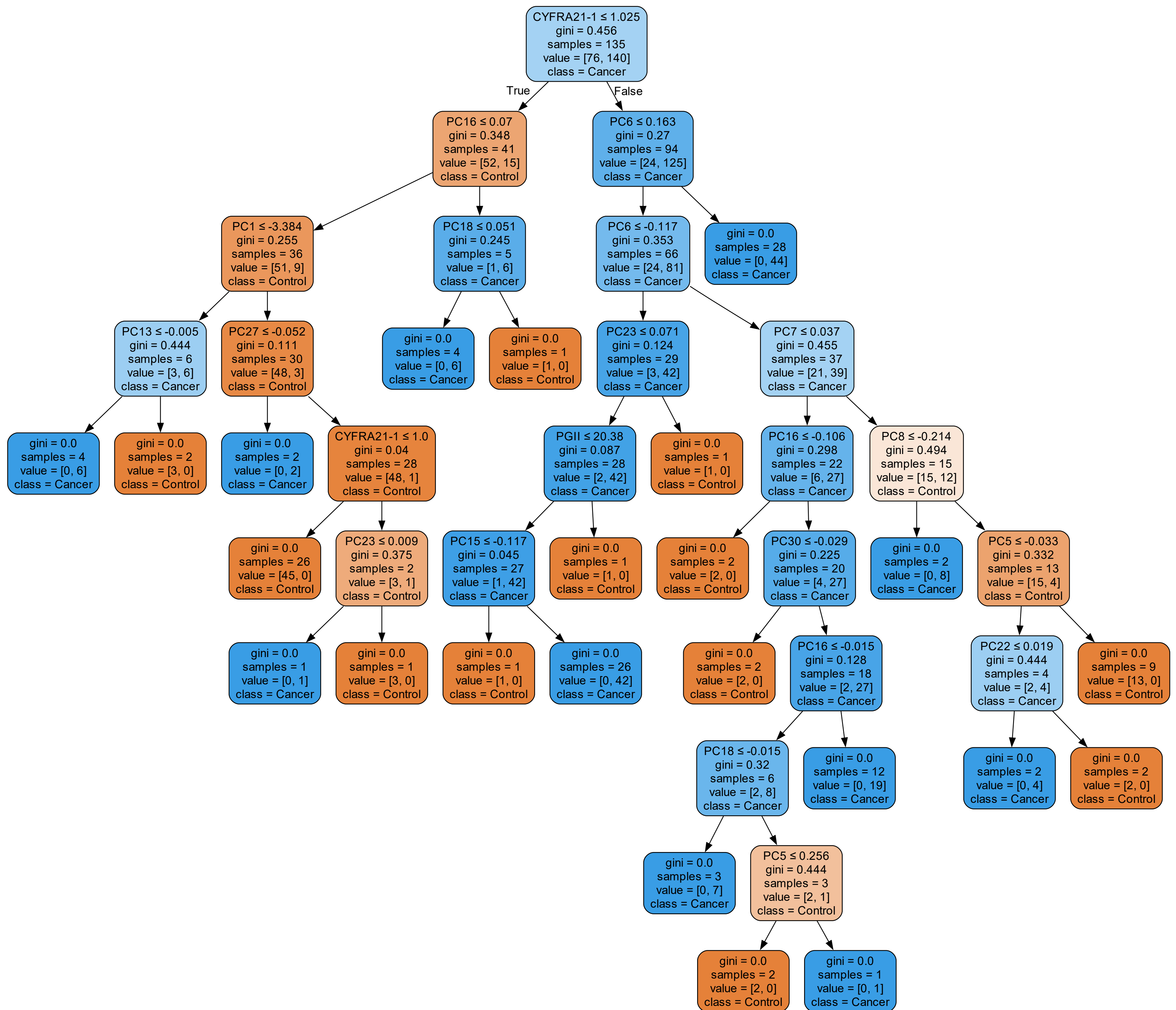

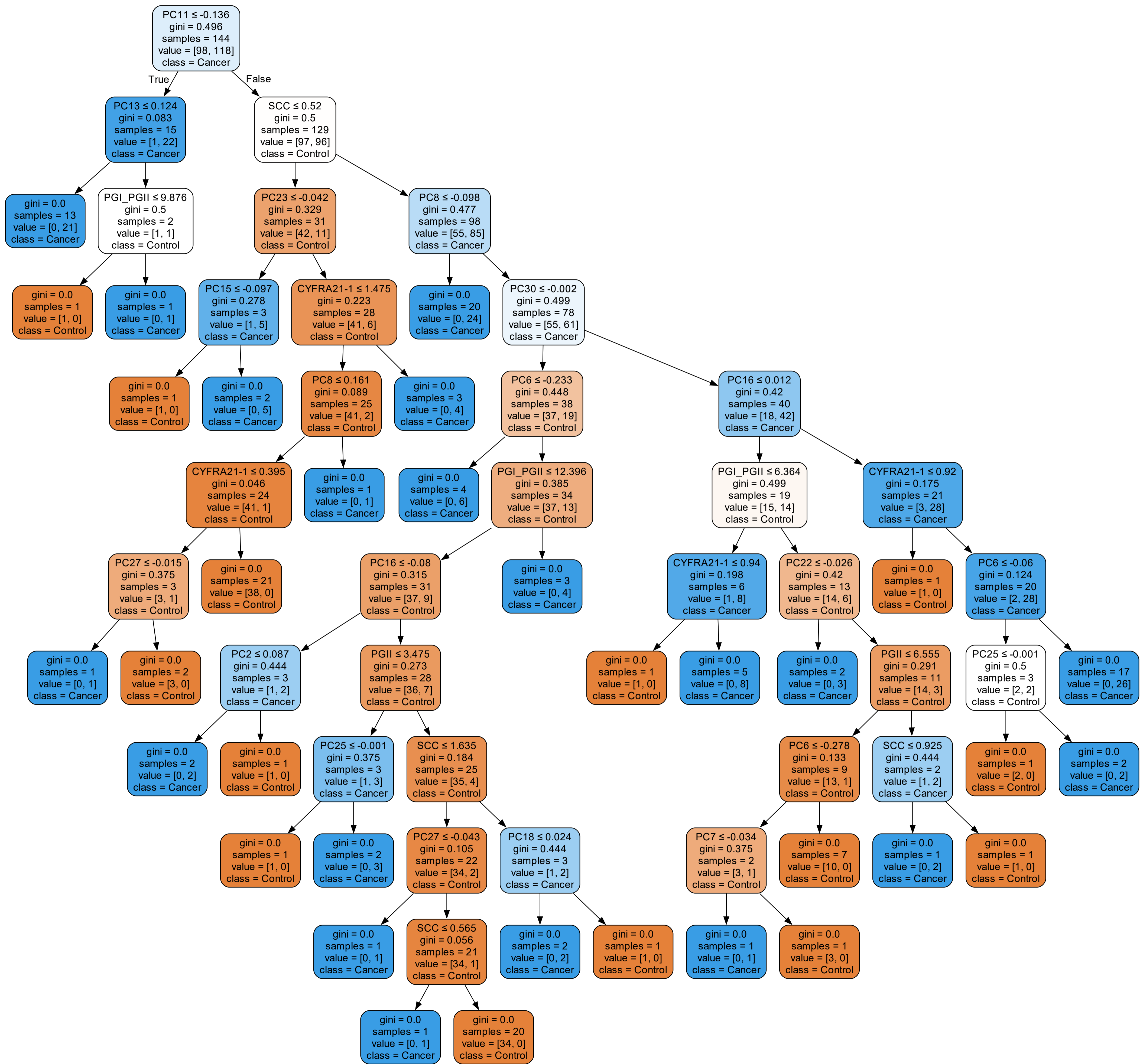

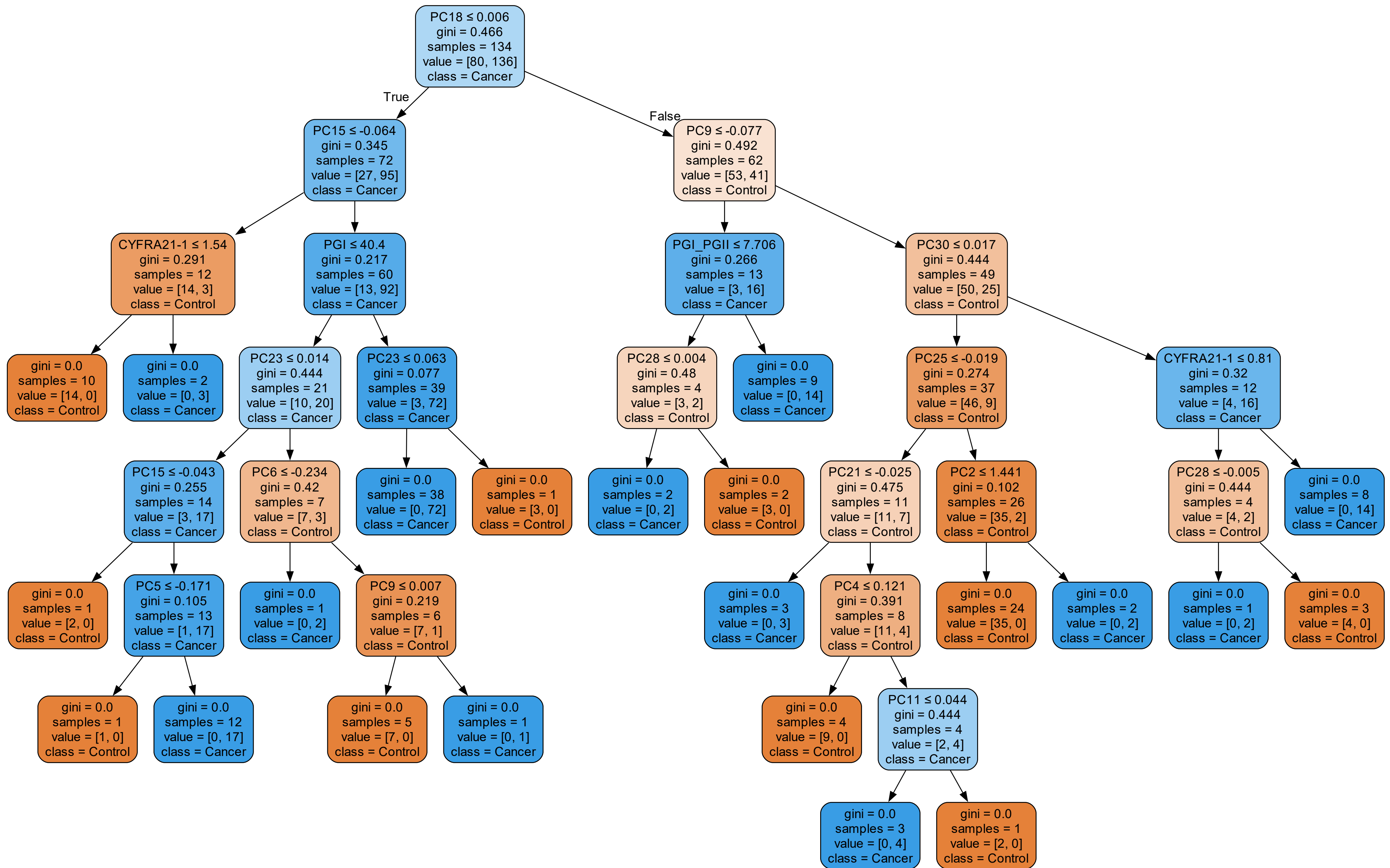

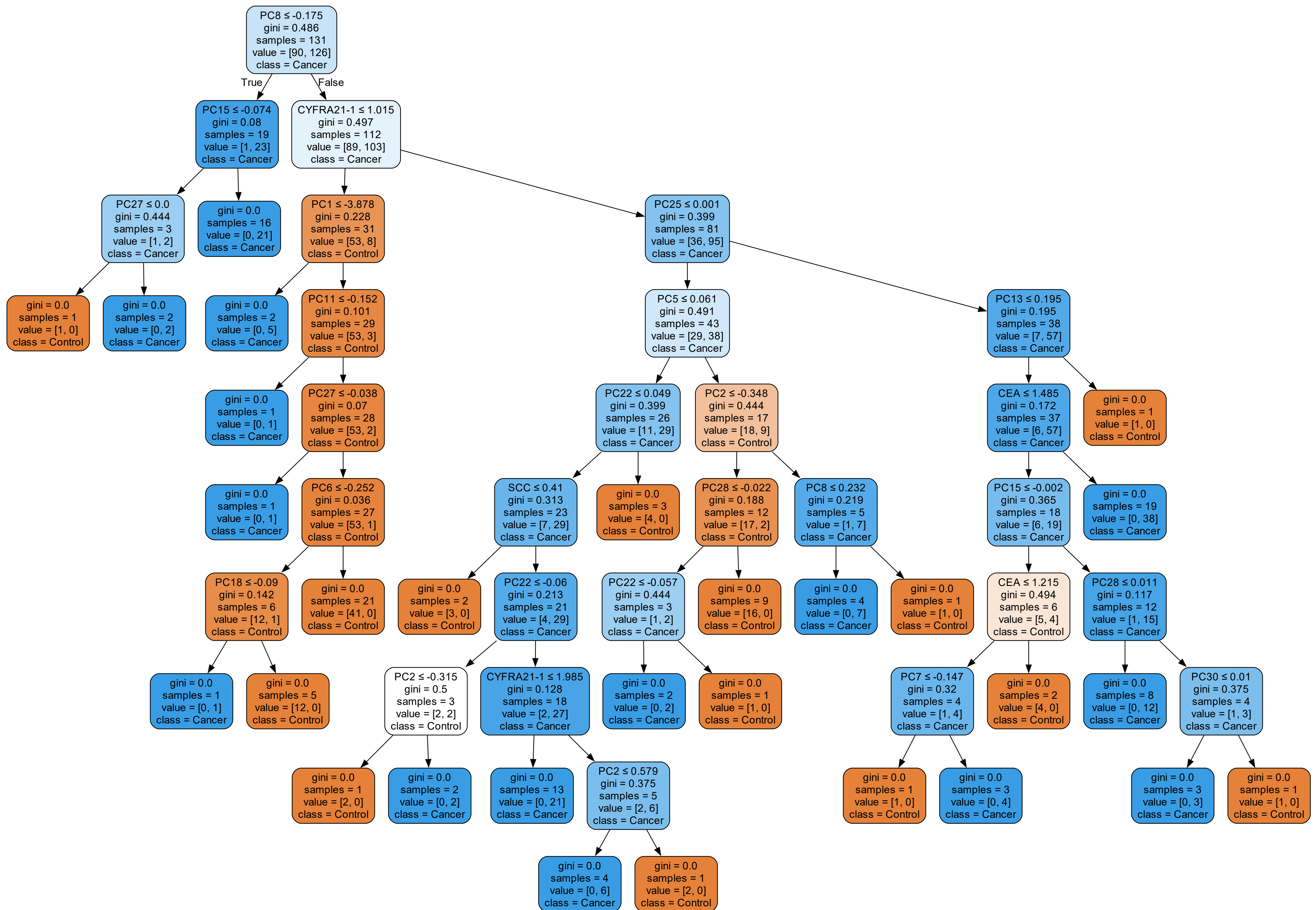

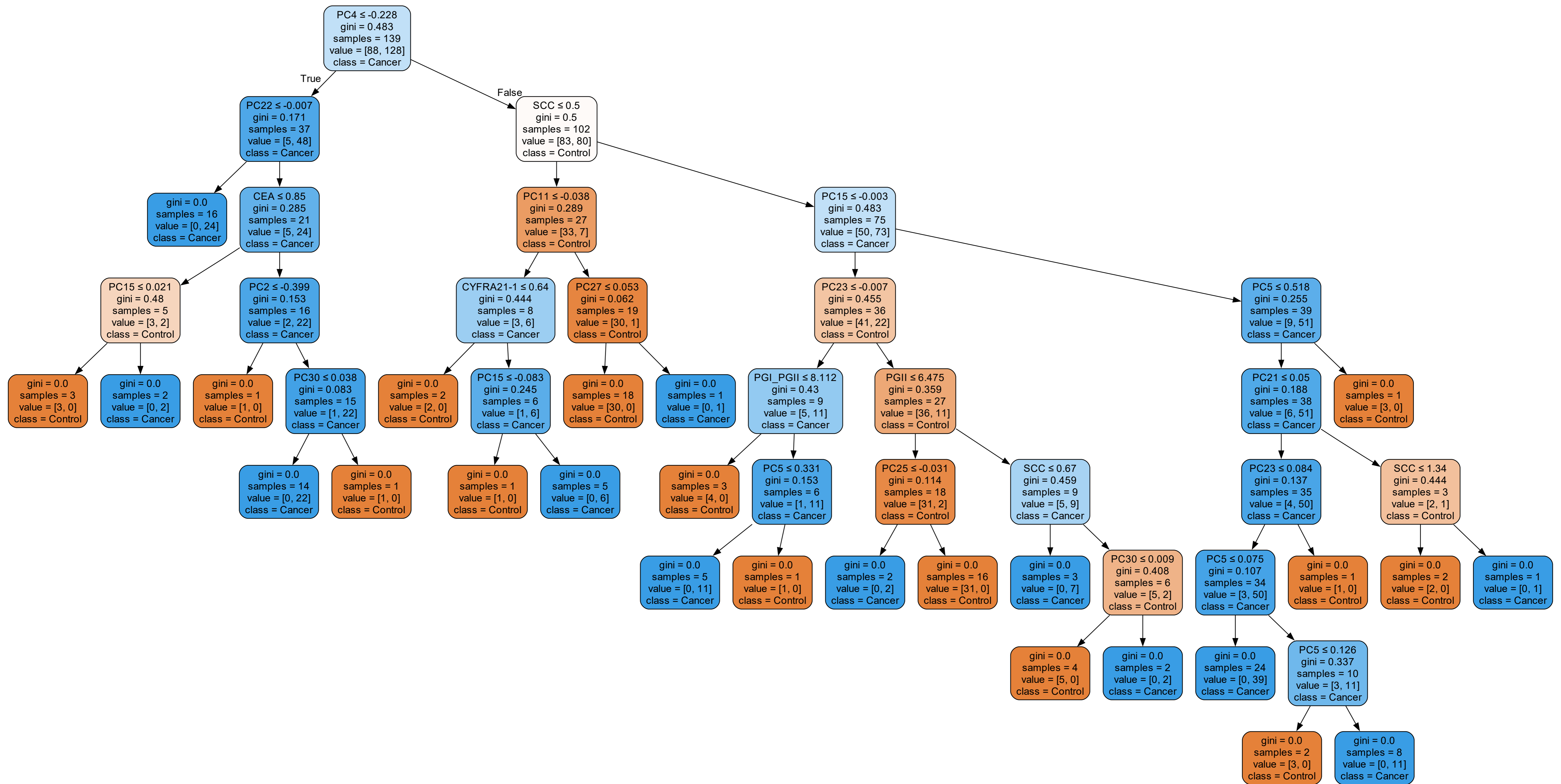

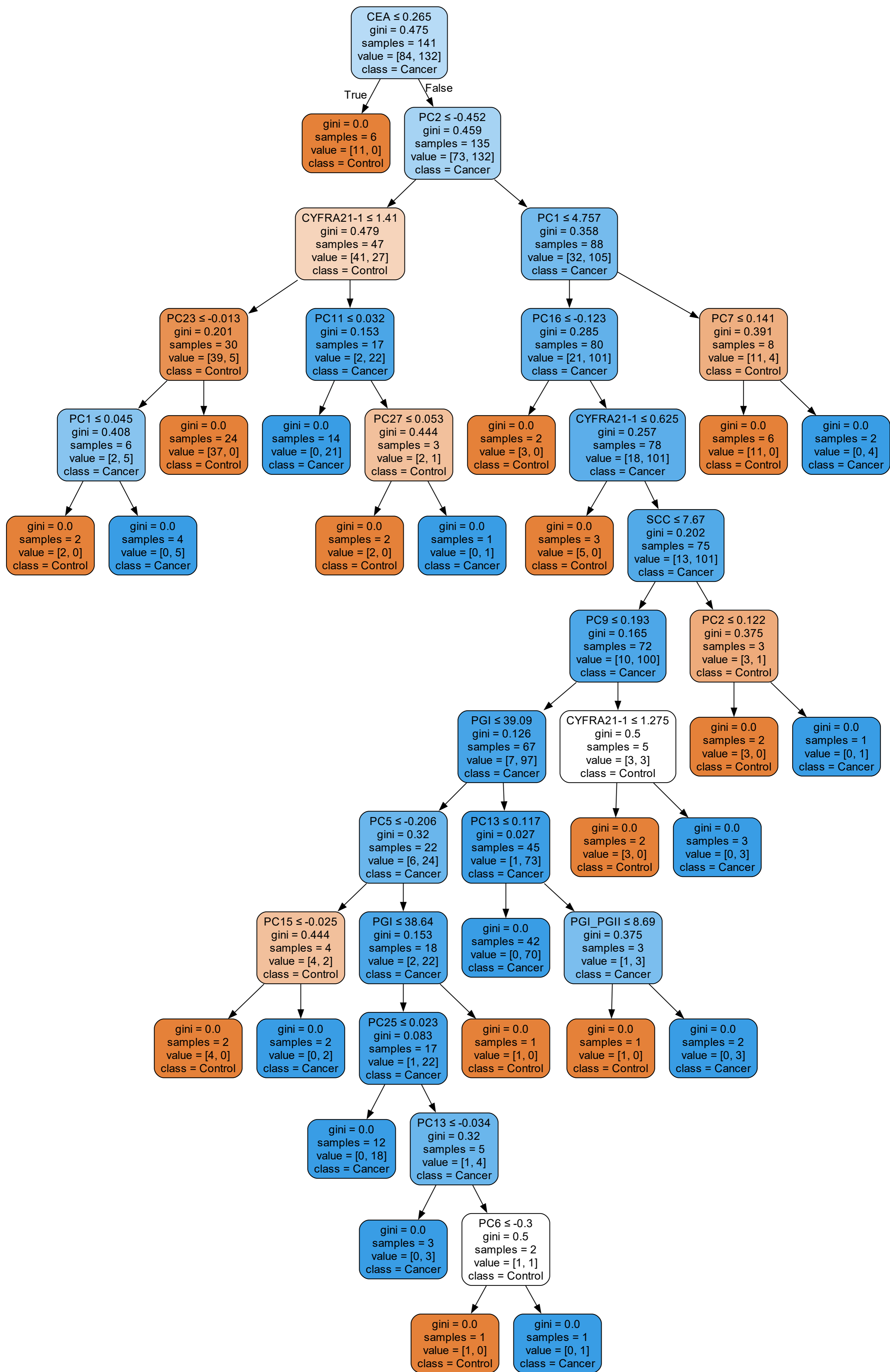

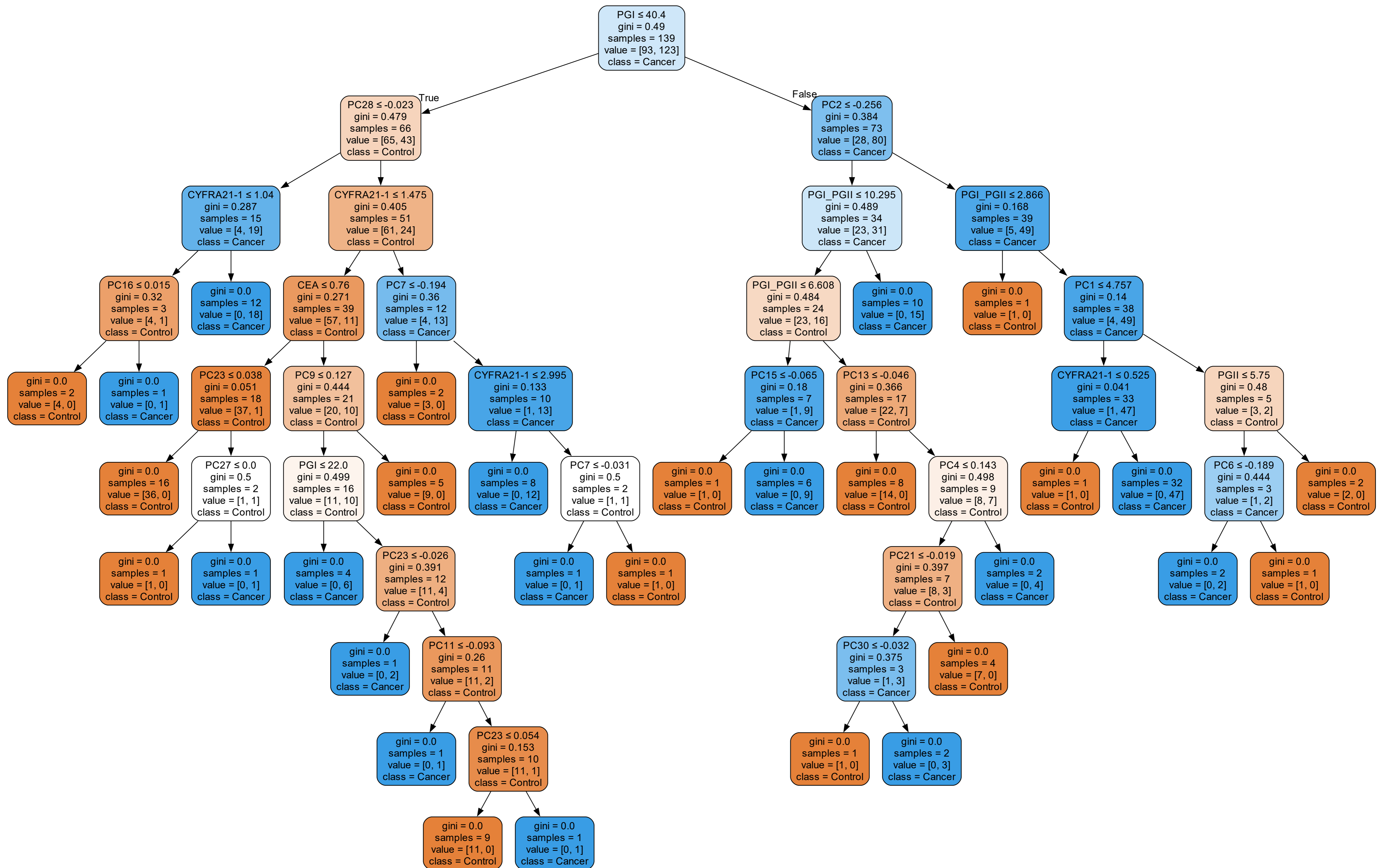

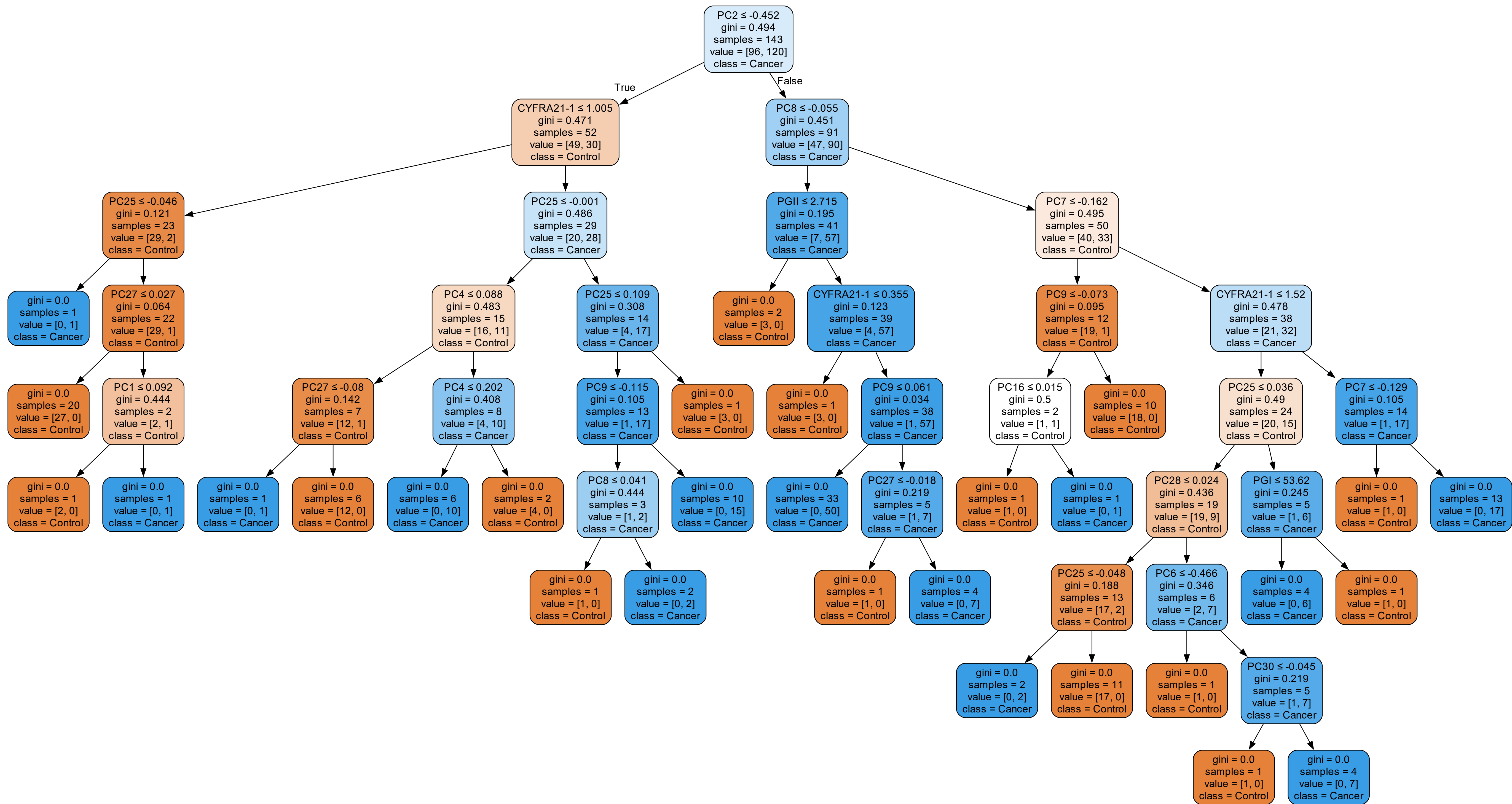

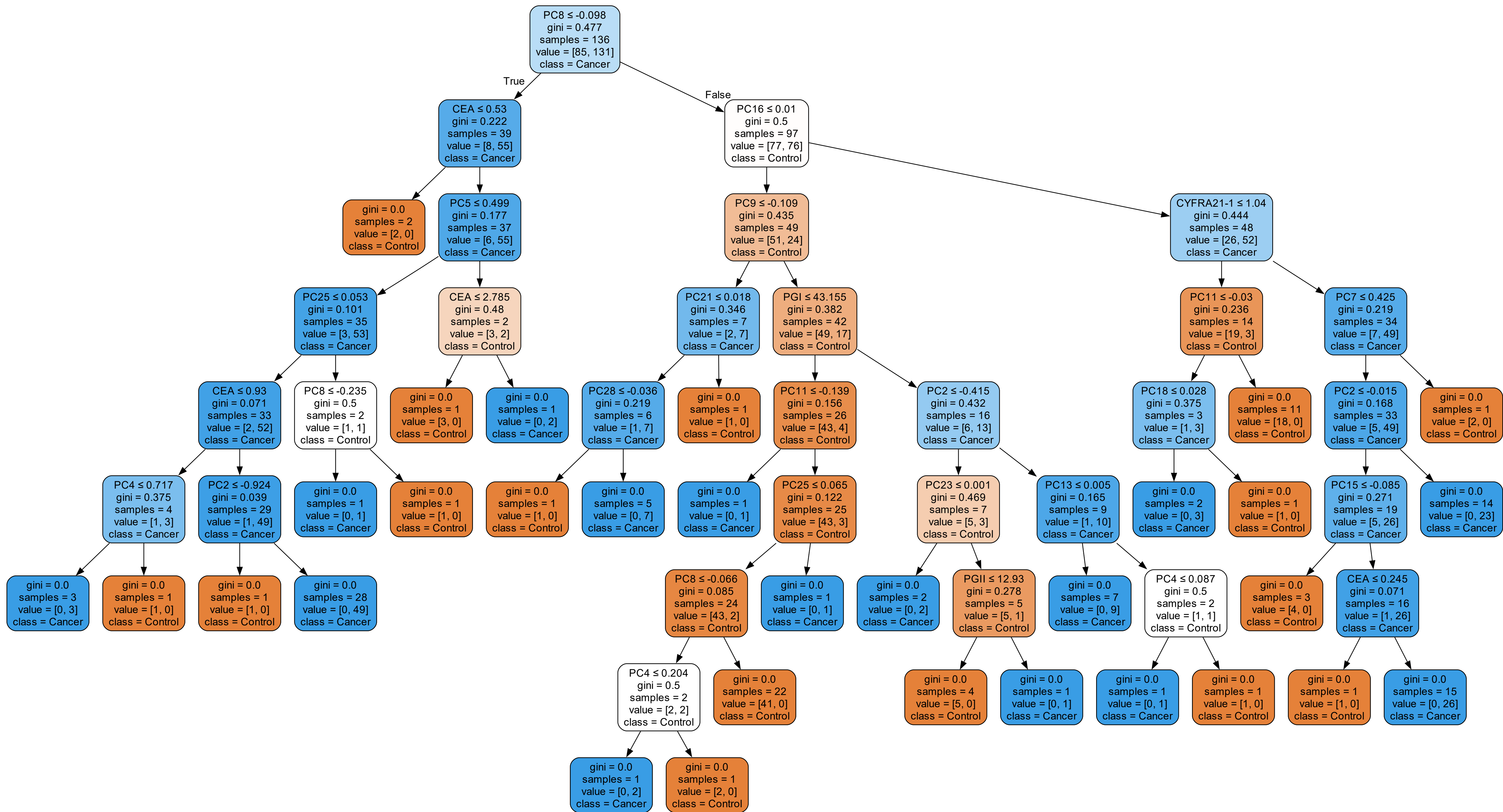

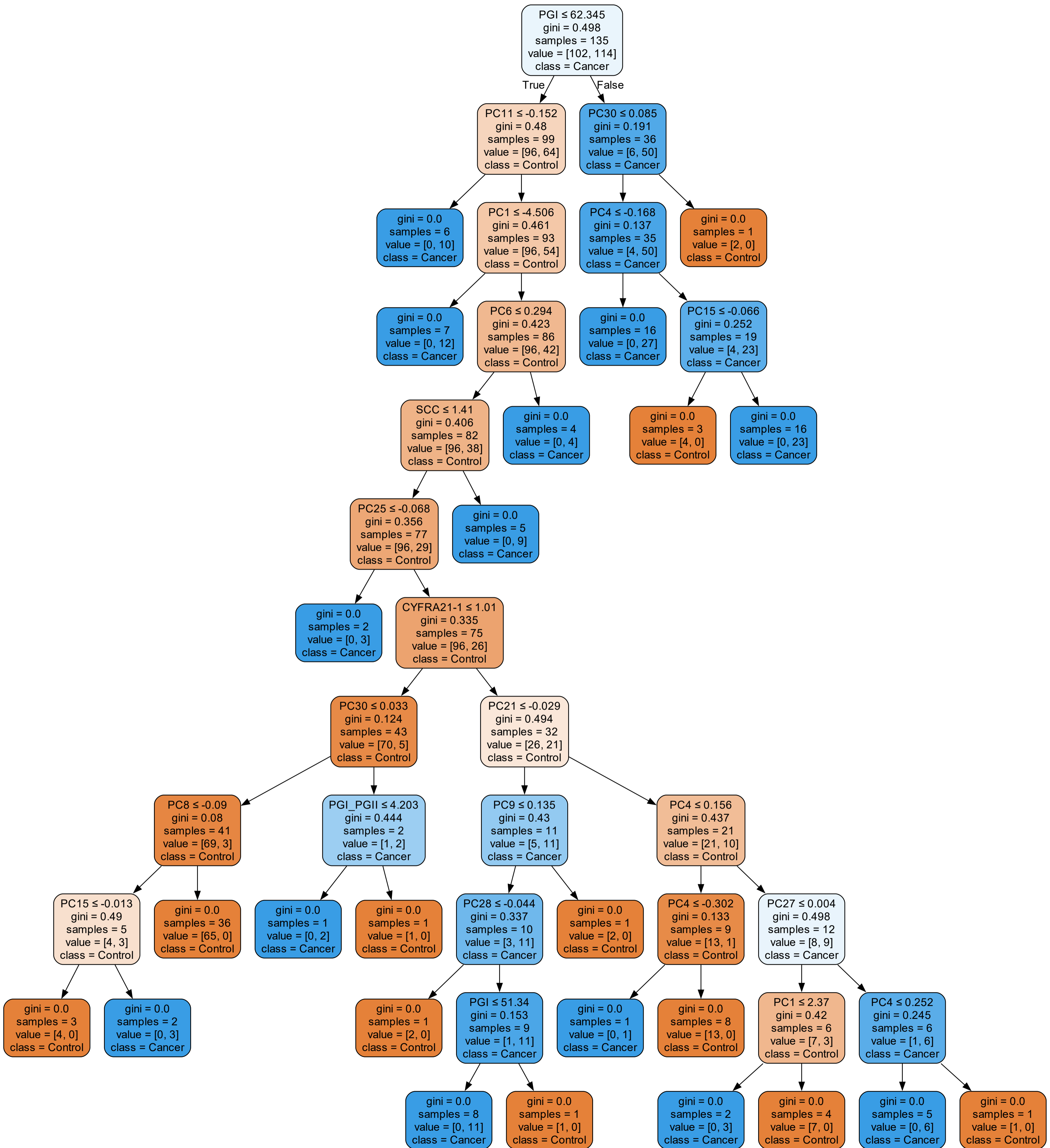

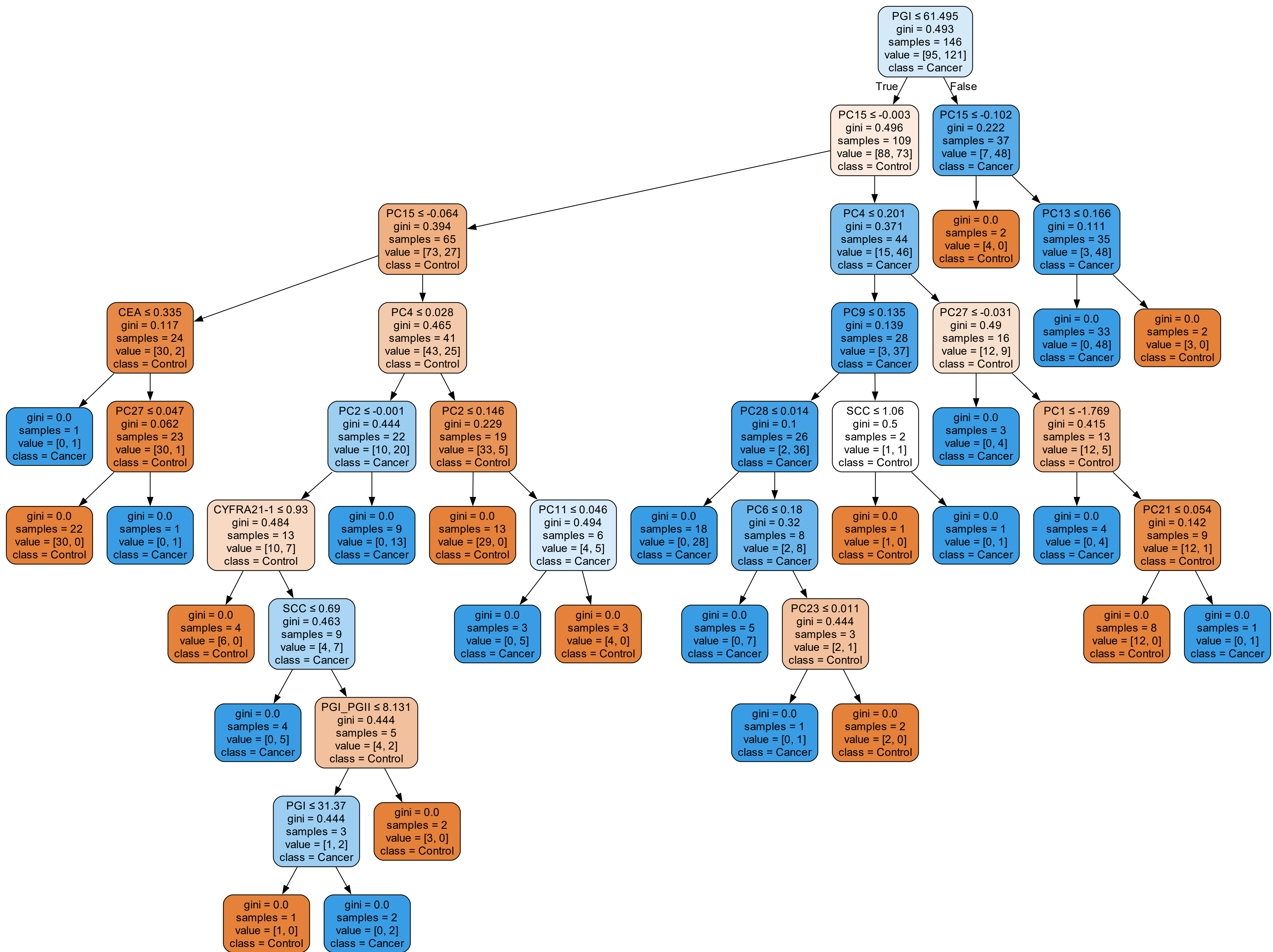

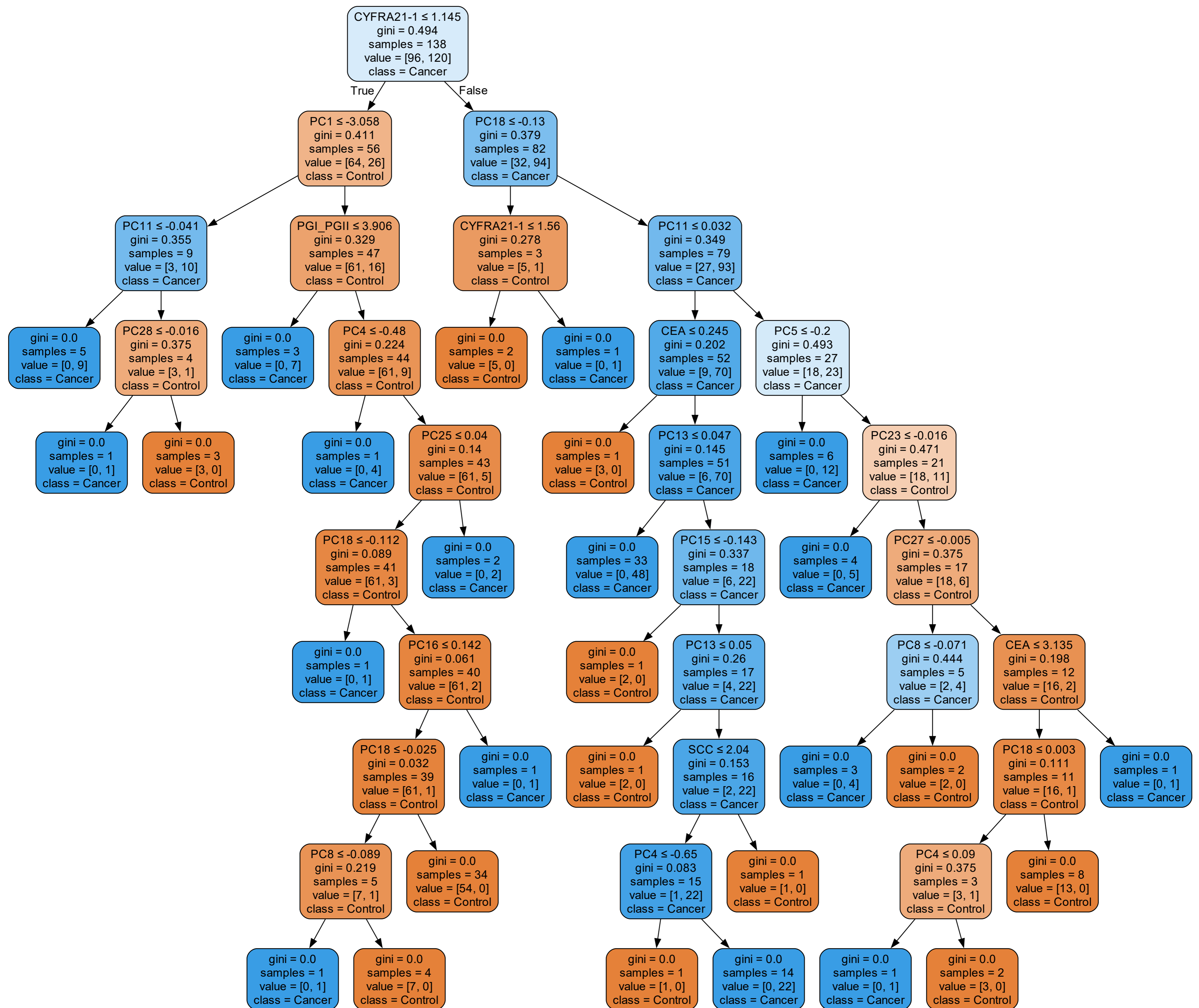

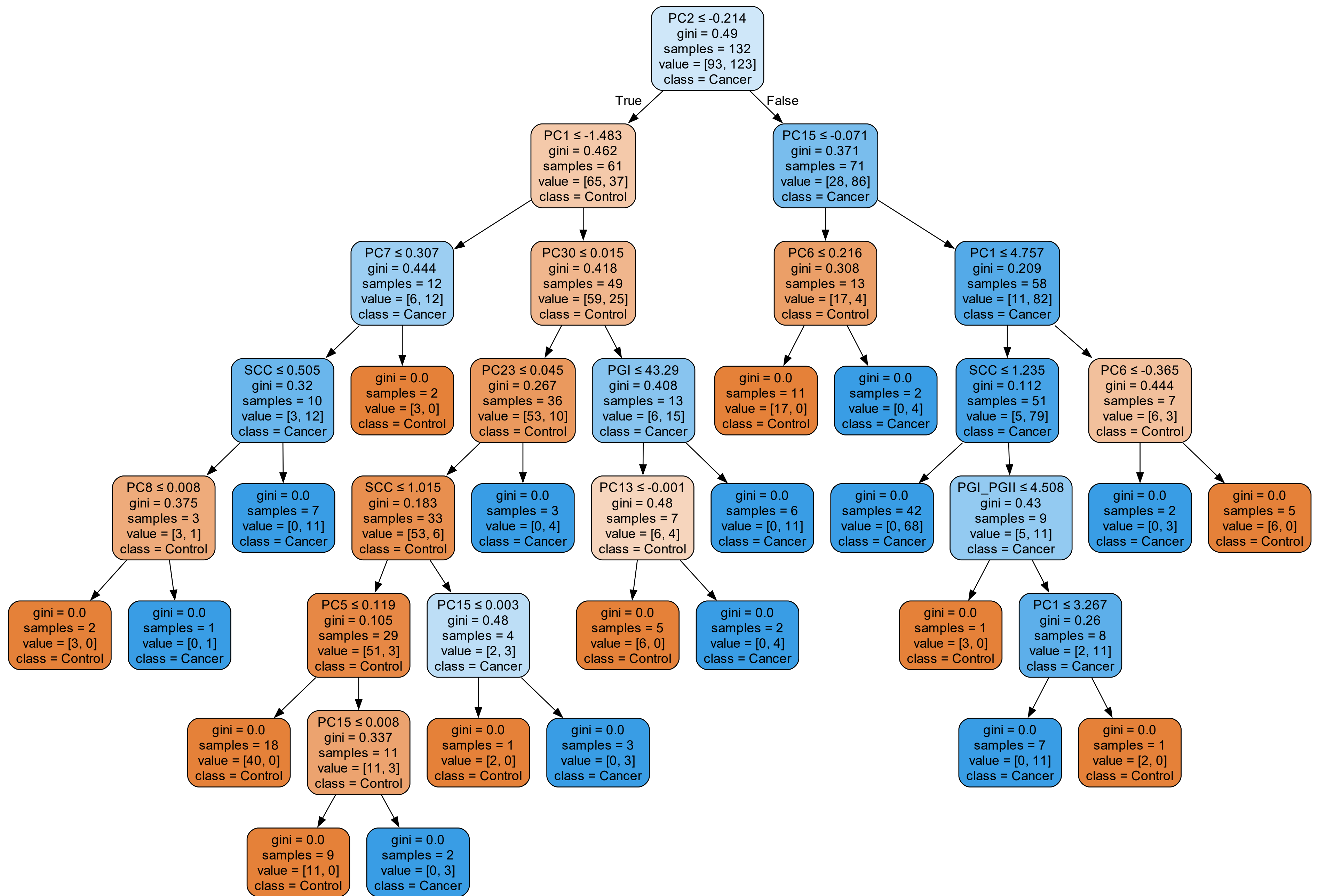

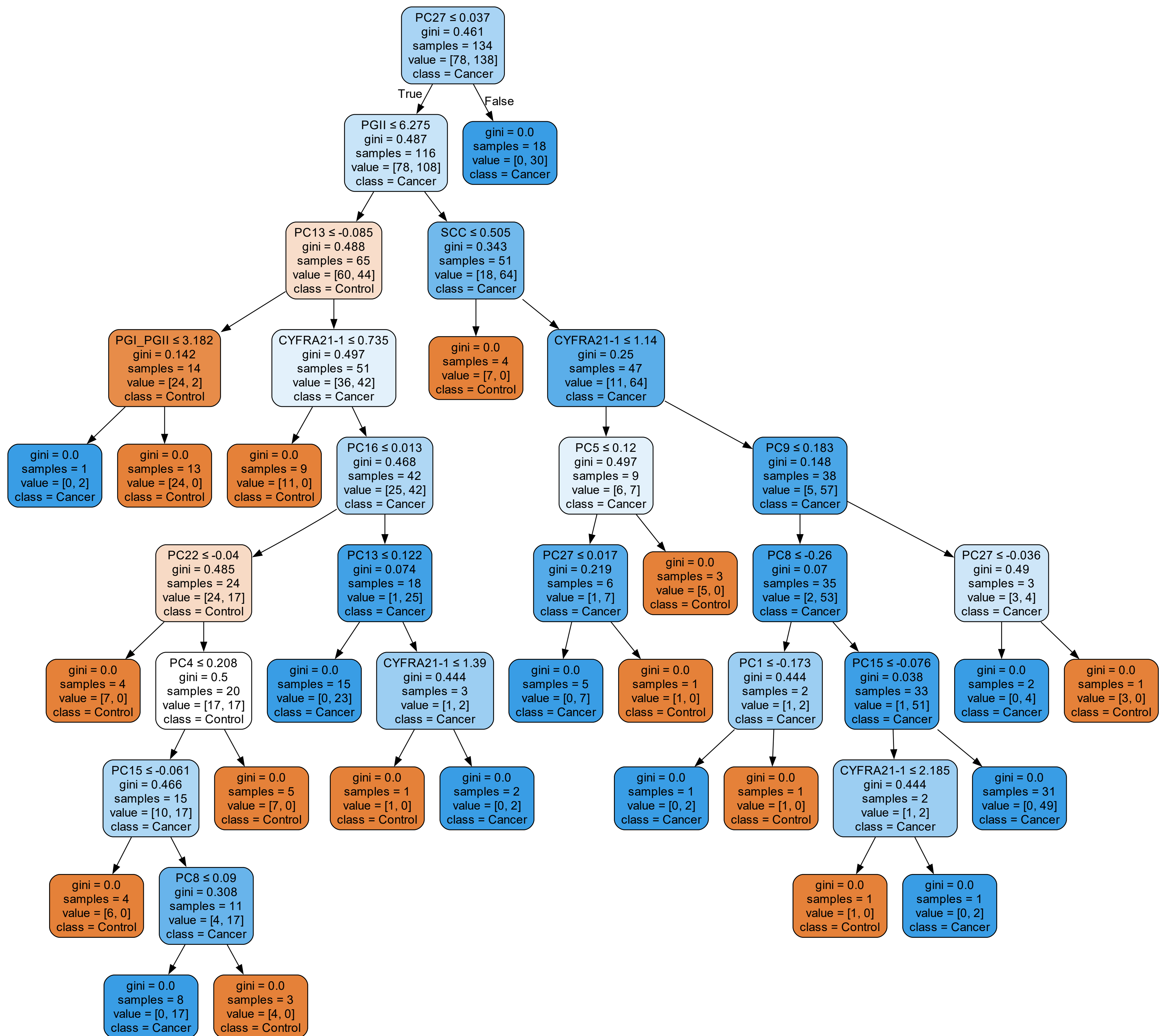

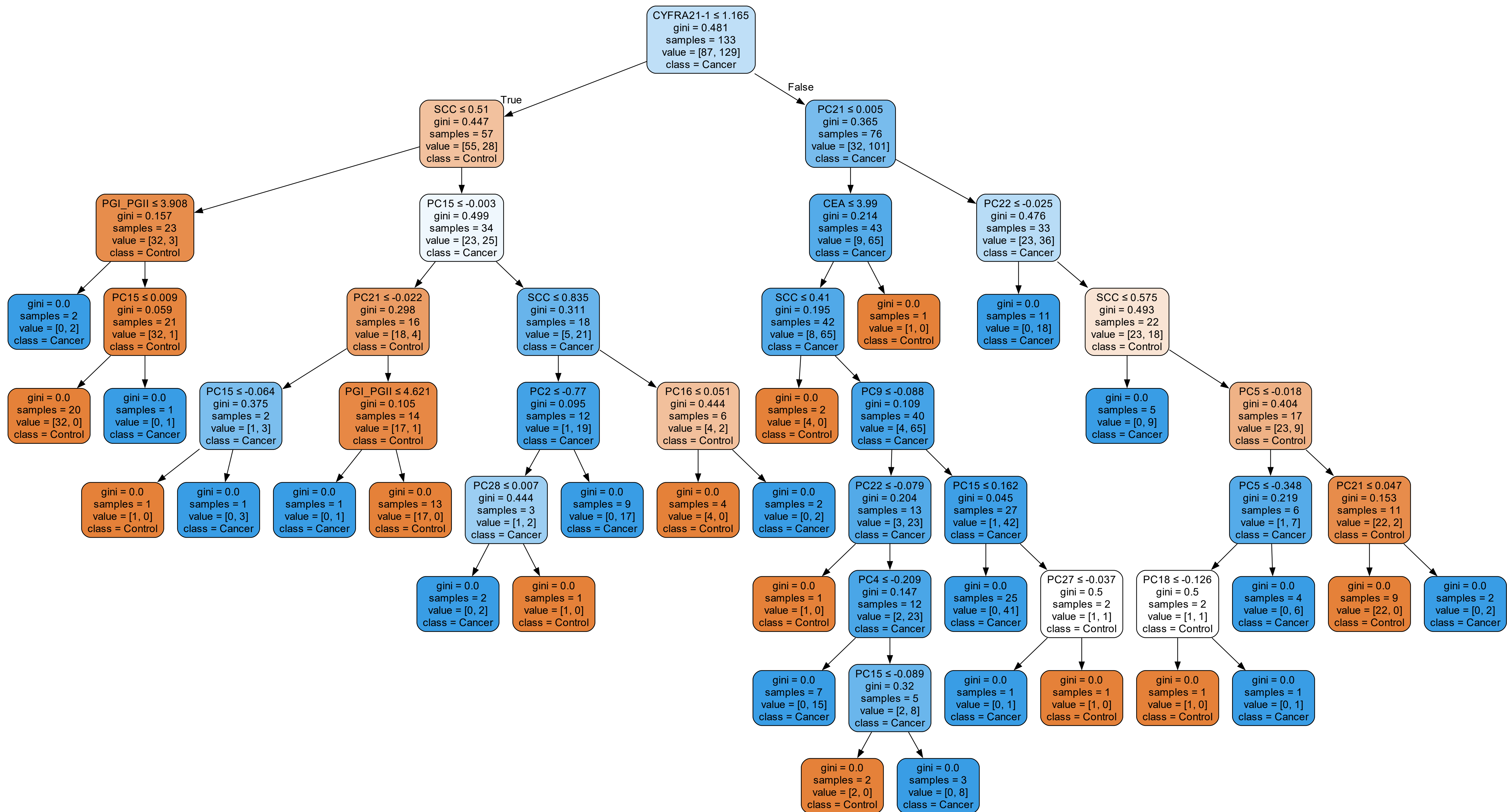

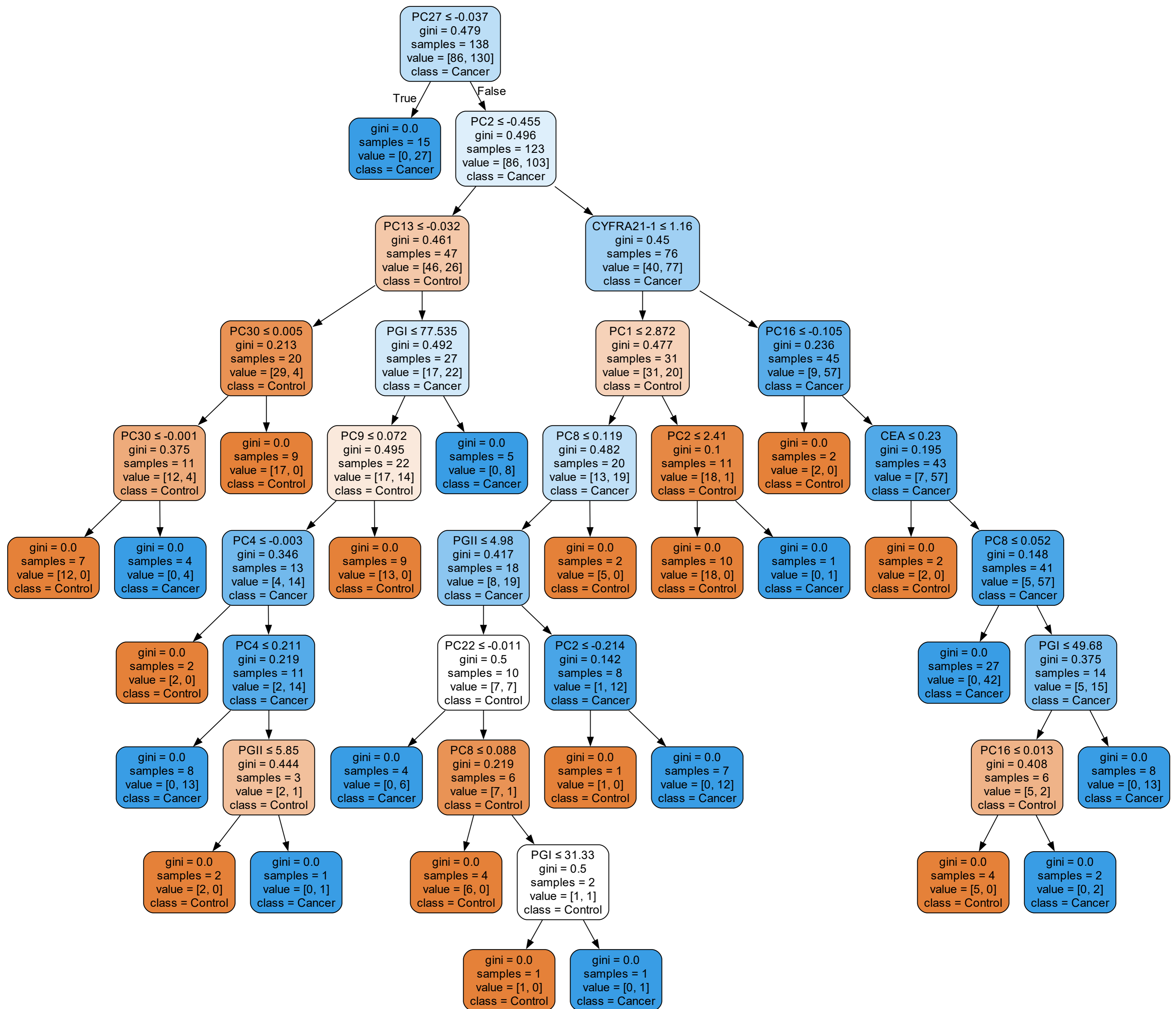

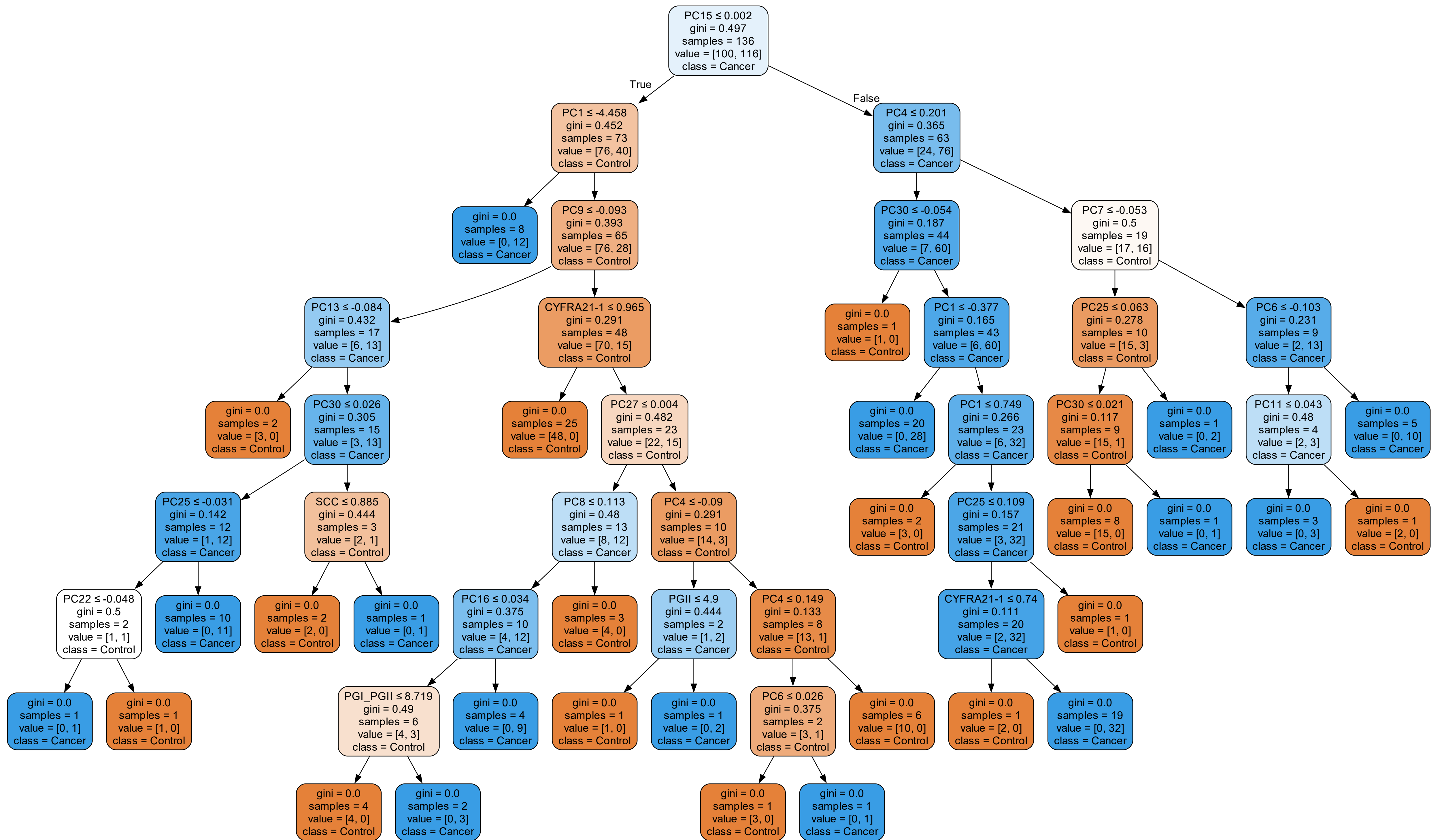

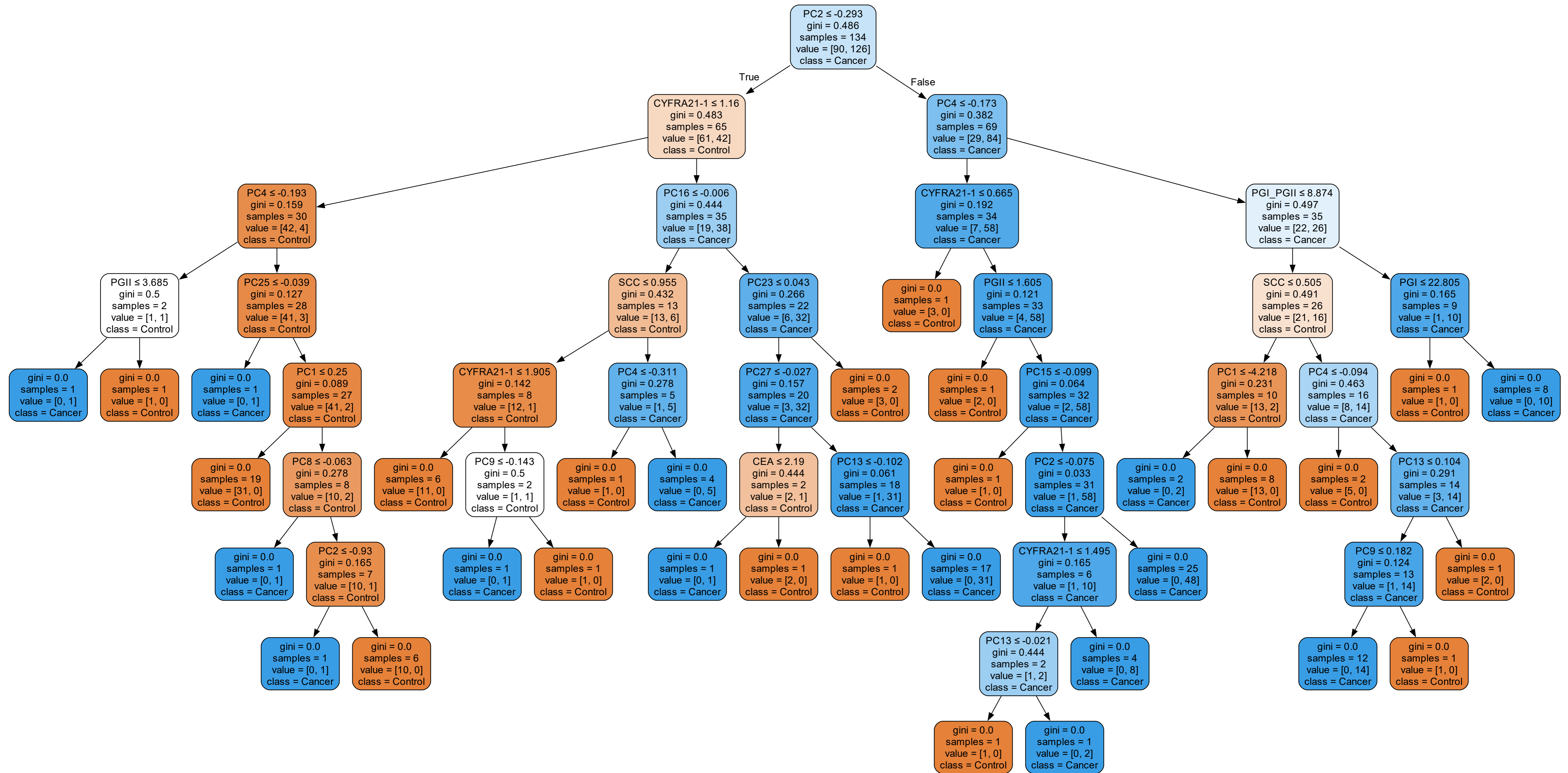

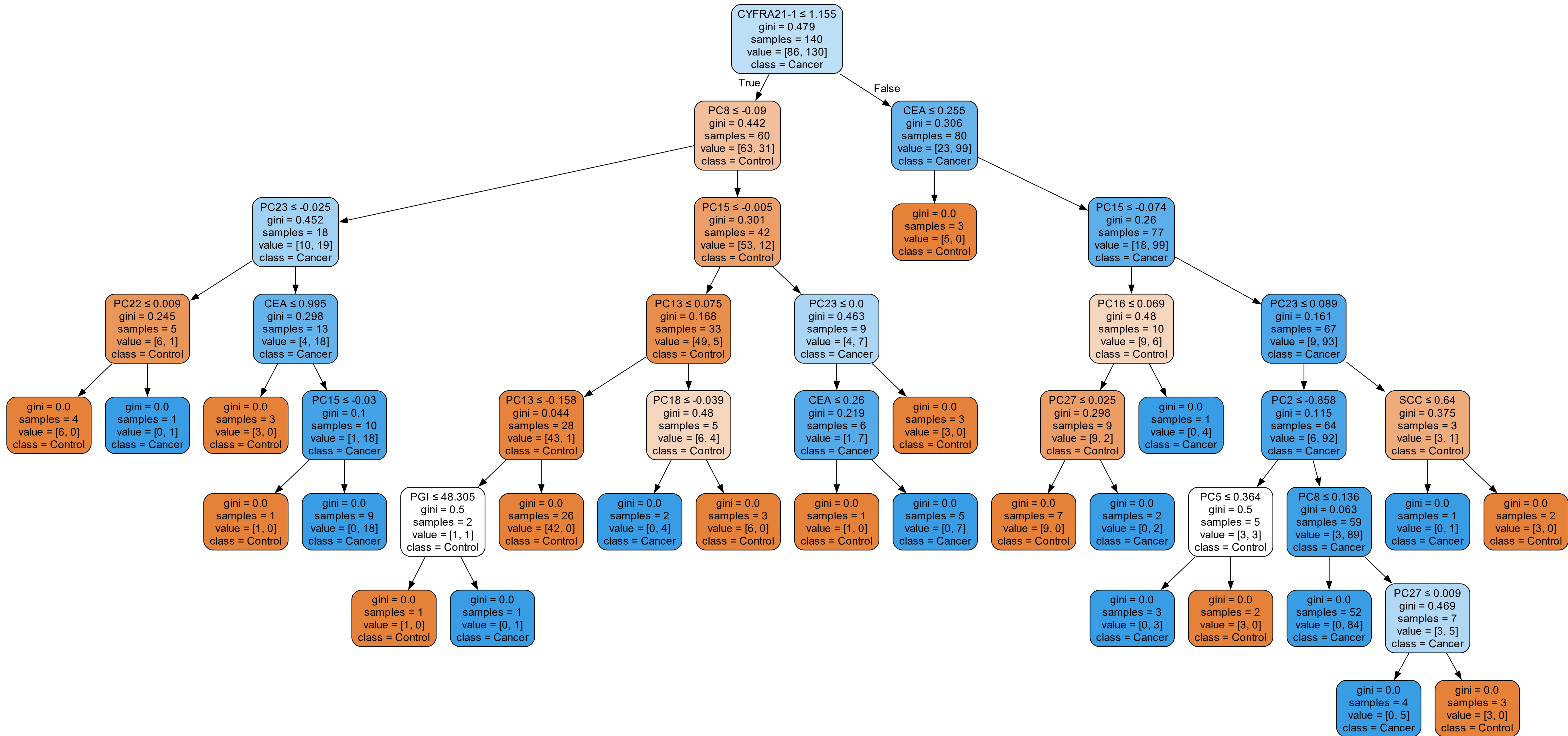

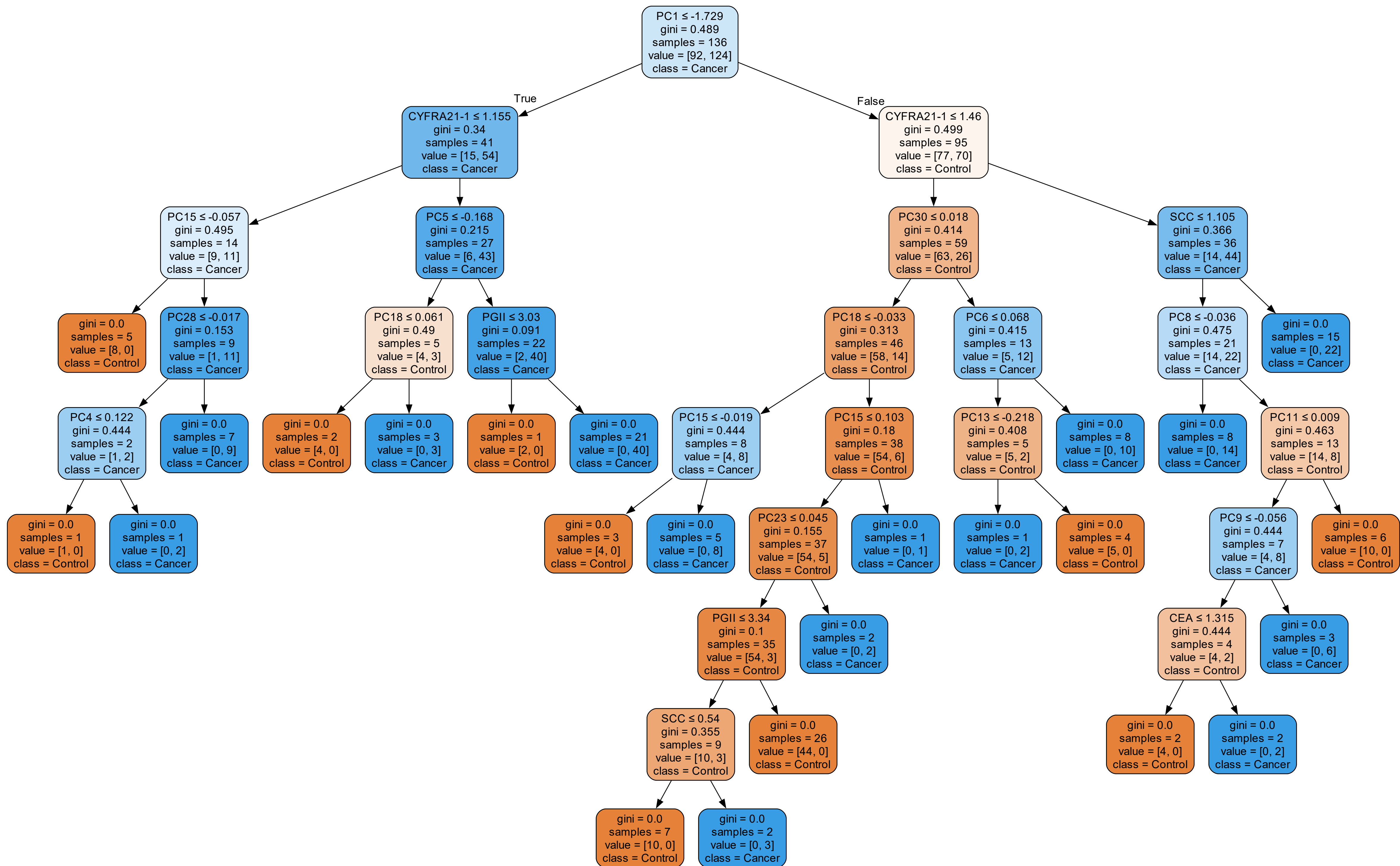

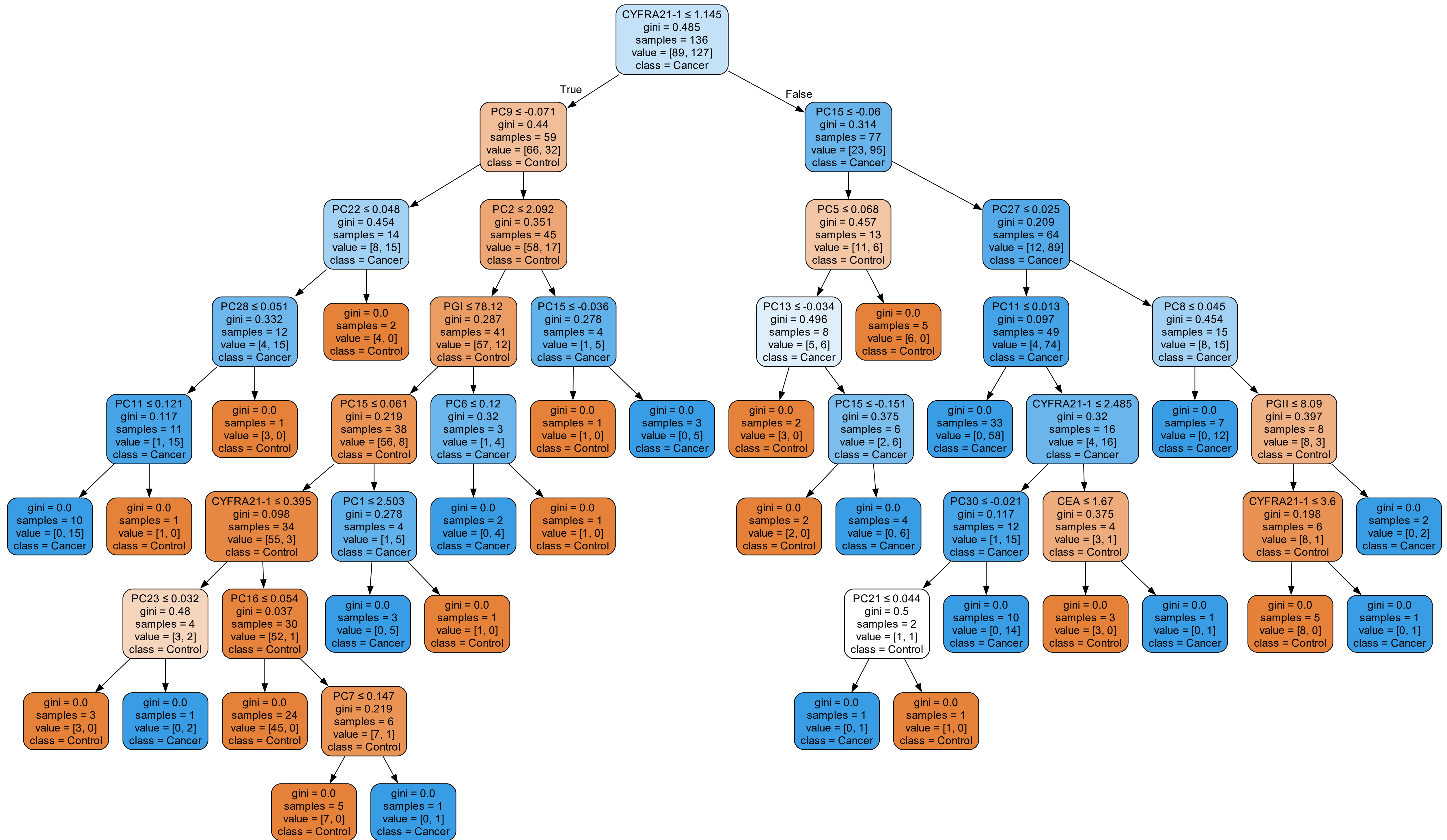

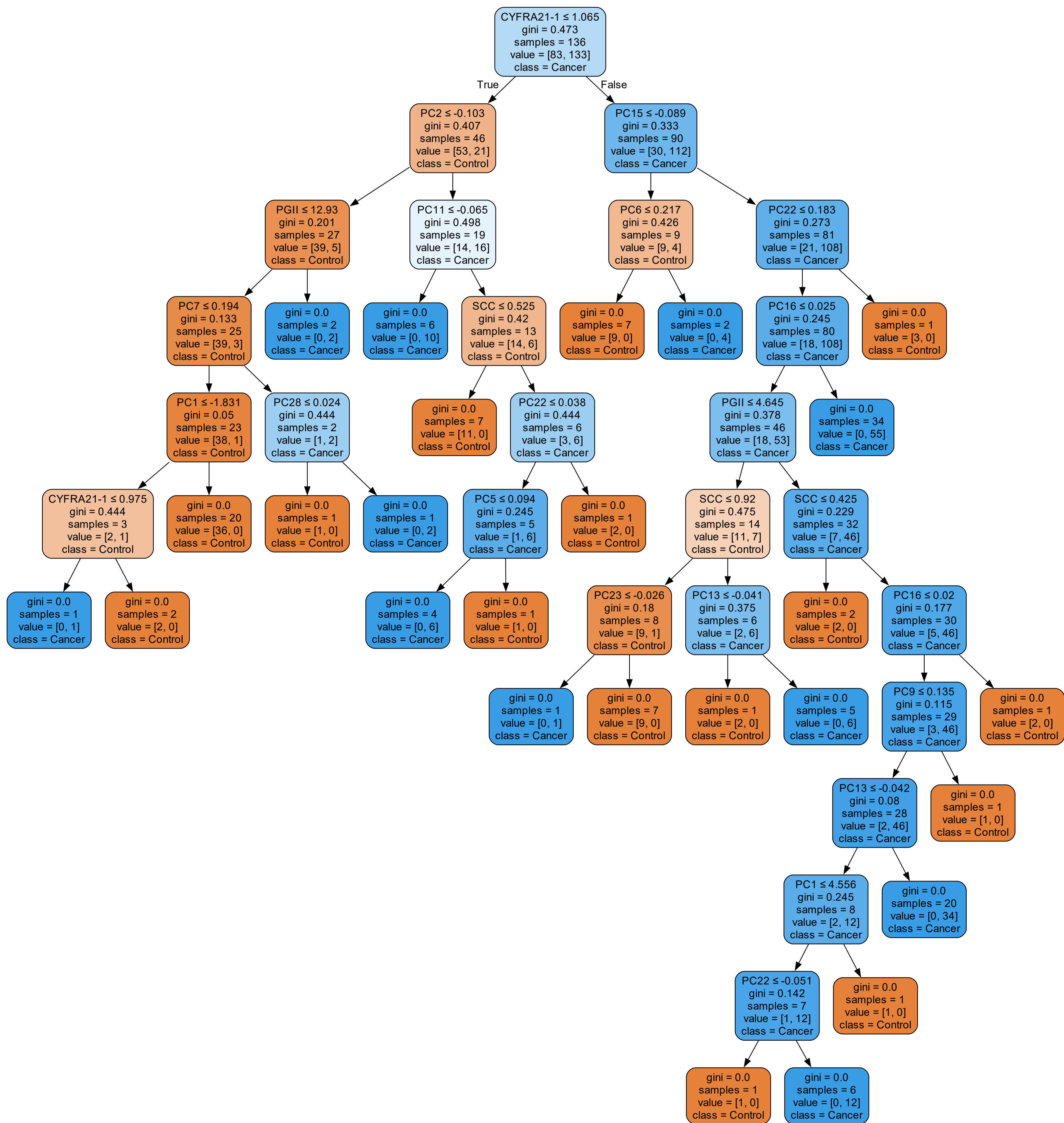

Supplement: Supplementary file 2 — Supplementary Material 2 [file 40364_2025_840_MOESM2_ESM.pdf]
